# Genetic insights into the relationship between immune cell characteristics and ischemic stroke: A bidirectional Mendelian randomization study

**DOI:** 10.1111/ene.16226

**Published:** 2024-02-07

**Authors:** Xia Deng, Shuai Hou, Yanqiang Wang, Haiyan Yang, Chunping Wang

**Affiliations:** ^1^ Shandong Second Medical University Weifang China; ^2^ Department II of Neurology Affiliated Hospital of Shandong Second Medical University Weifang China; ^3^ Emergency Department Yantaishan hospital Yantai China

**Keywords:** causal inference, immunity, ischemic stroke, MR analysis, neuroinflammation

## Abstract

**Background and purpose:**

Ischemic stroke, a major contributor to global disability and mortality, is underpinned by intricate pathophysiological mechanisms, notably neuroinflammation and immune cell dynamics. Prior research has identified a nuanced and often paradoxical link between immune cell phenotypes and ischemic stroke susceptibility. The aim of this study was to elucidate the potential causal links between the median fluorescence intensity (MFI) and morphological parameters (MP) of 731 immune cell types and ischemic stroke risk.

**Methods:**

By analyzing extensive genetic datasets, we conducted comprehensive Mendelian randomization (MR) analyses to discern the genetic correlations between diverse immune cell attributes (MFI and MP) and ischemic stroke risk.

**Results:**

Our study identified key immune cell signatures linked to ischemic stroke risk. Both B cells and T cells, among other immune cell types, have a bidirectional influence on stroke risk. Notably, the regulatory T‐cell phenotype demonstrates significant neuroprotective properties, with all odds ratio (OR) values and confidence intervals (CIs) being less than 1. Furthermore, CD39 phenotype immune cells, particularly CD39+ CD8+ T cells (inverse variance weighting [IVW] OR 0.92, 95% CI 0.87–0.97; *p* = 0.002) and CD39+ activated CD4 regulatory T cells (IVW OR 0.93, 95% CI 0.90–0.97; *p* < 0.001), show notable neuroprotection against ischemic stroke.

**Conclusion:**

This investigation provides new genetic insights into the interplay between various immune cells and ischemic stroke, underscoring the complex role of immune processes in stroke pathogenesis. These findings lay a foundation for future research, which may confirm and expand upon these links, potentially leading to innovative immune‐targeted therapies for stroke prevention and management.

## INTRODUCTION

Ischemic stroke, marked by cerebral blood flow obstruction, is a major global health concern, leading to significant mortality and morbidity [1]. This condition results from a confluence of genetic, environmental, and physiological influences [2]. The immune system's involvement in ischemic stroke pathogenesis has recently gained considerable attention. Immune cells, known to drive inflammatory processes around ischemic events, can critically influence stroke outcomes [3].

Neuroinflammation plays a crucial role in the pathophysiology of ischemic stroke, characterized by a complex inflammatory cascade within the brain [4]. This process is initiated by resident immune cells, augmented by peripheral immune components such as neutrophils, macrophages, and T lymphocytes [4, 5]. A pivotal aspect of this cascade is the disruption of the blood–brain barrier, neuronal damage and cerebral edema, which significantly intensifies inflammatory infiltration following ischemic stroke [6, 7]. A study underscored the association between elevated levels of specific inflammatory mediators, such as CD40 ligand (CD40L) and monocyte chemoattractant protein‐1 (MCP‐1), and the risk of long‐term nonfatal vascular events in ischemic stroke patients [8]. These inflammatory responses are integral to the pathogenesis of ischemic stroke, contributing to the development of thrombosis and atherosclerosis. Furthermore, a direct correlation has been established between various inflammatory markers, including white blood cell count, and prognostic factors such as mortality, cognitive impairment, and functional status [9, 10]. These insights enhance our understanding of the pathophysiological mechanisms underlying ischemic stroke and aid in developing predictive and interventional strategies for its prognosis. A study by Tuttolomondo et al. highlights the significance of inflammation in ischemic stroke pathogenesis [11]. This research suggests that early intervention with high‐dose atorvastatin may improve inflammatory markers and enhance functional recovery in patients, indicating a potential therapeutic pathway targeting early and aggressive inflammation management in stroke cases.

Advancements in genetic research have enabled deeper exploration of the intricate relationship between immune cell diversity and ischemic stroke. Utilizing Mendelian randomization (MR), which employs genetic variants as instrumental variables [12], this study investigates the causal impact of immune cells on stroke risk. MR provides a novel perspective in understanding genetic correlations between an extensive range of immune cells and ischemic stroke risk.

Previous research has underscored complex interactions between various immune cells and the central nervous system in the context of ischemic stroke. Ischemic strokes, the predominant stroke type, initiate a series of biological reactions involving both neuroglial and peripheral immune cells. These interactions significantly affect the progression of brain damage and stroke outcomes. Although neuroglial cell research has been extensive, studies on peripheral immune cells in ischemic stroke have been more limited and narrow in scope, resulting in incomplete insights into the peripheral immune system's role in stroke [13]. The immune response post‐stroke is multifaceted and contradictory. Peripheral immune cells, such as neutrophils, T cells, B cells, dendritic cells, and macrophages, infiltrate the ischemic brain tissue and influence the progression of injury. Their dual roles involve both exacerbating and mitigating ischemic damage, including neuronal apoptosis, repair, differentiation, and neuroregeneration [14, 15]. Moreover, the complexity of immune responses and individual genetic differences pose challenges in understanding these associations.

Originally designed as an alternative to randomized controlled trials [16], MR provides robust causal evidence between exposures and outcomes via genetic variations [17]. The strength of MR lies in its ability to identify causal relationships independent of confounding factors and reverse causality, due to the random allocation of genetic variations prior to disease onset [18]. Through MR, this study aims to conduct an exhaustive analysis of various peripheral immune cell types and characteristics, seeking a more comprehensive understanding of the immunogenetic aspects of ischemic stroke.

## METHODS

### Research design

**FIGURE 1:**
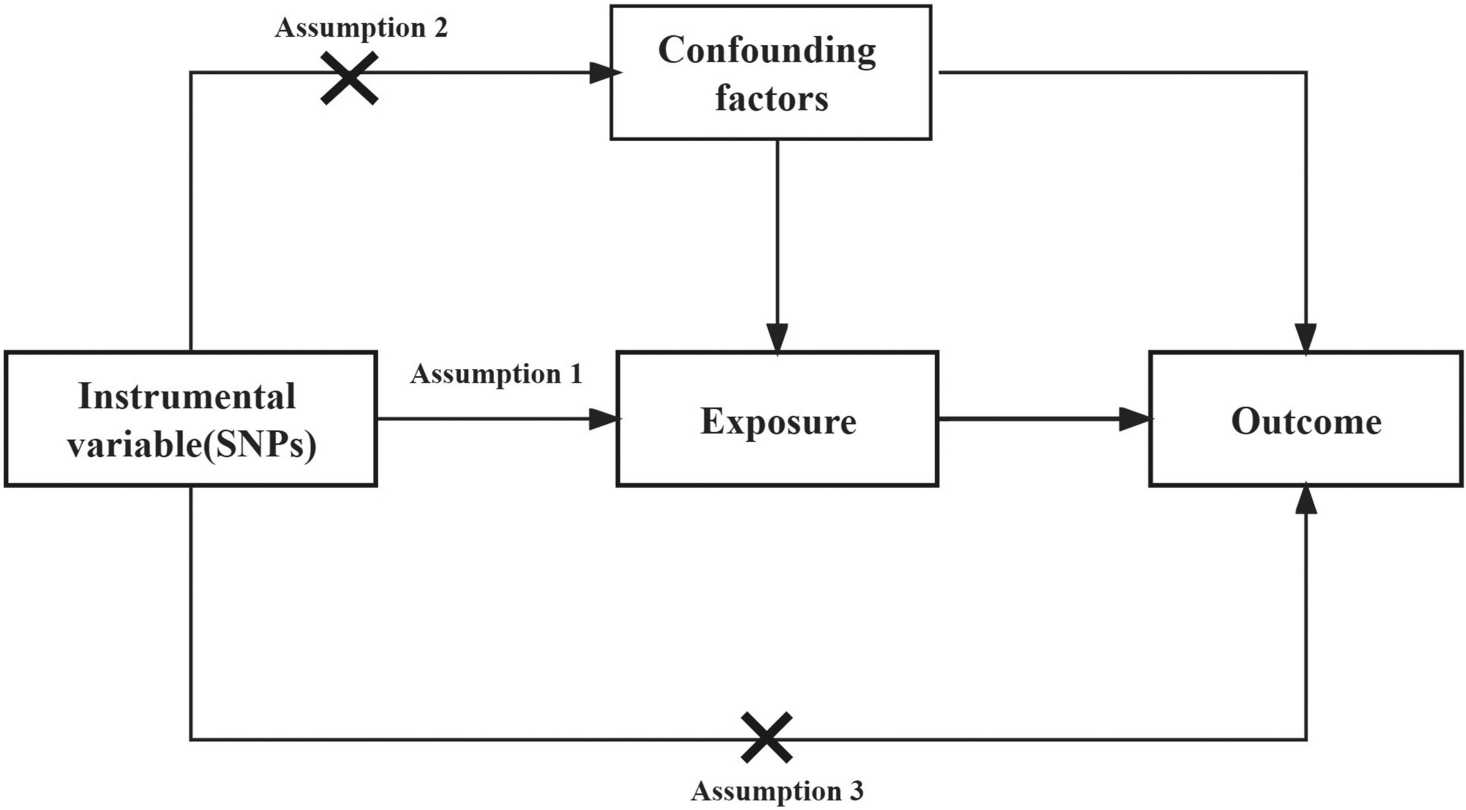
The three core assumptions of Mendelian randomization. SNP, single nucleotide polymorphism.

Our study used a two‐sample MR approach to examine the bidirectional causal link between 421 immunological cell traits and ischemic stroke. Using this method, we sought to determine the causal influence of immune traits on ischemic stroke. MR analysis adheres to three critical assumptions: (i) the genetic variants must be strongly associated with the exposure; (ii) the variants should be independent of confounding factors; and (iii) the variants must affect the outcome solely through the exposure (Figure 1).

### Data sources

Data were sourced from a comprehensive genome‐wide association study (GWAS), offering a detailed statistical summary of immunological traits (accession numbers GCST0001391 to GCST0002121) [19]. This dataset includes 731 distinct immune phenotypes, classified into four groups: absolute cell counts (118 phenotypes), median fluorescence intensity (MFI) of surface antigens (389 phenotypes), morphological parameters (MP; 32 phenotypes), and relative cell counts (192 phenotypes). Our primary focus was on MFI and MP data, offering an extensive overview of immune characteristics. The dataset also details various cell types, including B cells, dendritic cells (CDCs), T cells, regulatory T cells (Tregs), monocytes, granulocytes, myeloid cells, and natural killer cells. For ischemic stroke, data labeled “ischemic stroke, excluding all hemorrhagic” (finn‐b‐I9_STR_EXH) were used, consisting of 10,551 cases and 202,223 controls, totaling 16,380,445 single nucleotide polymorphisms (SNPs; https://gwas.mrcieu.ac.uk/datasets/finn‐b‐I9_STR_EXH/).

### Data processing

In accordance with the foundational assumptions of MR, and drawing on recent literature, our study set the significance threshold for each immune trait's instrumental variables at 5 × 10–6 [20, 21]. The linkage disequilibrium (LD) among SNPs was calculated using the genomes of 1000 individuals from a European population as a reference. We selected SNPs with an LD coefficient (*r* ^2^) below 0.001 and independent SNPs located over 10,000 kilobases apart, aiming to identify SNPs in the European population with independent genetic effects. In our analysis, phenotypic F‐statistics were computed for each instrumental variable to ascertain their strength and reduce potential biases arising from weak instruments. Instrumental variables demonstrating F‐statistics below the threshold of 10 were systematically excluded from further consideration.

### Statistical processing

All analyses were performed using R software, version 4.3.1.

#### Two‐sample Mendelian randomization study and sensitivity analysis

Our study conducted two‐sample MR analyses on various immune cell‐related phenotypes including B cells, T cells, Tregs, and other immune cells. We employed random‐effects inverse variance weighting (IVW), MR‐Egger, and weighted median (WM) methods for these analyses. The primary outcomes were derived using the IVW method, with statistical significance set at *p* < 0.05. IVW gives more weight to variants with smaller variances, hence more precise estimates, by weighting a variant's effect on the outcome inversely to its variance. This method prioritizes more reliable, lower variance estimates and uses the reciprocal of the outcome variance for weighting, excluding the intercept in regression fitting [22]. For pleiotropy assessment, where a single gene influences multiple phenotypes, we utilized MR‐Egger regression. This method includes an intercept evaluation to detect pleiotropy, with a significant intercept (*p* < 0.05) indicating notable horizontal pleiotropy [23]. We also employed Cochran's Q statistic to examine heterogeneity, which measures the consistency of genetic variants' effects on phenotypes. A significant Cochran's Q (*p* < 0.05) suggests considerable heterogeneity in the effects of different genetic variants [24]. For heterogeneous data without pleiotropic effects (*p* > 0.05), the WM approach was used [23].

#### Reverse Mendelian randomization analysis

Using the same MR methods, we investigated whether ischemic stroke causally affects immune cell traits and explored the potential for reverse causation. In this reverse MR analysis, ischemic stroke was considered the exposure factor, and various immune cell traits were treated as outcomes.

## RESULTS

### Forward Mendelian randomization analysis results

**FIGURE 2:**
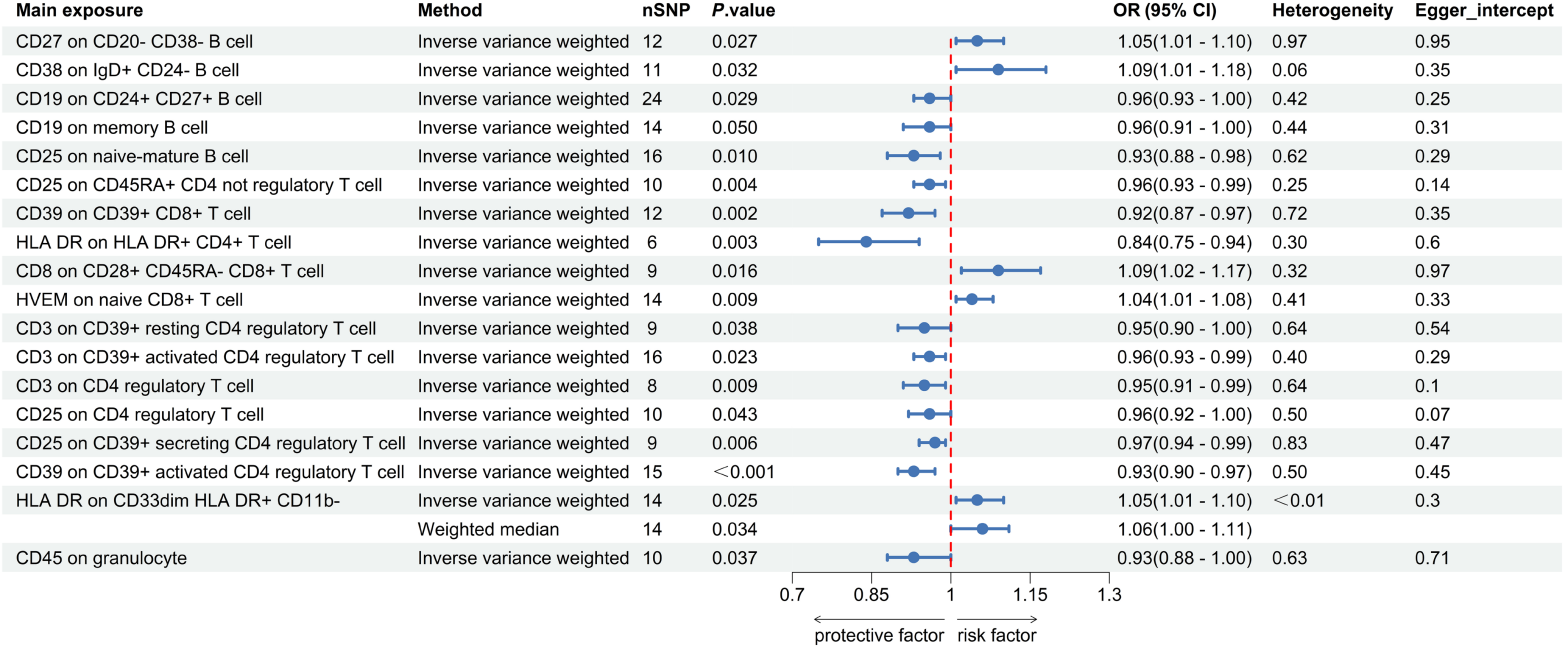
Causal effects of immune cells on ischemic stroke. CI, confidence interval; OR, odds ratio; SNP, single nucleotide polymorphism.

Table S1 provides SNP‐related information. As shown in Figure 2 and Table S2, 19 immune phenotypes suggestive of a link with ischemic stroke were identified, all within MFI traits. No significant associations were found with MP. These phenotypes comprised five T‐cell‐related, six Treg‐related, six B‐cell‐related, one myeloid cell‐related, and one granulocyte‐related trait (Table S3).

For B cells, three traits were linked with increased ischemic stroke risk: B‐cell activating factor receptor (BAFF‐R) on IgD + CD38– unswitched memory B cells (IVW: odds ratio [OR] 1.04, 95% confidence interval [CI] 1.01–1.07; *p* = 0.018), CD27 on CD20– CD38– B cells (IVW: OR 1.05, 95% CI 1.01–1.10; *p* = 0.027), and CD38 on IgD+ CD24– B cells (IVW: OR 1.09, 95% CI 1.01–1.18; *p* = 0.032). Three traits were found to be protective: CD19 on CD24+ CD27+ B cells (IVW: OR 0.96, 95% CI 0.93–1.00), CD19 on memory B cells (IVW: OR 0.96, 95% CI 0.91–1.00), and CD26 on immature‐mature B cells (IVW: OR 0.93, 95% CI 0.88–0.98).

In T cells, CD8 on CD28+ CD45RA– CD8+ T cells (IVW: OR 1.09, 95% CI 1.02–1.17; *p* = 0.016) and herpes virus entry mediator (HVEM) on immature CD8+ T cells (IVW: OR 1.04, 95% CI 1.01–1.08; *p* = 0.009) were associated with increased stroke risk. Three traits showed protective effects: CD25 on CD45RA+ CD4 non‐regulatory T cells (IVW: OR 0.96, 95% CI 0.93–0.99; *p* = 0.004), CD39 on CD39+ CD8+ T cells (IVW: OR 0.92, 95% CI 0.87–0.97; *p* = 0.002), and Human leucocyte antigen (HLA) DR on HLA DR+ CD4+ T cells (IVW: OR 0.84, 95% CI 0.75–0.94; *p* = 0.003).

Remarkably, all seven significant traits in Tregs were neuroprotective, including CD3 on CD39+ resting and activated CD4 Tregs, CD25 on CD4 Tregs, and CD39 on CD39+ secreting CD4 Tregs, with respective ORs and CIs detailed in Figure 2.

For other immune cells, CD45 on granulocytes showed neuroprotective effects (IVW: OR 0.93, 95% CI 0.88–1.00; *p* = 0.037), while HLA DR on CD33dim HLA DR+ CD11b– (IVW: OR 1.05, 95% CI 1.01–1.10; *p* = 0.025) and CD62L on CD62L+ plasmacytoid dendritic cells (IVW: OR 1.06, 95% CI 1.00–1.13; *p* = 0.042) were linked with increased ischemic stroke risk.

No significant heterogeneity or pleiotropy was found in B cells, T cells, or monocytes (*p* > 0.05). However, for myeloid cells, notable heterogeneity was observed in CD33dim HLA DR+ CD11b– for HLA DR (Cochran's Q *p* = 0.004). Following Burgess and Thompson's recommendation for heterogeneous data without pleiotropic effects (Cochran's Q *p* < 0.05), the WM method indicated a potential increase in stroke risk for CD33dim HLA DR+ CD11b– (WM: OR 1.06, 95% CI 1.00–1.11; *p* = 0.034). CD28 on CD4 Tregs and CD62L on CD62L+ plasmacytoid dendritic cells, showing significant pleiotropy (*p* < 0.05), were excluded from our analysis (Figures S1 and S2 and Table S4).

### Reverse Mendelian randomization analysis results

**FIGURE 3:**
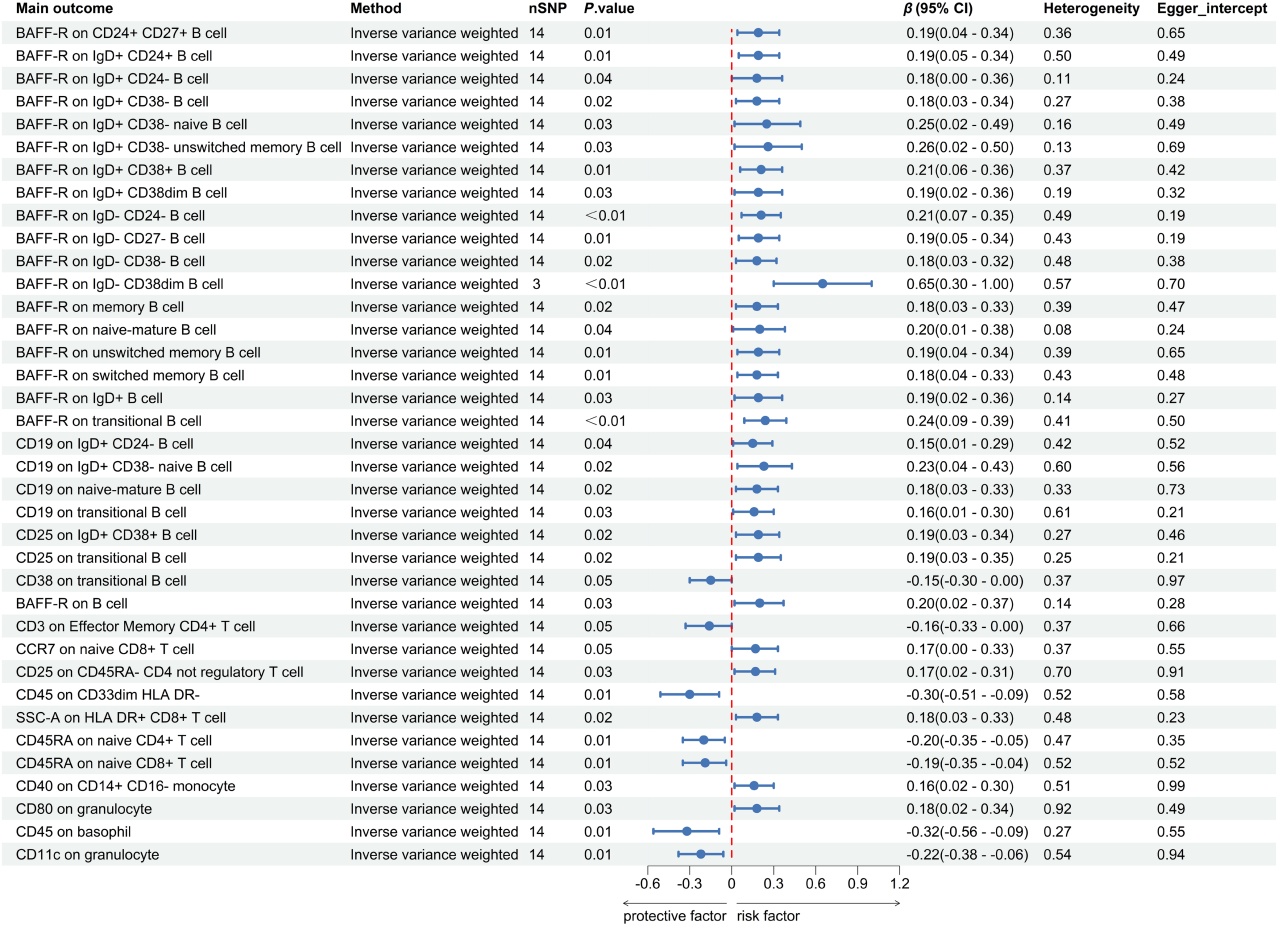
Causal effects of ischemic stroke on immune cells. CI, confidence interval; OR, odds ratio; SNP, single nucleotide polymorphism.

Table S5 provides SNP‐related information. Figure 3 details 37 immune phenotypes identified as potentially influenced by ischemic stroke. Of these, 26 are associated with B cells, seven with T cells, three with granulocytes, and one with monocytes (Tables S6 and S7).

In the case of B cells, except for a decrease in CD38 on transitional B cells post‐ischemic stroke (IVW: *β* = −0.15, 95% CI −0.30–0.00; *p* = 0.047), the other 24 traits showed an increase (Figure 3 and Table S6). Notably, BAFF‐R on IgD+ CD38− unswitched memory B cells (IVW: *β* = 0.26, 95% CI 0.02–0.50; *p* = 0.034) demonstrated statistical significance, indicating a possible reverse causal relationship.

For T cells, the onset of ischemic stroke was associated with an increase in CC‐chemokine receptor 7 (CCR7) on immature CD8+ T cells (IVW: *β* = 0.17, 95% CI 0.00–0.33; *p* = 0.047), CD25 on CD45RA– CD4 non‐regulatory T cells (IVW: *β* = 0.17, 95% CI 0.02–0.31; *p* = 0.025), and side scatter (SSC)‐A on HLA DR+ CD8+ T cells (IVW: *β* = 0.18, 95% CI 0.03–0.33; *p* = 0.020). Decreases were noted in CD3 on effector memory CD4+ T cells (IVW: *β* = −0.16, 95% CI −0.33–0.00; *p* = 0.050), CD45 on CD33dim HLA DR− (IVW: *β* = −0.30, 95% CI −0.51 to −0.09; *p* = 0.005), CD45RA on immature CD4+ T cells (IVW: *β* = −0.20, 95% CI −0.35 to −0.05; *p* = 0.008), and CD45RA on immature CD8+ T cells (IVW: *β* = −0.19, 95% CI  −0.35 to −0.04; *p* = 0.014).

Regarding other immune cells, increases were observed in CD40 on CD14+ CD16− monocytes (IVW: *β* = 0.16, 95% CI 0.02–0.30; *p* = 0.030) and CD80 on granulocytes (IVW: *β* = 0.18, 95% CI 0.02–0.34; *p* = 0.029). Conversely, decreases were noted in CD45 on basophilic granulocytes (IVW: *β* = −0.32, 95% CI −0.56 to −0.09; *p* = 0.006) and CD11c on granulocytes (IVW: *β* = −0.22, 95% CI −0.38 to −0.06; *p* = 0.007).

No significant heterogeneity or pleiotropy was found (Table S8 and Figures S3 and S4).

## DISCUSSION

This study represents a pioneering effort to systematically delineate the causal link between immune phenotypes and ischemic stroke. Leveraging data from GWASs, we evaluated 421 immune cell phenotypes for their association with ischemic stroke. Our MR analysis pinpointed 23 immune cell phenotypes that potentially affect the risk of ischemic stroke. Out of these, eight phenotypes, excluding those exhibiting reverse causation, are believed to contribute to the onset of ischemic stroke, whereas 15 exhibit neuroprotective properties.

B cells play a multifaceted role in the immune response to stroke, displaying both detrimental and restorative effects. After ischemic stroke, the compromised blood–brain barrier may permit B cells and other peripheral immune cells to enter ischemic brain tissue [25, 26]. These cells are crucial in modulating the progression of ischemic brain injury and influencing the secondary inflammatory response post‐stroke [15, 27]. However, studies by Schuhmann et al. indicate that targeting B cells does not significantly alter lesion volume or functional outcomes during the acute phase of experimental stroke [28]. Our findings mirror this complexity, revealing that while some B‐cell phenotypes, such as CD19 on CD24+ CD27+ B cells, CD19 on memory B cells, and CD26 on immature‐mature B cells, offer neuroprotection, others such as absolute B‐cell counts, CD27 on CD20–CD38– B cells, and CD38 on IgD+ CD24– B cells, are associated with increased stroke risk. This underscores the need for further research into the nuanced role of B‐cell‐related phenotypes in stroke.

Similarly, T cells are primarily known for their pro‐inflammatory effects in ischemic stroke, invading through the blood–brain barrier, choroid plexus, and meninges [29, 30, 31, 32]. They exacerbate post‐stroke inflammation by producing inflammatory cytokines such as interferon (IFN)‐γ, interleukin (IL)‐21, tumor necrosis factor (TNF), and IL‐17 [33, 34, 35, 36, 37]. Interactions between T lymphocytes and platelets can also intensify ischemic stroke progression by worsening microvascular dysfunction and inflammation [38]. Nonetheless, our analysis suggests a dual role for T cells in stroke pathogenesis, attributable to their regulatory function in inflammation, a critical factor in stroke. The inflammatory response following ischemic stroke is complex, contributing to both brain injury and repair, with T cells playing a pivotal role in balancing these outcomes.

Our findings robustly demonstrate that numerous immune characteristics instrumental in improving ischemic stroke are present in Tregs. Earlier studies indicate that Tregs initially mitigate acute brain injury by moderating the immune response, largely due to their immunosuppressive capabilities and interactions with other immune cells, such as neutrophils [39, 40]. On a molecular level, Tregs secrete cytokines including IL‐10, TGF‐β, and IL‐35. These cytokines suppress the activity of other immune cells, thereby fostering an anti‐inflammatory environment that safeguards neural tissue [41, 42, 43]. Tregs employ surface molecules such as PD‐L1, CTLA‐4, and galectin‐1 for essential direct cell‐to‐cell interactions, which are pivotal for their immunosuppressive role and in maintaining the integrity of the blood–brain barrier [44, 45, 46]. Additionally, Tregs are capable of modulating astrocyte responses. This modulation involves diminishing the formation of reactive astrocytes and reducing inflammatory glial scarring, partly through bimodal protein activity and potential IL‐10 factors, thus promoting a milieu conducive to neuronal survival [47, 48, 49]. Moreover, Tregs secrete neurotrophic factors such as brain‐derived neurotrophic factor, which plays a crucial role in neural repair and regeneration following a stroke [50].

Our study distinctly identified that CD39 phenotypes across various immune cells exert a neuroprotective impact on ischemic stroke. Research demonstrates that CD39 catalyzes the conversion of extracellular adenosine triphosphate (ATP) to adenosine monophosphate (AMP), followed by CD73 transforming AMP into the immunosuppressive nucleoside adenosine (ADO) [51]. This enzymatic process, with CD39 as a key regulator, serves as an immune modulator, transitioning the cellular milieu from an ATP‐driven pro‐inflammatory state to an ADO‐predominant anti‐inflammatory state, thereby playing a vital role in immune regulation [51]. The absence of CD39 correlates with reduced liver insulin sensitivity and increased levels of pro‐inflammatory cytokines, including IL‐6, IL‐1β, TNF‐α, and IFN‐γ, intensifying the inflammatory response [52, 53]. Our findings further substantiate that CD39 presence in CD39+ CD8+ T cells and CD39+ activated CD4 Tregs is inversely associated with stroke occurrence. Notably, CD39 on CD39+ activated CD4 Tregs emerges as a pivotal immune element for neuroprotection in ischemic stroke. This research delineates specific immune characteristics, underscoring the potential for targeted therapy involving Tregs in future ischemic stroke interventions.

Our study leverages the MR approach to discern a potential causal relationship between immune phenotypes and ischemic stroke risk, moving beyond simple associative studies. MR significantly mitigates confounding factors by using genetic variants as instrumental variables, a crucial technique in overcoming common obstacles in observational studies [54]. This method offers a comprehensive understanding of immunological mechanisms, specifically examining the impact of immune cells such as B cells, T cells, and Tregs on stroke risk. Importantly, it aids in identifying immune phenotypes that may increase or decrease stroke risk, providing vital insights to aid the development of targeted therapies. This approach is essential for enabling more precise and personalized strategies in stroke prevention and treatment.

This study has some limitations. As a pioneering study in mapping the entire causal relationship between the immune landscape and ischemic stroke, it is inherently exploratory. Our goal was to examine as many immune phenotypes as possible that could affect stroke onset, thereby setting a course for future research. Consequently, we did not apply multiple testing corrections. The study is underpinned by an MR analysis, using SNP data sourced from the OPEN GWAS database. The aggregate nature of these data precludes access to individual‐level details, which consequently limits our capacity to categorize ischemic stroke into more specific subtypes. Nevertheless, the extensive genetic variant coverage in our dataset establishes a robust basis for exploring the general association between immune cell characteristics and ischemic stroke risk. It is projected that future research will increasingly focus on the classification of ischemic stroke. Moreover, the issue of pleiotropy is addressed based on the statistical significance of pleiotropy tests alone, without excluding pleiotropic instrumental variables. While these limitations increase the risk of type I errors, they decrease the chance of type II errors. This approach allows for a more expansive and systematic exploration of the immune landscape in ischemic stroke, creating a pathway for future studies on precise targets.

Ultimately, our study offers a detailed and systematic insight into the immune dynamics in ischemic stroke. It establishes a foundation for future precision medicine endeavors, focusing on specific immune phenotypes for stroke prevention and treatment. Our research marks a significant step in elucidating the intricate relationship between the immune system and ischemic stroke, providing crucial directions for subsequent investigations.

## AUTHOR CONTRIBUTIONS

**Shuai Hou:** Writing – original draft; writing – review and editing; conceptualization; methodology; software; data curation; validation. **Xia Deng:** Writing – original draft; writing – review and editing; view and editing; visualization; formal analysis; project administration; supervision. **Haiyan Yang:** Writing – review and editing; supervision; formal analysis; project administration; funding acquisition. **Chunping Wang:** Writing – review and editing; supervision; formal analysis; project administration; funding acquisition.

## FUNDING INFORMATION

This research was supported by the funding of the Key Research and Development Program of Shandong Province (grant no. 2023RKY07003), Yuan Du Scholars and Weifang Key Laboratory.

## CONFLICT OF INTEREST STATEMENT

The authors declare that the research was conducted in the absence of any commercial or financial relationships that could be construed as a potential conflict of interest.

## Supporting information


Figures S1–S4

Table S1

Table S2

Table S3

Table S4

Table S5

Table S6

Table S7

Table S8

## Data Availability

The original contributions presented in the study are included in the supporting information (Figures S1–S4 and Tables [Link], [Link]), further inquiries can be directed to the corresponding authors.
