# Supplementary material for: Genetic insights into the relationship between immune cell characteristics and ischemic stroke: A bidirectional Mendelian randomization study
Source: Eur J Neurol. 2024 Feb 7;31(5):e16226. doi: 10.1111/ene.16226 (PMC11236043; doi:10.1111/ene.16226)
Supplement: Supplementary file 1 — Figures S1–S4 [file ENE-31-e16226-s009.pdf]

Supplementary Figures:

Supplementary Figure 1. Sensitivity analysis of forward Mendelian randomization (scatter plot).

Supplementary Figure 2. Sensitivity analysis of forward Mendelian randomization (Funnel plot).

Supplementary Figure 3. Sensitivity analysis of reverse Mendelian randomization (scatter plot).

Supplementary Figure 4. Sensitivity analysis of reverse Mendelian randomization (Funnel plot).

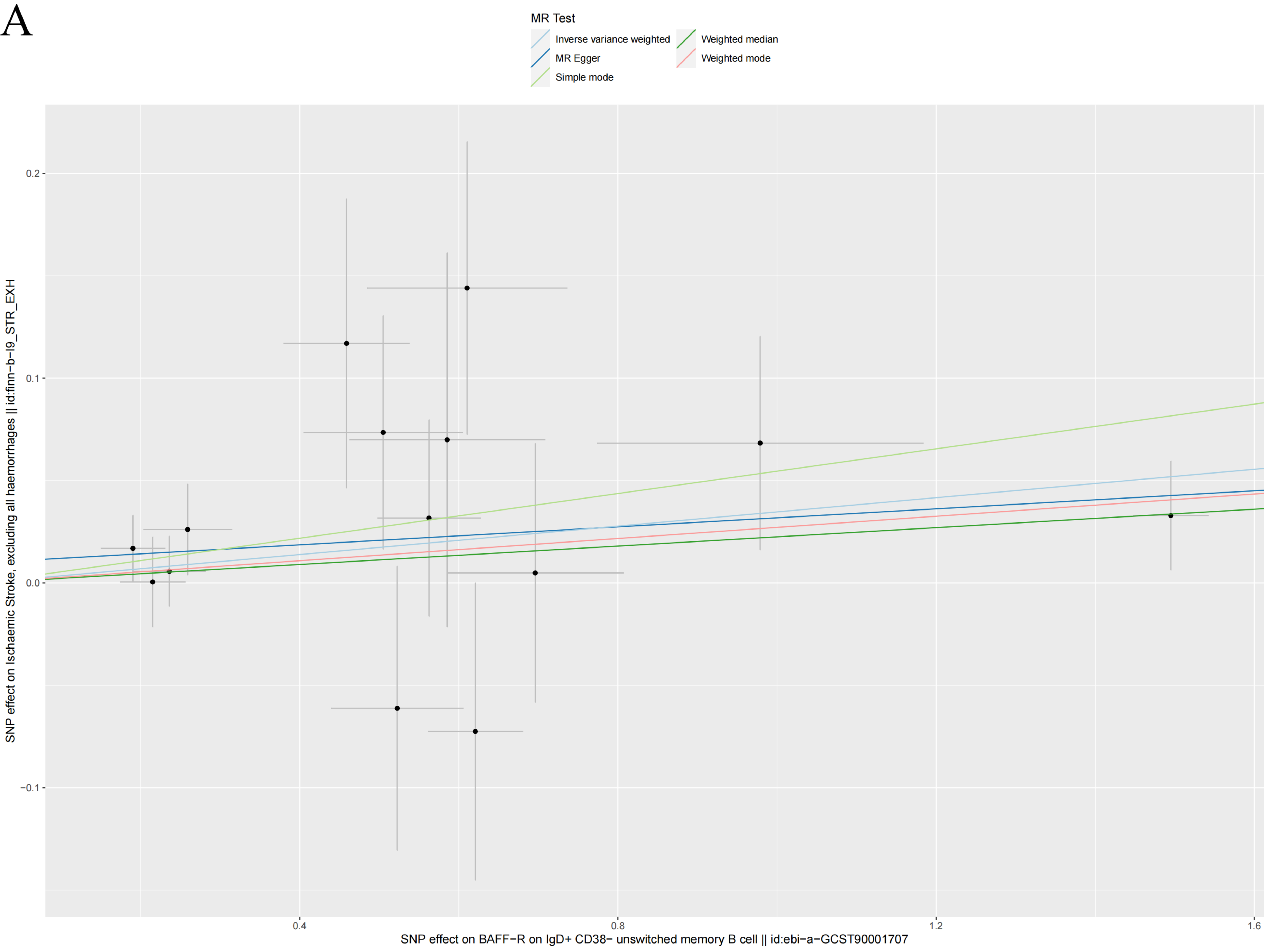
A
MR Test
Inverse variance weighted
MR Egger
Simple mode
Weighted median
Weighted mode
0.2
0.1
0.0
−0.1
0.4
0.8
1.2
1.6
SNP effect on BAFF−R on IgD+ CD38− unswitched memory B cell || id:ebi−a−GCST90001707
SNP effect on Ischaemic Stroke, excluding all haemorrhages || id:finn−b−I9_STR_EXH

B

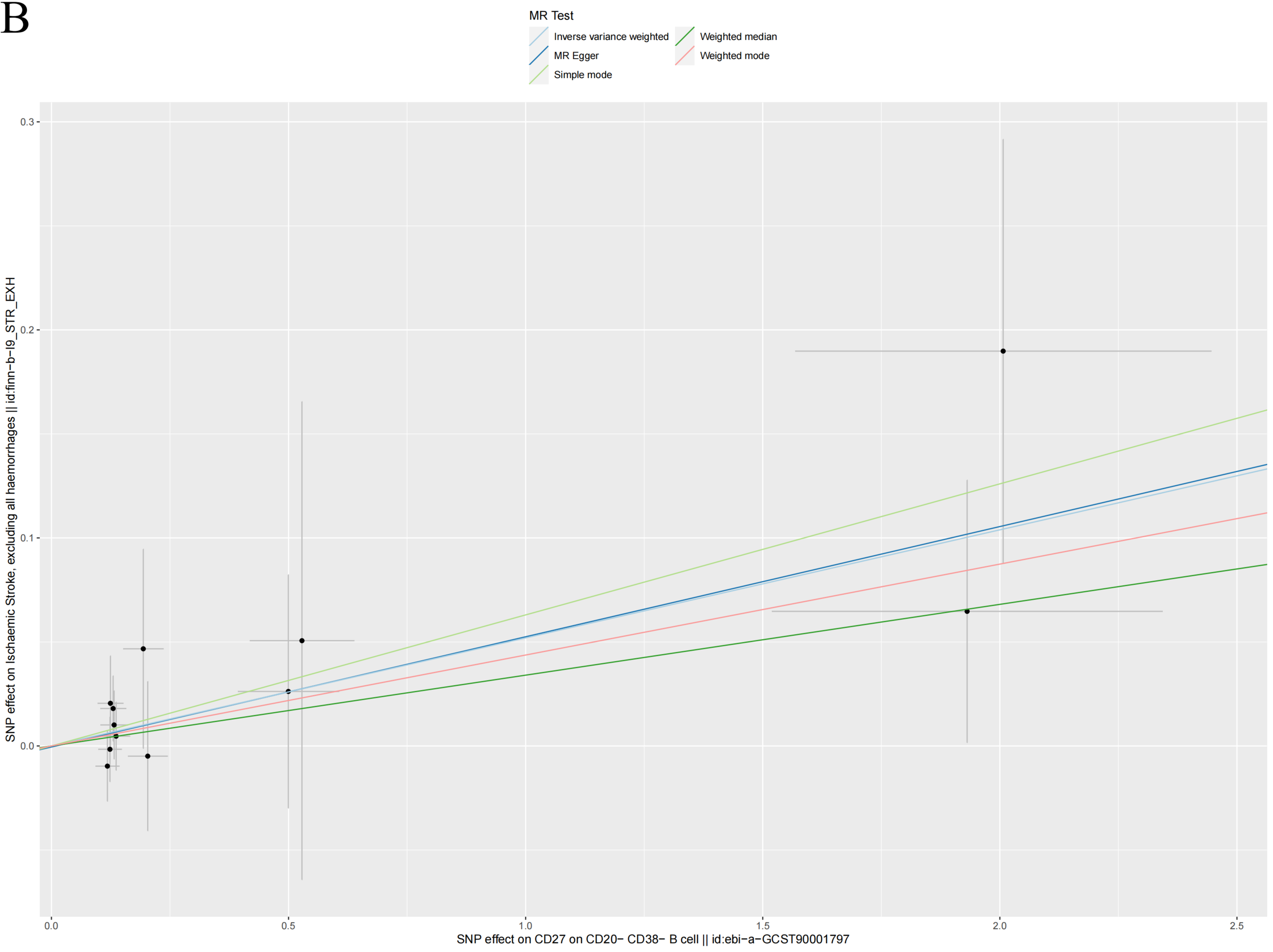
MR Test
Inverse variance weighted
MR Egger
Simple mode
Weighted median
Weighted mode
0.3
0.2
0.1
0.0
0.0
0.5
1.0
1.5
2.0
2.5
SNP effect on Ischaemic Stroke, excluding all haemorrhages || id:finn-b-I9_STR_EXH
SNP effect on CD27 on CD20- CD38- B cell || id:ebi-a-GCST90001797

C

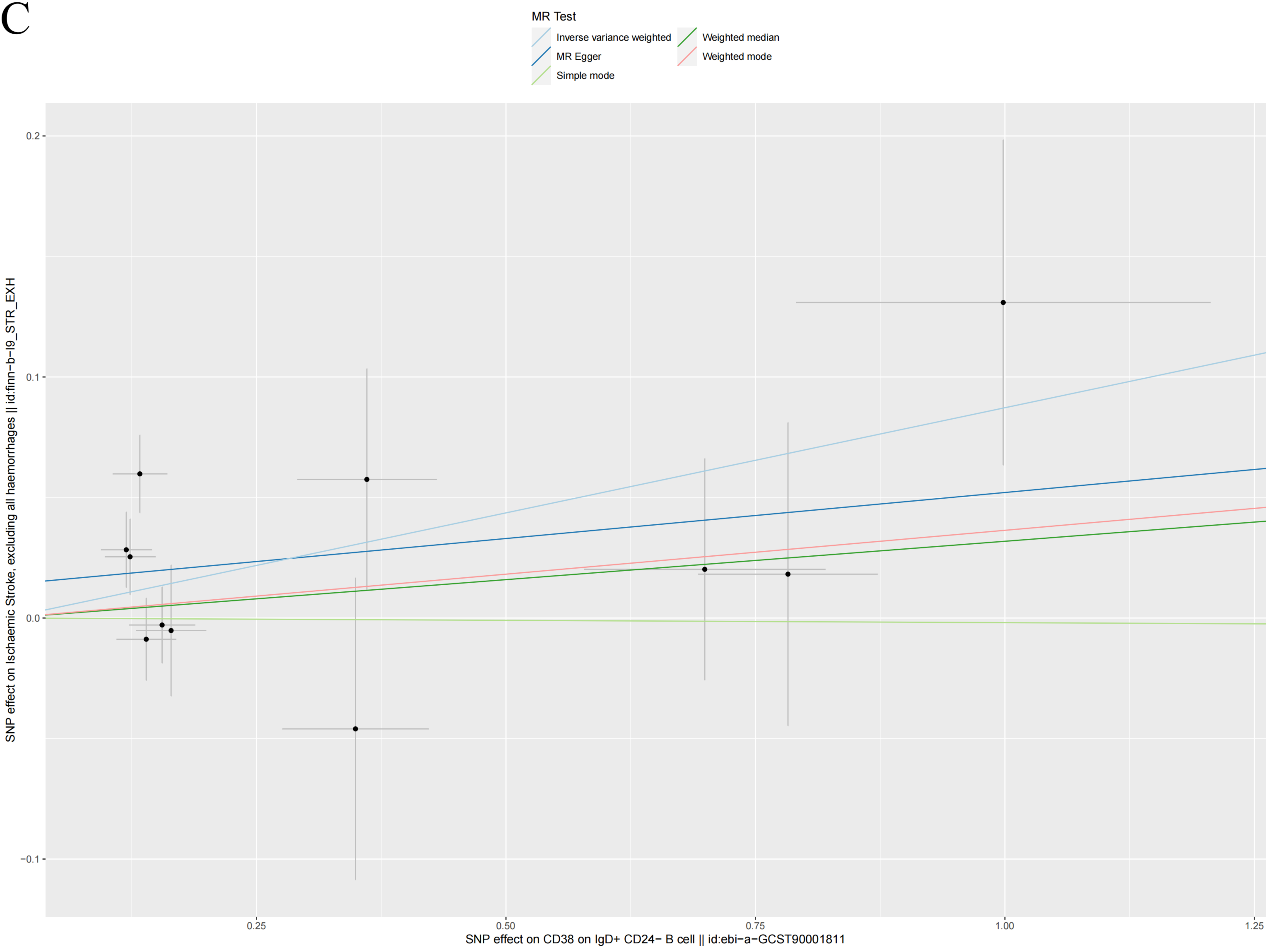
MR Test
Inverse variance weighted
MR Egger
Simple mode
Weighted median
Weighted mode
0.2
0.1
0.0
−0.1
0.25
0.50
0.75
1.00
1.25
SNP effect on Ischaemic Stroke, excluding all haemorrhages || id:finn-b-I9_STR_EXH
SNP effect on CD38 on IgD+ CD24− B cell || id:ebi-a-GCST90001811

D

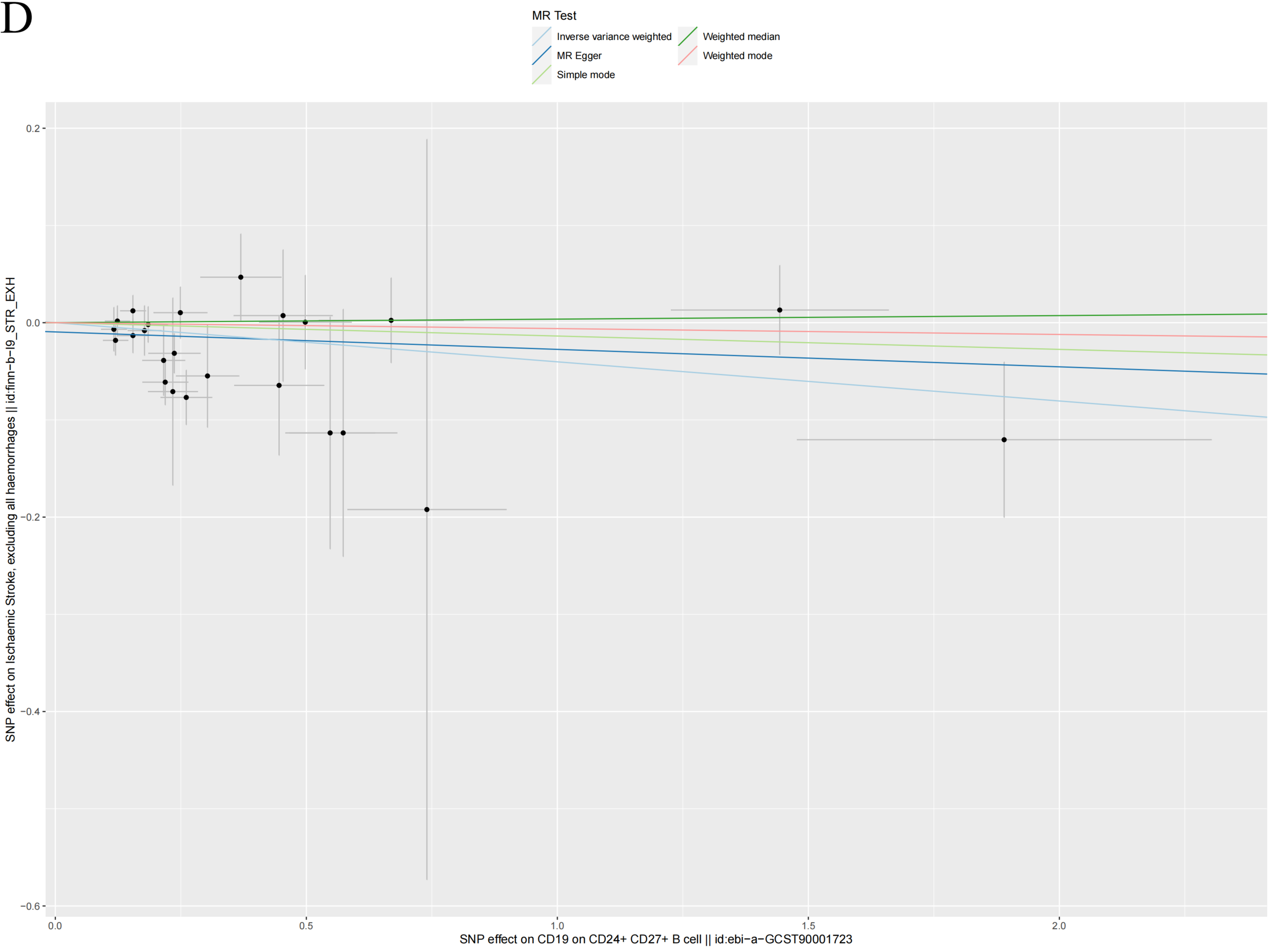

MR Test
Inverse variance weighted
MR Egger
Simple mode
Weighted median
Weighted mode
0.2
0.0
−0.2
−0.4
−0.6
0.0
0.5
1.0
1.5
2.0
SNP effect on Ischaemic Stroke, excluding all haemorrhages || id:finn−b−I9_STR_EXH
SNP effect on CD19 on CD24+ CD27+ B cell || id:ebi−a−GCST90001723

E

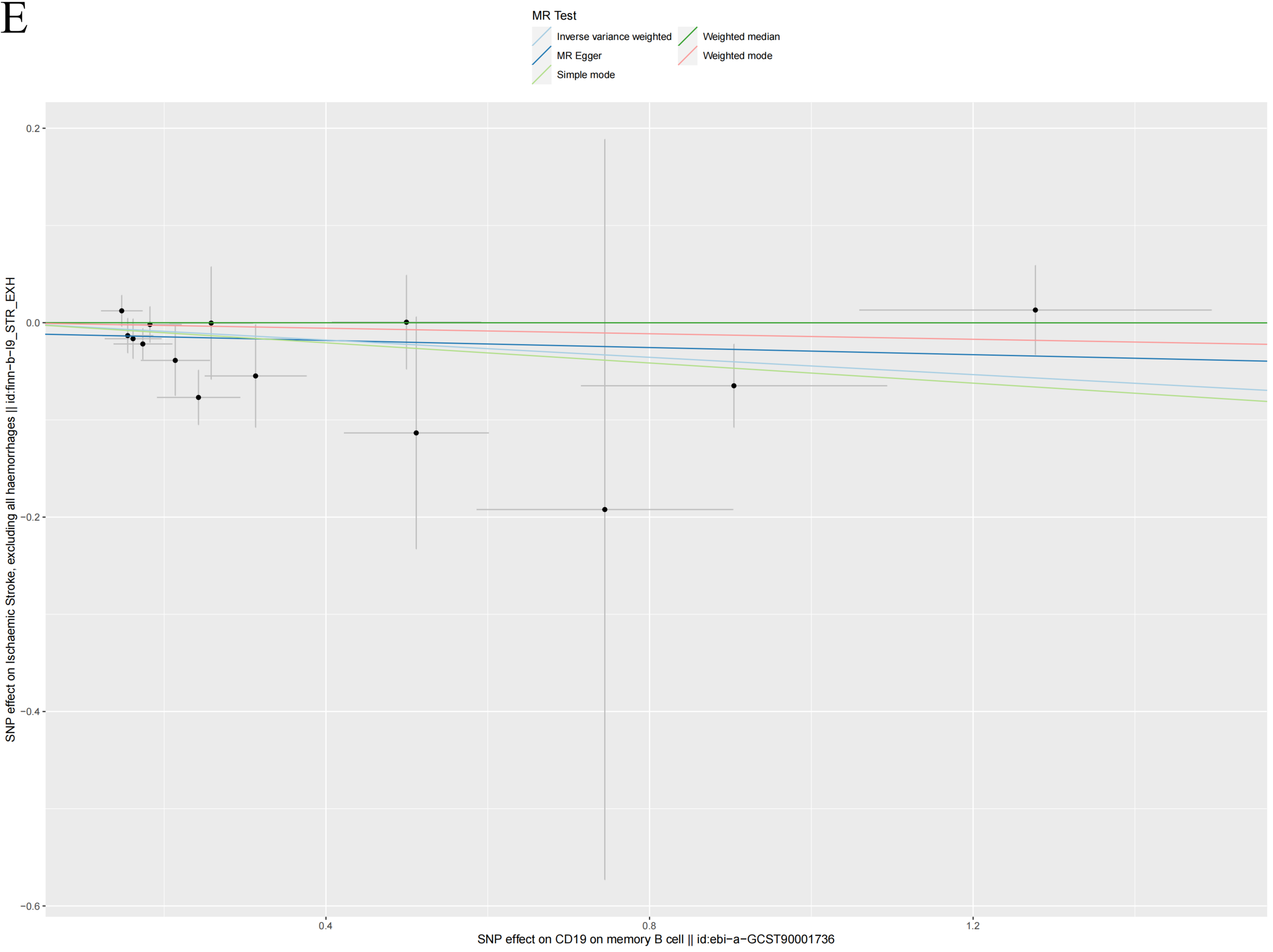

MR Test
Inverse variance weighted
MR Egger
Simple mode
Weighted median
Weighted mode
0.2
0.0
−0.2
−0.4
−0.6
0.4
0.8
1.2
SNP effect on CD19 on memory B cell || id:ebi−a−GCST90001736
SNP effect on Ischaemic Stroke, excluding all haemorrhages || id:finn−b−I9_STR_EXH

F

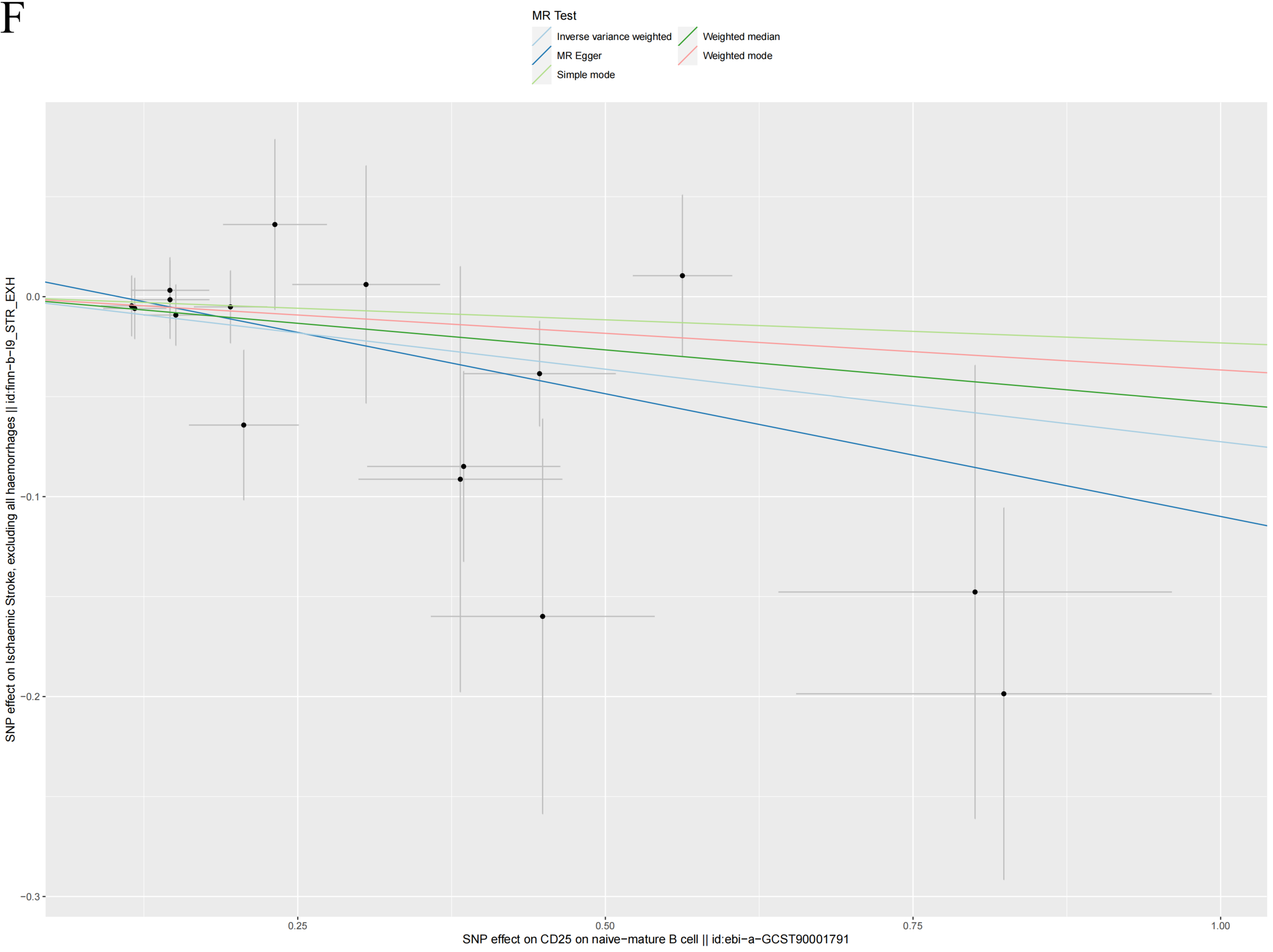

MR Test
Inverse variance weighted
MR Egger
Simple mode
Weighted median
Weighted mode
0.0
−0.1
−0.2
−0.3
SNP effect on Ischaemic Stroke, excluding all haemorrhages || id:finn−b−I9_STR_EXH
0.25
0.50
0.75
1.00
SNP effect on CD25 on naive−mature B cell || id:ebi−a−GCST90001791

G

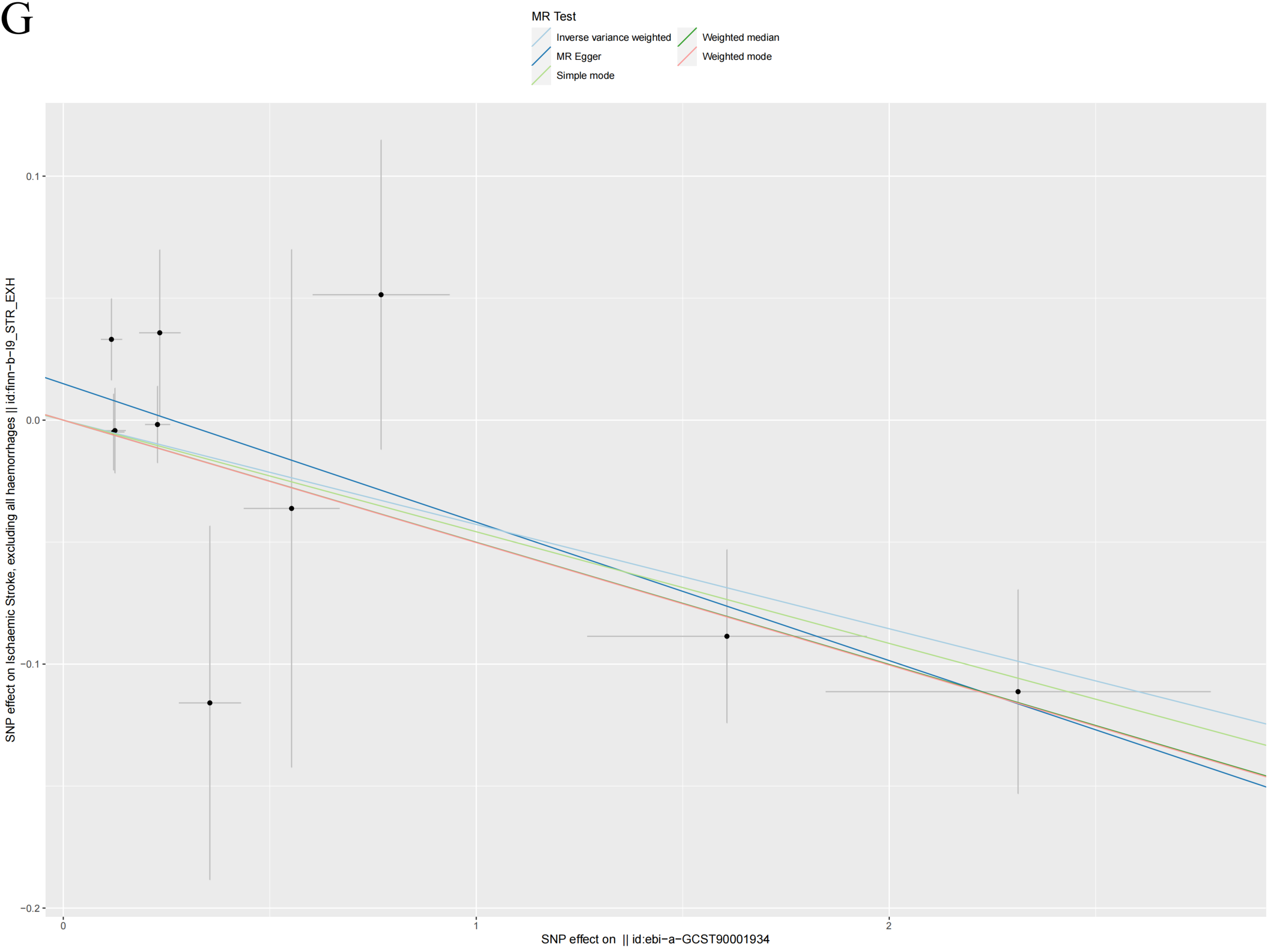
MR Test
Inverse variance weighted
MR Egger
Simple mode
Weighted median
Weighted mode
0.1
0.0
−0.1
−0.2
0
1
2
SNP effect on Ischaemic Stroke, excluding all haemorrhages || id:finn-b-I9_STR_EXH
SNP effect on || id:ebi-a-GCST90001934

H

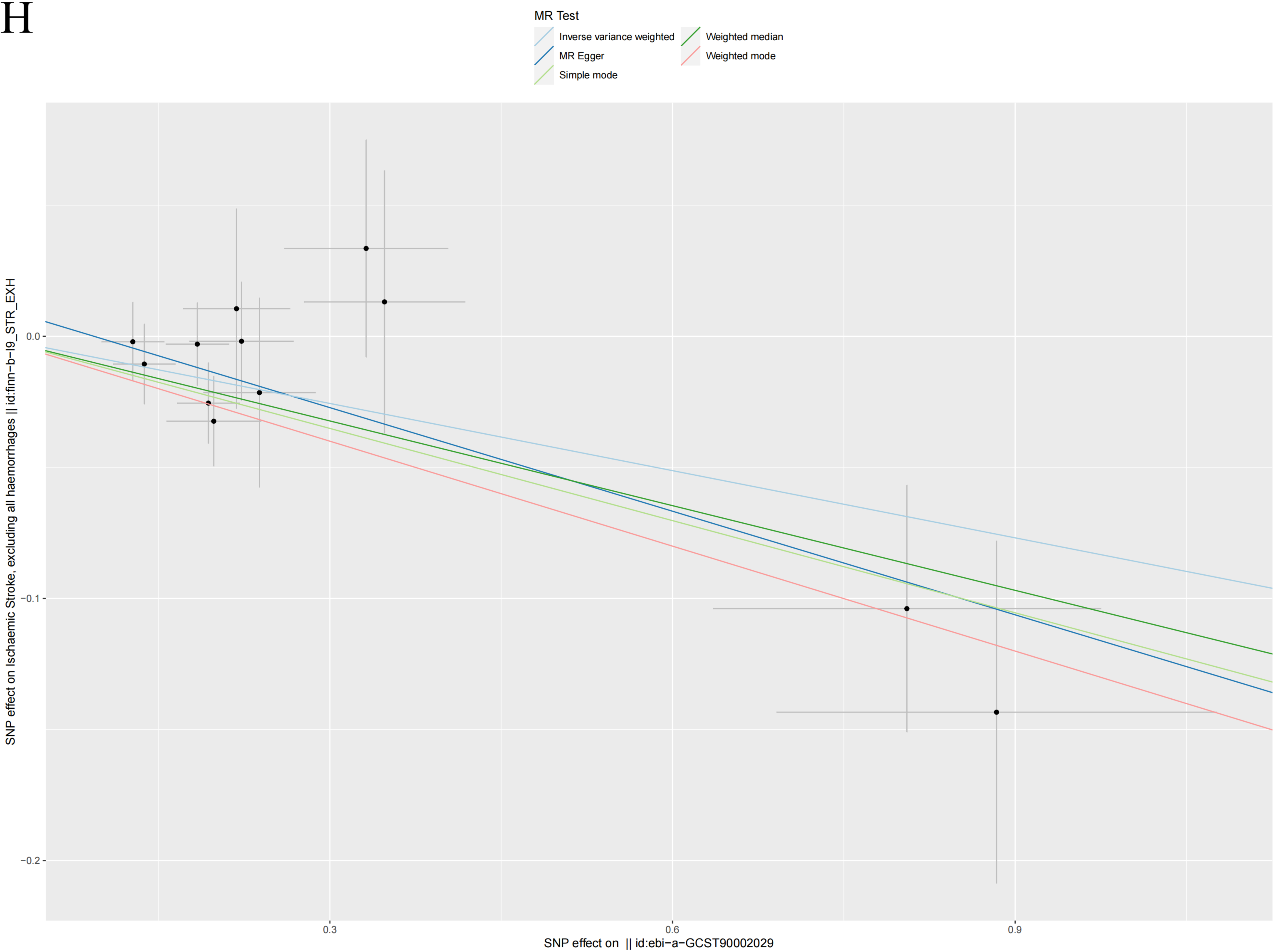
MR Test
Inverse variance weighted
MR Egger
Simple mode
Weighted median
Weighted mode
SNP effect on Ischaemic Stroke, excluding all haemorrhages || id:finn-b-I9_STR_EXH
0.0
−0.1
−0.2
0.3
0.6
0.9
SNP effect on || id:ebi-a-GCST90002029

I

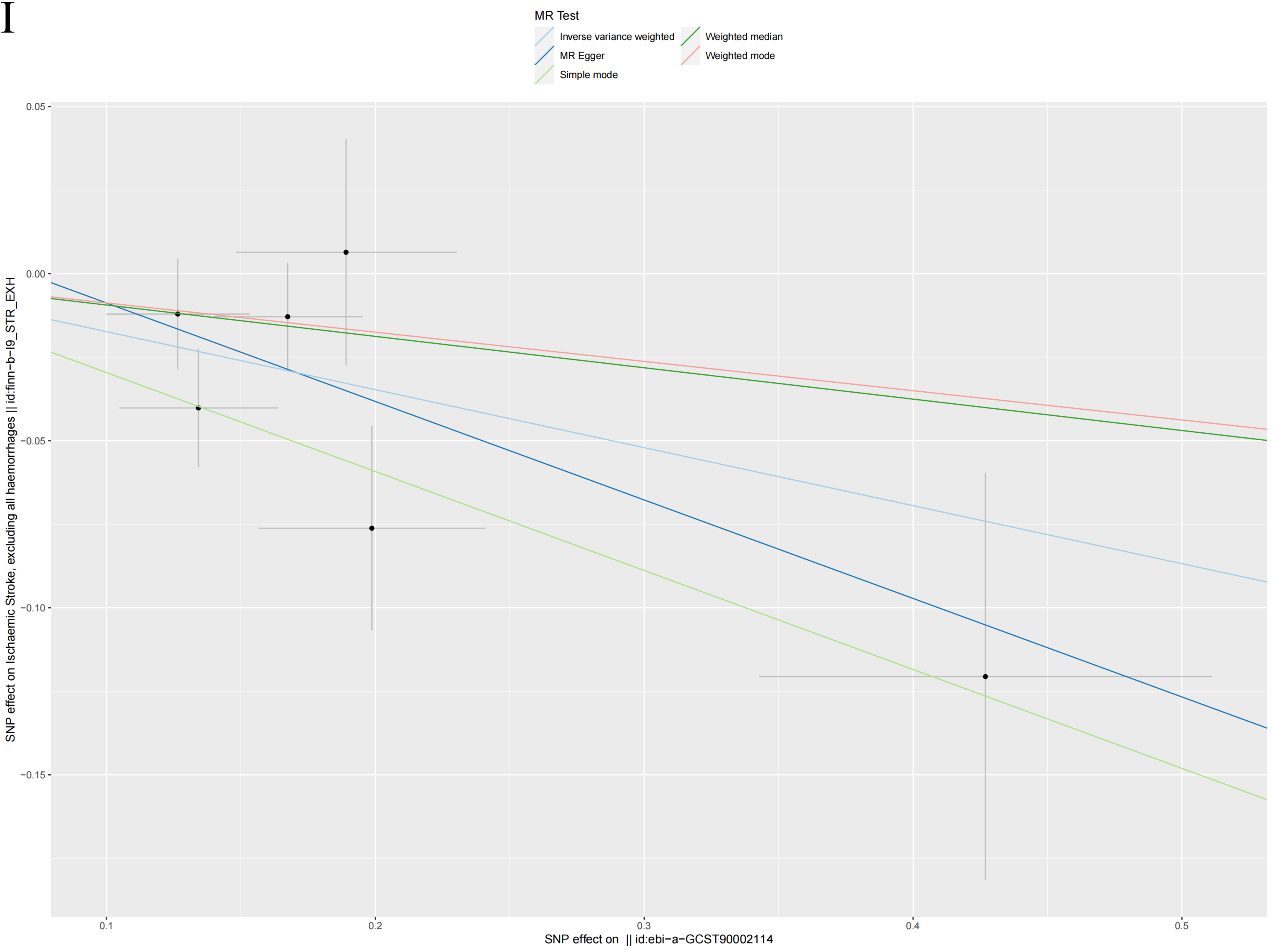
MR Test
Inverse variance weighted
MR Egger
Simple mode
Weighted median
Weighted mode
0.05
0.00
−0.05
−0.10
−0.15
0.1
0.2
0.3
0.4
0.5
SNP effect on Ischaemic Stroke, excluding all haemorrhages || id:finn−b−I9_STR_EXH
SNP effect on || id:ebi−a−GCST90002114

J

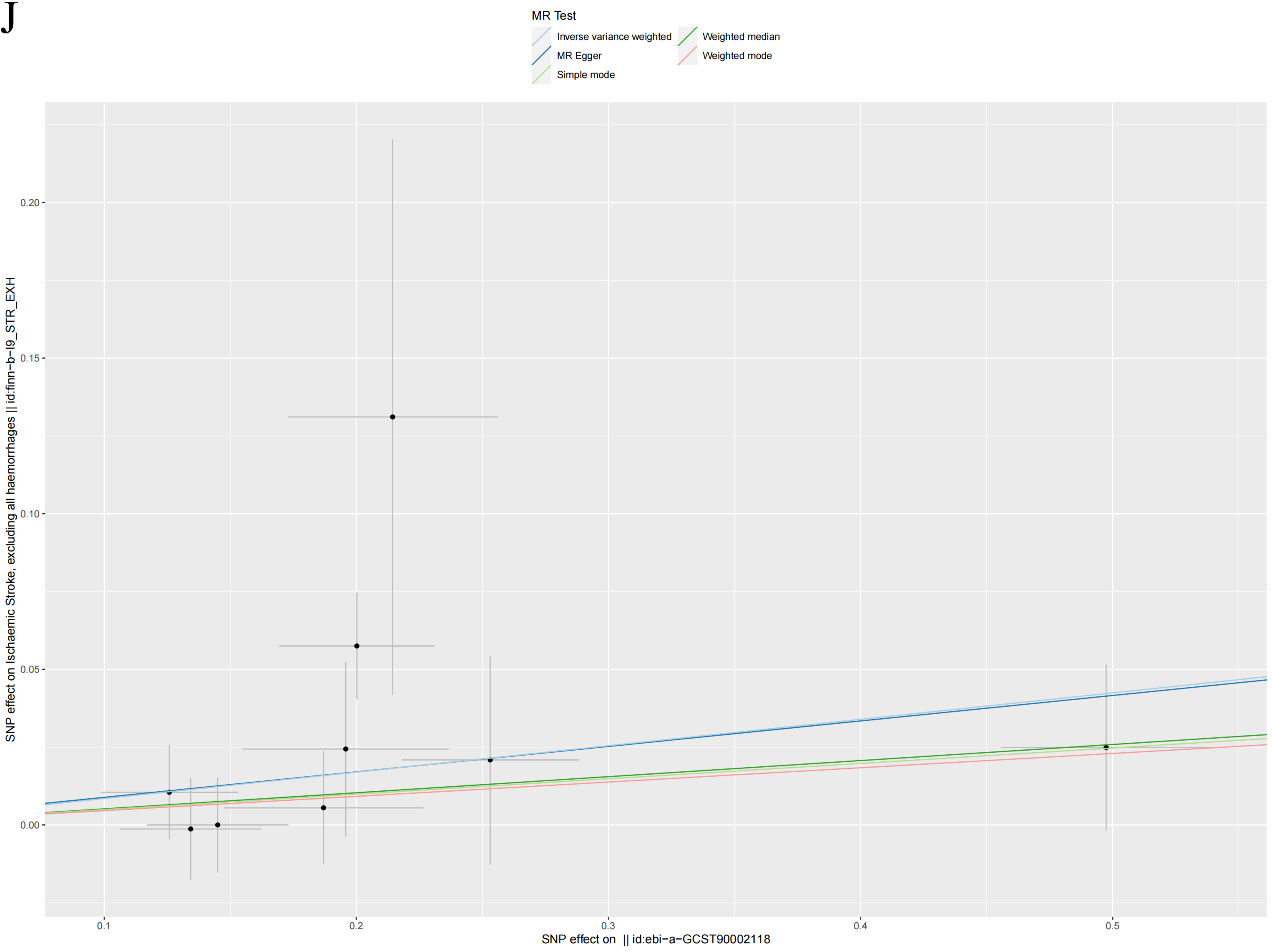

MR Test
Inverse variance weighted
MR Egger
Simple mode
Weighted median
Weighted mode
SNP effect on Ischaemic Stroke, excluding all haemorrhages || id:finn-b-I9_STR_EXH
0.20
0.15
0.10
0.05
0.00
0.1
0.2
0.3
0.4
0.5
SNP effect on || id:ebi-a-GCST90002118

K

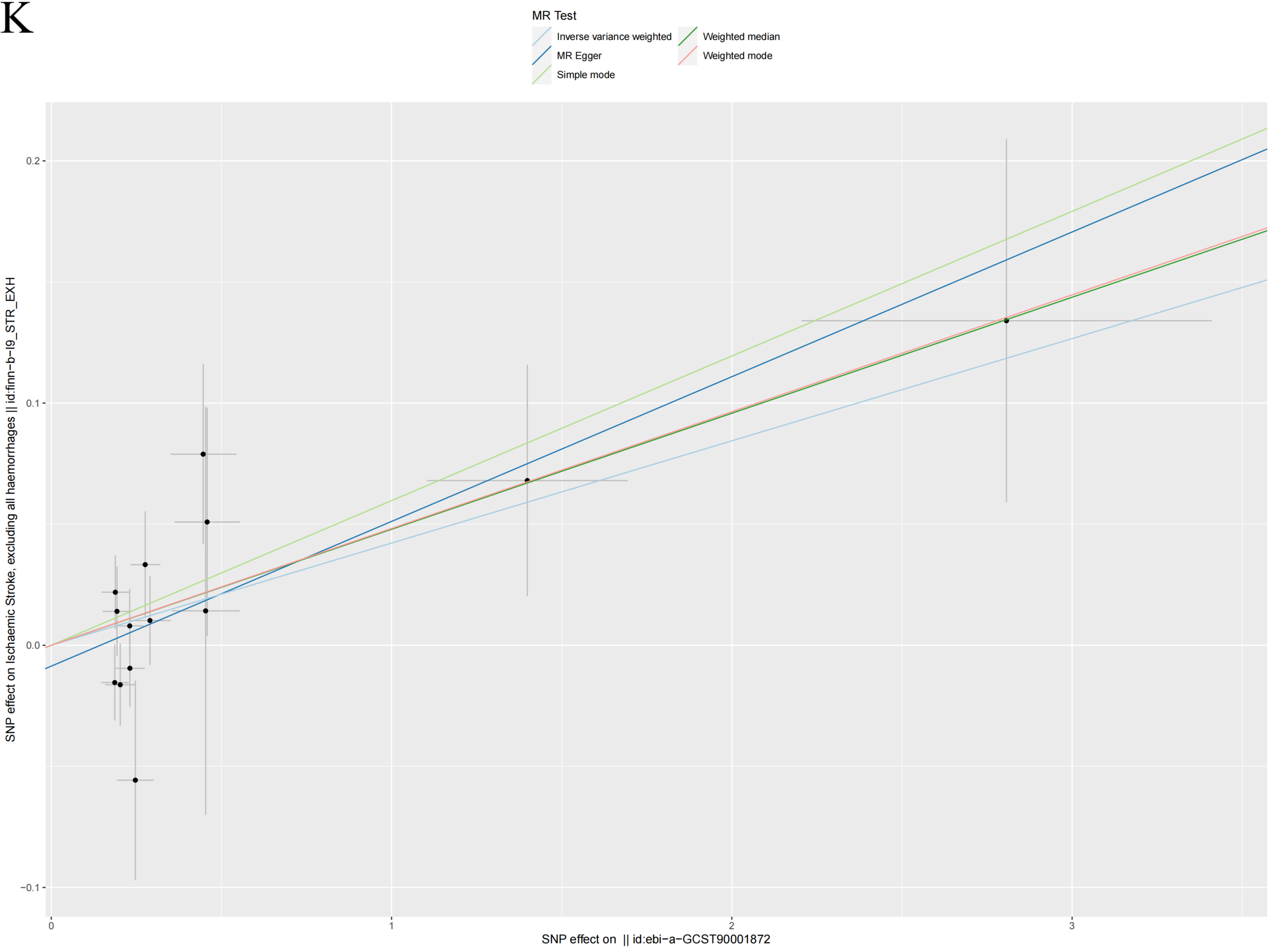
MR Test
Inverse variance weighted
MR Egger
Simple mode
Weighted median
Weighted mode
SNP effect on Ischaemic Stroke, excluding all haemorrhages || id:finn-b-I9_STR_EXH
0.2
0.1
0.0
−0.1
0
1
2
3
SNP effect on || id:ebi−a−GCST90001872

L

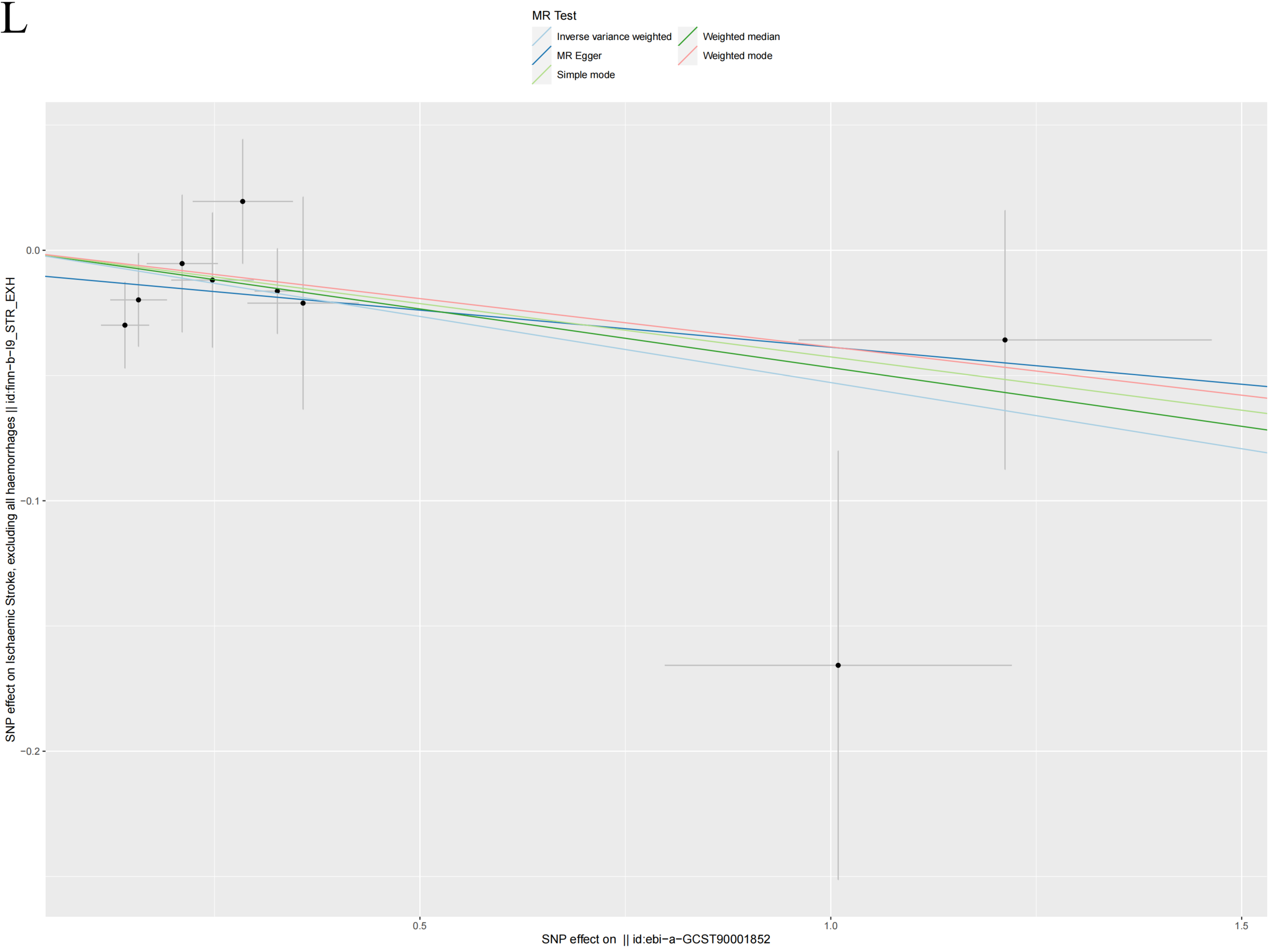

MR Test
Inverse variance weighted
MR Egger
Simple mode
Weighted median
Weighted mode
0.0
−0.1
−0.2
0.5
1.0
1.5
SNP effect on Ischaemic Stroke, excluding all haemorrhages || id:finn−b−I9_STR_EXH
SNP effect on || id:ebi−a−GCST90001852

M

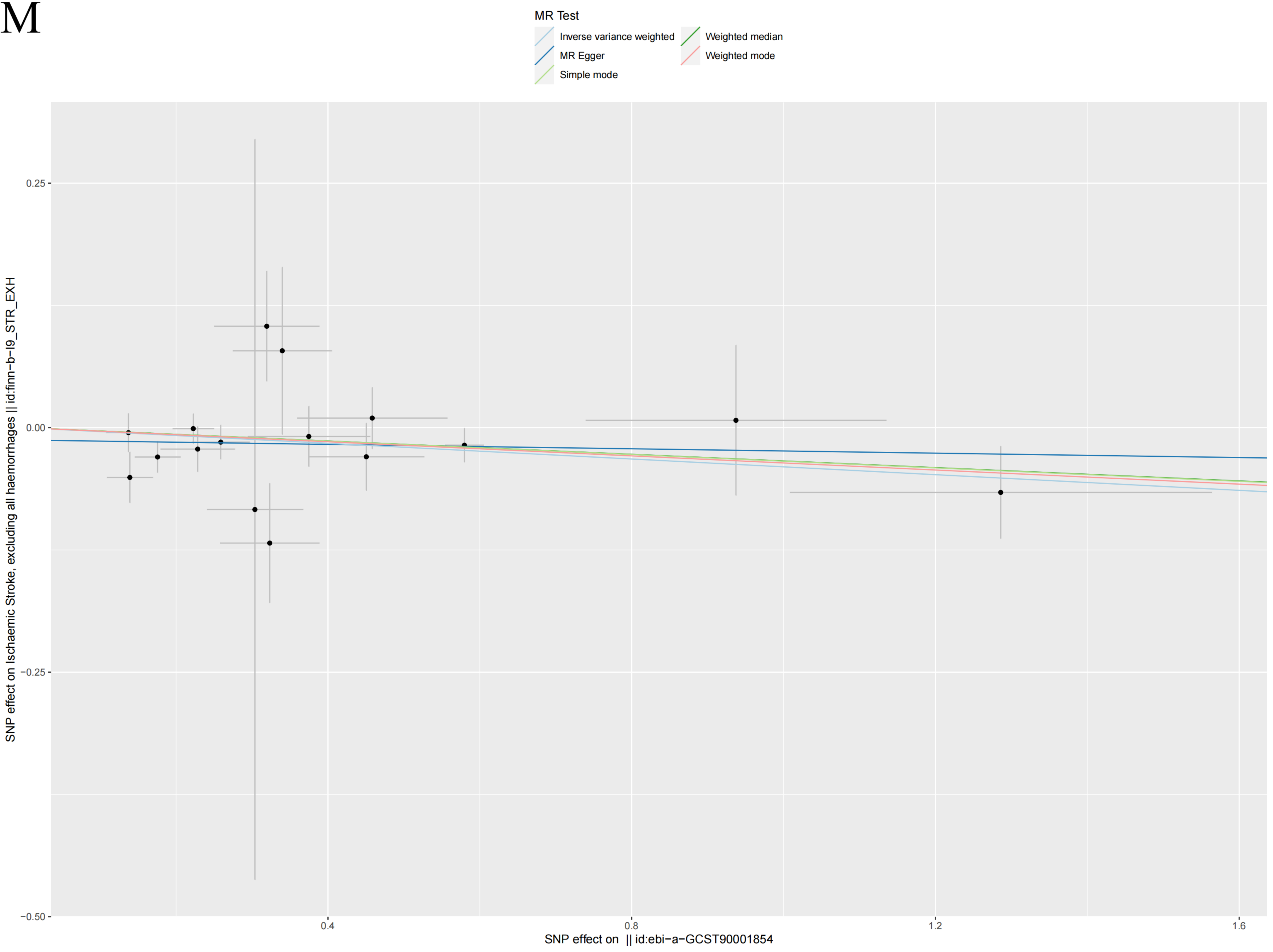

MR Test
Inverse variance weighted
MR Egger
Simple mode
Weighted median
Weighted mode
0.25
0.00
−0.25
−0.50
0.4
0.8
1.2
1.6
SNP effect on Ischaemic Stroke, excluding all haemorrhages || id:finn−b−I9_STR_EXH
SNP effect on || id:ebi−a−GCST90001854

N

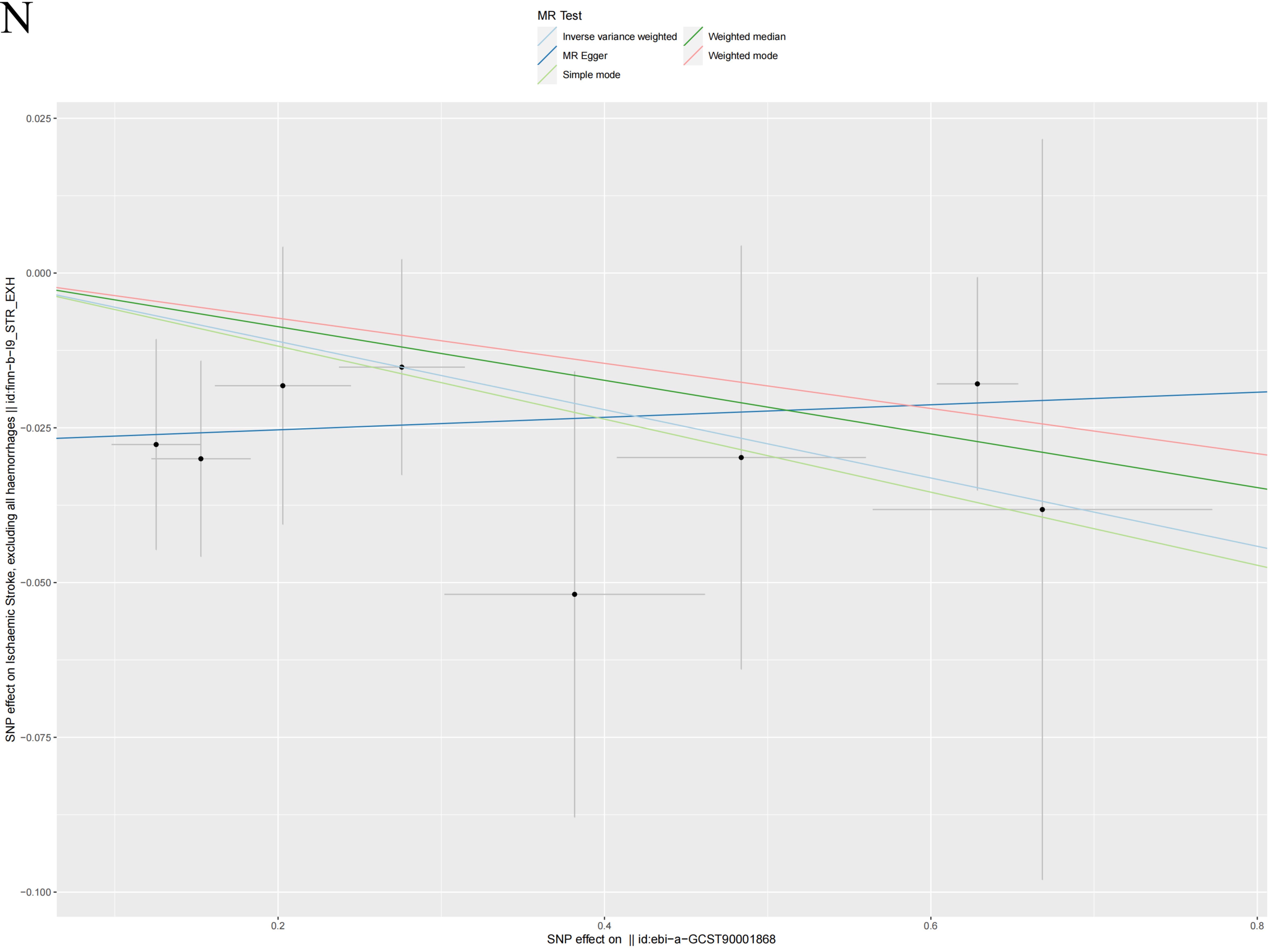
MR Test
Inverse variance weighted
MR Egger
Simple mode
Weighted median
Weighted mode
0.025
0.000
−0.025
−0.050
−0.075
−0.100
SNP effect on Ischaemic Stroke, excluding all haemorrhages || id:finn−b−I9_STR_EXH
0.2
0.4
0.6
0.8
SNP effect on || id:ebi−a−GCST90001868

O

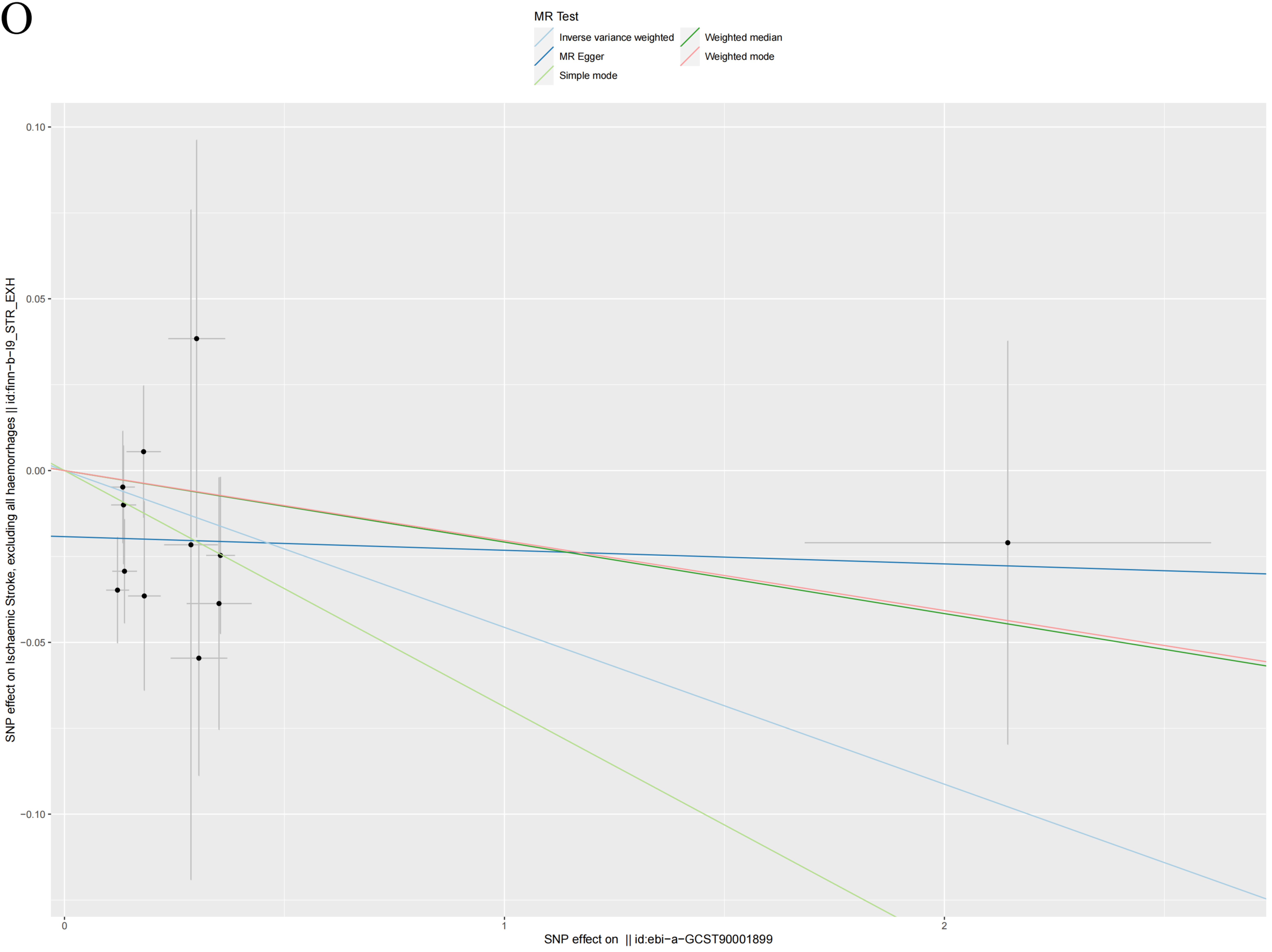

MR Test
Inverse variance weighted
MR Egger
Simple mode
Weighted median
Weighted mode
0.10
0.05
0.00
−0.05
−0.10
SNP effect on Ischaemic Stroke, excluding all haemorrhages || id:finn-b-I9_STR_EXH
0
1
2
SNP effect on || id:ebi-a-GCST90001899

P

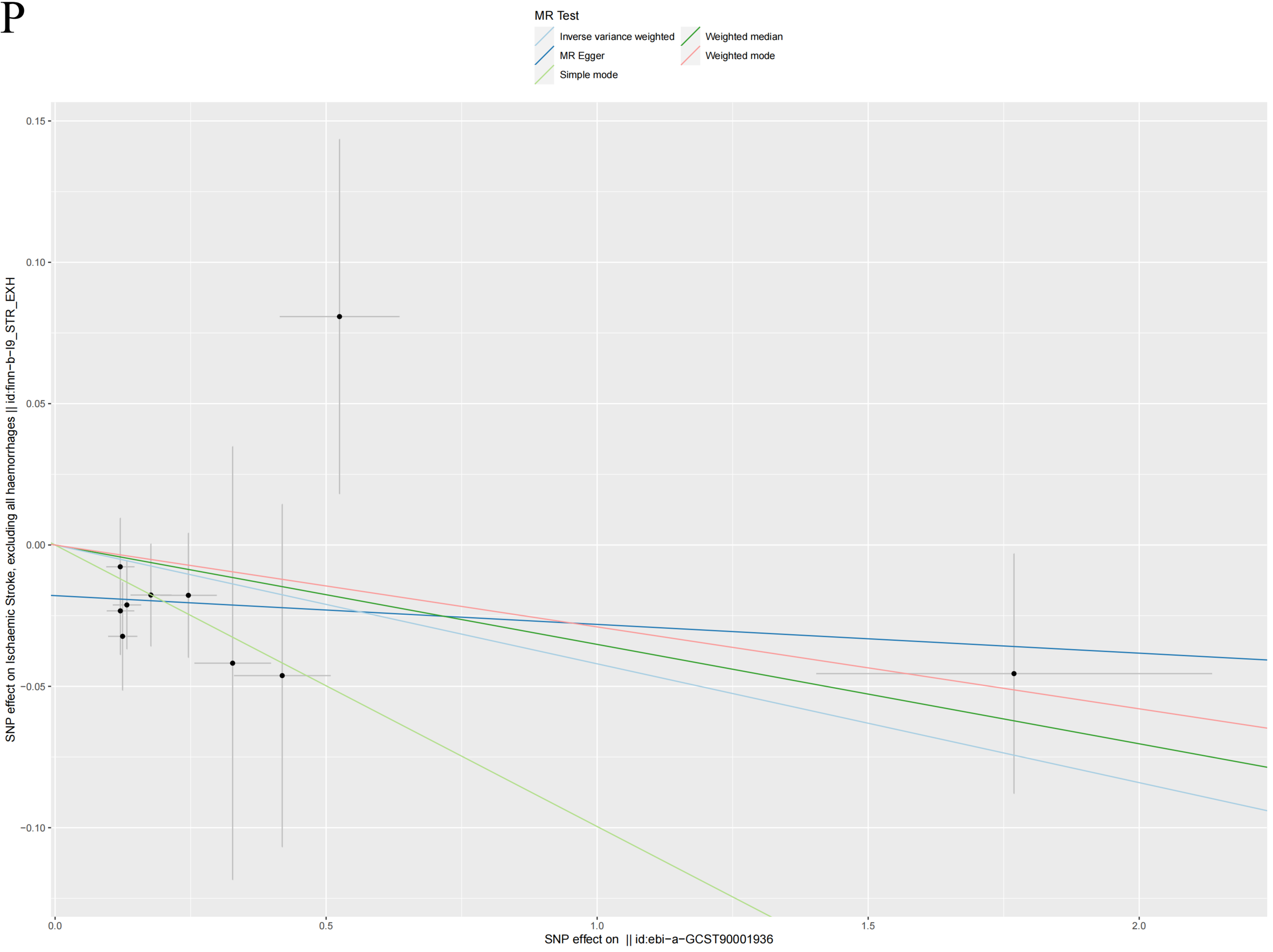
MR Test
Inverse variance weighted
MR Egger
Simple mode
Weighted median
Weighted mode
0.15
0.10
0.05
0.00
−0.05
−0.10
0.0
0.5
1.0
1.5
2.0
SNP effect on Ischaemic Stroke, excluding all haemorrhages || id:finn-b-I9_STR_EXH
SNP effect on || id:ebi-a-GCST90001936

Q

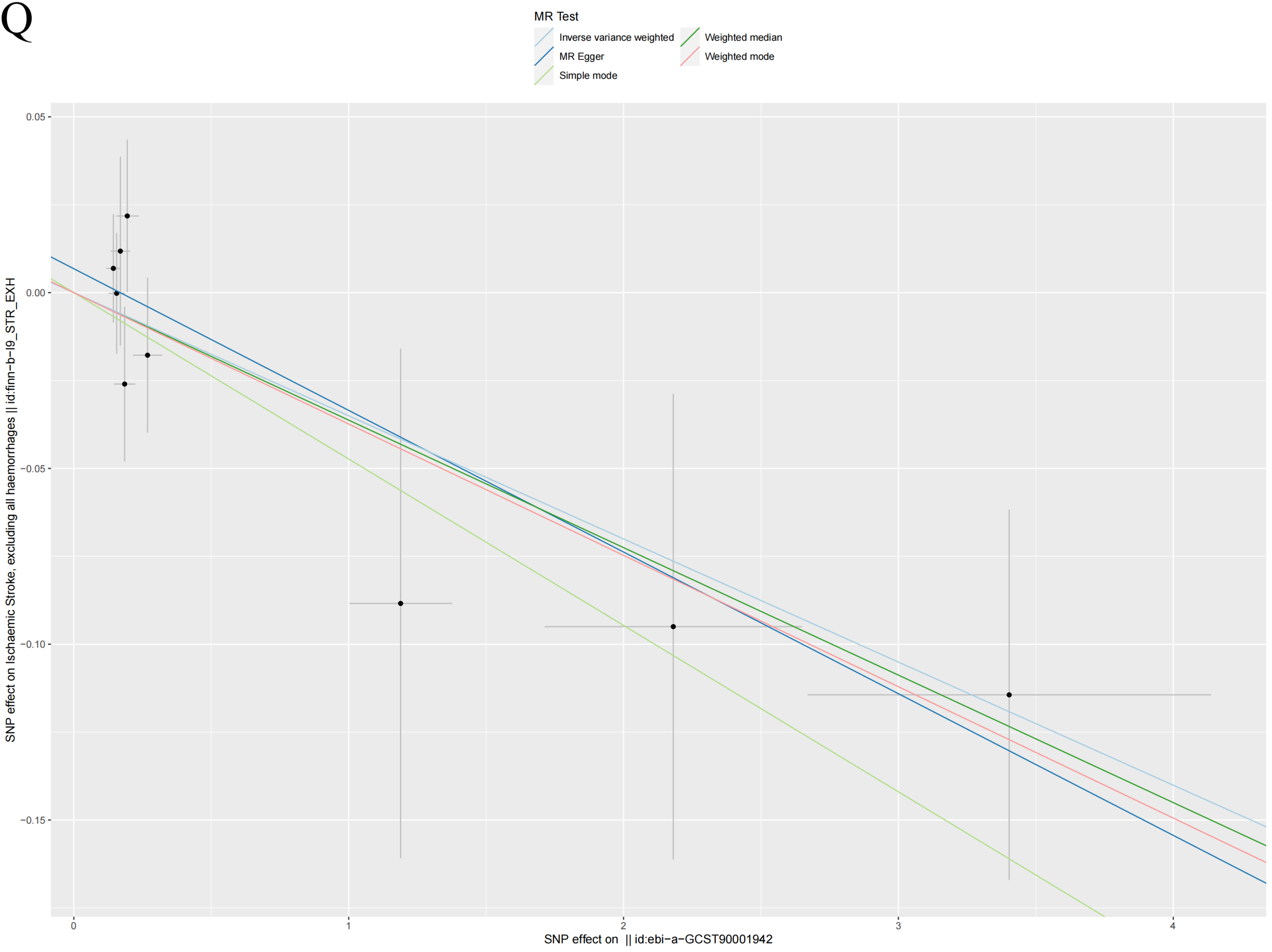
MR Test
Inverse variance weighted
MR Egger
Simple mode
Weighted median
Weighted mode
0.05
0.00
−0.05
−0.10
−0.15
0
1
2
3
4
SNP effect on Ischaemic Stroke, excluding all haemorrhages || id:finn-b-I9_STR_EXH
SNP effect on || id:ebi-a-GCST90001942

R

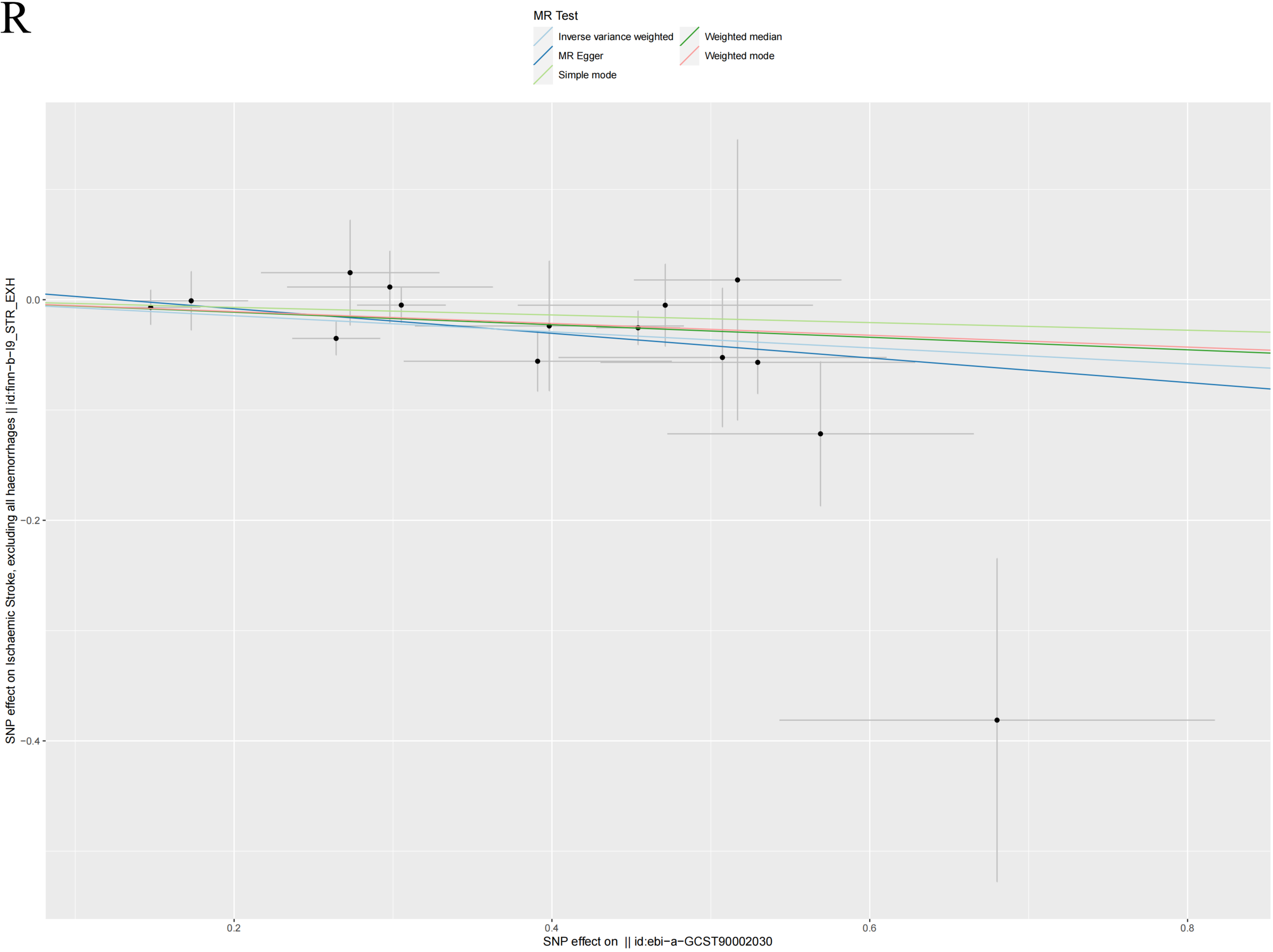
MR Test
Inverse variance weighted
MR Egger
Simple mode
Weighted median
Weighted mode
SNP effect on Ischaemic Stroke, excluding all haemorrhages || id:finn-b-I9_STR_EXH
0.0
−0.2
−0.4
0.2
0.4
0.6
0.8
SNP effect on || id:ebi-a-GCST90002030

S

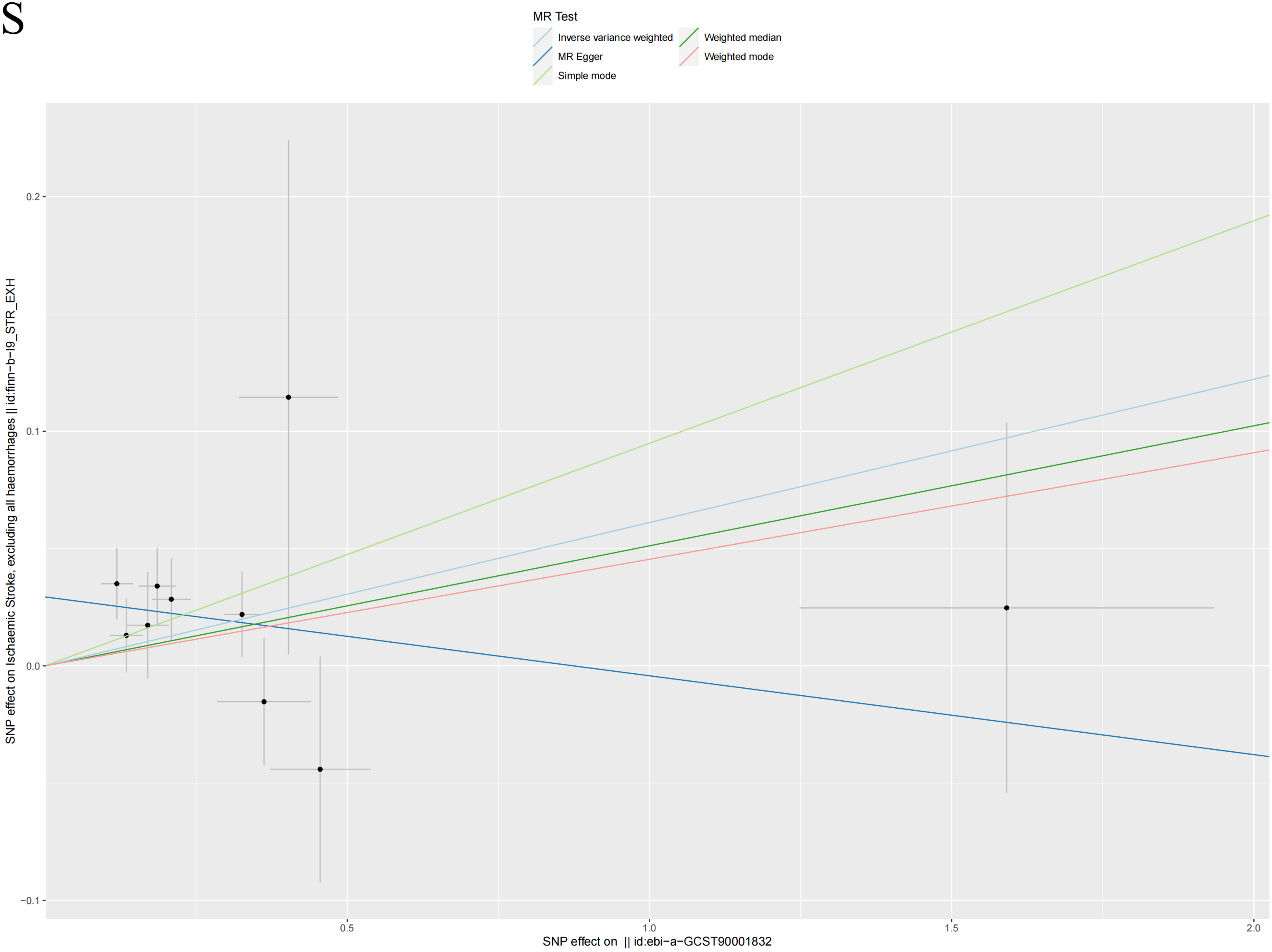

MR Test
Inverse variance weighted
MR Egger
Simple mode
Weighted median
Weighted mode
0.2
0.1
0.0
−0.1
0.5
1.0
1.5
2.0
SNP effect on Ischaemic Stroke, excluding all haemorrhages || id:finn−b−I9_STR_EXH
SNP effect on || id:ebi−a−GCST90001832

T

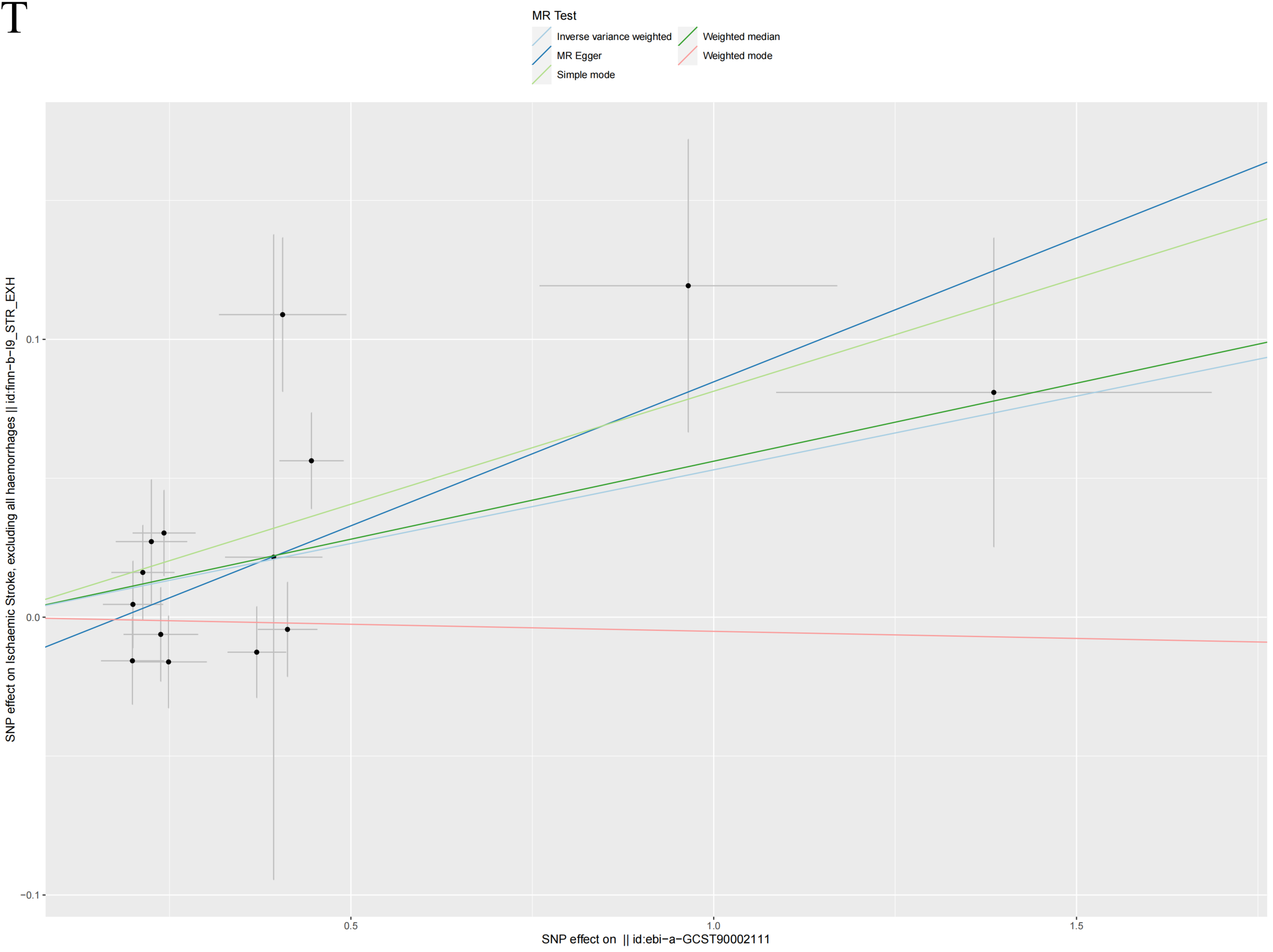

MR Test
Inverse variance weighted
MR Egger
Simple mode
Weighted median
Weighted mode
SNP effect on Ischaemic Stroke, excluding all haemorrhages || id:finn-b-I9_STR_EXH
0.1
0.0
−0.1
0.5
1.0
1.5
SNP effect on || id:ebi−a−GCST90002111

U

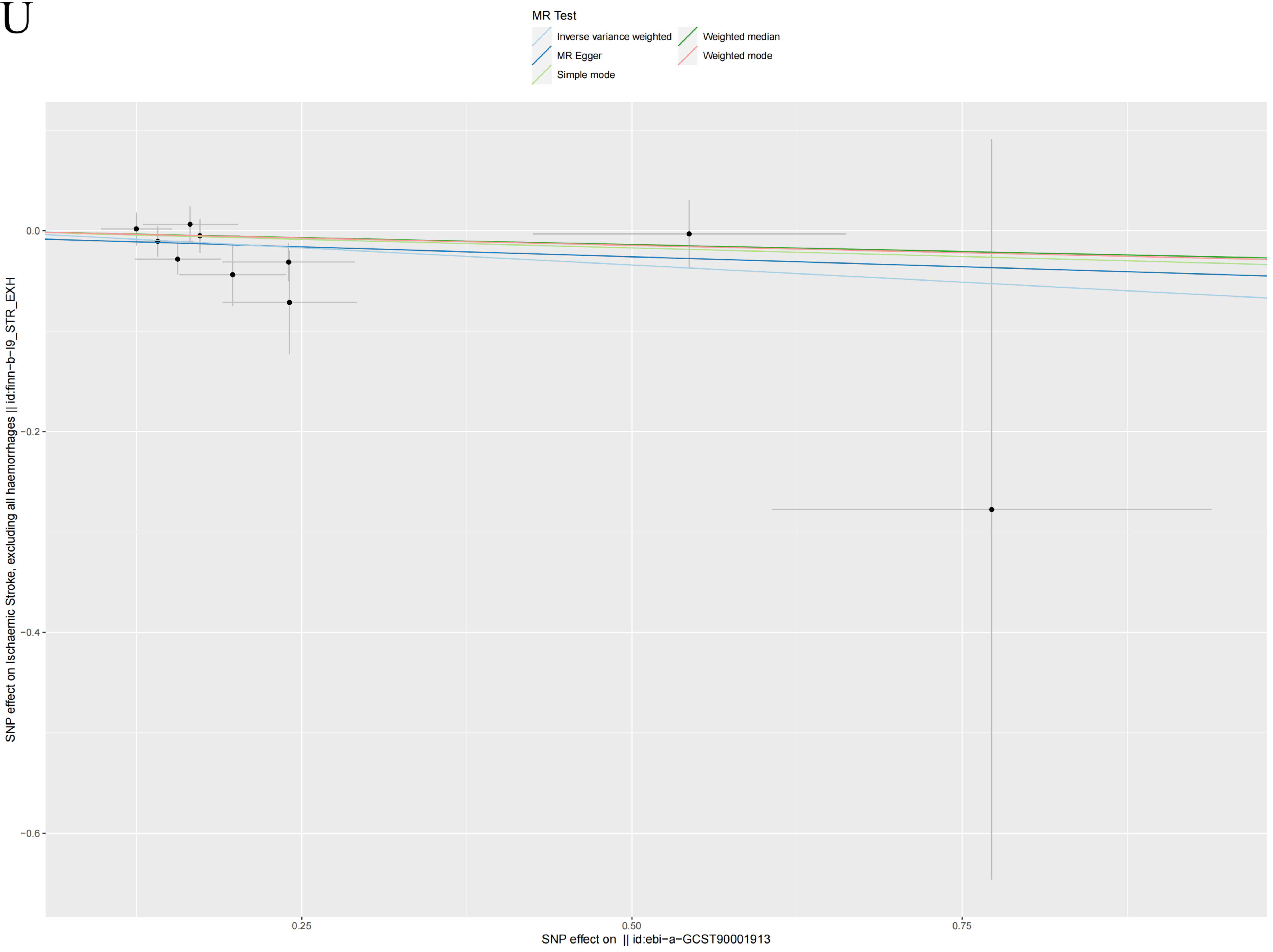
MR Test
Inverse variance weighted
MR Egger
Simple mode
Weighted median
Weighted mode
0.0
−0.2
−0.4
−0.6
0.25
0.50
0.75
SNP effect on Ischaemic Stroke, excluding all haemorrhages || id:finn−b−I9_STR_EXH
SNP effect on || id:ebi−a−GCST90001913

Supplementary Figure 1. Sensitivity analysis of forward Mendelian randomization (Scatter plot).

A. Scatter plot between BAFF-R on IgD+ CD38- unswitched memory B cell and IS; B. Scatter plot between CD27 on CD20- CD38- B cell and IS; C. Scatter plot between CD38 on IgD+ CD24- B cell and IS; D. Scatter plot between CD19 on CD24+ CD27+ B cell and IS; E. Scatter plot between CD19 on memory B cell and IS; F. Scatter plot between CD25 on naive-mature B cell and IS; G. Scatter plot between CD25 on CD45RA+ CD4 not regulatory T cell and IS; H. Scatter plot between CD39 on CD39+ CD8+ T cell and IS; I. Scatter plot between HLA DR on HLA DR+ CD4+ T cell and IS; J. Scatter plot between CD8 on CD28+ CD45RA- CD8+ T cell and IS; K. Scatter plot between HVEM on naive CD8+ T cell and IS; L. Scatter plot between CD3 on CD39+ resting CD4 regulatory T cell and IS; M. Scatter plot between CD3 on CD39+ activated CD4 regulatory T cell and IS; N. Scatter plot between CD3 on CD4 regulatory T cell and IS; O. Scatter plot between CD28 on CD4 regulatory T cell and IS; P. Scatter plot between CD25 on CD4 regulatory T cell and IS; Q. Scatter plot between CD25 on CD39+ secreting CD4 regulatory T cell and IS; R. Scatter plot between CD39 on CD39+ activated CD4 regulatory T cell and IS; S. Scatter plot between CD62L on CD62L+ plasmacytoid Dendritic Cell and IS; T. Scatter plot between HLA DR on CD33dim HLA DR+ CD11b- and IS; U. Scatter plot between CD45 on granulocyte and IS.

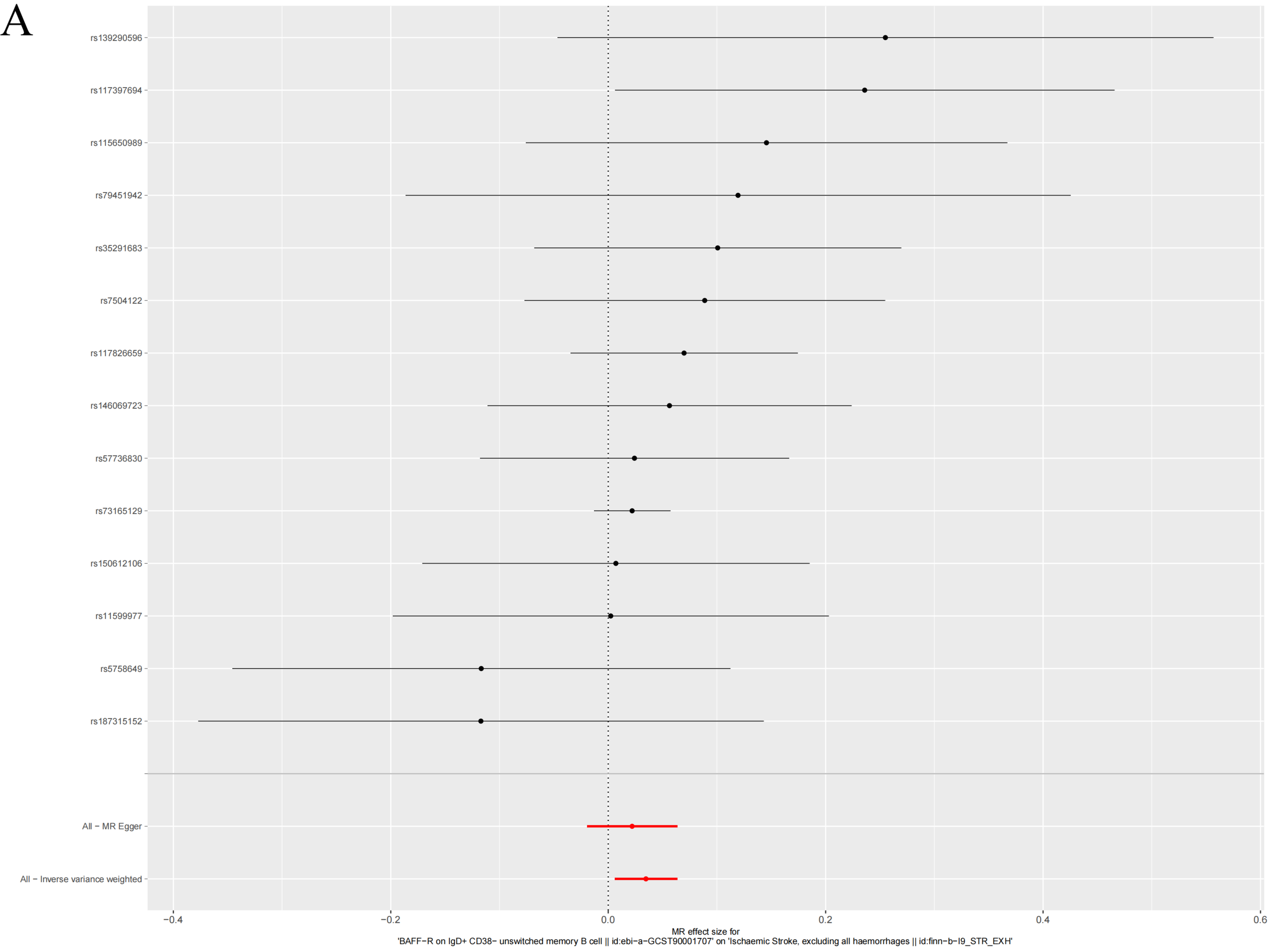
A
rs139290596
rs117397694
rs115650989
rs79451942
rs35291683
rs7504122
rs117826659
rs146069723
rs57736830
rs73165129
rs150612106
rs11599977
rs5758649
rs187315152
All − MR Egger
All − Inverse variance weighted
−0.4
−0.2
0.0
0.2
0.4
0.6
MR effect size for
'BAFF−R on IgD+ CD38− unswitched memory B cell || id:ebi−a−GCST90001707' on 'Ischaemic Stroke, excluding all haemorrhages || id:finn−b−I9_STR_EXH'

B

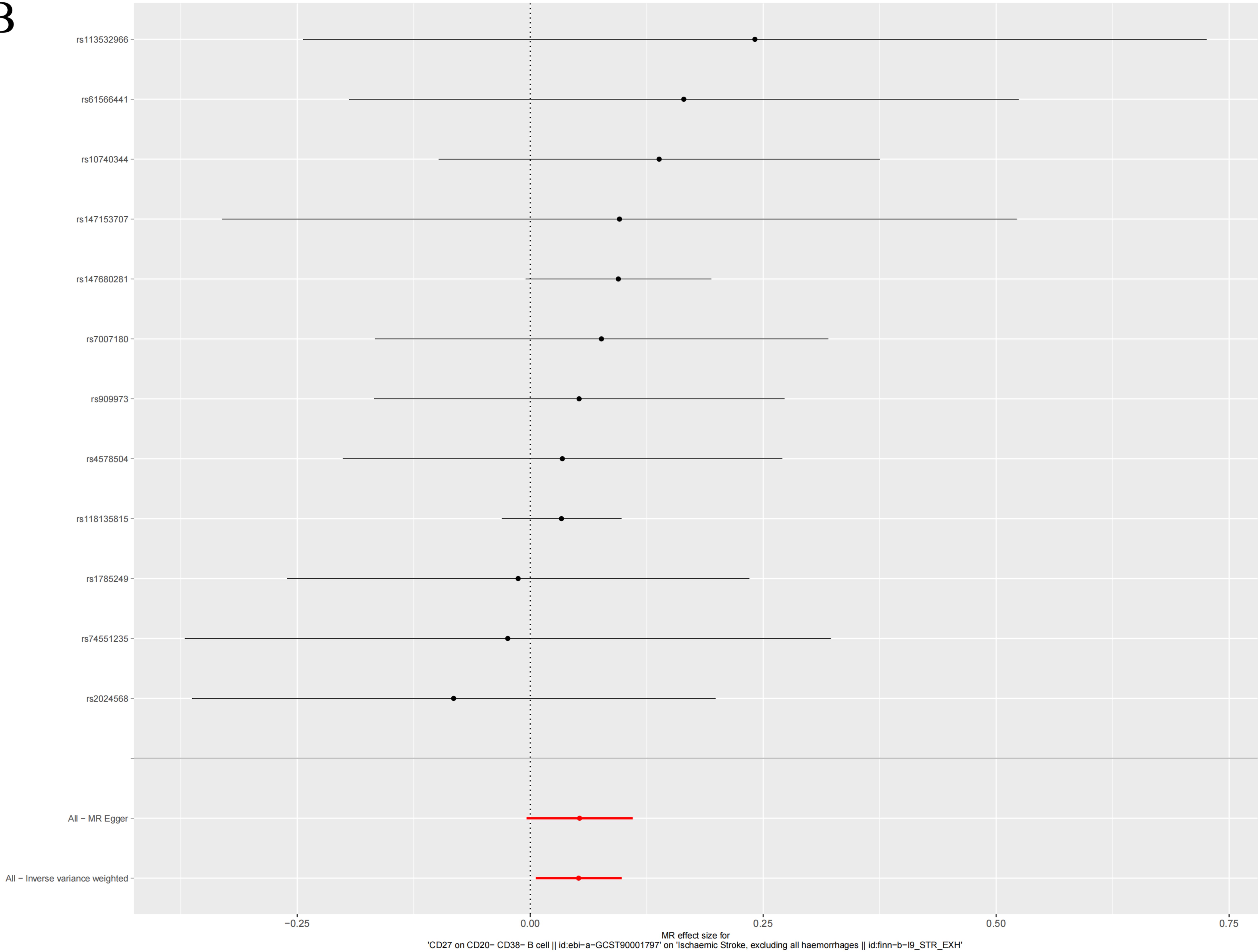

rs113532966
rs61566441
rs10740344
rs147153707
rs147680281
rs7007180
rs909973
rs4578504
rs118135815
rs1785249
rs74551235
rs2024568
All − MR Egger
All − Inverse variance weighted
−0.25
0.00
0.25
0.50
0.75
MR effect size for
'CD27 on CD20− CD38− B cell || id:ebi−a−GCST90001797' on 'Ischaemic Stroke, excluding all haemorrhages || id:finn−b−I9_STR_EXH'

C

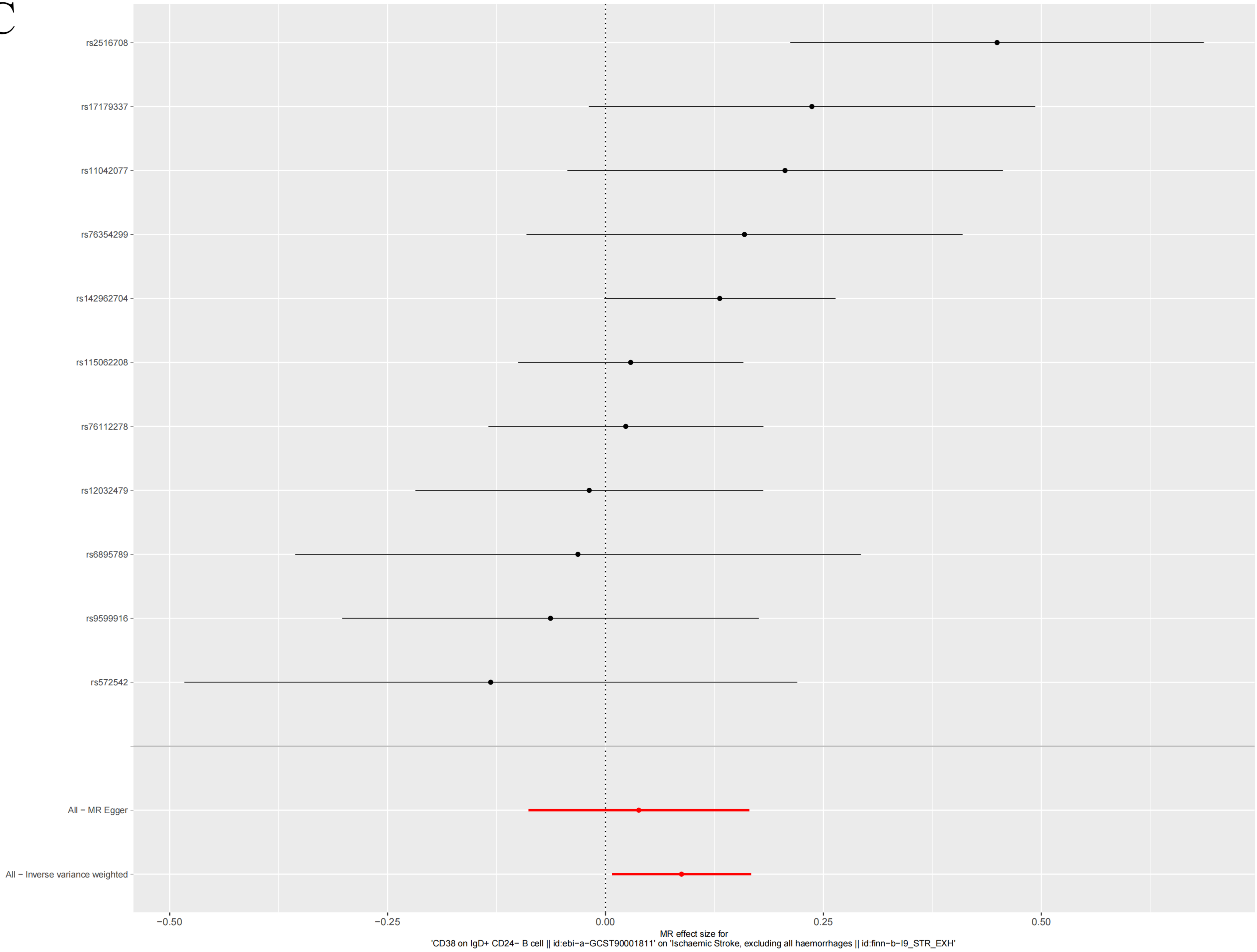
rs2516708
rs17179337
rs11042077
rs76354299
rs142962704
rs115062208
rs76112278
rs12032479
rs6895789
rs9599916
rs572542
All − MR Egger
All − Inverse variance weighted
−0.50
−0.25
0.00
0.25
0.50
MR effect size for
'CD38 on IgD+ CD24− B cell || id:ebi−a−GCST90001811' on 'Ischaemic Stroke, excluding all haemorrhages || id:finn−b−I9_STR_EXH'

D

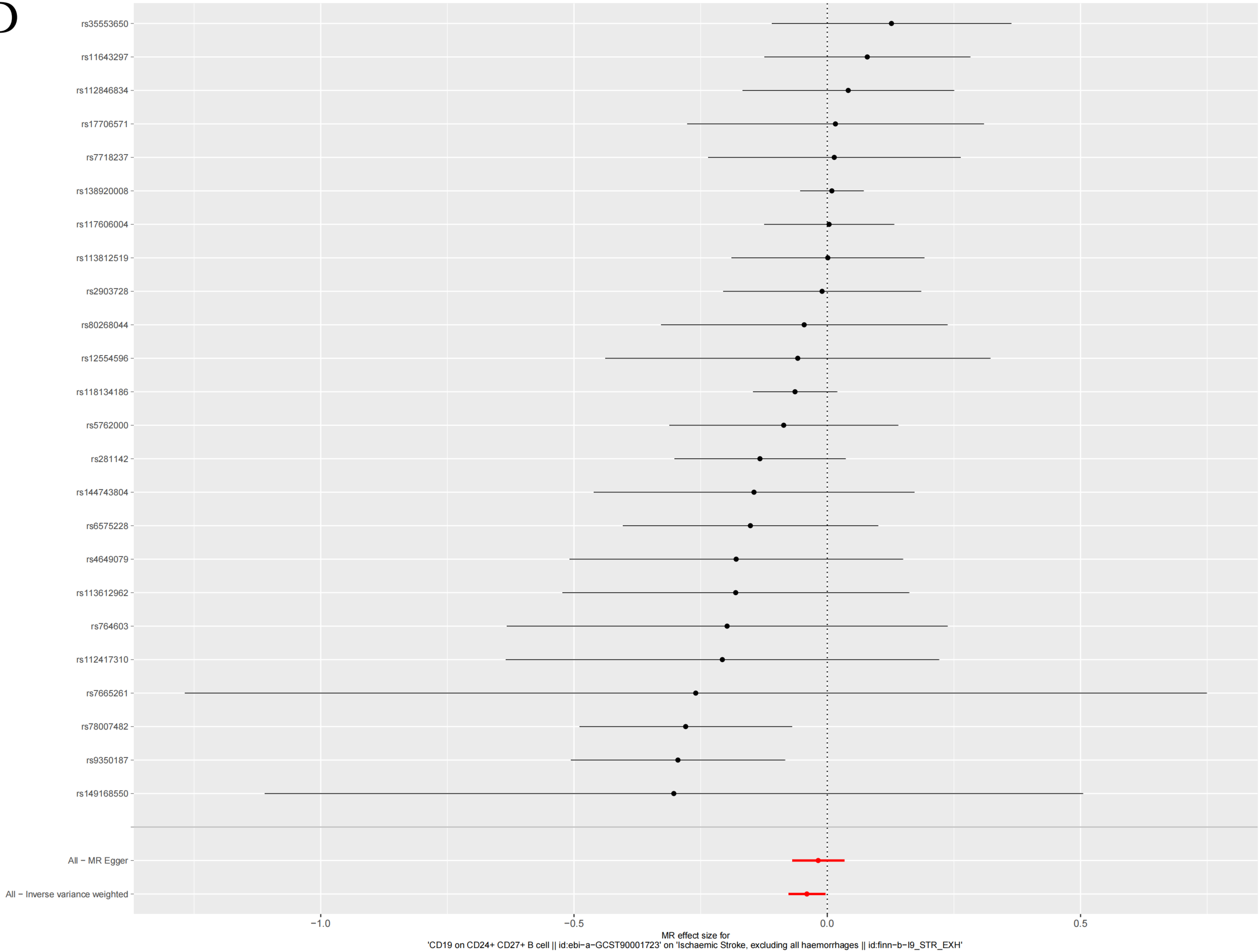
rs35553650
rs11643297
rs112846834
rs17706571
rs7718237
rs138920008
rs117606004
rs113812519
rs2903728
rs80268044
rs12554596
rs118134186
rs5762000
rs281142
rs144743804
rs6575228
rs4649079
rs113612962
rs764603
rs112417310
rs7665261
rs78007482
rs9350187
rs149168550
All − MR Egger
All − Inverse variance weighted
−1.0
−0.5
0.0
0.5
MR effect size for
'CD19 on CD24+ CD27+ B cell || id:ebi−a−GCST90001723' on 'Ischaemic Stroke, excluding all haemorrhages || id:finn−b−I9_STR_EXH'

E

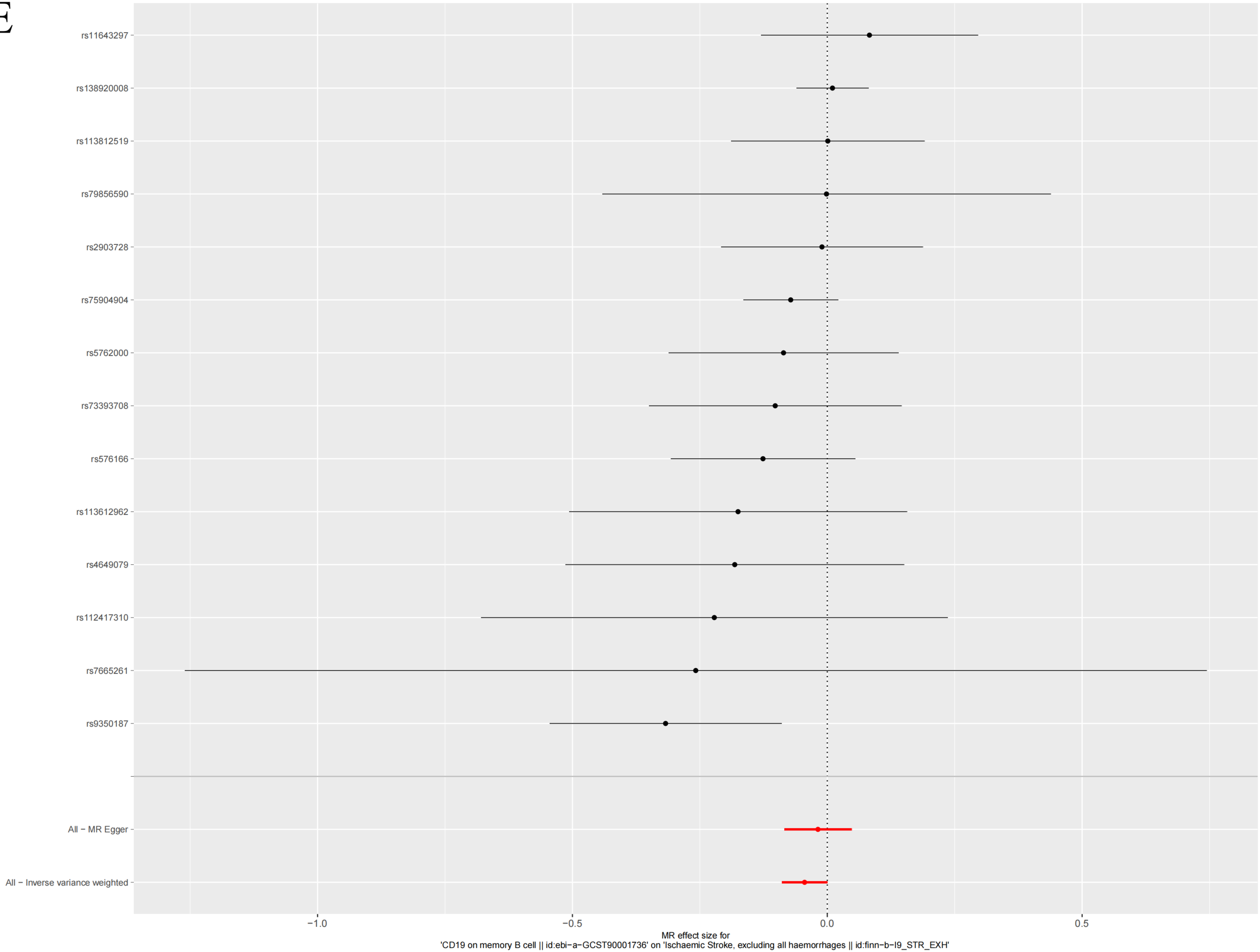
rs11643297
rs138920008
rs113812519
rs79856590
rs2903728
rs75904904
rs5762000
rs73393708
rs576166
rs113612962
rs4649079
rs112417310
rs7665261
rs9350187
All − MR Egger
All − Inverse variance weighted
−1.0
−0.5
0.0
0.5
MR effect size for
'CD19 on memory B cell || id:ebi−a−GCST90001736' on 'Ischaemic Stroke, excluding all haemorrhages || id:finn−b−I9_STR_EXH'

F

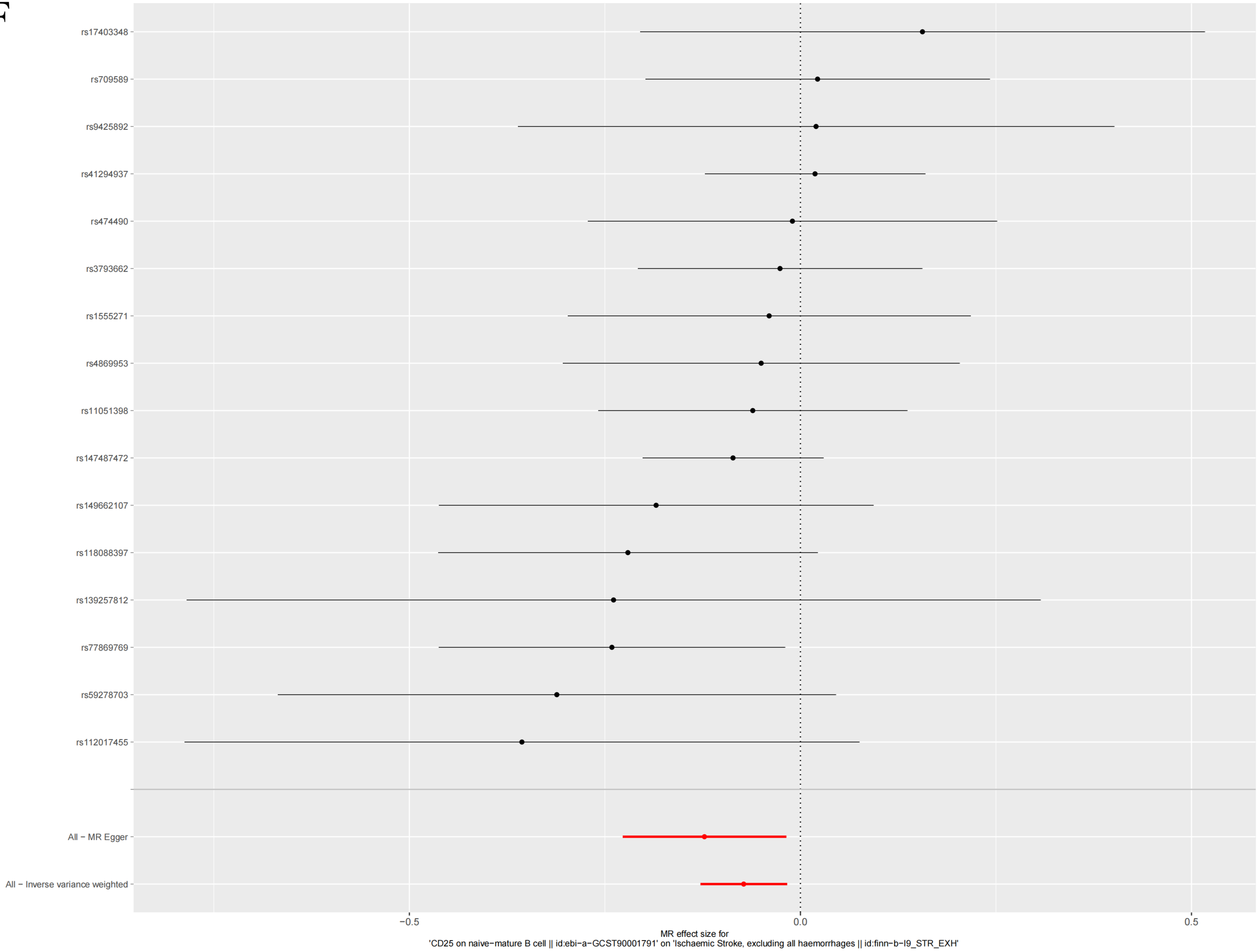

rs17403348
rs709589
rs9425892
rs41294937
rs474490
rs3793662
rs1555271
rs4869953
rs11051398
rs147487472
rs149662107
rs118088397
rs139257812
rs77869769
rs59278703
rs112017455
All − MR Egger
All − Inverse variance weighted
−0.5
0.0
0.5
MR effect size for
'CD25 on naive−mature B cell || id:ebi−a−GCST90001791' on 'Ischaemic Stroke, excluding all haemorrhages || id:finn−b−I9_STR_EXH'

G

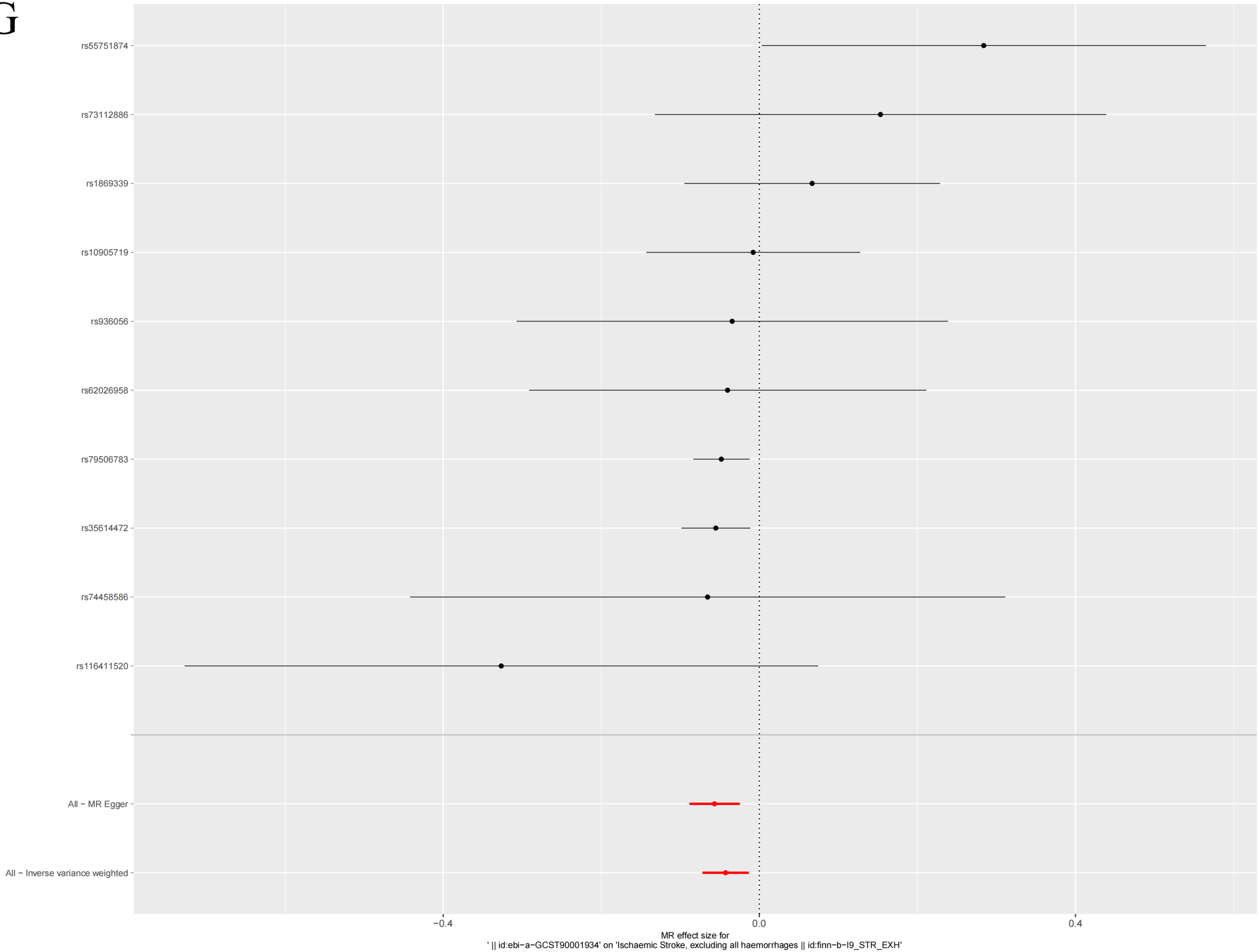
rs55751874
rs73112886
rs1869339
rs10905719
rs936056
rs62026958
rs79506783
rs35614472
rs74458586
rs116411520
All − MR Egger
All − Inverse variance weighted
−0.4
0.0
0.4
MR effect size for
' || id:ebi−a−GCST90001934' on 'Ischaemic Stroke, excluding all haemorrhages || id:finn−b−I9_STR_EXH'

H

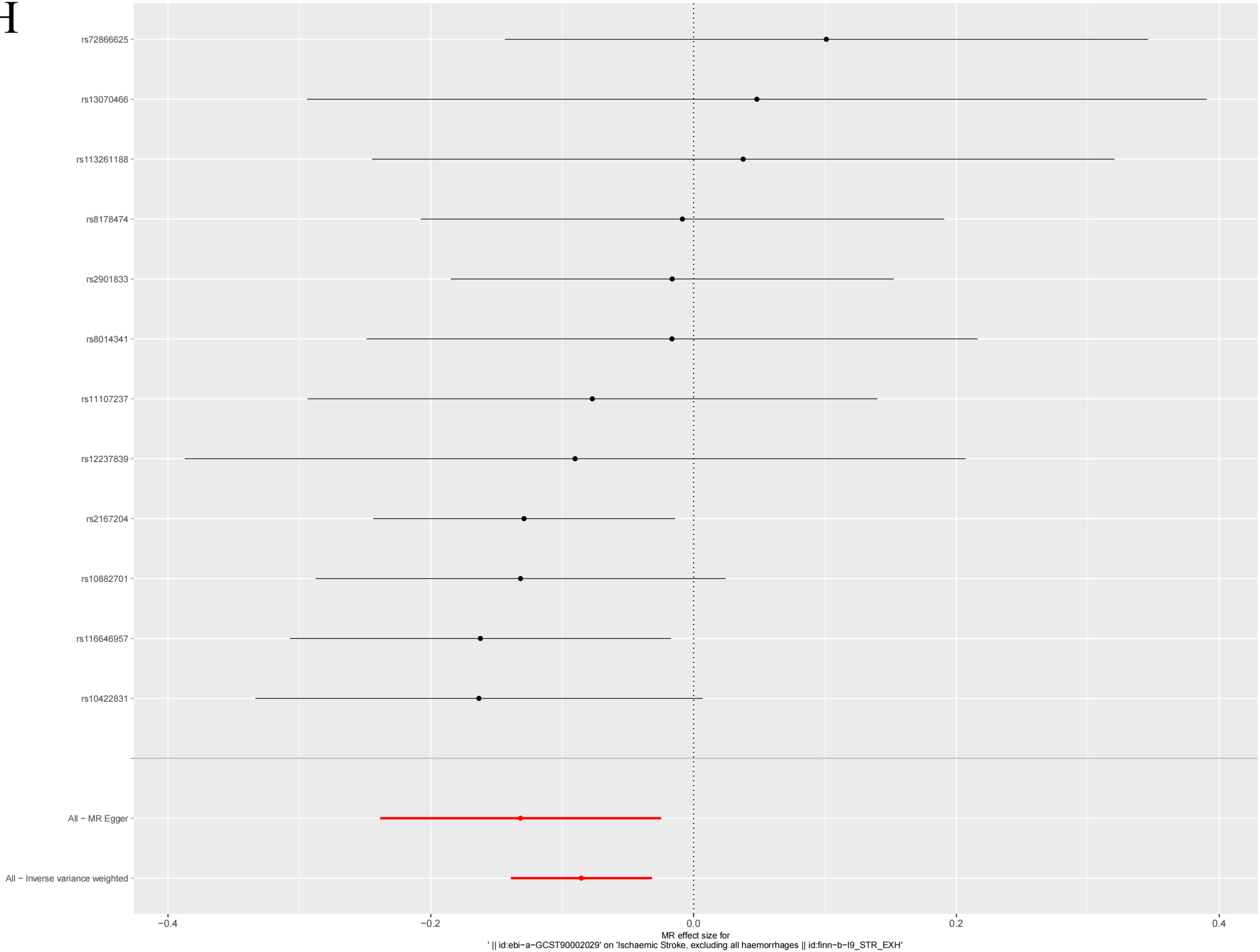
rs72866625
rs13070466
rs113261188
rs8178474
rs2901833
rs8014341
rs11107237
rs12237839
rs2167204
rs10882701
rs116646957
rs10422831
All − MR Egger
All − Inverse variance weighted
−0.4
−0.2
0.0
0.2
0.4
MR effect size for
' || id:ebi−a−GCST90002029' on 'Ischaemic Stroke, excluding all haemorrhages || id:finn−b−I9_STR_EXH'

I

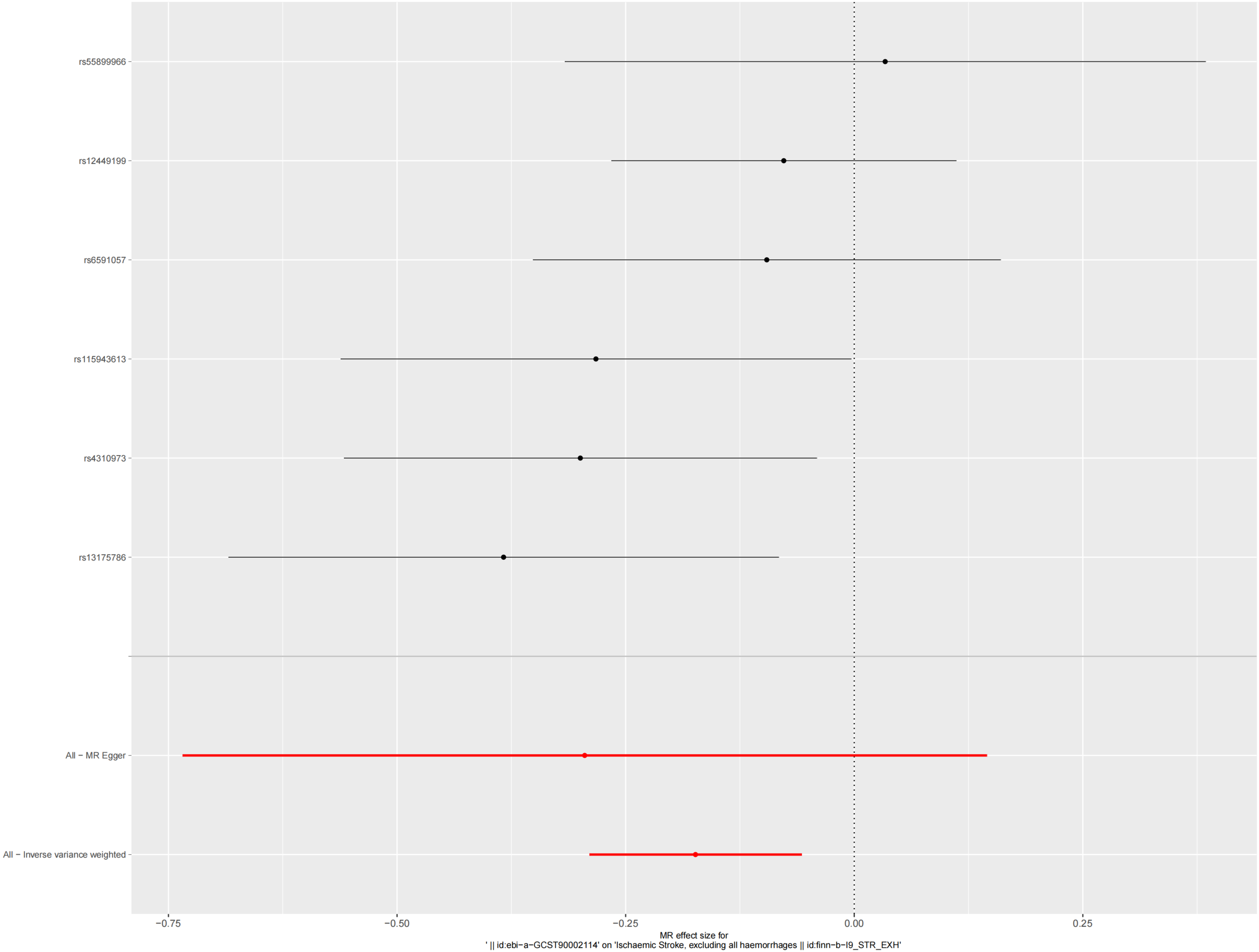
rs55899966
rs12449199
rs6591057
rs115943613
rs4310973
rs13175786
All − MR Egger
All − Inverse variance weighted
−0.75
−0.50
−0.25
0.00
0.25
MR effect size for
' || id:ebi−a−GCST90002114' on 'Ischaemic Stroke, excluding all haemorrhages || id:finn−b−I9_STR_EXH'

J

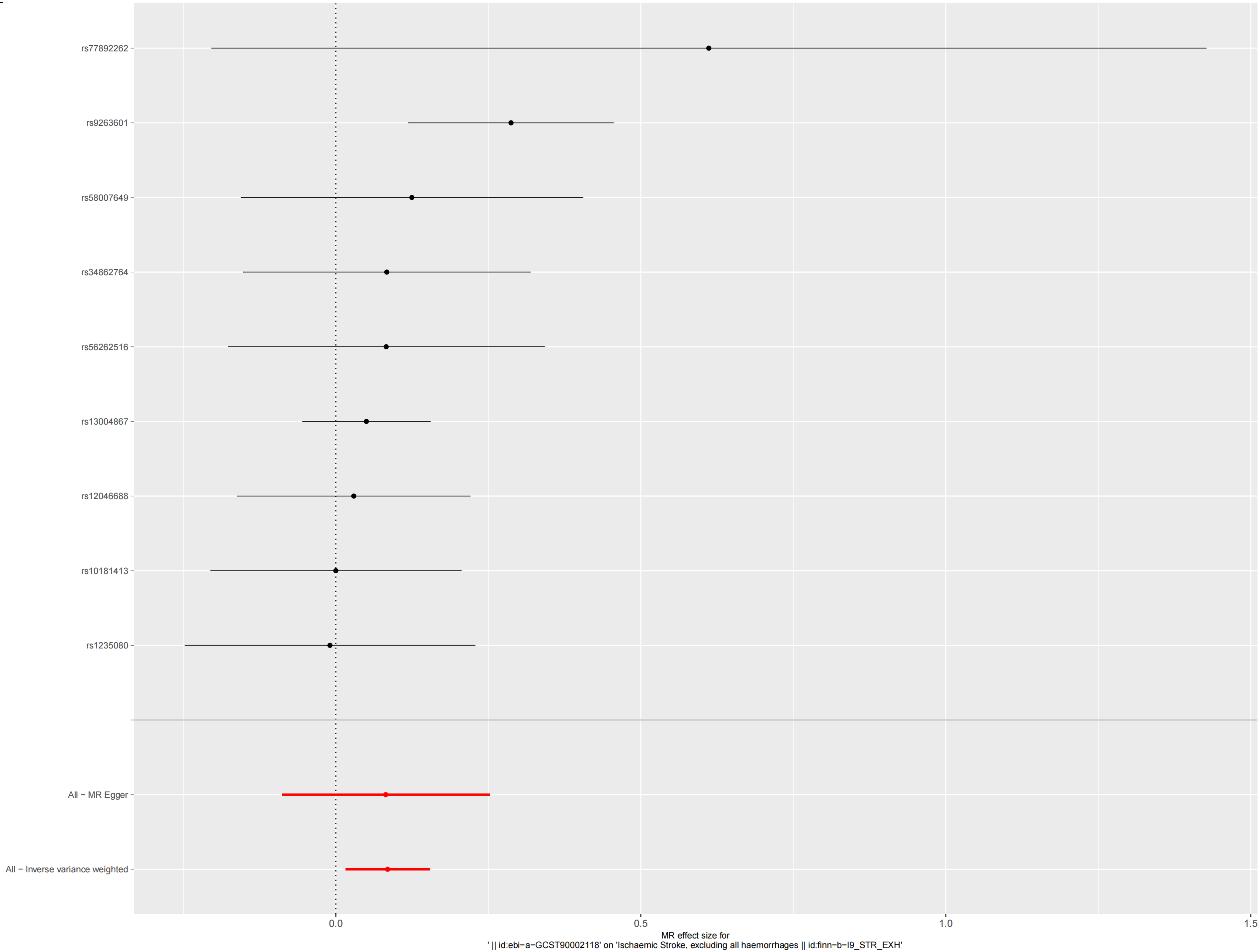
rs77892262
rs9263601
rs58007649
rs34862764
rs56262516
rs13004867
rs12046688
rs10181413
rs1235080
All − MR Egger
All − Inverse variance weighted
0.0
0.5
1.0
1.5
MR effect size for
' || id:ebi−a−GCST90002118' on 'Ischaemic Stroke, excluding all haemorrhages || id:finn−b−I9_STR_EXH'

K

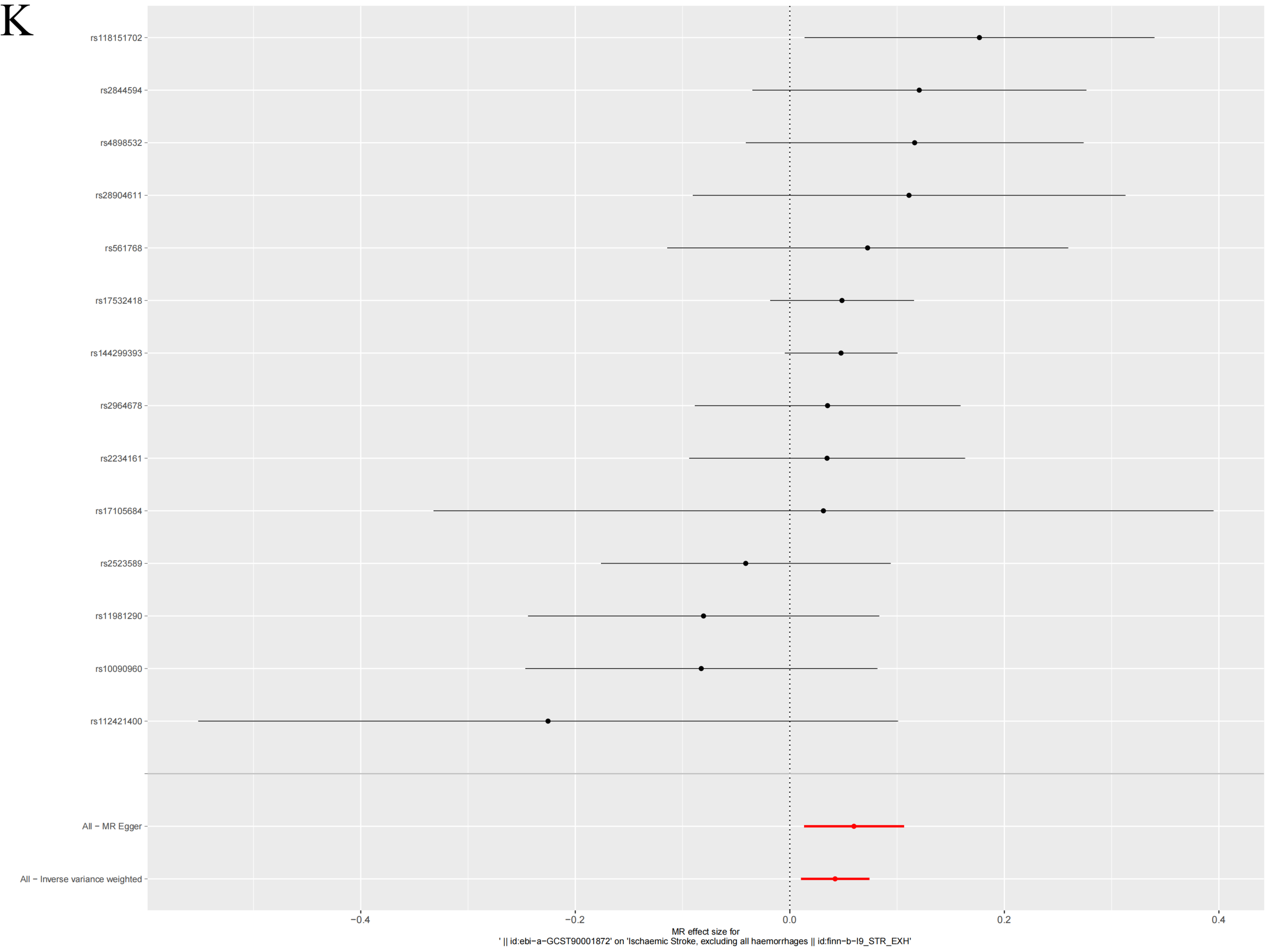
rs118151702
rs2844594
rs4898532
rs28904611
rs561768
rs17532418
rs144299393
rs2964678
rs2234161
rs17105684
rs2523589
rs11981290
rs10090960
rs112421400
All − MR Egger
All − Inverse variance weighted
−0.4
−0.2
0.0
0.2
0.4
MR effect size for
' || id:ebi−a−GCST90001872' on 'Ischaemic Stroke, excluding all haemorrhages || id:finn−b−I9_STR_EXH'

L

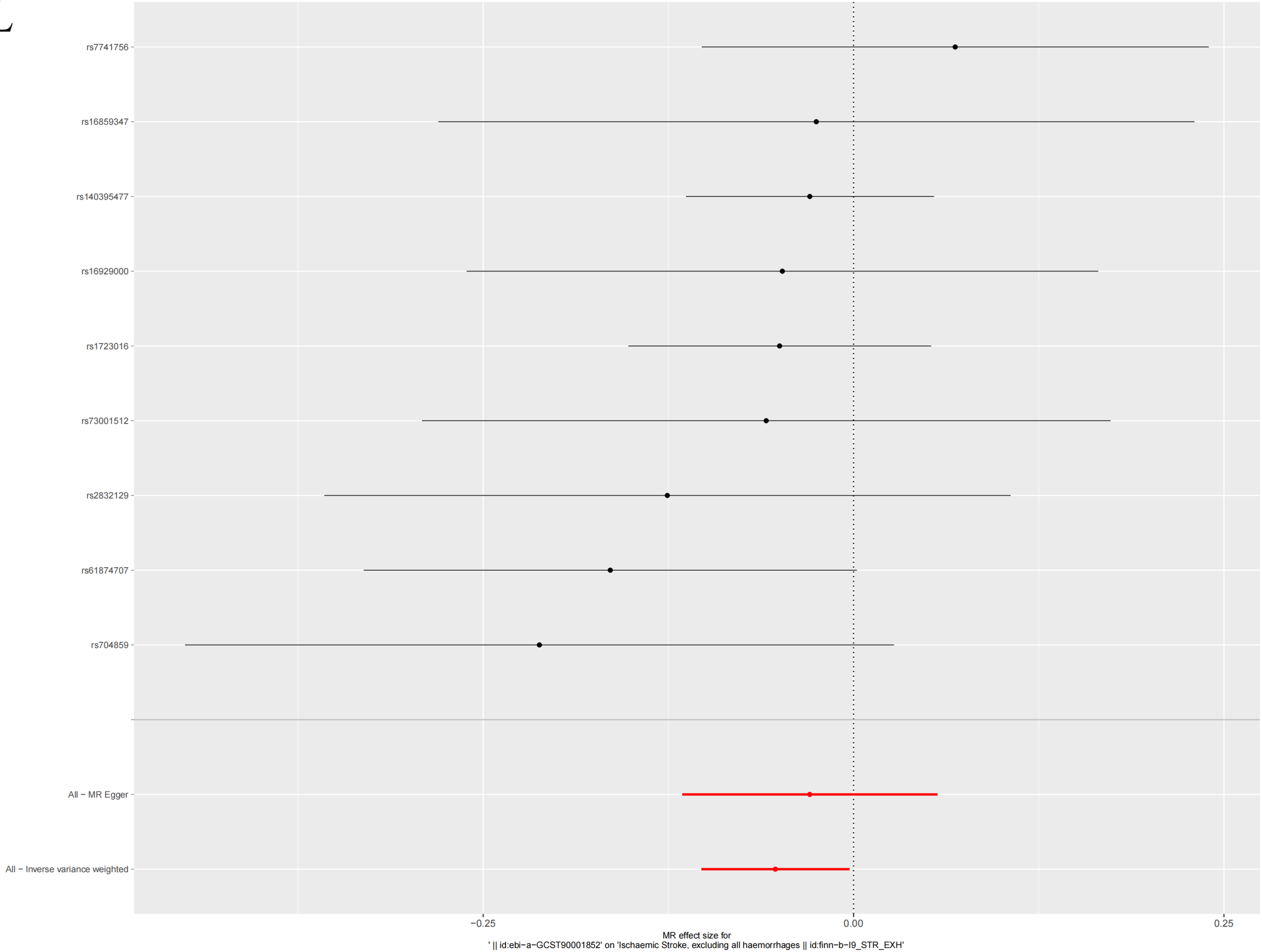

rs7741756
rs16859347
rs140395477
rs16929000
rs1723016
rs73001512
rs2832129
rs61874707
rs704859
All − MR Egger
All − Inverse variance weighted
−0.25
0.00
0.25
MR effect size for
' || id:ebi−a−GCST90001852' on 'Ischaemic Stroke, excluding all haemorrhages || id:finn−b−I9_STR_EXH'

M

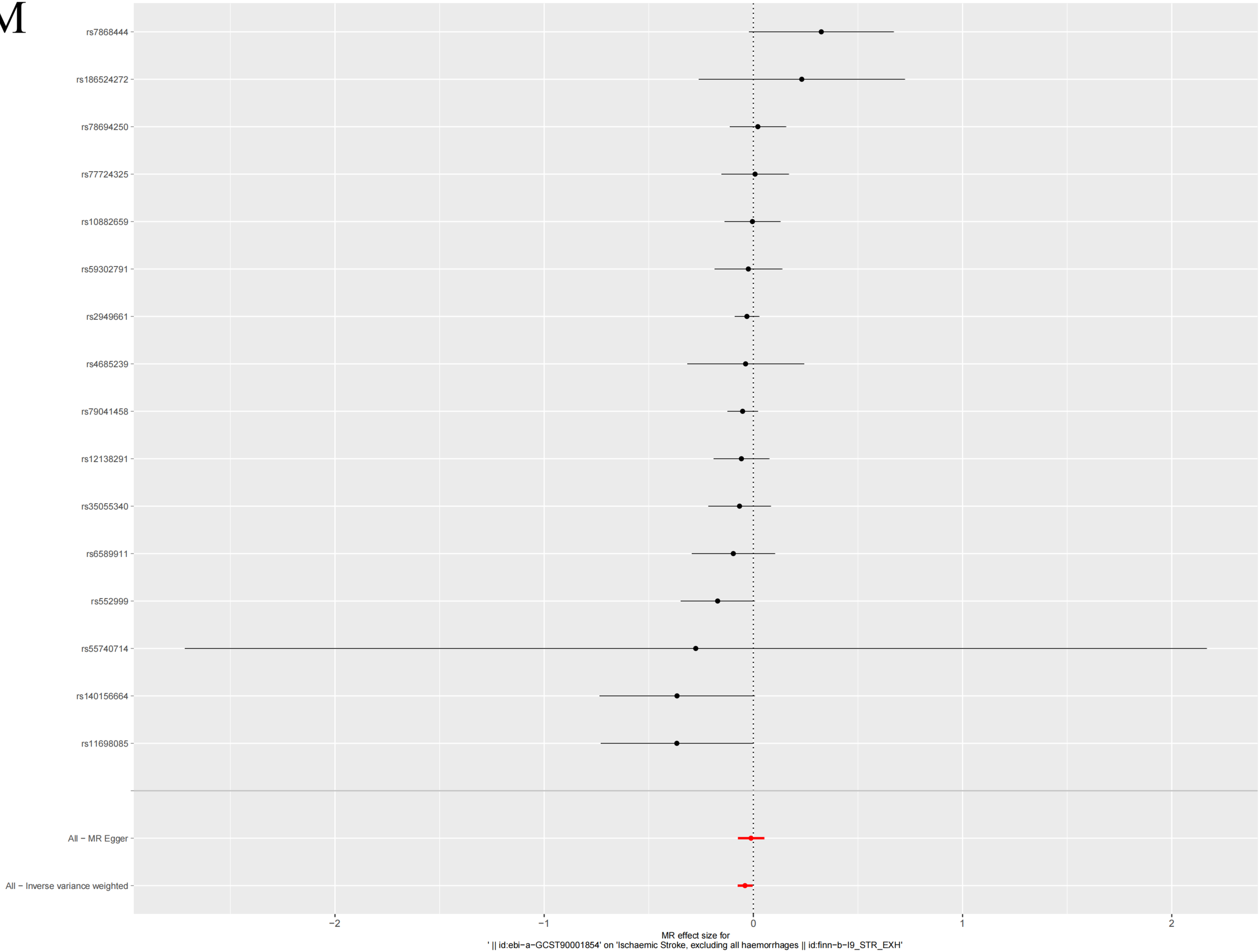

rs7868444
rs186524272
rs78694250
rs77724325
rs10882659
rs59302791
rs2949661
rs4685239
rs79041458
rs12138291
rs35055340
rs6589911
rs552999
rs55740714
rs140156664
rs11698085
All − MR Egger
All − Inverse variance weighted
−2
−1
0
1
2
MR effect size for
' || id:ebi−a−GCST90001854' on 'Ischaemic Stroke, excluding all haemorrhages || id:finn−b−I9_STR_EXH'

N

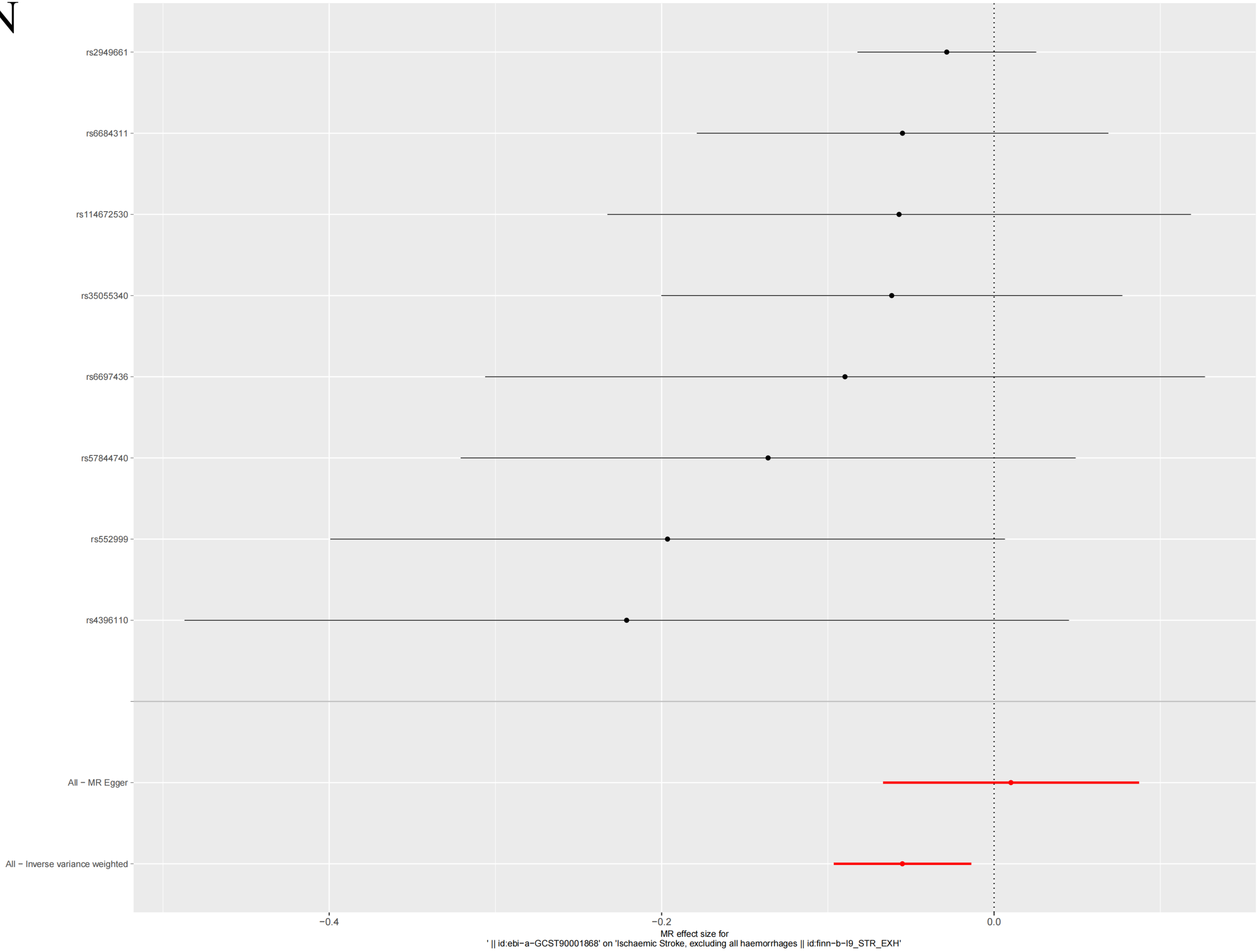
rs2949661
rs6684311
rs114672530
rs35055340
rs6697436
rs57844740
rs552999
rs4396110
All − MR Egger
All − Inverse variance weighted
−0.4
−0.2
0.0
MR effect size for
' || id:ebi−a−GCST90001868' on 'Ischaemic Stroke, excluding all haemorrhages || id:finn−b−I9_STR_EXH'

O

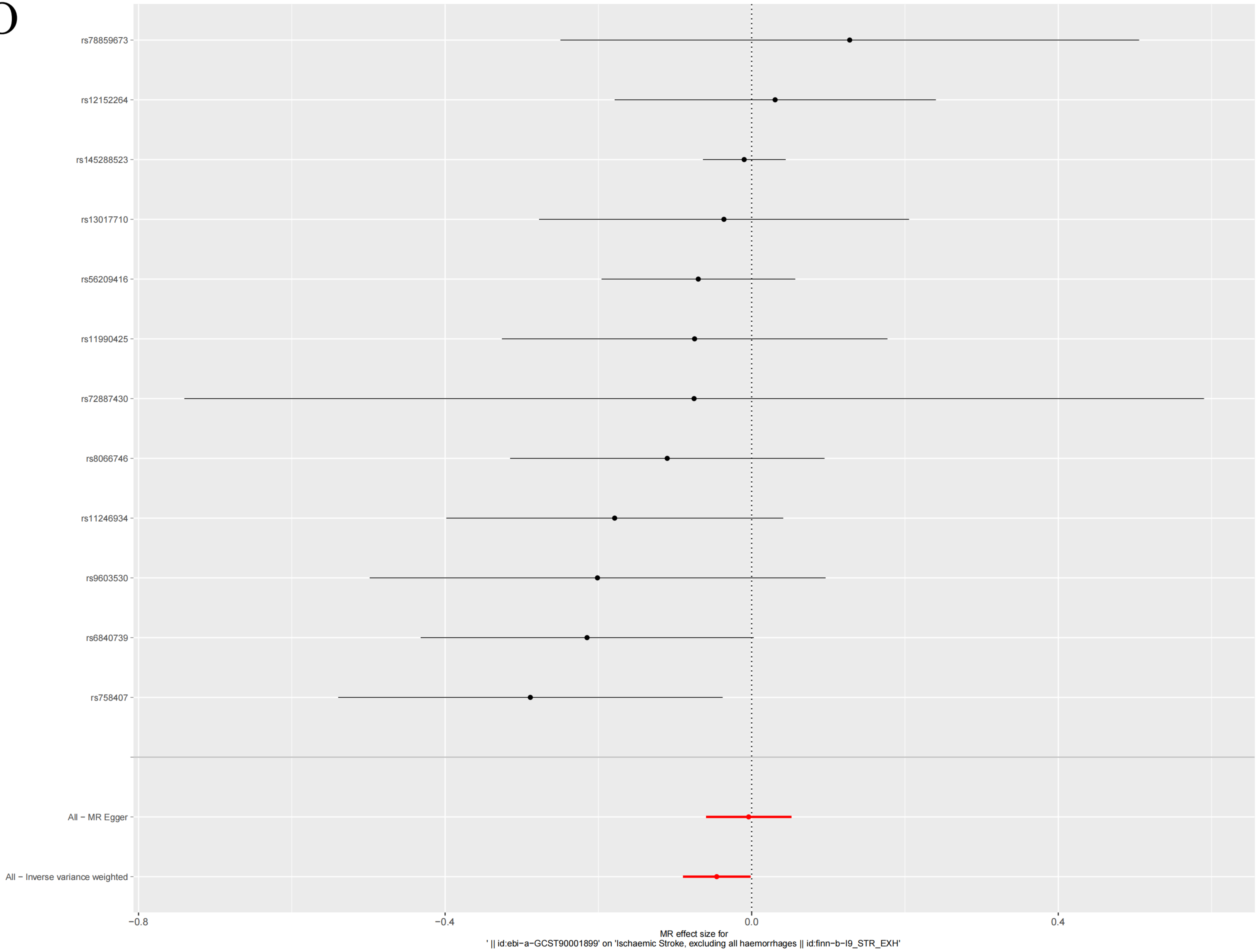
rs78859673
rs12152264
rs145288523
rs13017710
rs56209416
rs11990425
rs72887430
rs8066746
rs11246934
rs9603530
rs6840739
rs758407
All − MR Egger
All − Inverse variance weighted
−0.8
−0.4
0.0
0.4
MR effect size for
' || id:ebi−a−GCST90001899' on 'Ischaemic Stroke, excluding all haemorrhages || id:finn−b−I9_STR_EXH'

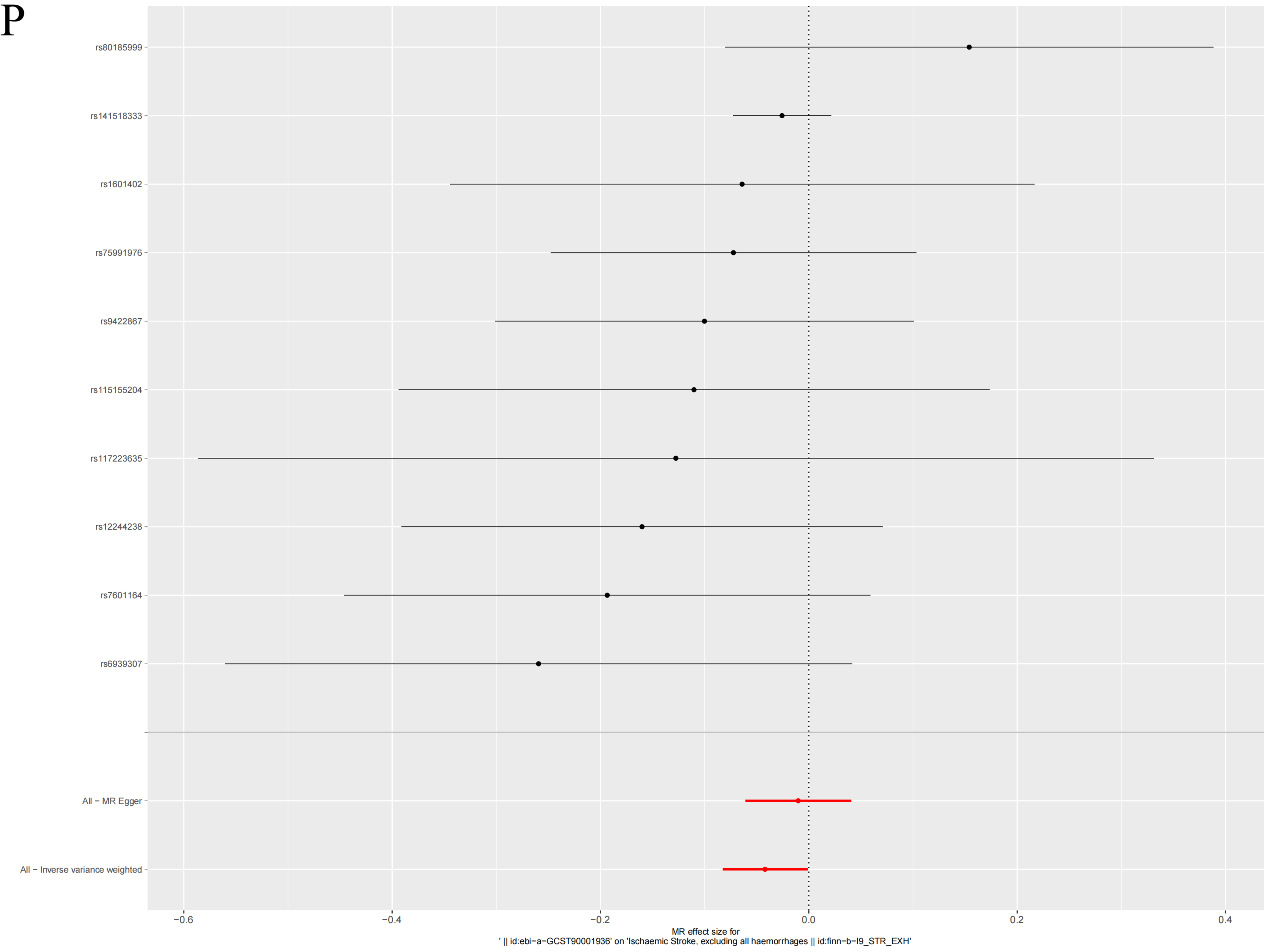
P
rs80185999
rs141518333
rs1601402
rs75991976
rs9422867
rs115155204
rs117223635
rs12244238
rs7601164
rs6939307
All − MR Egger
All − Inverse variance weighted
−0.6
−0.4
−0.2
0.0
0.2
0.4
MR effect size for
' || id:ebi−a−GCST90001936' on 'Ischaemic Stroke, excluding all haemorrhages || id:finn−b−I9_STR_EXH'

Q

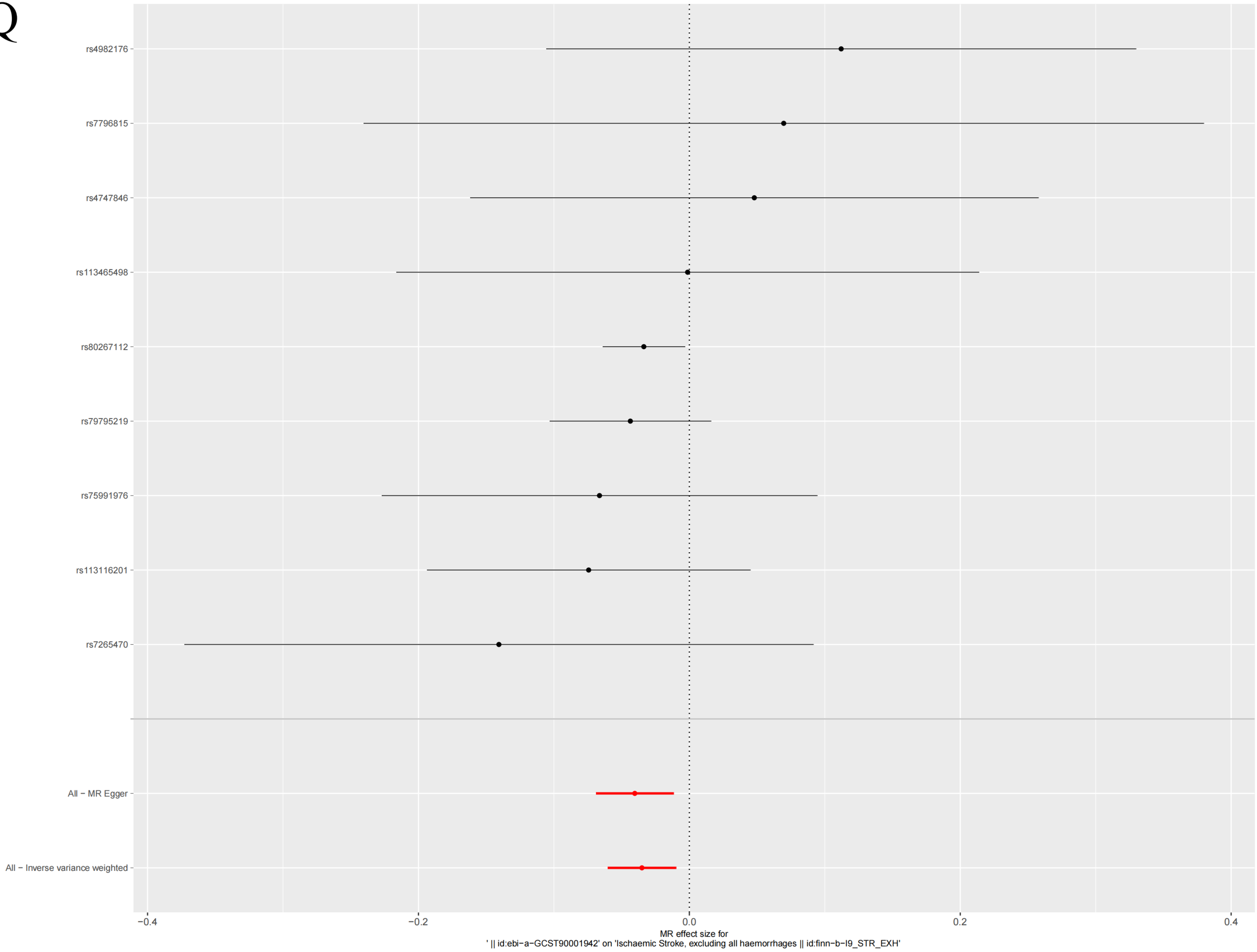
rs4982176
rs7796815
rs4747846
rs113465498
rs80267112
rs79795219
rs75991976
rs113116201
rs7265470
All − MR Egger
All − Inverse variance weighted
−0.4
−0.2
0.0
0.2
0.4
MR effect size for
' || id:ebi−a−GCST90001942' on 'Ischaemic Stroke, excluding all haemorrhages || id:finn−b−I9_STR_EXH'

R

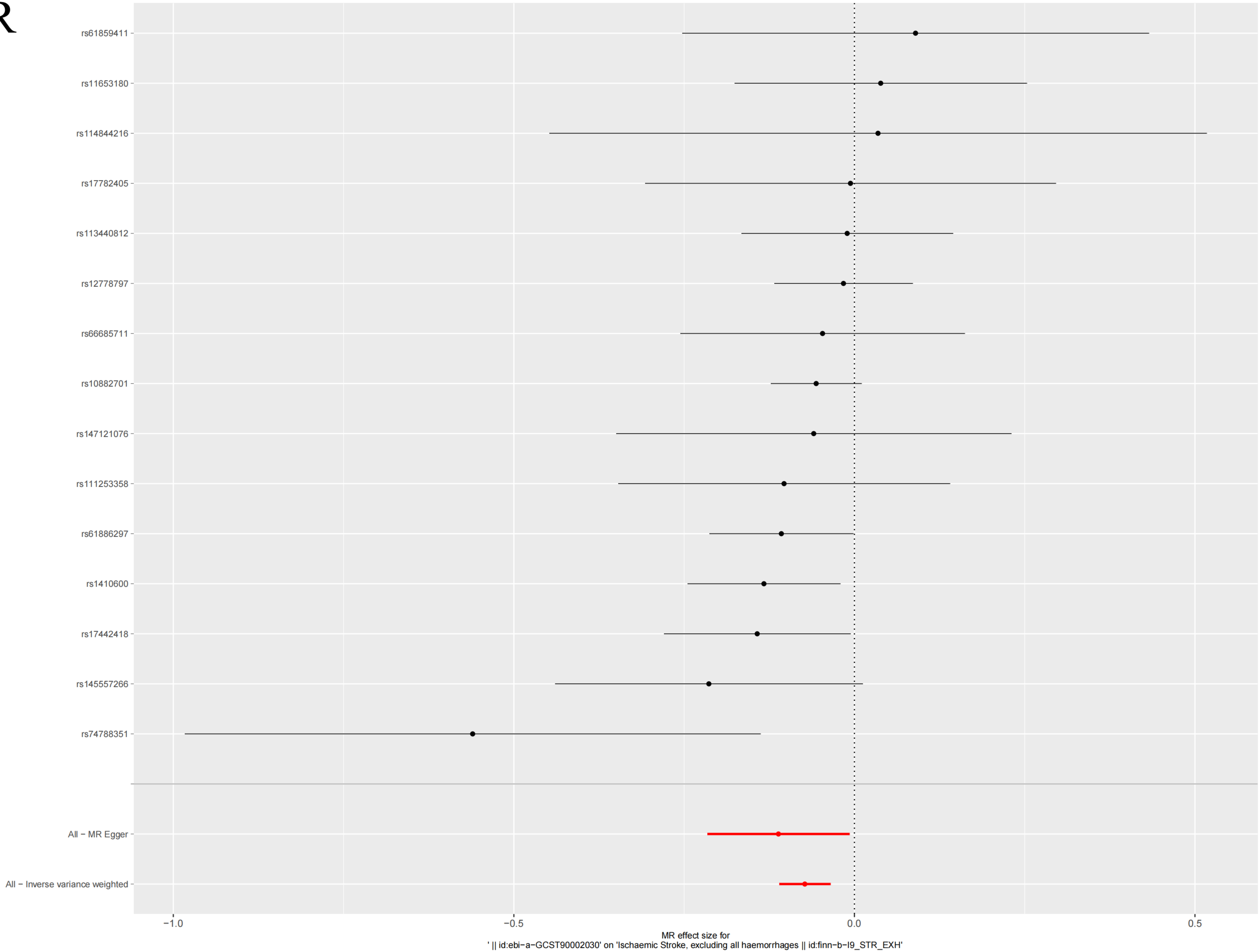
rs61859411
rs11653180
rs114844216
rs17782405
rs113440812
rs12778797
rs66685711
rs10882701
rs147121076
rs111253358
rs61886297
rs1410600
rs17442418
rs145557266
rs74788351
All − MR Egger
All − Inverse variance weighted
−1.0
−0.5
0.0
0.5
MR effect size for
' || id:ebi−a−GCST90002030' on 'Ischaemic Stroke, excluding all haemorrhages || id:finn−b−I9_STR_EXH'

S

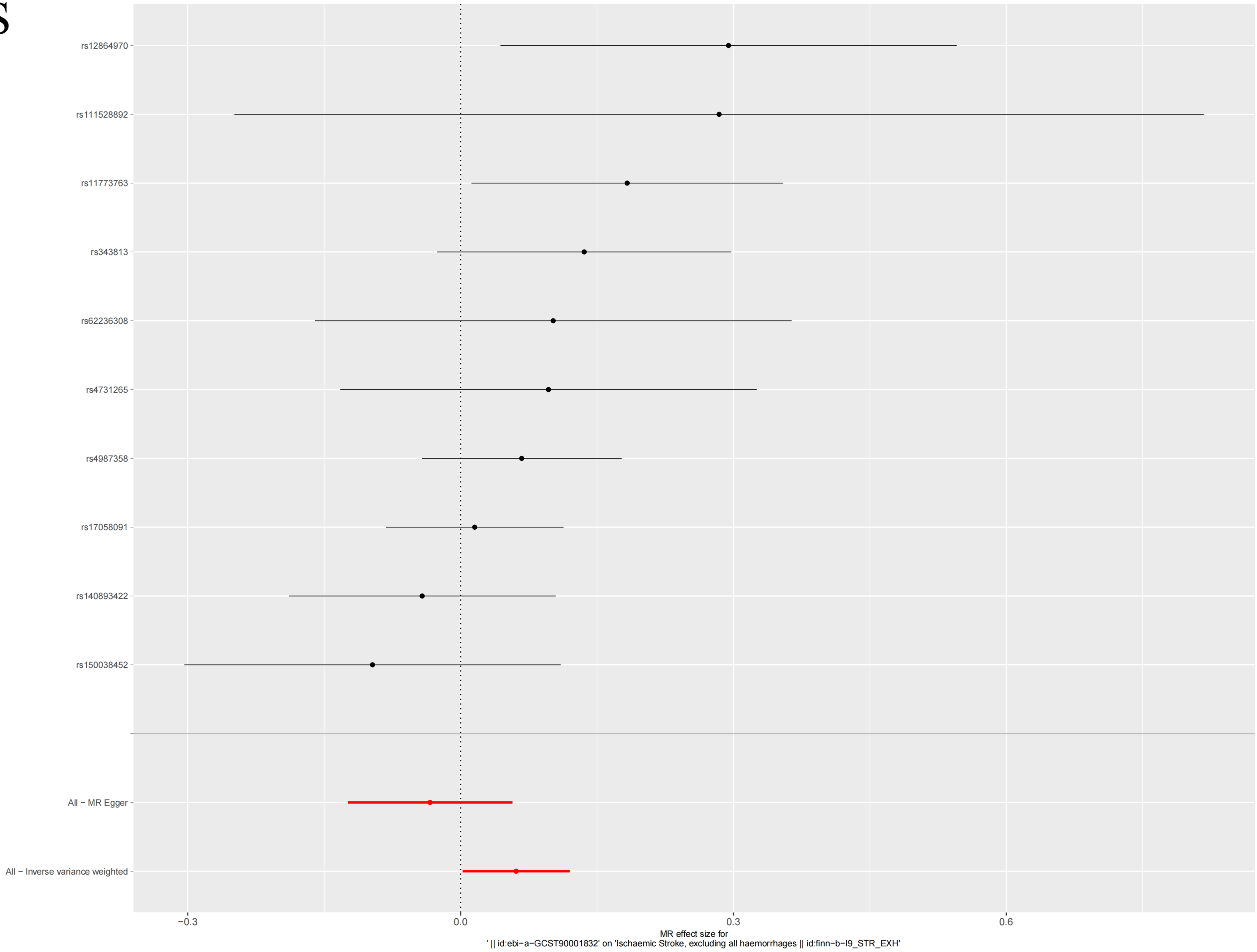
rs12864970
rs111528892
rs11773763
rs343813
rs62236308
rs4731265
rs4987358
rs17058091
rs140893422
rs150038452
All − MR Egger
All − Inverse variance weighted
−0.3
0.0
0.3
0.6
MR effect size for
' || id:ebi−a−GCST90001832' on 'Ischaemic Stroke, excluding all haemorrhages || id:finn−b−I9_STR_EXH'

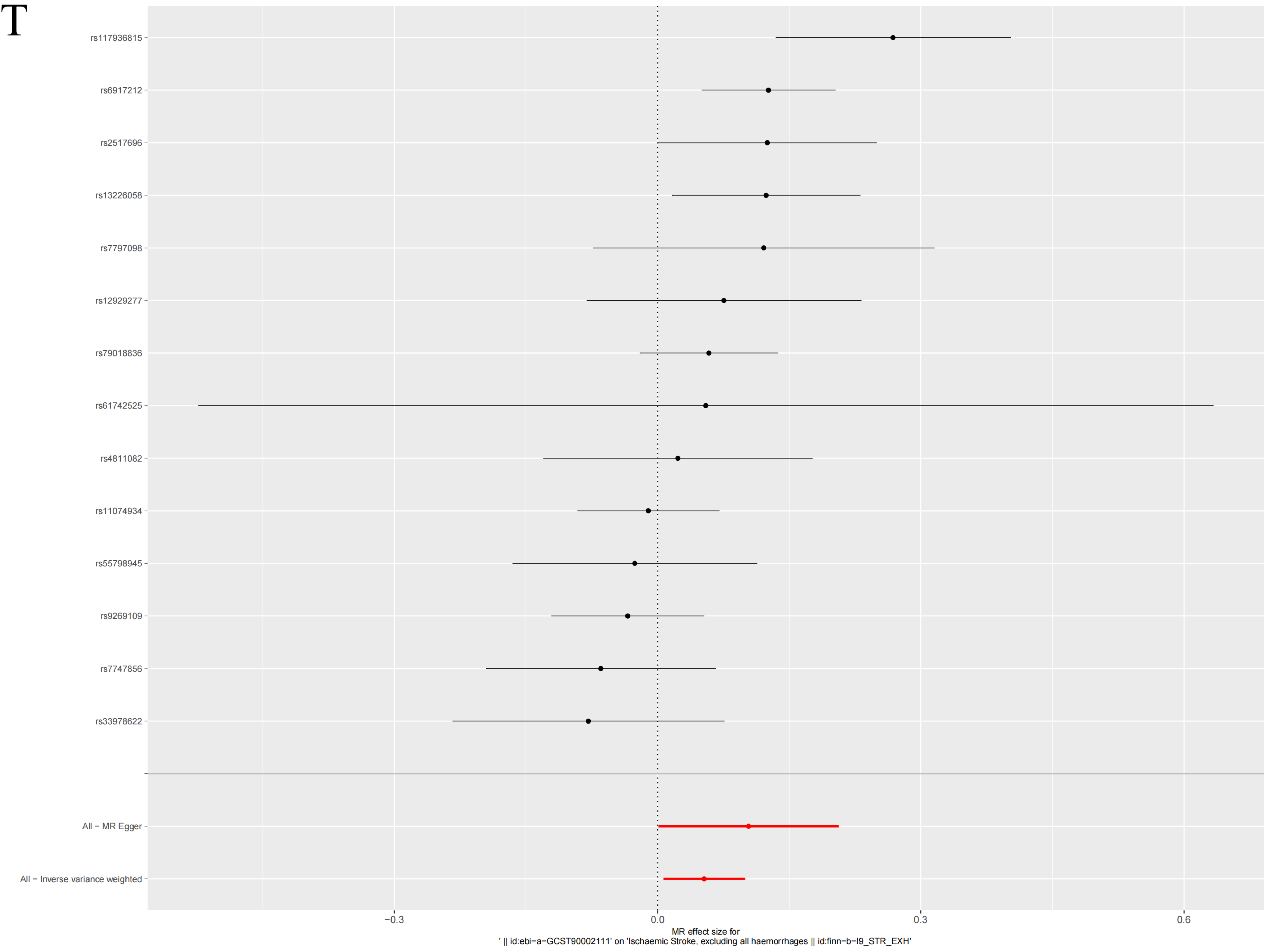
T
rs117936815
rs6917212
rs2517696
rs13226058
rs7797098
rs12929277
rs79018836
rs61742525
rs4811082
rs11074934
rs55798945
rs9269109
rs7747856
rs33978622
All − MR Egger
All − Inverse variance weighted
−0.3
0.0
0.3
0.6
MR effect size for
' || id:ebi−a−GCST90002111' on 'Ischaemic Stroke, excluding all haemorrhages || id:finn−b−I9_STR_EXH'

U

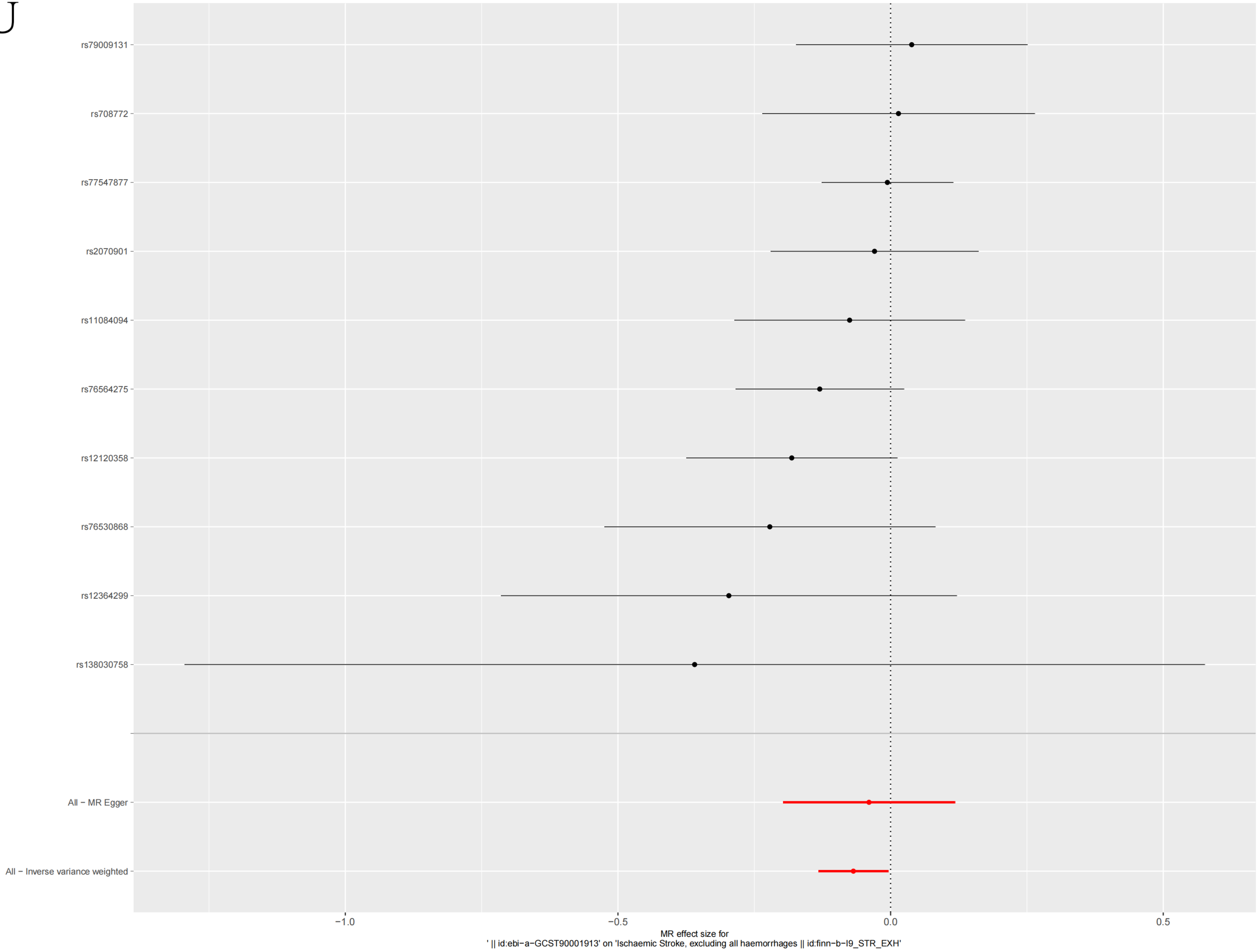
rs79009131
rs708772
rs77547877
rs2070901
rs11084094
rs76564275
rs12120358
rs76530868
rs12364299
rs138030758
All − MR Egger
All − Inverse variance weighted
−1.0
−0.5
0.0
0.5
MR effect size for
' || id:ebi−a−GCST90001913' on 'Ischaemic Stroke, excluding all haemorrhages || id:finn−b−I9_STR_EXH'

Supplementary Figure 2. Sensitivity analysis of forward Mendelian randomization (Funnel plot).

A. Funnel plot between BAFF-R on IgD+ CD38- unswitched memory B cell and IS; B. Funnel plot between CD27 on CD20- CD38- B cell and IS; C. Funnel plot between CD38 on IgD+ CD24- B cell and IS; D. Funnel plot between CD19 on CD24+ CD27+ B cell and IS; E. Funnel plot between CD19 on memory B cell and IS; F. Funnel plot between CD25 on naive-mature B cell and IS; G. Funnel plot between CD25 on CD45RA+ CD4 not regulatory T cell and IS; H. Funnel plot between CD39 on CD39+ CD8+ T cell and IS; I. Funnel plot between HLA DR on HLA DR+ CD4+ T cell and IS; J. Funnel plot between CD8 on CD28+ CD45RA- CD8+ T cell and IS; K. Funnel plot between HVEM on naive CD8+ T cell and IS; L. Funnel plot between CD3 on CD39+ resting CD4 regulatory T cell and IS; M. Funnel plot between CD3 on CD39+ activated CD4 regulatory T cell and IS; N. Funnel plot between CD3 on CD4 regulatory T cell and IS; O. Funnel plot between CD28 on CD4 regulatory T cell and IS; P. Funnel plot between CD25 on CD4 regulatory T cell and IS; Q. Funnel plot between CD25 on CD39+ secreting CD4 regulatory T cell and IS; R. Funnel plot between CD39 on CD39+ activated CD4 regulatory T cell and IS; S. Funnel plot between CD62L on CD62L+ plasmacytoid Dendritic Cell and IS; T. Funnel plot between HLA DR on CD33dim HLA DR+ CD11b- and IS; U. Funnel plot between CD45 on granulocyte and IS;

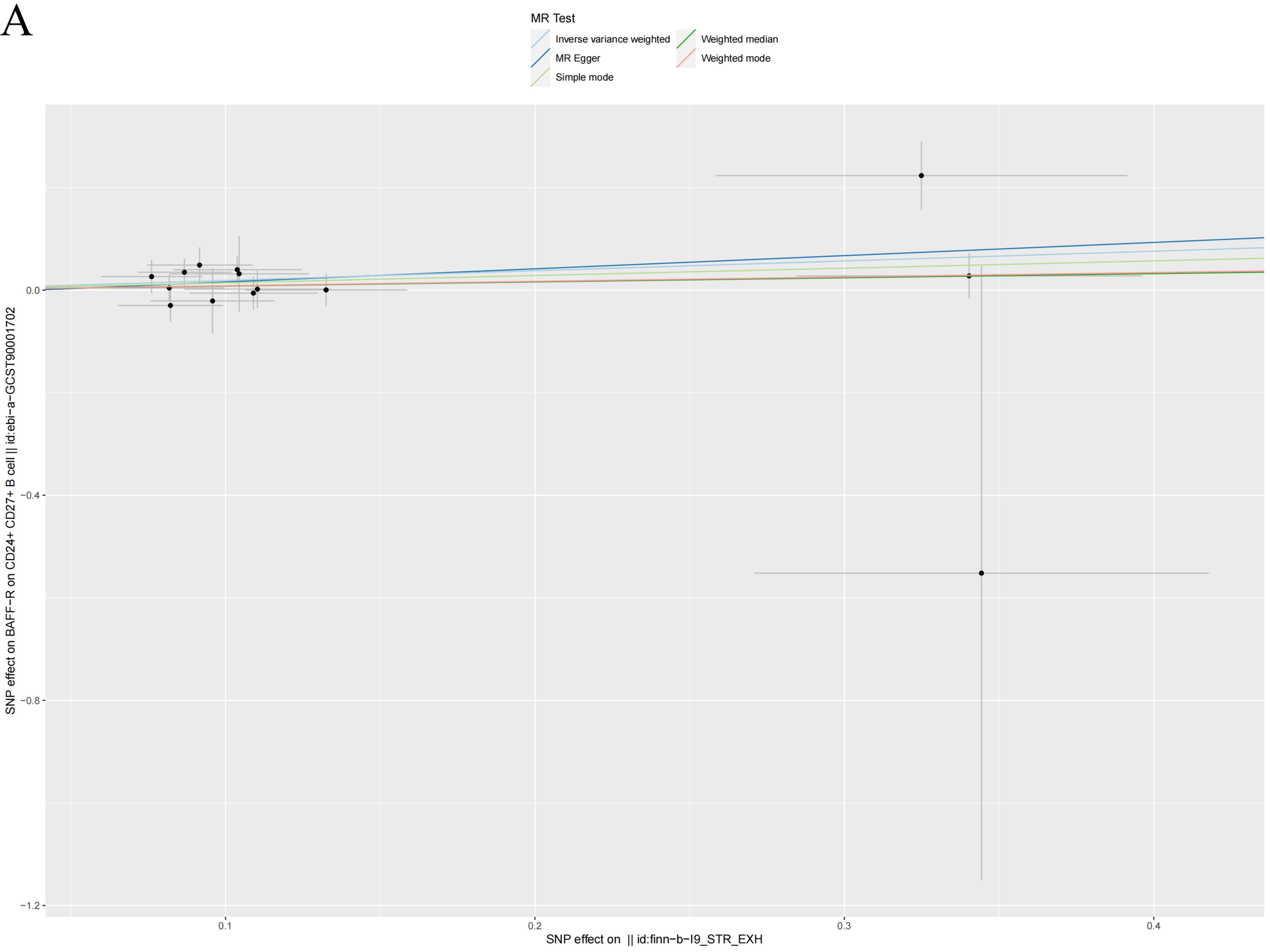
A
MR Test
Inverse variance weighted
MR Egger
Simple mode
Weighted median
Weighted mode
SNP effect on BAFF−R on CD24+ CD27+ B cell || id:ebi−a−GCST90001702
0.0
−0.4
−0.8
−1.2
0.1
0.2
0.3
0.4
SNP effect on || id:finn−b−I9_STR_EXH

B

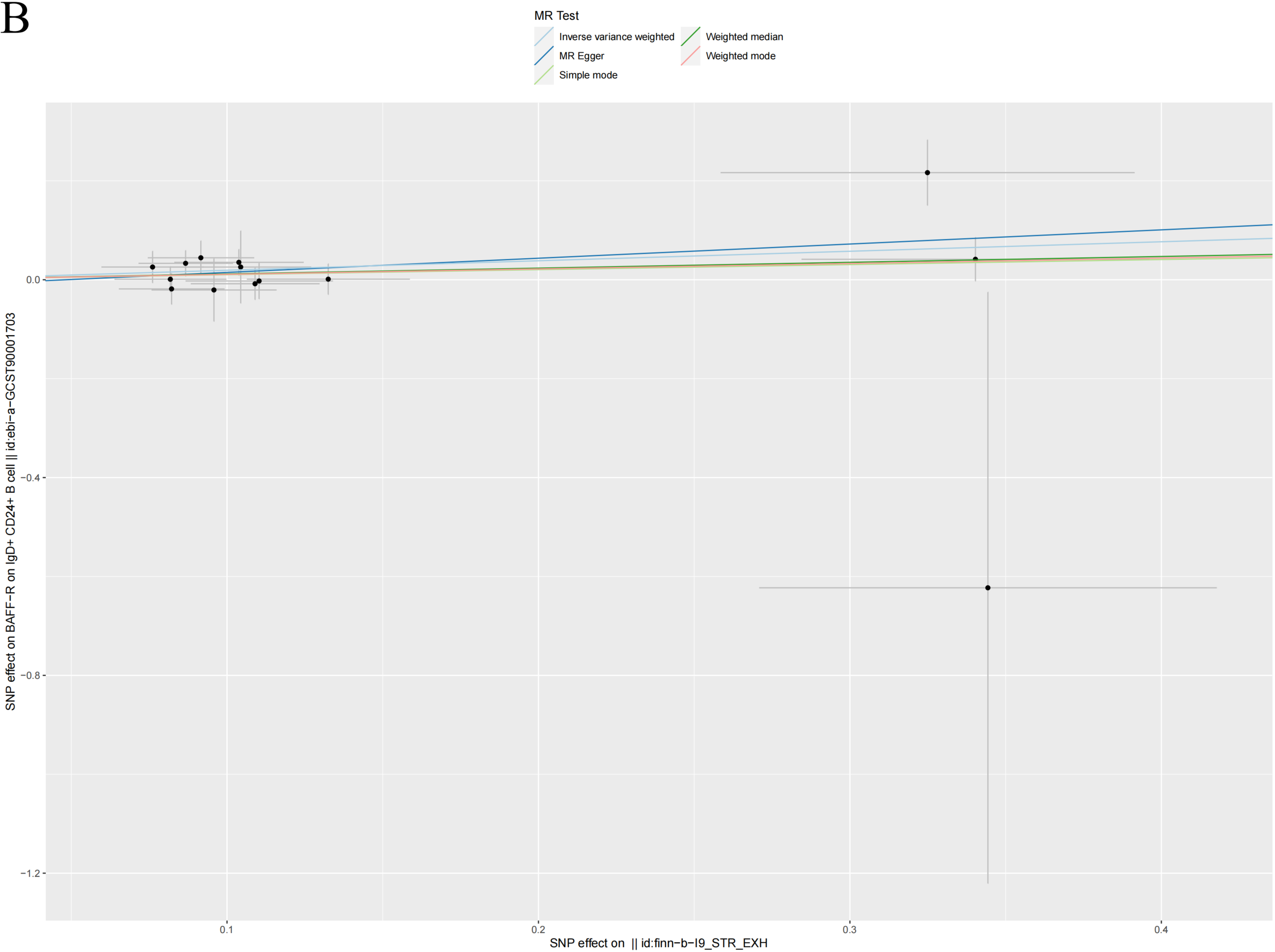

MR Test
Inverse variance weighted
MR Egger
Simple mode
Weighted median
Weighted mode
0.0
-0.4
-0.8
-1.2
0.1
0.2
0.3
0.4
SNP effect on BAFF-R on IgD+ CD24+ B cell || id:ebi-a-GCST90001703
SNP effect on || id:finn-b-I9_STR_EXH

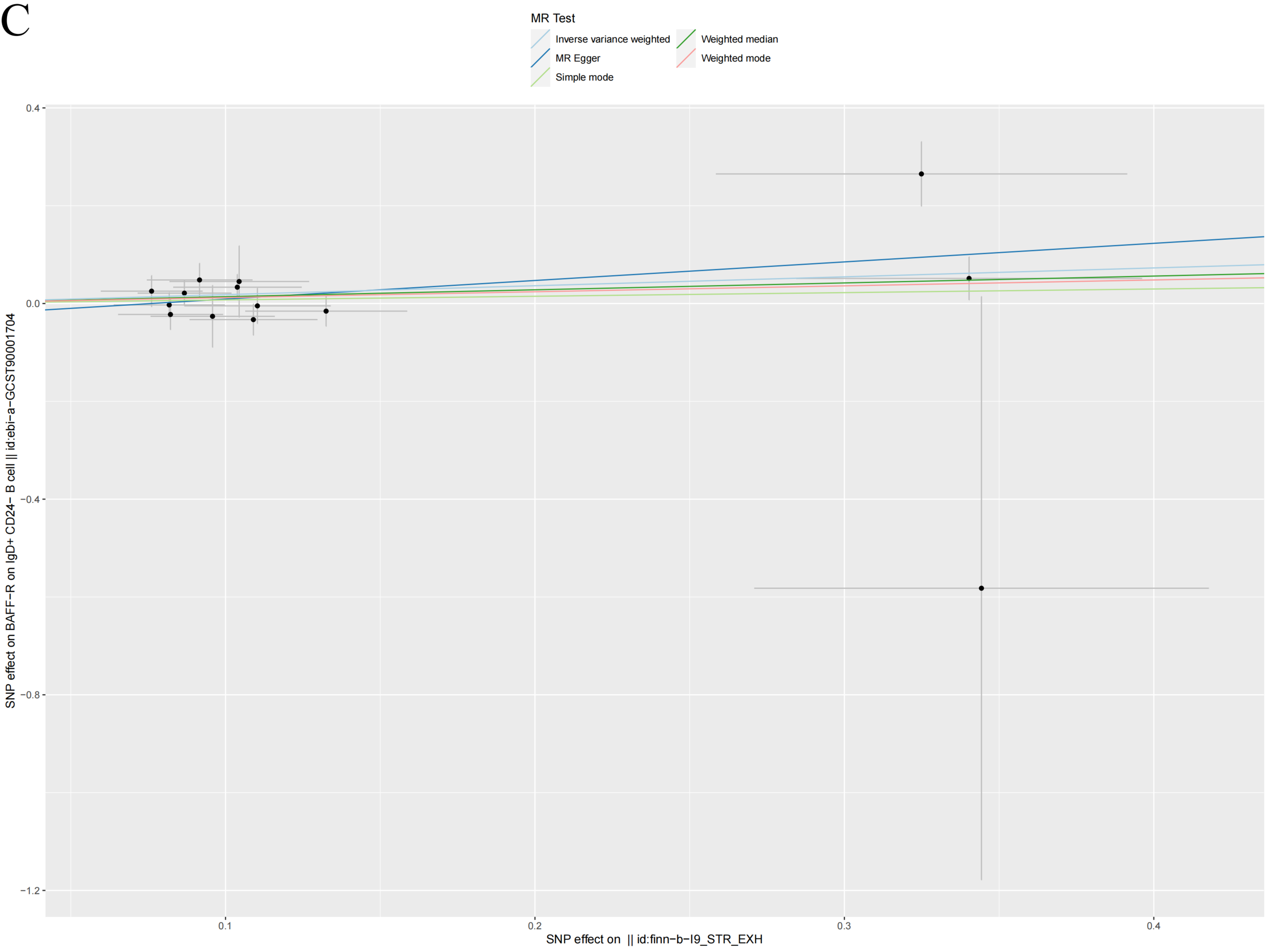

C
MR Test
Inverse variance weighted
MR Egger
Simple mode
Weighted median
Weighted mode
0.4
0.0
−0.4
−0.8
−1.2
0.1
0.2
0.3
0.4
SNP effect on BAFF−R on IgD+ CD24− B cell || id:ebi−a−GCST90001704
SNP effect on || id:finn−b−I9_STR_EXH

D

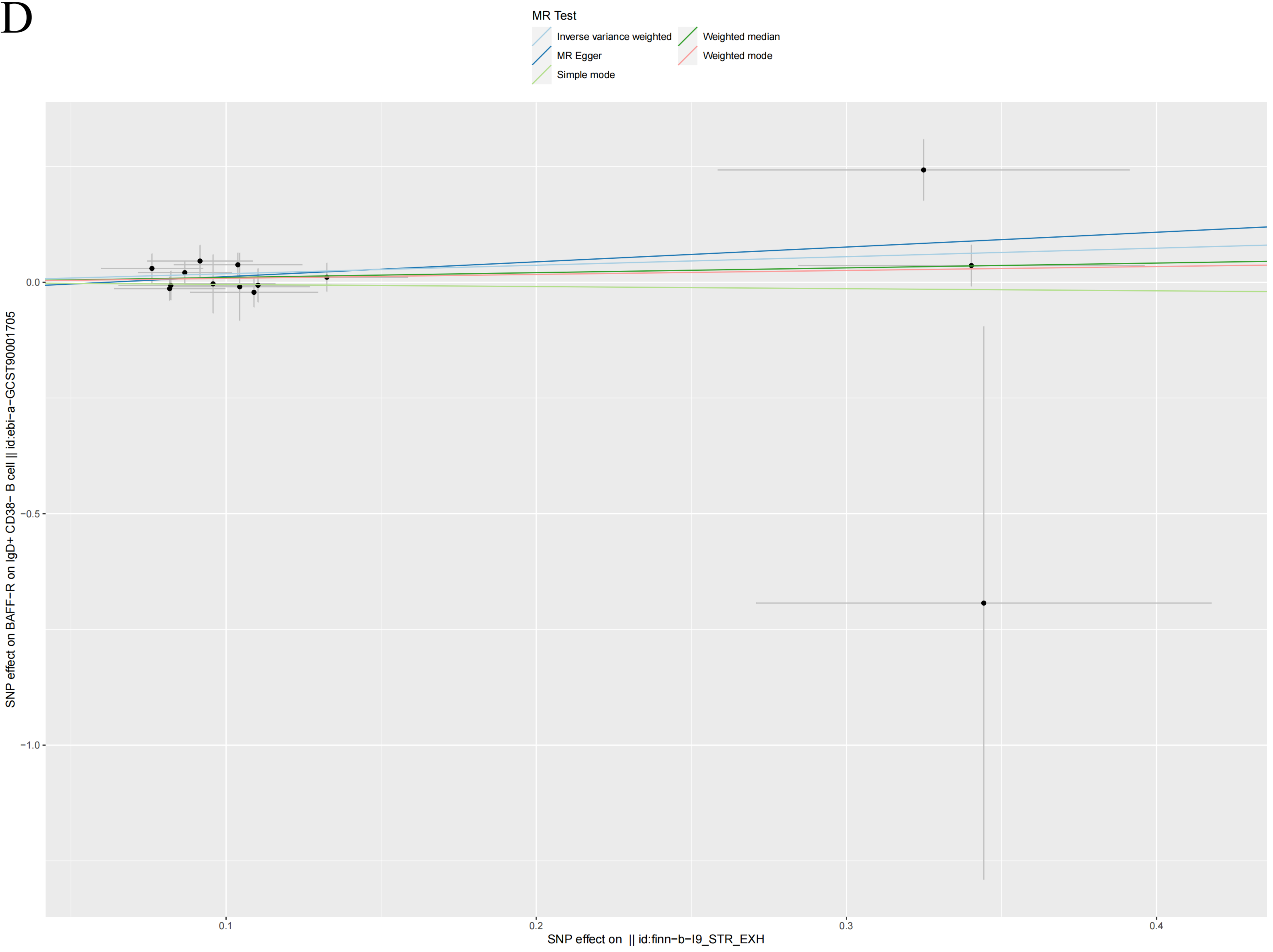
MR Test
Inverse variance weighted
MR Egger
Simple mode
Weighted median
Weighted mode
0.0
−0.5
−1.0
0.1
0.2
0.3
0.4
SNP effect on BAFF−R on IgD+ CD38− B cell || id:ebi−a−GCST90001705
SNP effect on || id:finn−b−I9_STR_EXH

E

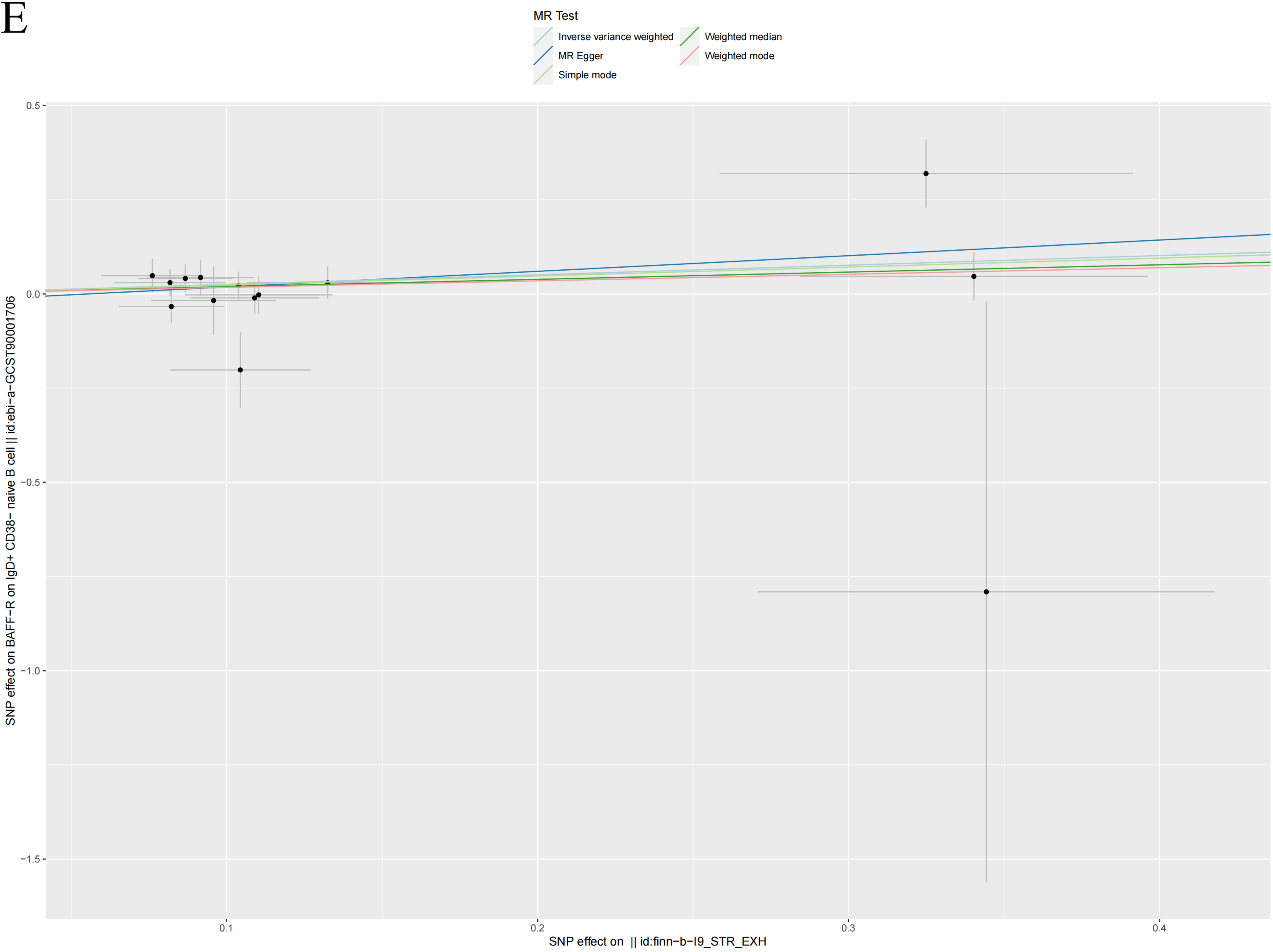
MR Test
Inverse variance weighted
MR Egger
Simple mode
Weighted median
Weighted mode
0.5
0.0
−0.5
−1.0
−1.5
0.1
0.2
0.3
0.4
SNP effect on BAFF−R on IgD+ CD38− naive B cell || id:ebi−a−GCST90001706
SNP effect on || id:finn−b−I9_STR_EXH

F

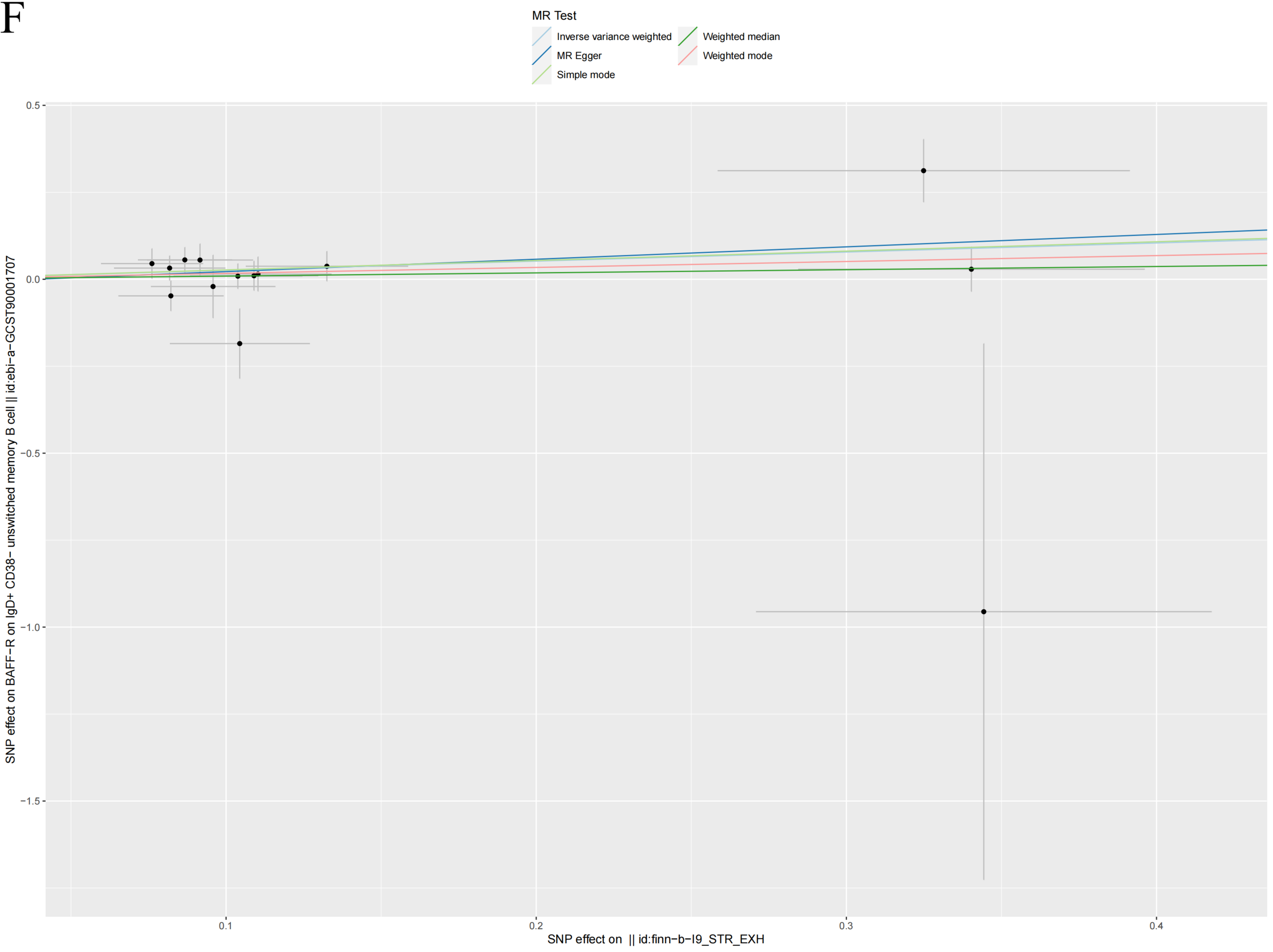

MR Test
Inverse variance weighted
MR Egger
Simple mode
Weighted median
Weighted mode
0.5
0.0
−0.5
−1.0
−1.5
0.1
0.2
0.3
0.4
SNP effect on BAFF−R on IgD+ CD38− unswitched memory B cell || id:ebi−a−GCST90001707
SNP effect on || id:finn−b−I9_STR_EXH

G

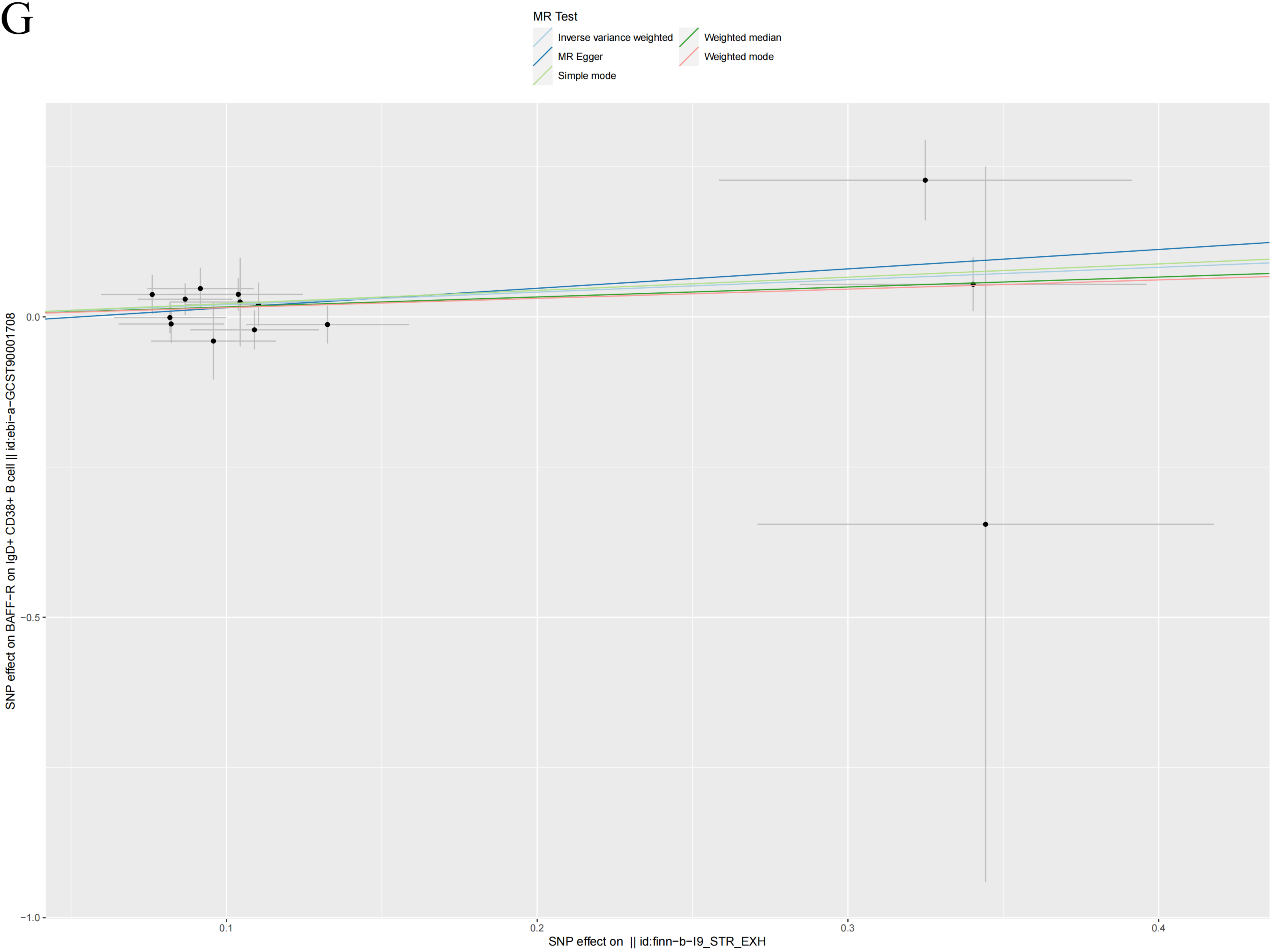
MR Test
Inverse variance weighted
MR Egger
Simple mode
Weighted median
Weighted mode
SNP effect on BAFF−R on IgD+ CD38+ B cell || id:ebi−a−GCST90001708
0.0
−0.5
−1.0
0.1
0.2
0.3
0.4
SNP effect on || id:finn−b−I9_STR_EXH

H

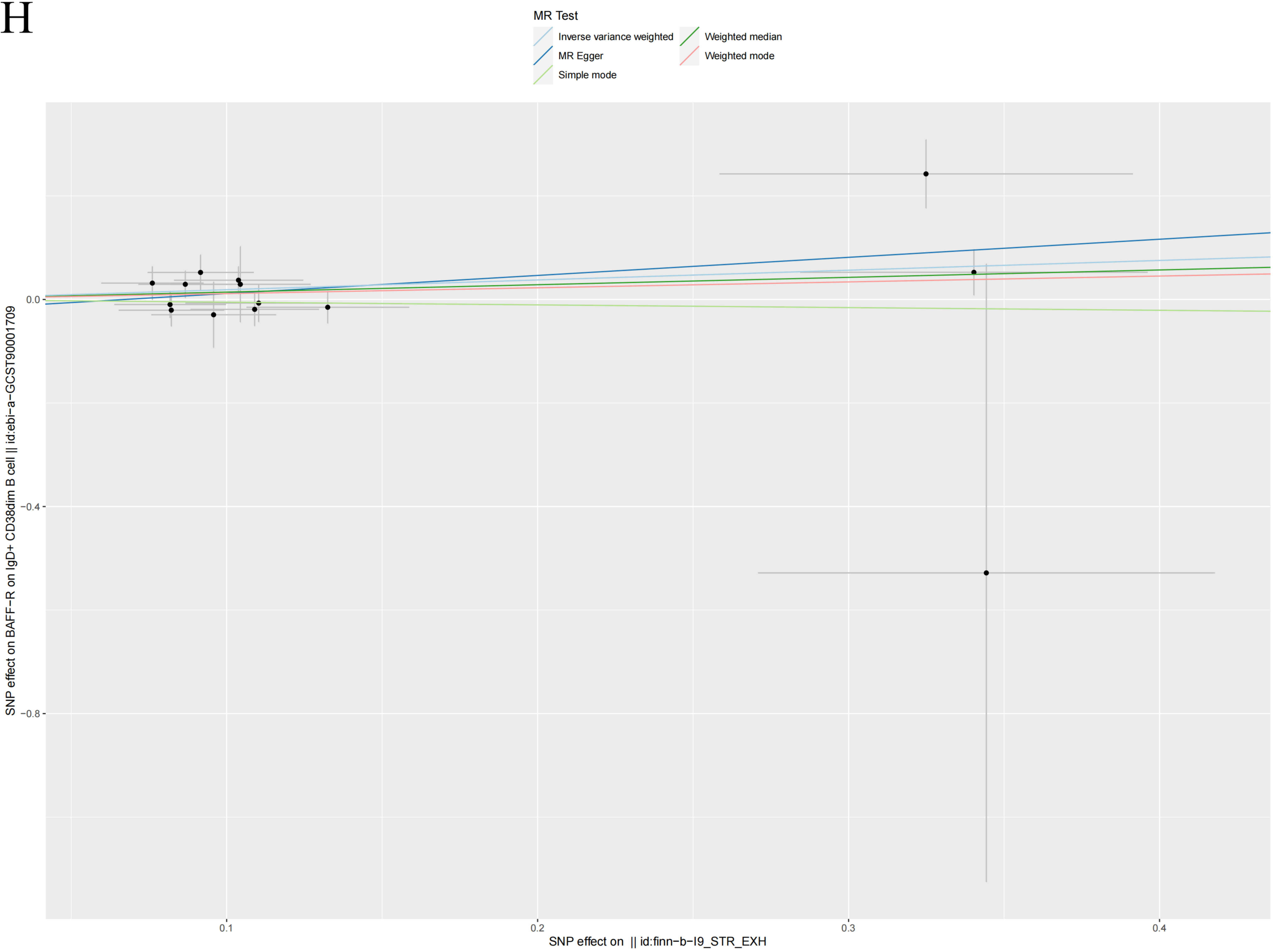

MR Test
Inverse variance weighted
MR Egger
Simple mode
Weighted median
Weighted mode
0.0
−0.4
−0.8
0.1
0.2
0.3
0.4
SNP effect on BAFF−R on IgD+ CD38dim B cell || id:ebi−a−GCST90001709
SNP effect on || id:finn−b−I9_STR_EXH

I

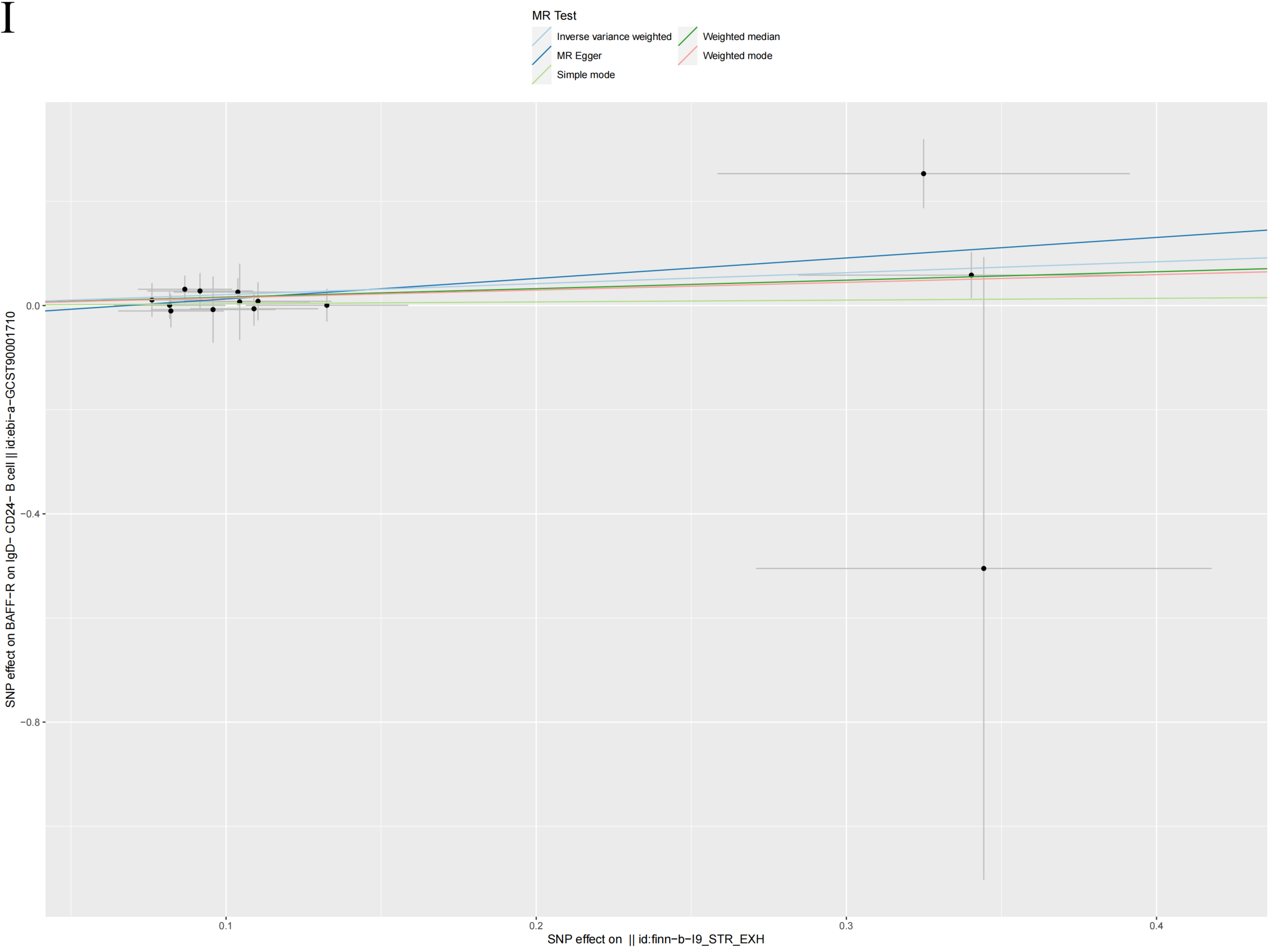

MR Test
Inverse variance weighted
MR Egger
Simple mode
Weighted median
Weighted mode
0.0
−0.4
−0.8
0.1
0.2
0.3
0.4
SNP effect on || id:finn−b−I9_STR_EXH
SNP effect on BAFF−R on IgD− CD24− B cell || id:ebi−a−GCST90001710

J

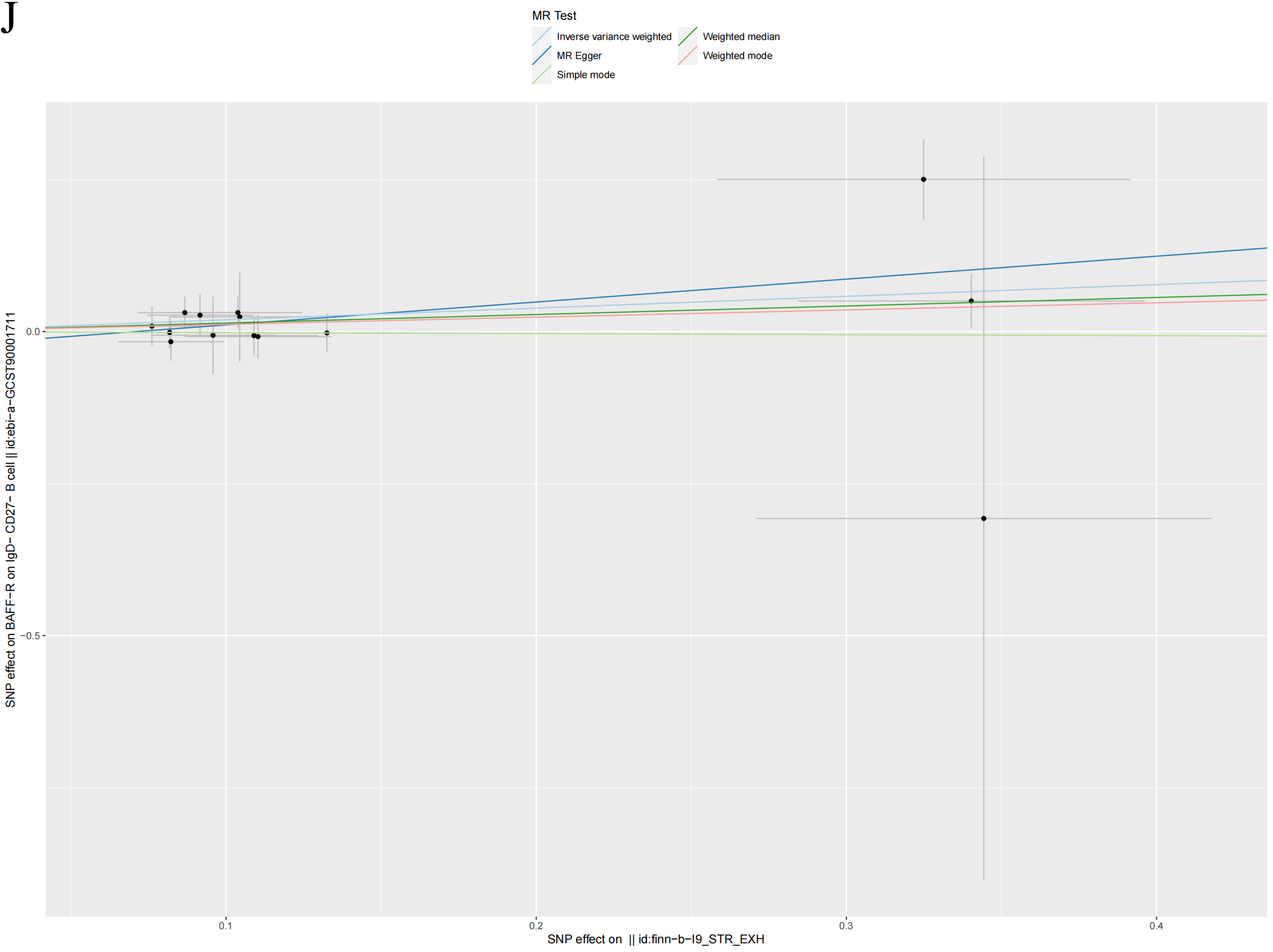

MR Test
Inverse variance weighted
MR Egger
Simple mode
Weighted median
Weighted mode
0.0
−0.5
0.1
0.2
0.3
0.4
SNP effect on BAFF−R on IgD− CD27− B cell || id:ebi−a−GCST90001711
SNP effect on || id:finn−b−I9_STR_EXH

K

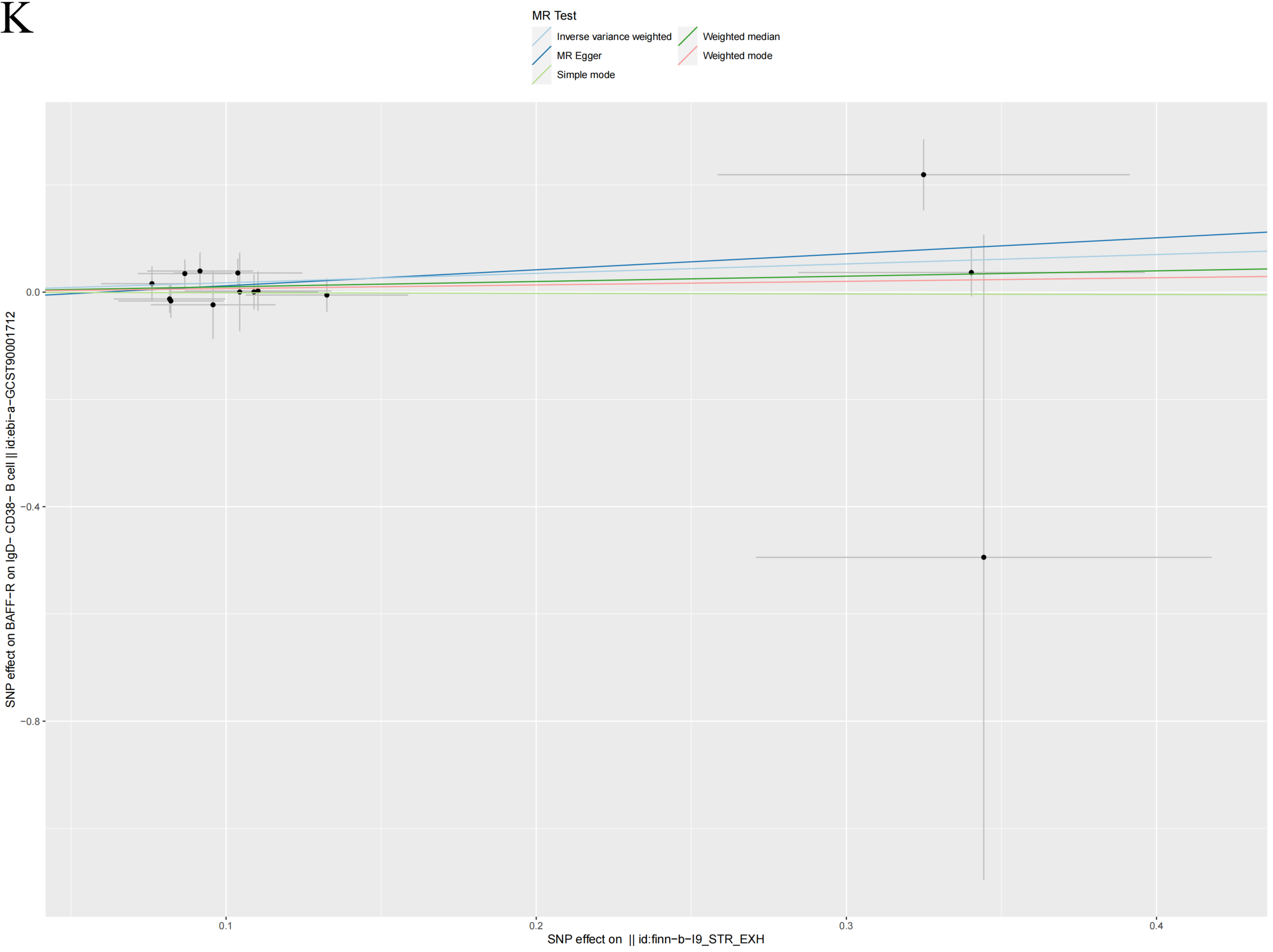
MR Test
Inverse variance weighted
MR Egger
Simple mode
Weighted median
Weighted mode
SNP effect on BAFF−R on IgD− CD38− B cell || id:ebi−a−GCST90001712
0.0
−0.4
−0.8
0.1
0.2
0.3
0.4
SNP effect on || id:finn−b−I9_STR_EXH

L

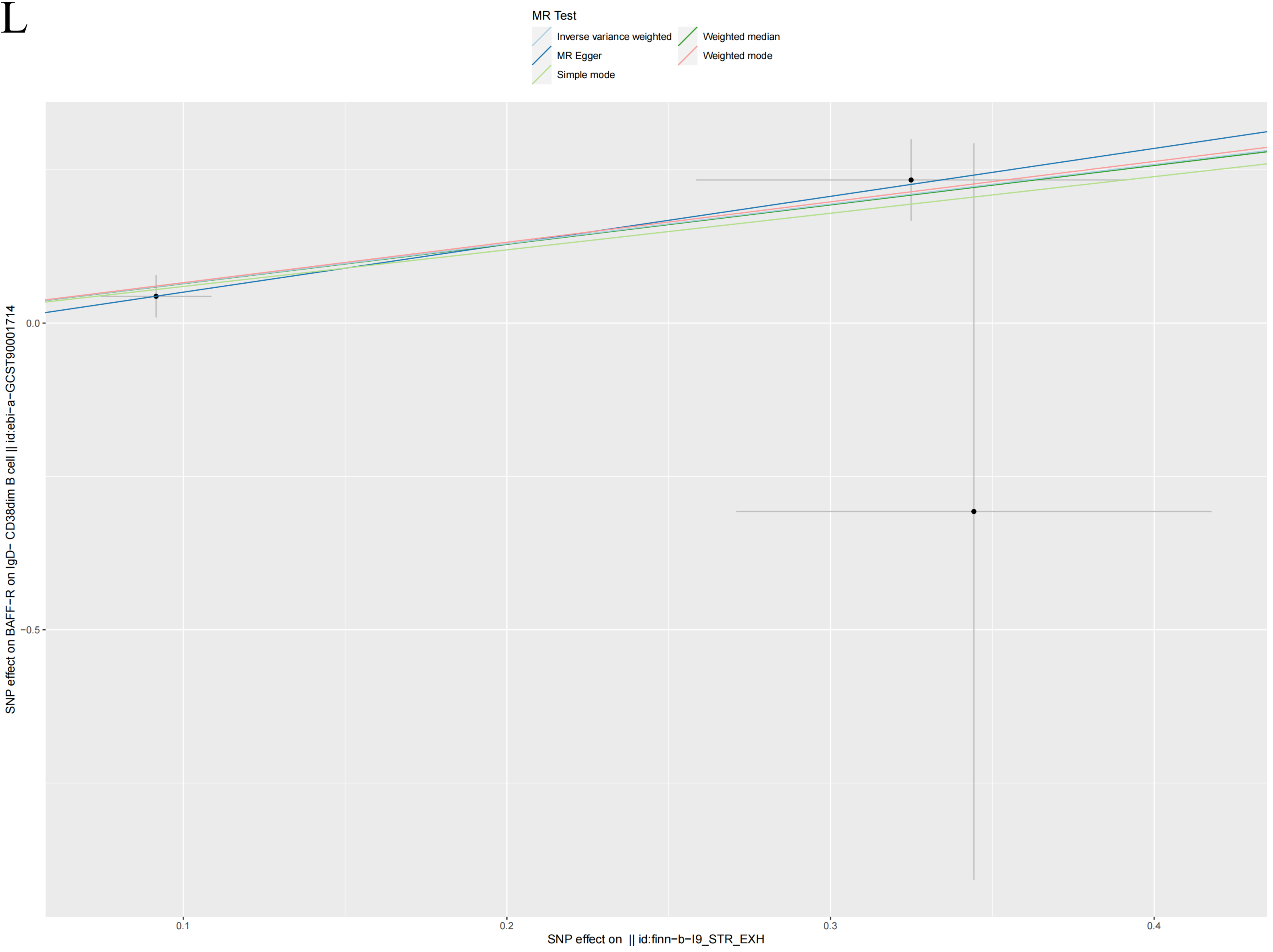
MR Test
Inverse variance weighted
MR Egger
Simple mode
Weighted median
Weighted mode
SNP effect on BAFF−R on IgD− CD38dim B cell || id:ebi−a−GCST90001714
0.0
−0.5
0.1
0.2
0.3
0.4
SNP effect on || id:finn−b−I9_STR_EXH

M

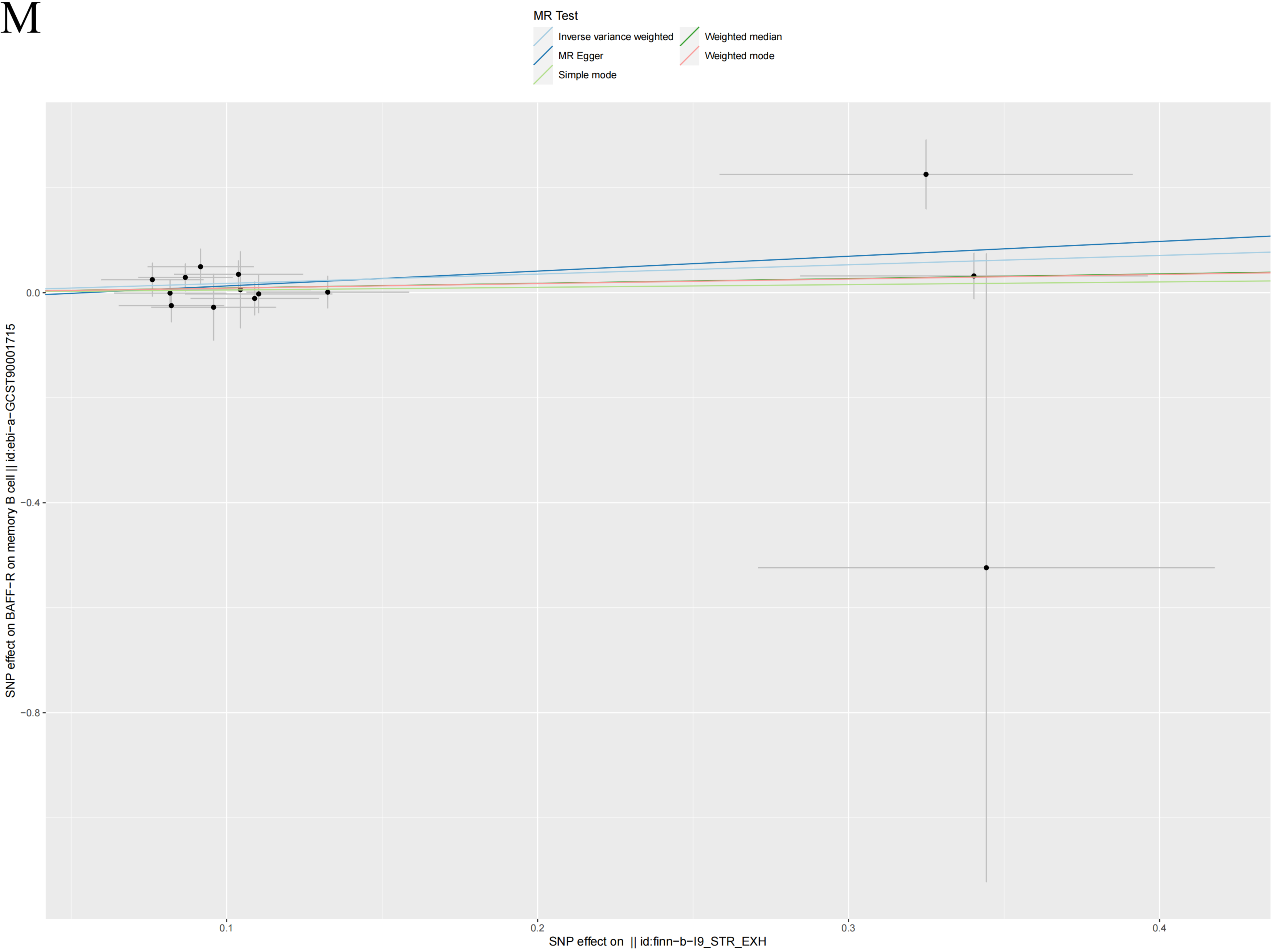

MR Test
Inverse variance weighted
MR Egger
Simple mode
Weighted median
Weighted mode
0.0
−0.4
−0.8
0.1
0.2
0.3
0.4
SNP effect on BAFF−R on memory B cell || id:ebi−a−GCST90001715
SNP effect on || id:finn−b−I9_STR_EXH

N

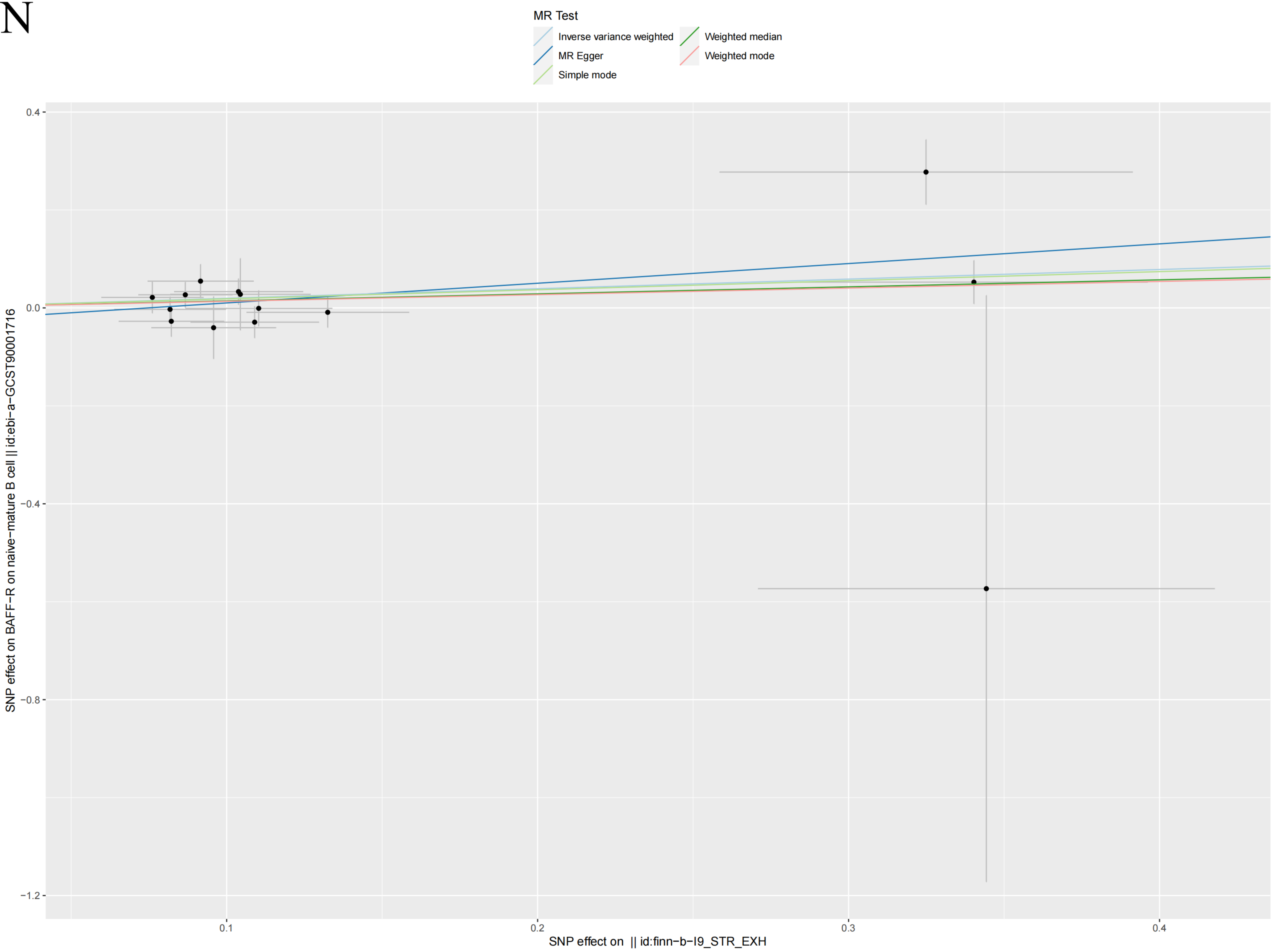

MR Test
Inverse variance weighted
MR Egger
Simple mode
Weighted median
Weighted mode
0.4
0.0
−0.4
−0.8
−1.2
0.1
0.2
0.3
0.4
SNP effect on BAFF−R on naive−mature B cell || id:ebi−a−GCST90001716
SNP effect on || id:finn−b−I9_STR_EXH

O

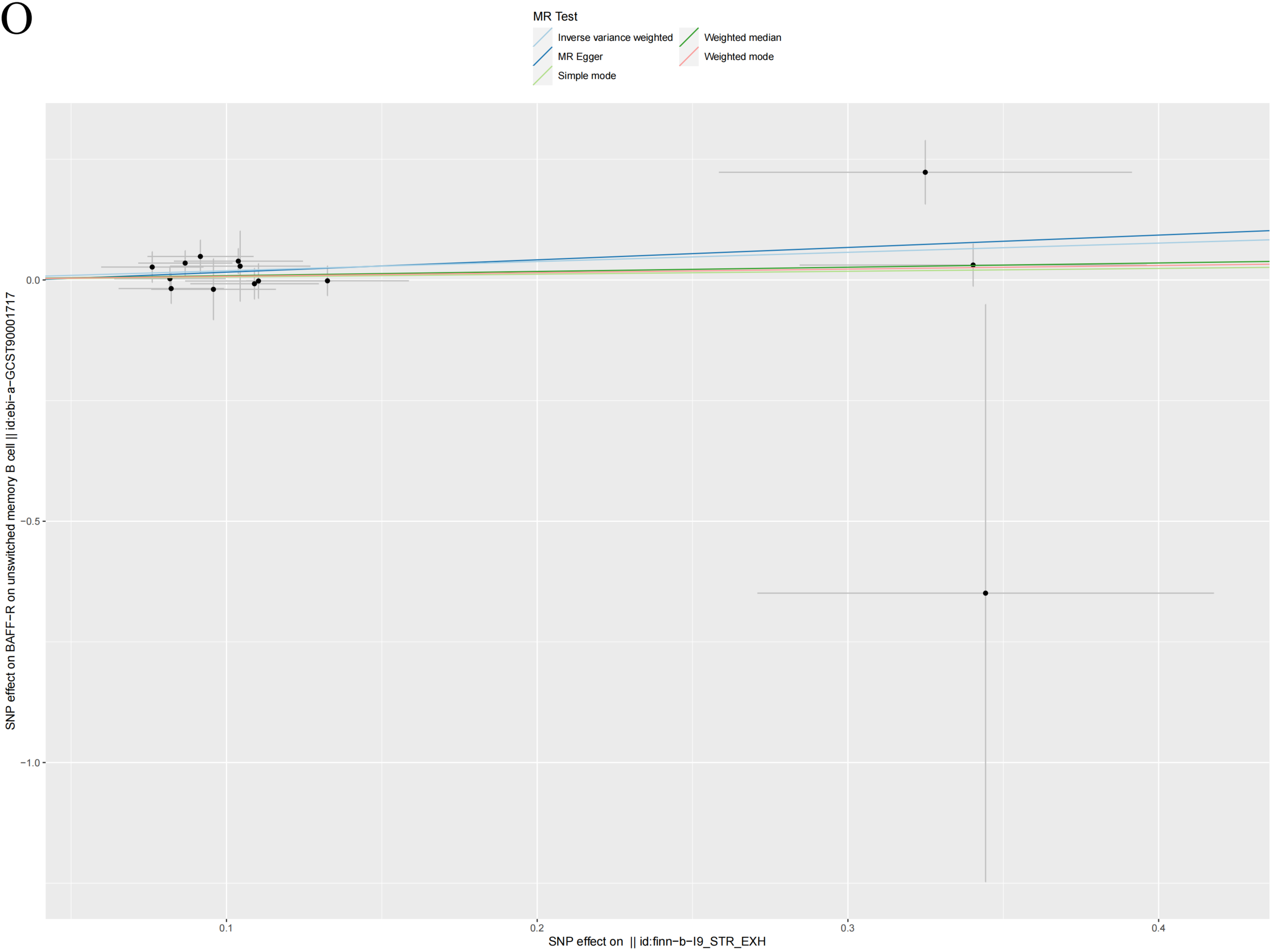
MR Test
Inverse variance weighted
MR Egger
Simple mode
Weighted median
Weighted mode
0.0
−0.5
−1.0
0.1
0.2
0.3
0.4
SNP effect on BAFF−R on unswitched memory B cell || id:ebi−a−GCST90001717
SNP effect on || id:finn−b−I9_STR_EXH

P

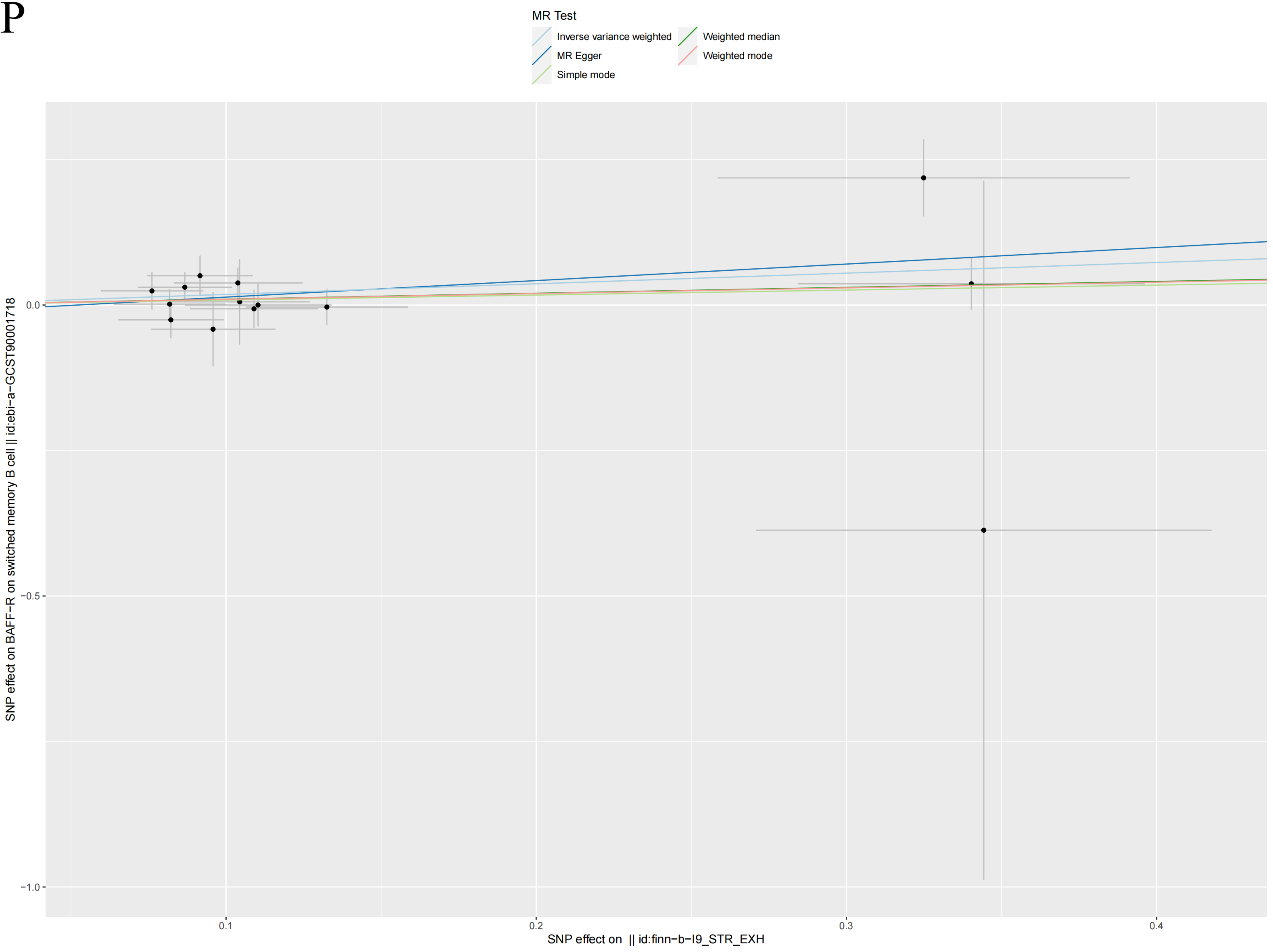
MR Test
Inverse variance weighted
MR Egger
Simple mode
Weighted median
Weighted mode
0.0
−0.5
−1.0
0.1
0.2
0.3
0.4
SNP effect on BAFF−R on switched memory B cell || id:ebi−a−GCST90001718
SNP effect on || id:finn−b−I9_STR_EXH

Q

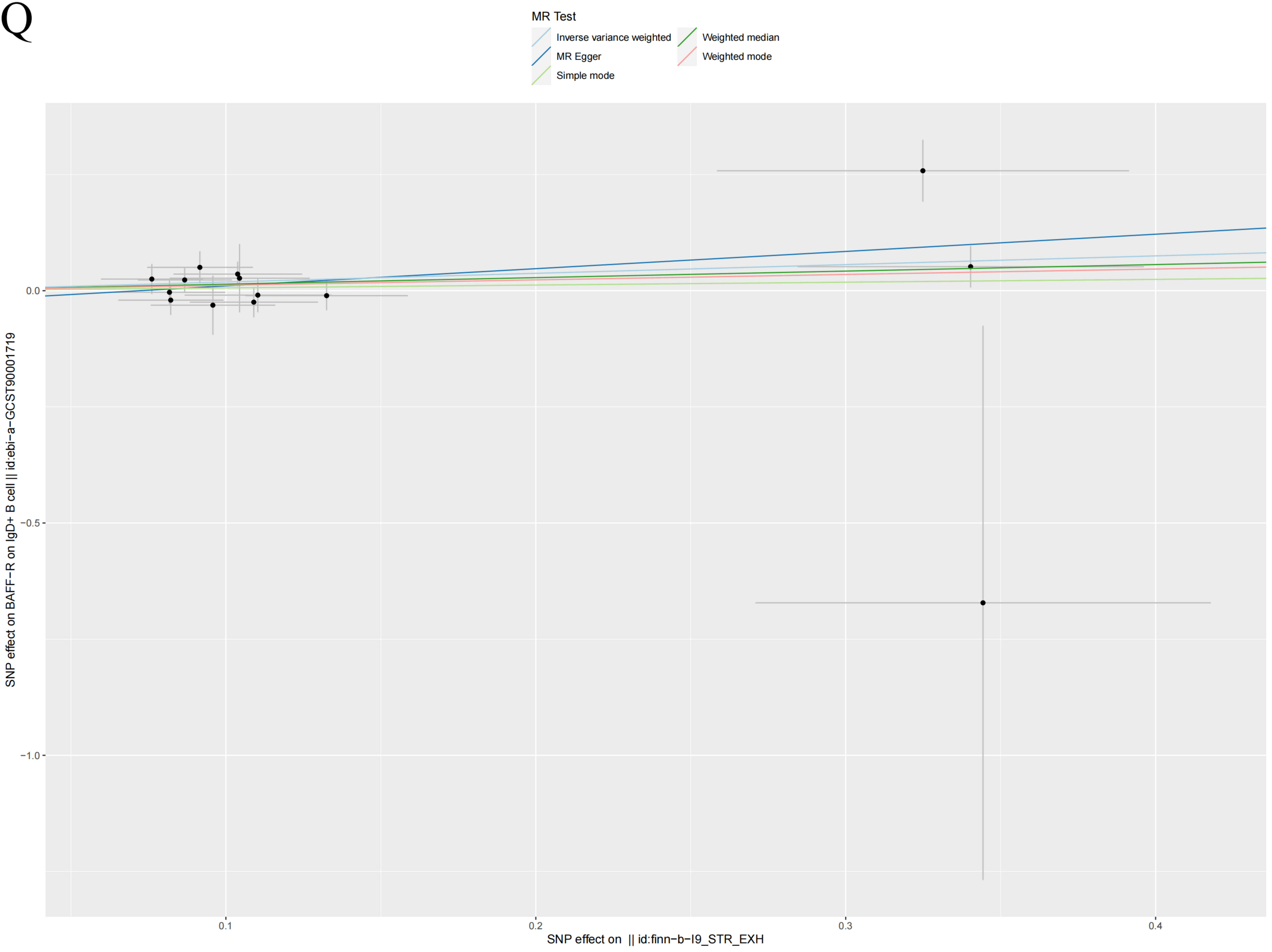
MR Test
Inverse variance weighted
MR Egger
Simple mode
Weighted median
Weighted mode
SNP effect on BAFF−R on IgD+ B cell || id:ebi−a−GCST90001719
SNP effect on || id:finn−b−I9_STR_EXH
0.0
−0.5
−1.0
0.1
0.2
0.3
0.4

R

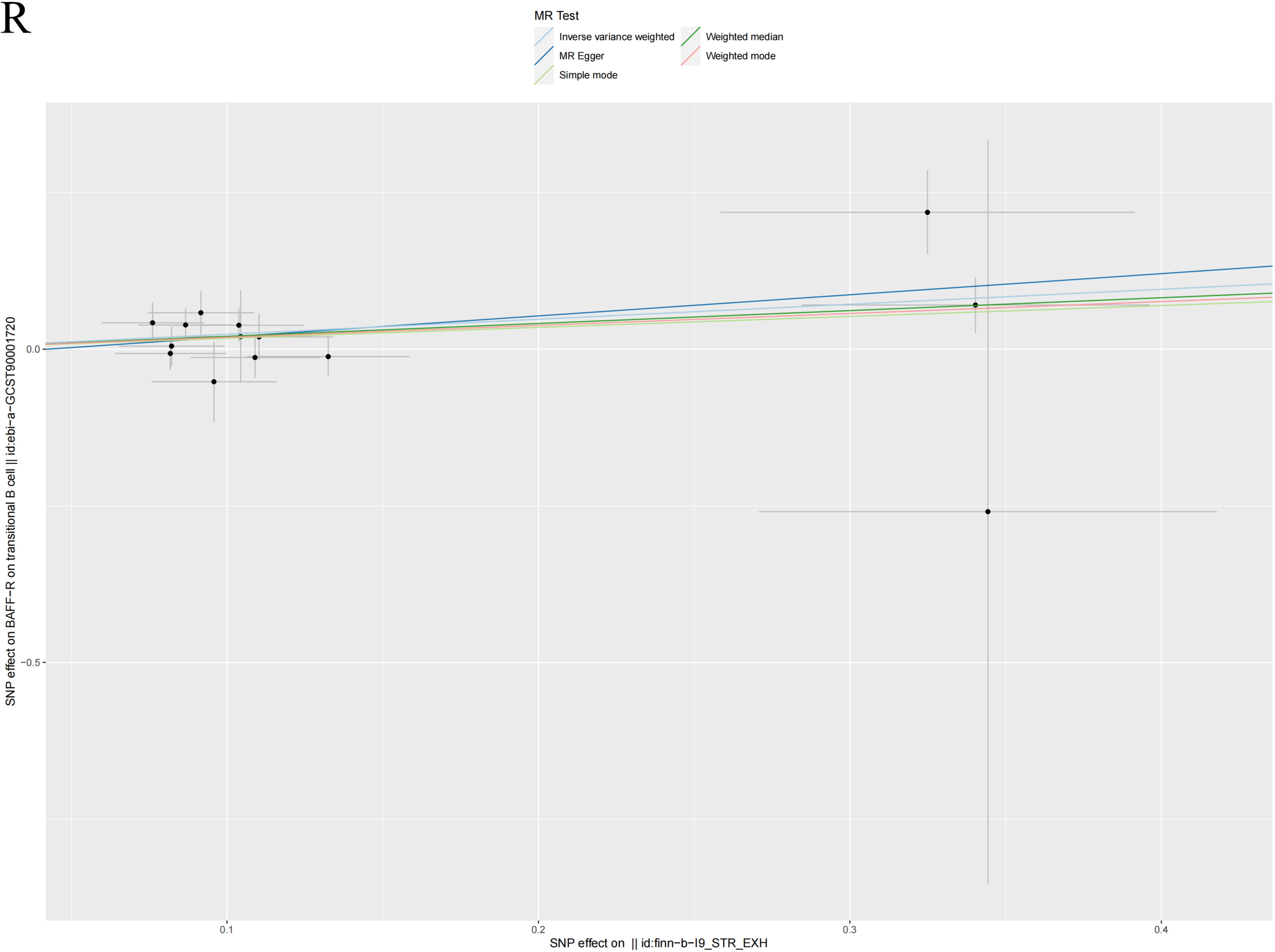

MR Test
Inverse variance weighted
MR Egger
Simple mode
Weighted median
Weighted mode
0.0
−0.5
0.1
0.2
0.3
0.4
SNP effect on BAFF−R on transitional B cell || id:ebi−a−GCST90001720
SNP effect on || id:finn−b−I9_STR_EXH

S

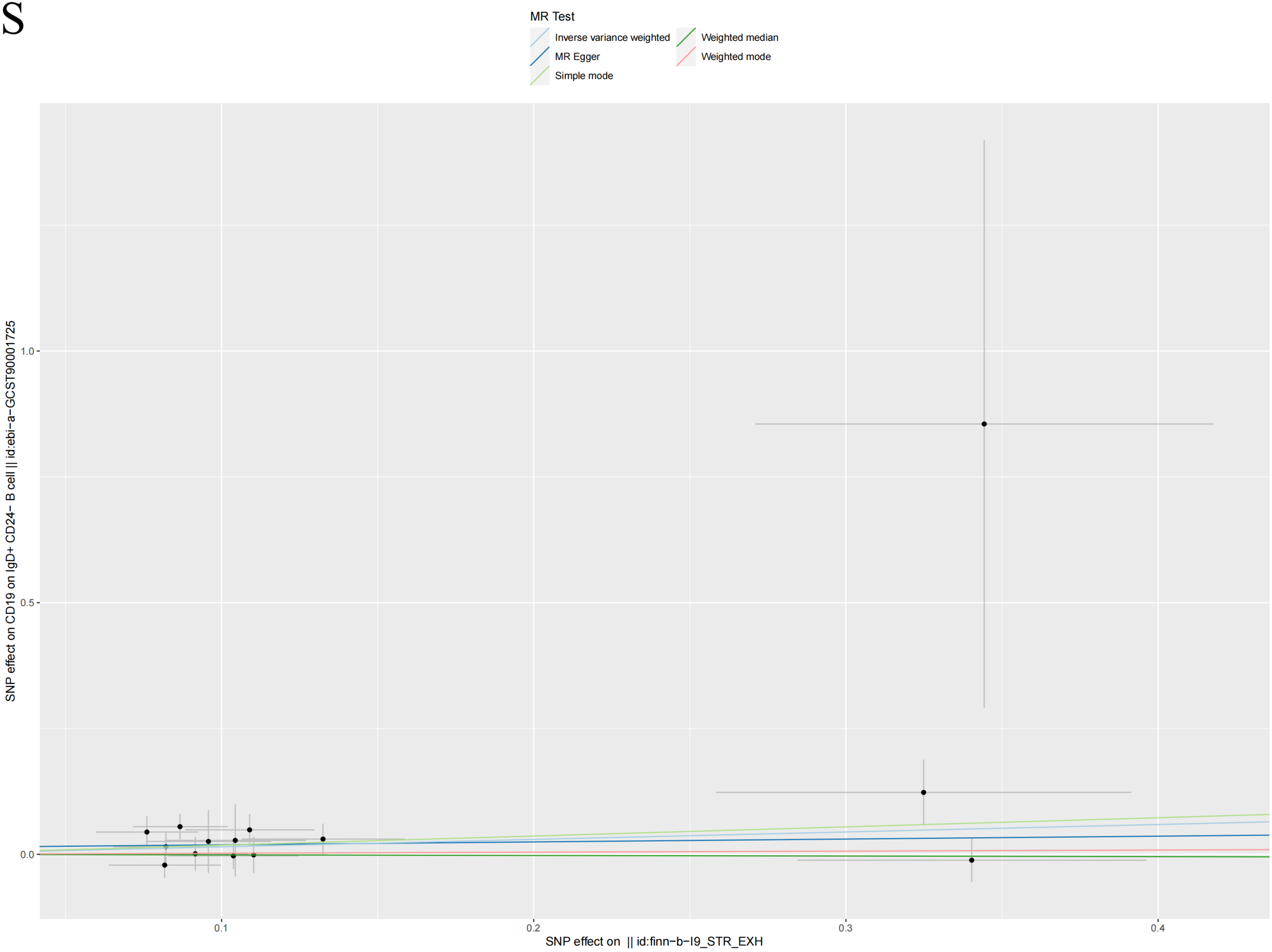

MR Test
Inverse variance weighted
MR Egger
Simple mode
Weighted median
Weighted mode
SNP effect on CD19 on IgD+ CD24− B cell || id:ebi−a−GCST90001725
1.0
0.5
0.0
0.1
0.2
0.3
0.4
SNP effect on || id:finn−b−I9_STR_EXH

T

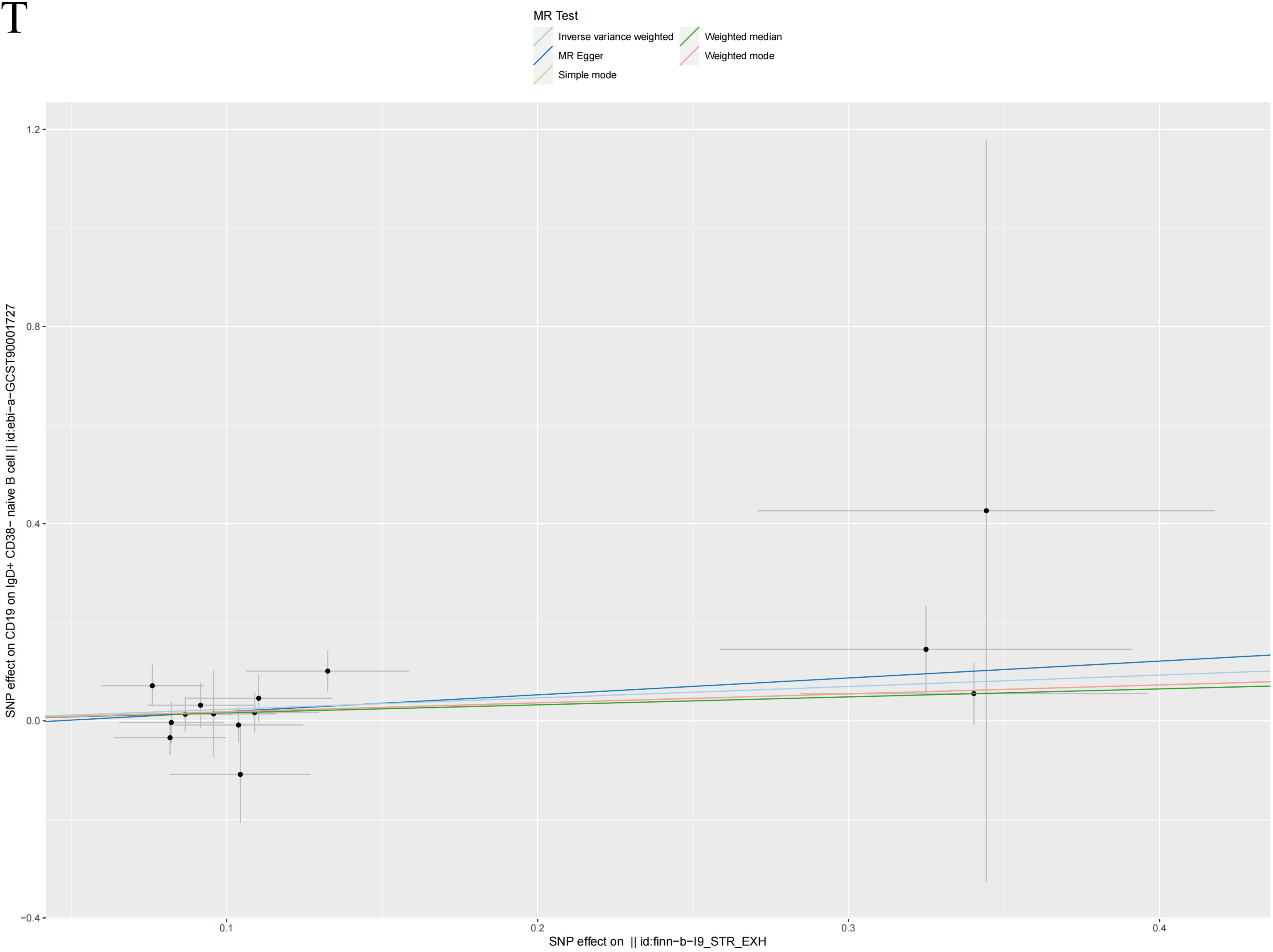

MR Test
Inverse variance weighted
MR Egger
Simple mode
Weighted median
Weighted mode
1.2
0.8
0.4
0.0
−0.4
0.1
0.2
0.3
0.4
SNP effect on CD19 on IgD+ CD38− naive B cell || id:ebi−a−GCST90001727
SNP effect on || id:finn−b−I9_STR_EXH

U

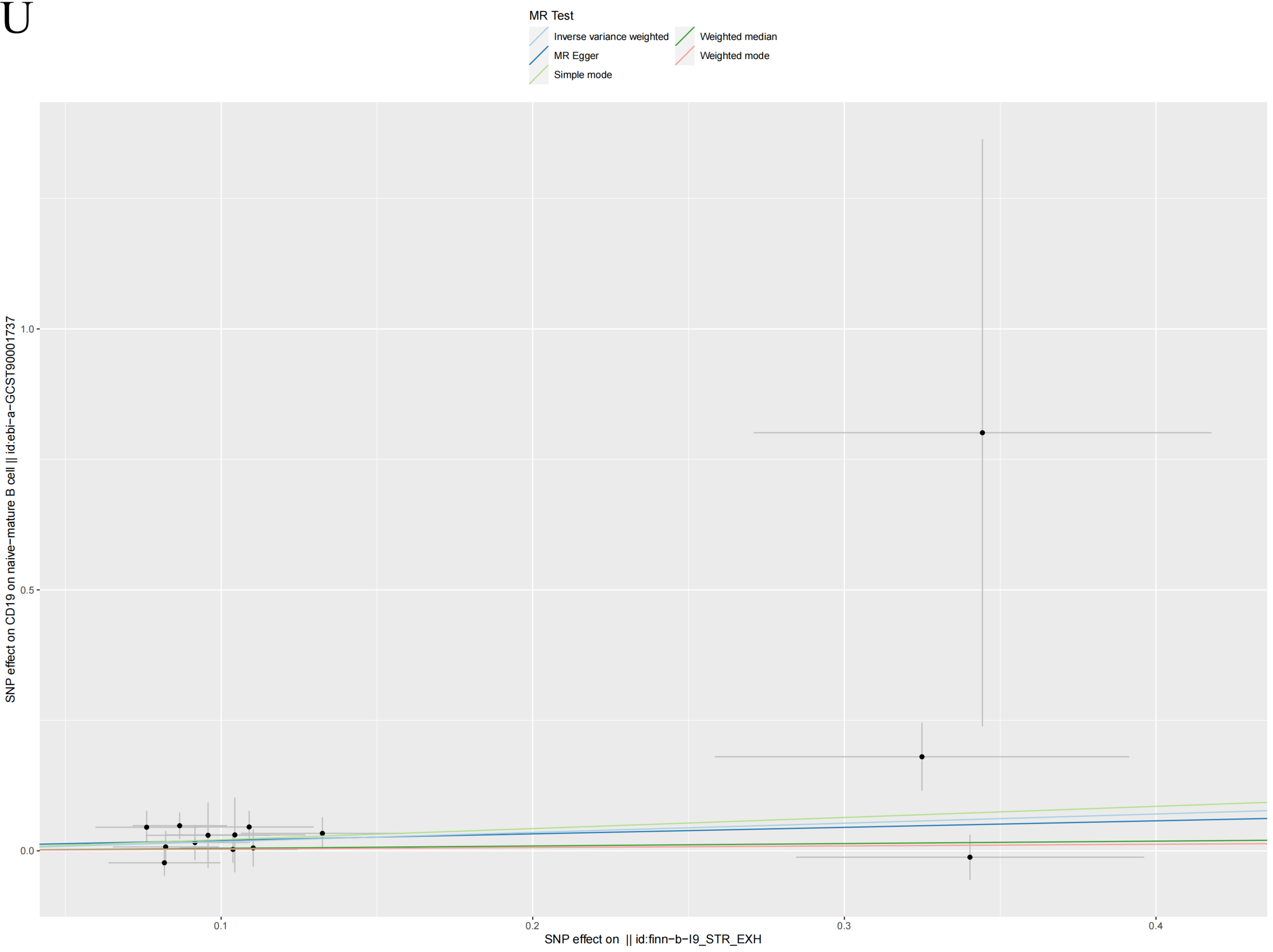

MR Test
Inverse variance weighted
MR Egger
Simple mode
Weighted median
Weighted mode
SNP effect on CD19 on naive−mature B cell || id:ebi−a−GCST90001737
1.0
0.5
0.0
0.1
0.2
0.3
0.4
SNP effect on || id:finn−b−I9_STR_EXH

V

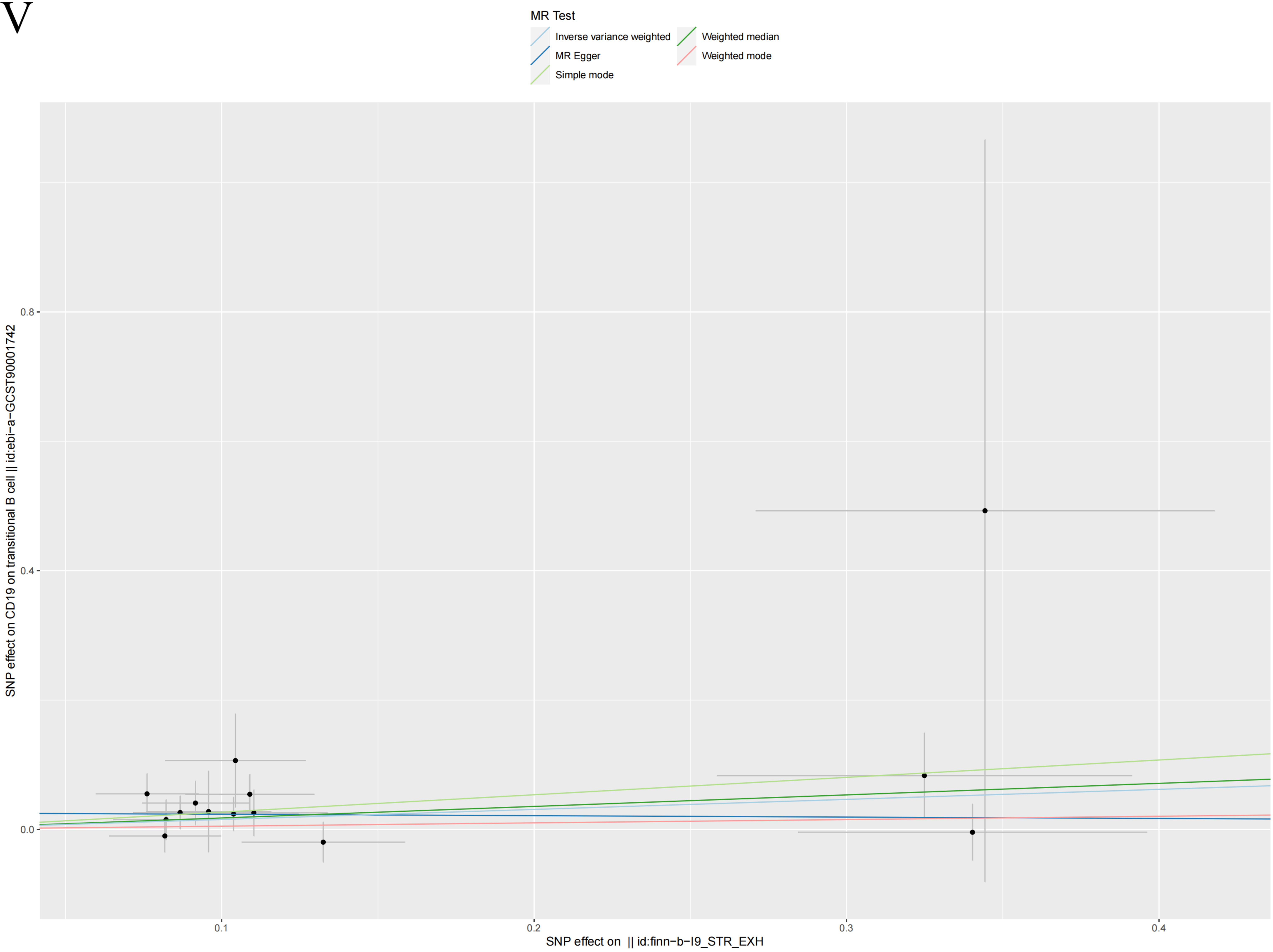

MR Test
Inverse variance weighted
MR Egger
Simple mode
Weighted median
Weighted mode
SNP effect on CD19 on transitional B cell || id:ebi-a-GCST90001742
0.8
0.4
0.0
SNP effect on || id:finn-b-I9_STR_EXH
0.1
0.2
0.3
0.4

W

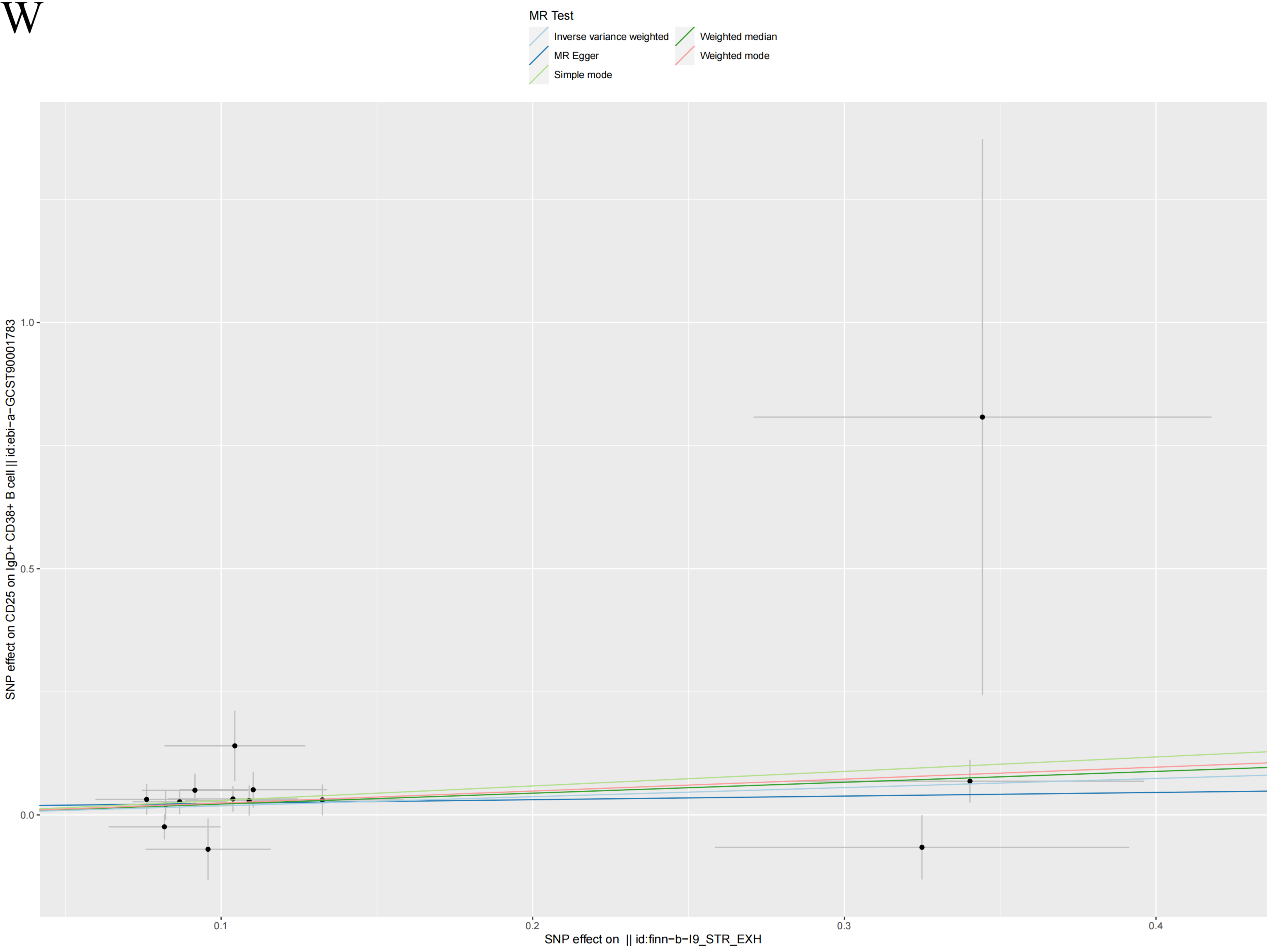

MR Test
Inverse variance weighted
MR Egger
Simple mode
Weighted median
Weighted mode
SNP effect on CD25 on IgD+ CD38+ B cell || id:ebi-a-GCST90001783
1.0
0.5
0.0
0.1
0.2
0.3
0.4
SNP effect on || id:finn-b-I9_STR_EXH

X

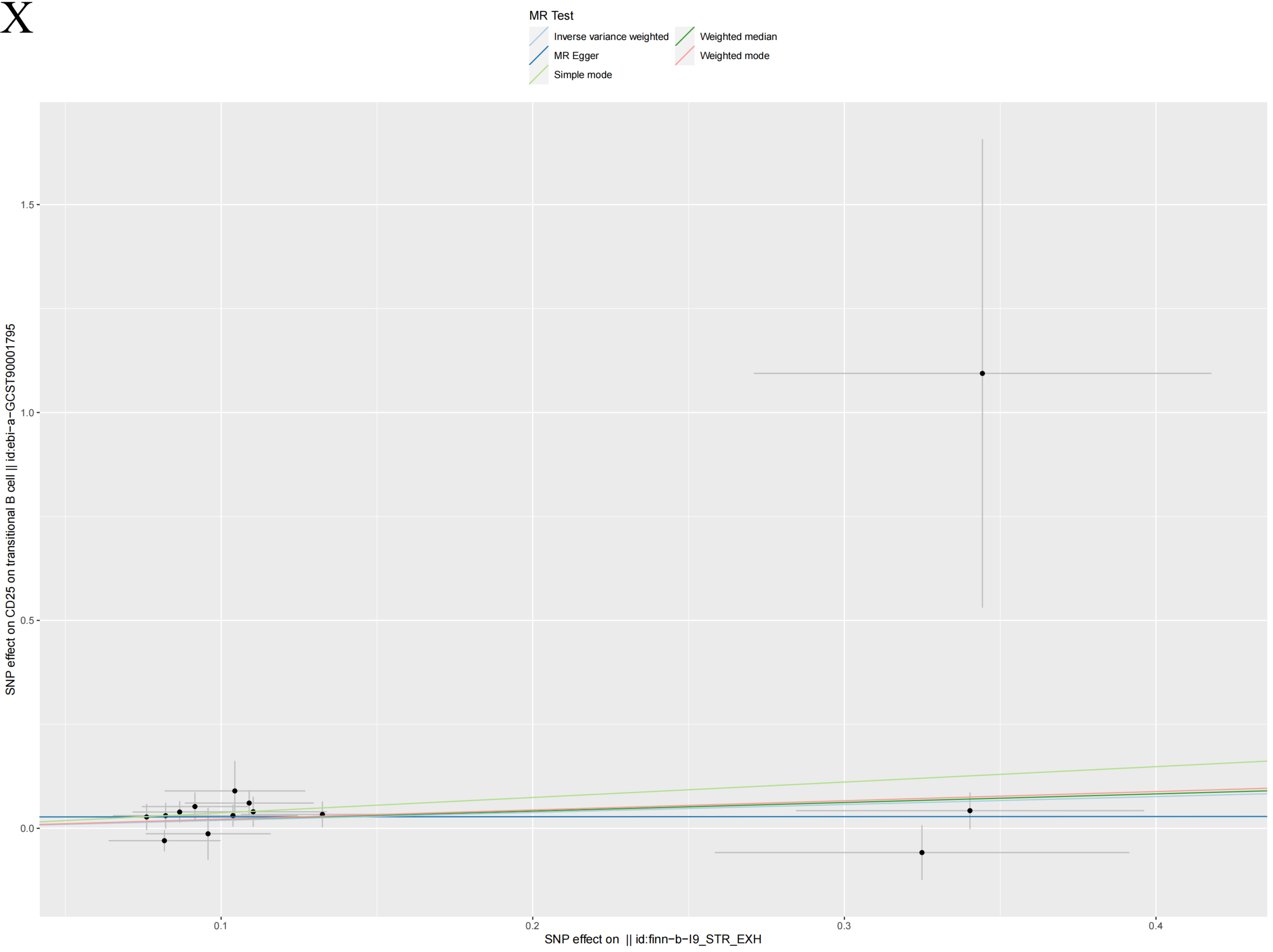
MR Test
Inverse variance weighted
MR Egger
Simple mode
Weighted median
Weighted mode
SNP effect on CD25 on transitional B cell || id:ebi−a−GCST90001795
1.5
1.0
0.5
0.0
0.1
0.2
0.3
0.4
SNP effect on || id:finn−b−I9_STR_EXH

Y

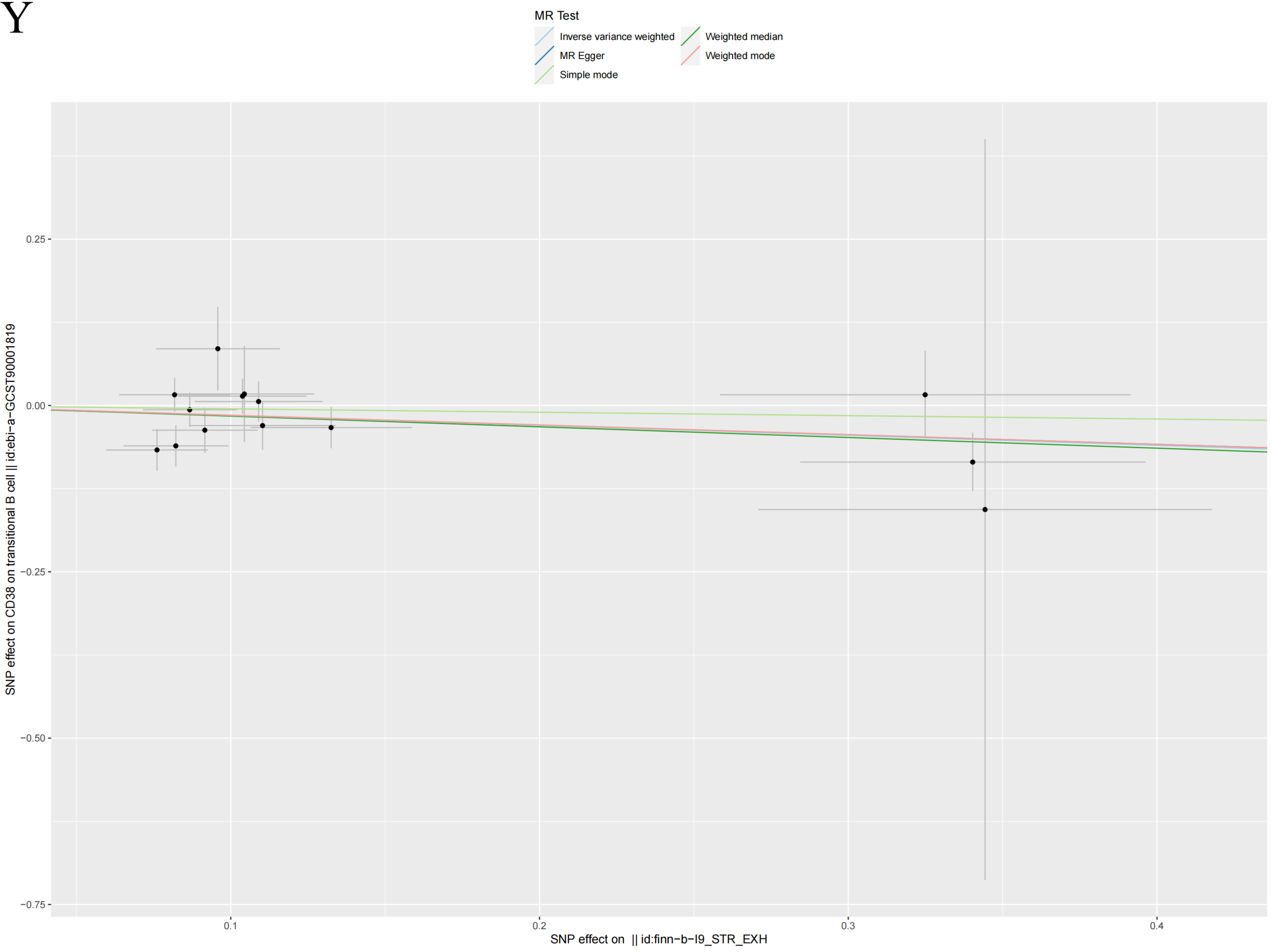

MR Test
Inverse variance weighted
MR Egger
Simple mode
Weighted median
Weighted mode
0.25
0.00
−0.25
−0.50
−0.75
0.1
0.2
0.3
0.4
SNP effect on CD38 on transitional B cell || id:ebi−a−GCST90001819
SNP effect on || id:finn−b−I9_STR_EXH

Z

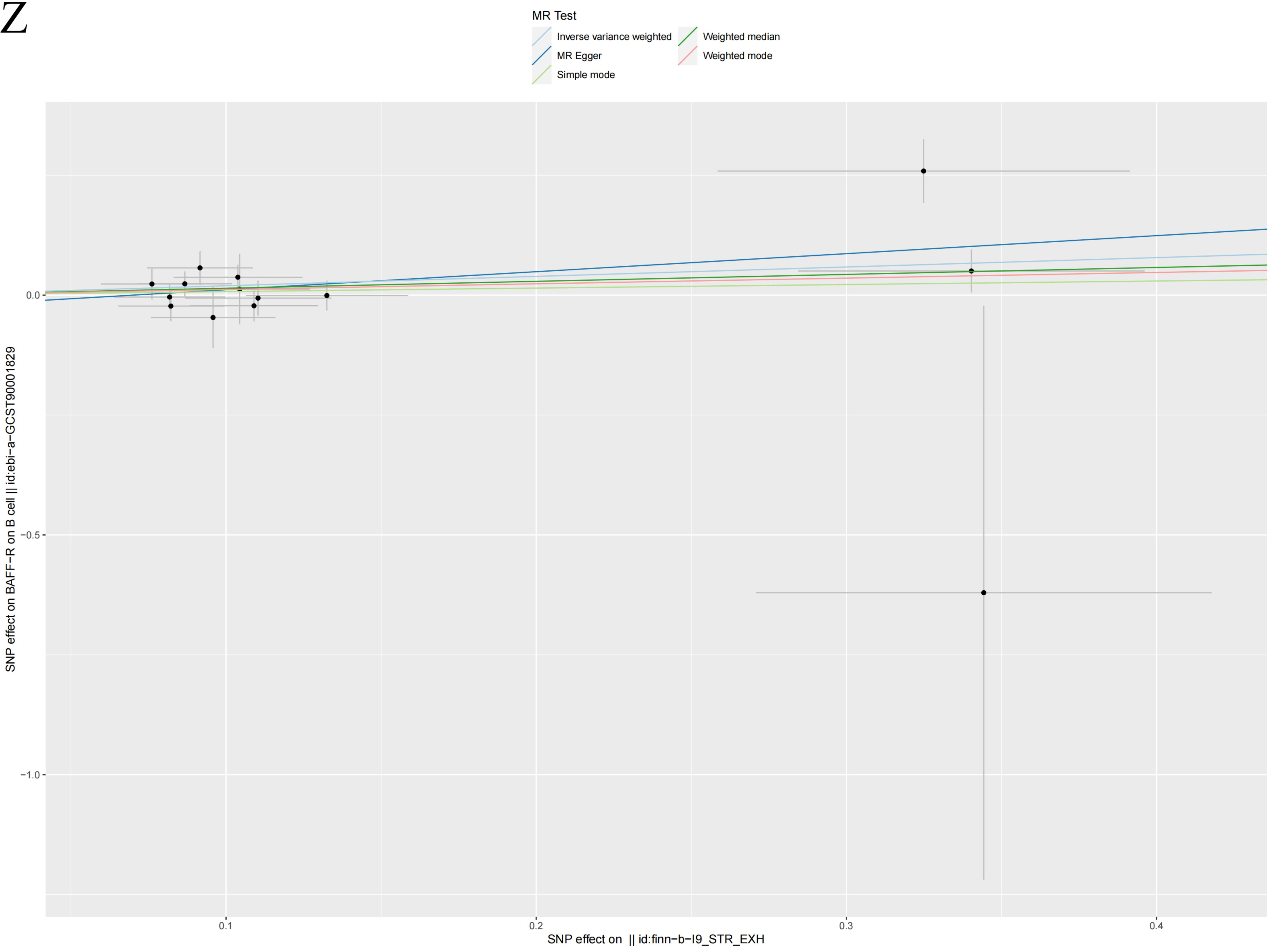
MR Test
Inverse variance weighted
MR Egger
Simple mode
Weighted median
Weighted mode
0.0
−0.5
−1.0
0.1
0.2
0.3
0.4
SNP effect on BAFF−R on B cell || id:ebi−a−GCST90001829
SNP effect on || id:finn−b−I9_STR_EXH

AA

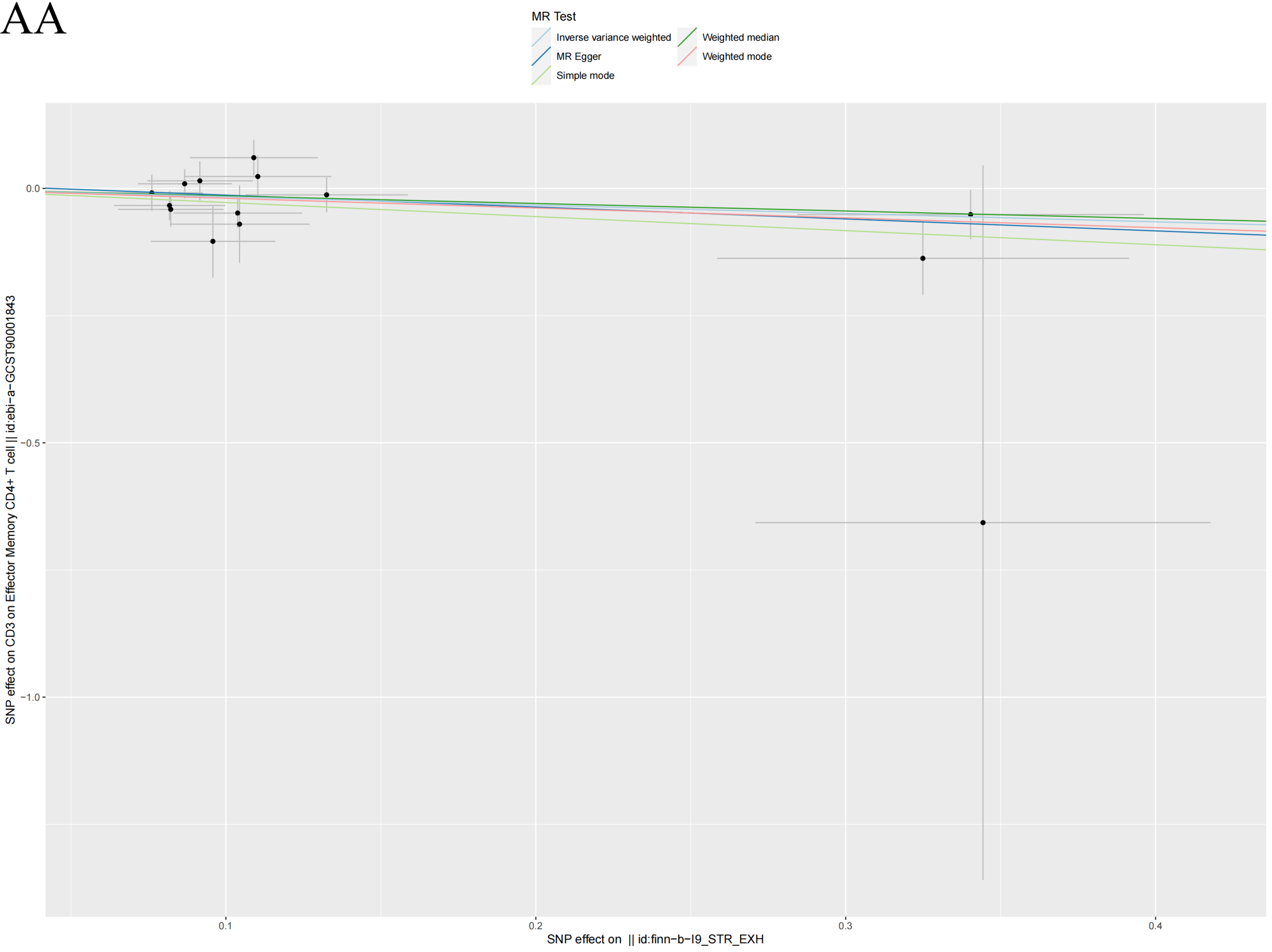

MR Test
Inverse variance weighted
MR Egger
Simple mode
Weighted median
Weighted mode
0.0
−0.5
−1.0
0.1
0.2
0.3
0.4
SNP effect on CD3 on Effector Memory CD4+ T cell || id:ebi−a−GCST90001843
SNP effect on || id:finn−b−I9_STR_EXH

AB

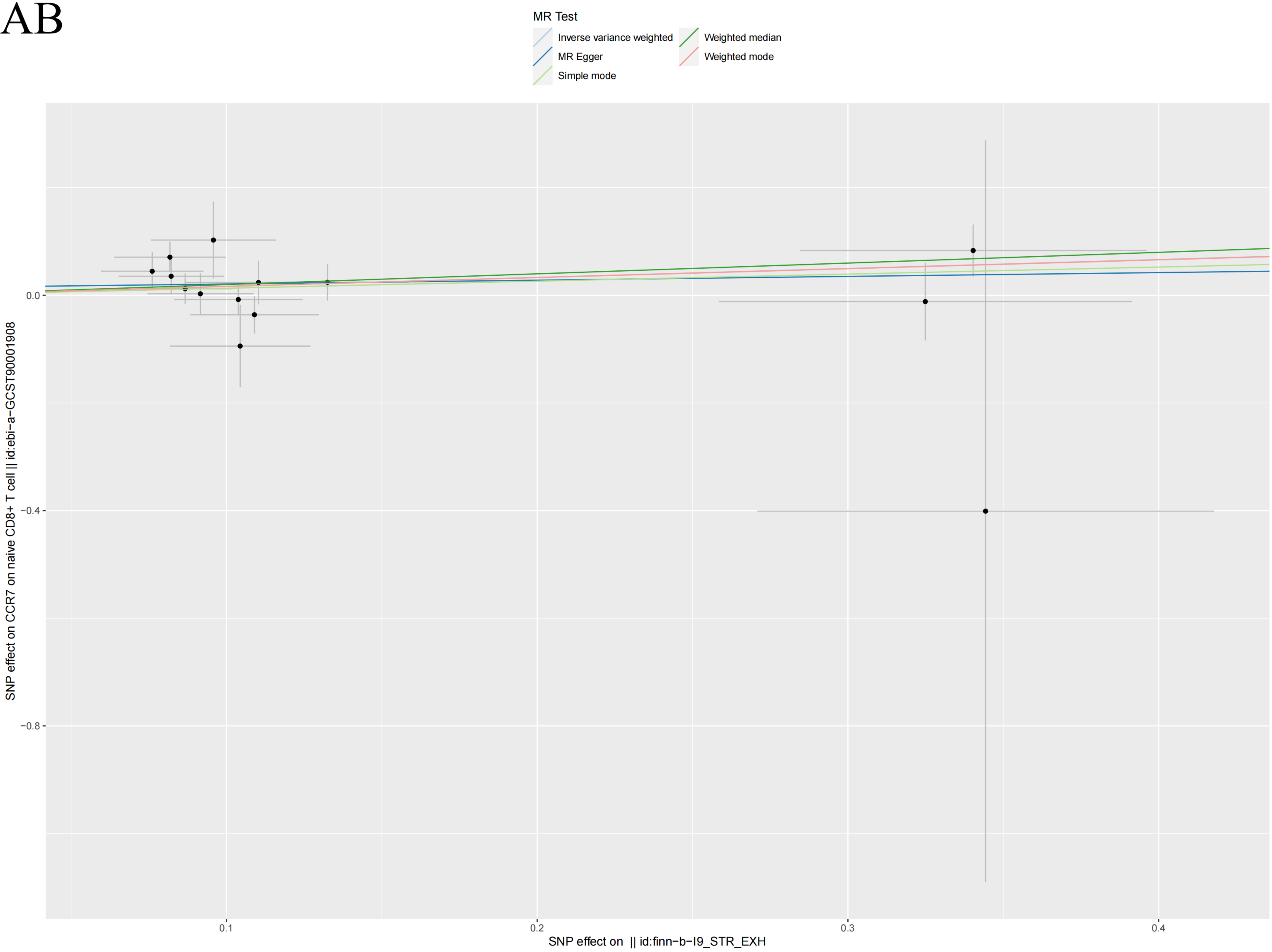

MR Test
Inverse variance weighted
MR Egger
Simple mode
Weighted median
Weighted mode
0.0
−0.4
−0.8
0.1
0.2
0.3
0.4
SNP effect on CCR7 on naive CD8+ T cell || id:ebi−a−GCST90001908
SNP effect on || id:finn−b−I9_STR_EXH

AC

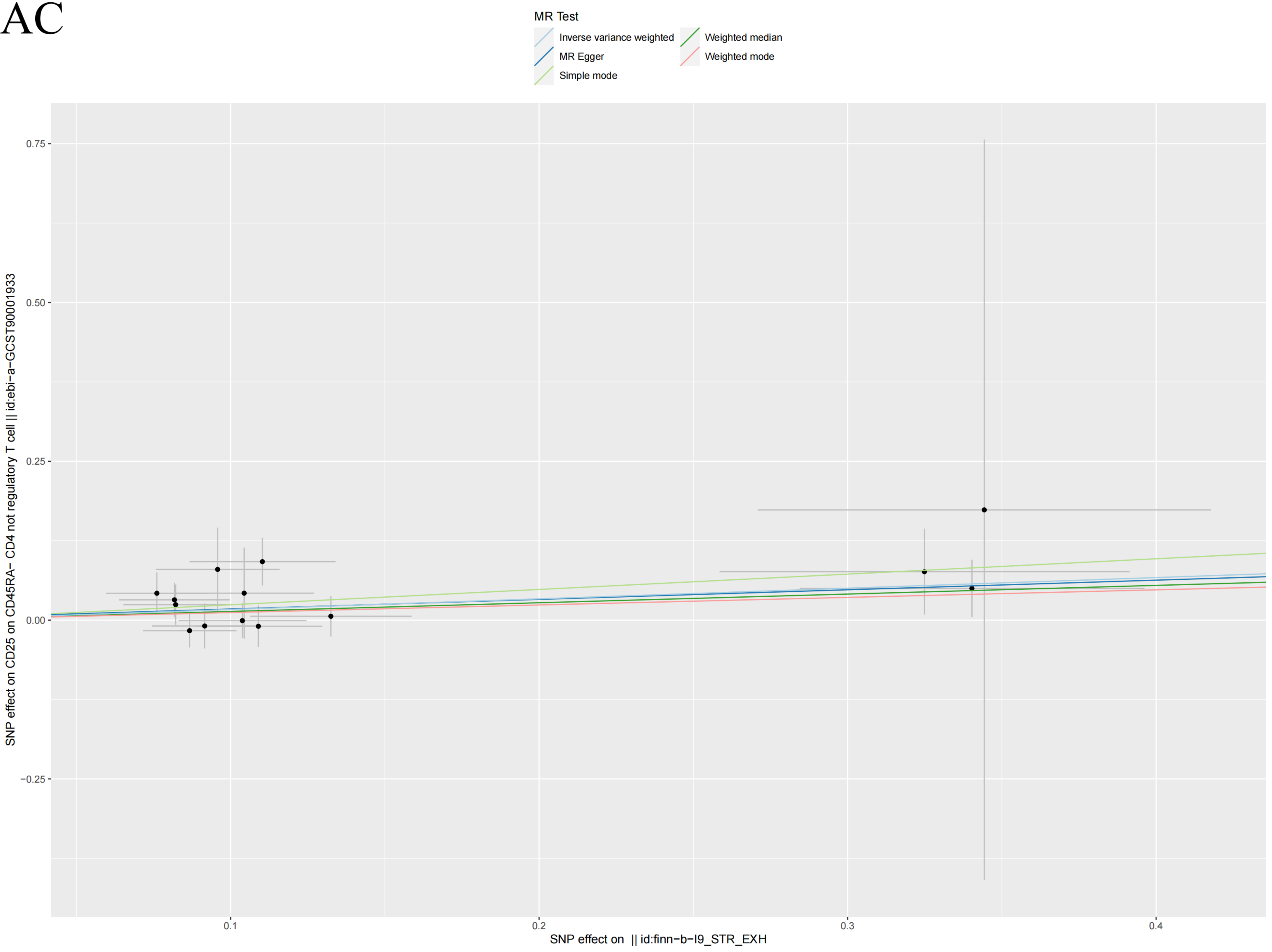
MR Test
Inverse variance weighted
MR Egger
Simple mode
Weighted median
Weighted mode
0.75
0.50
0.25
0.00
−0.25
SNP effect on CD25 on CD45RA− CD4 not regulatory T cell || id:ebi−a−GCST90001933
0.1
0.2
0.3
0.4
SNP effect on || id:finn−b−I9_STR_EXH

AD

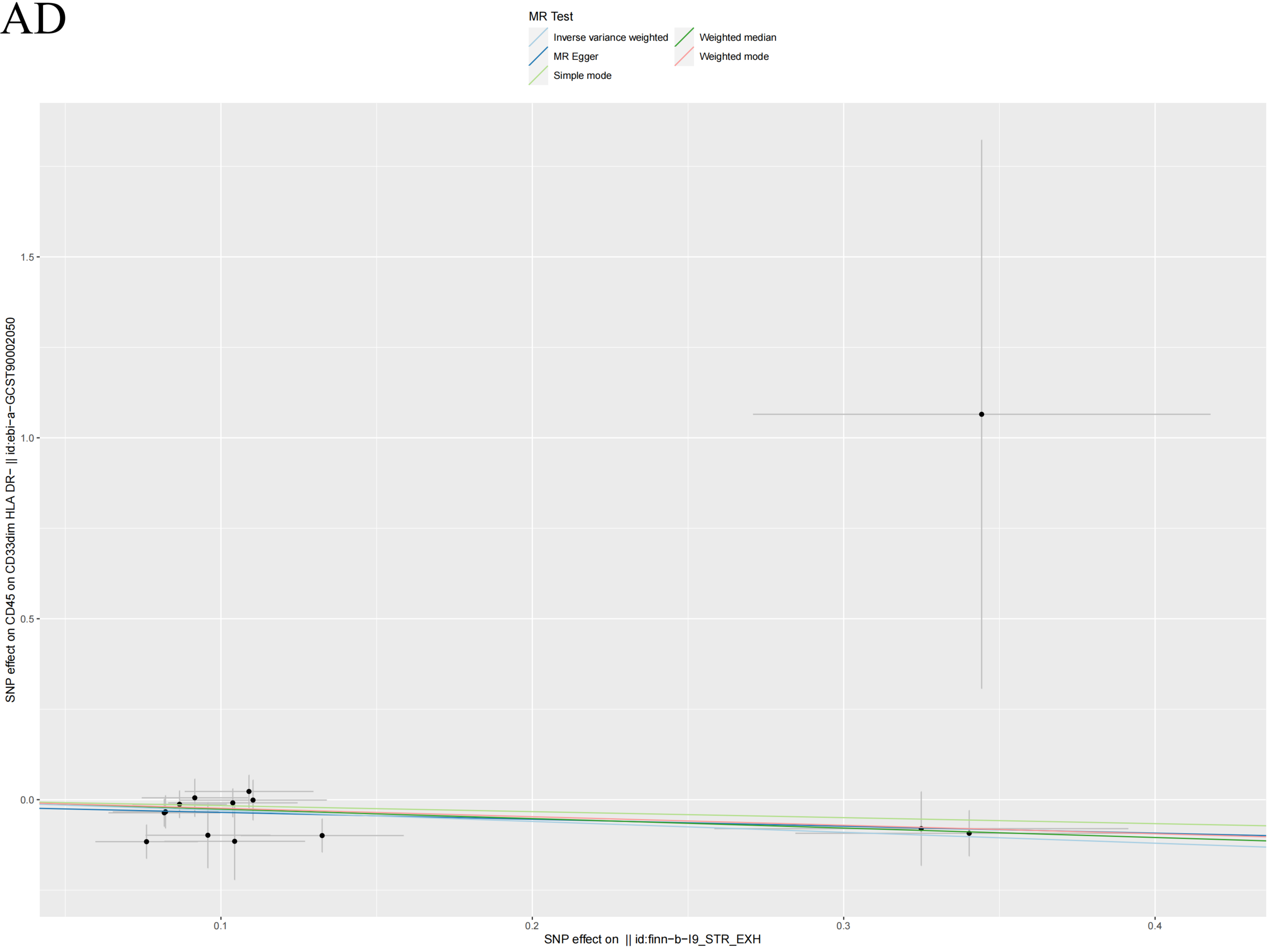
MR Test
Inverse variance weighted
MR Egger
Simple mode
Weighted median
Weighted mode
1.5
1.0
0.5
0.0
0.1
0.2
0.3
0.4
SNP effect on CD45 on CD33dim HLA DR− || id:ebi−a−GCST90002050
SNP effect on || id:finn−b−I9_STR_EXH

AE

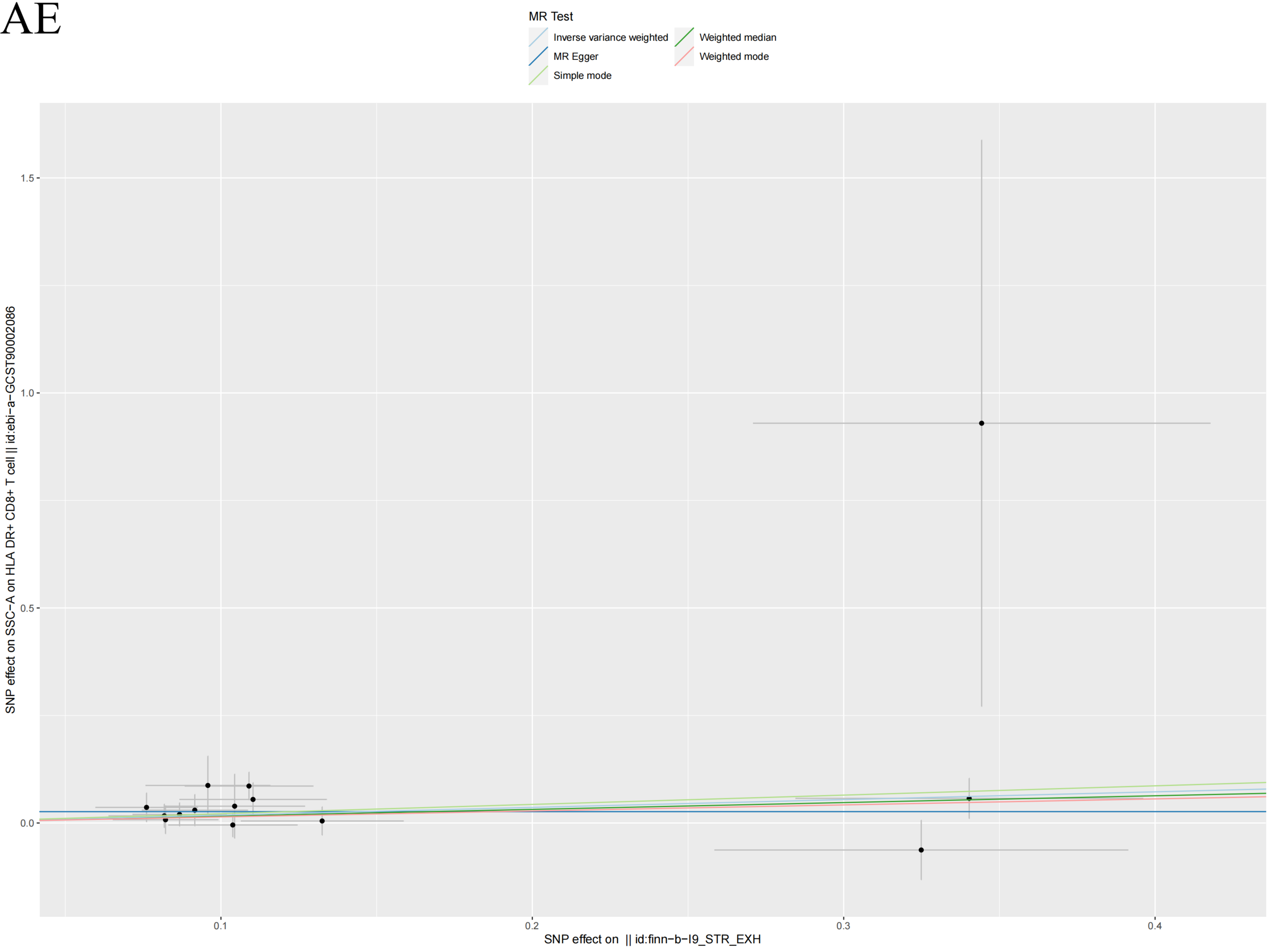

MR Test
Inverse variance weighted
MR Egger
Simple mode
Weighted median
Weighted mode
1.5
1.0
0.5
0.0
SNP effect on SSC−A on HLA DR+ CD8+ T cell || id:ebi−a−GCST90002086
0.1
0.2
0.3
0.4
SNP effect on || id:finn−b−I9_STR_EXH

AF

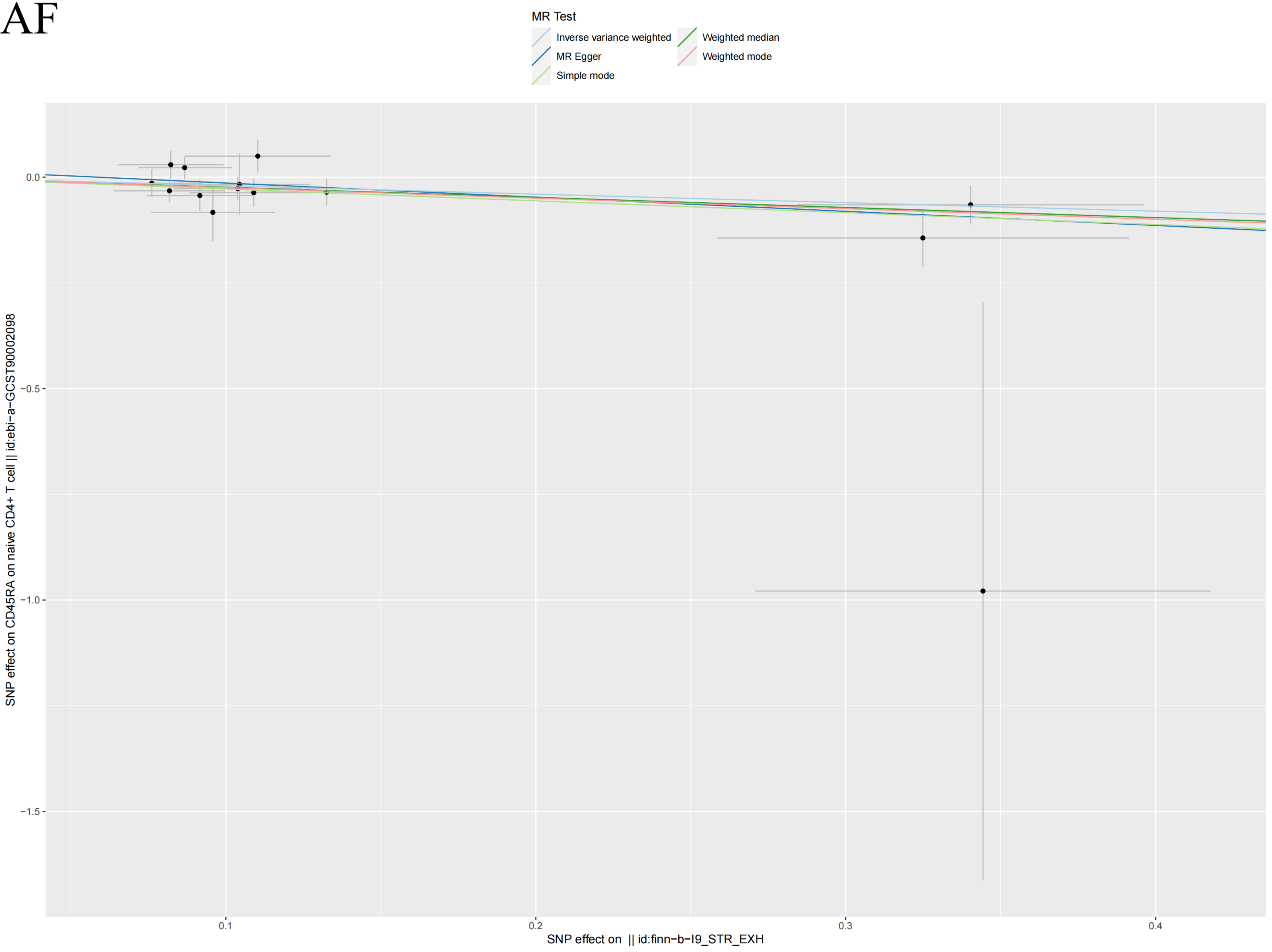

MR Test
Inverse variance weighted
MR Egger
Simple mode
Weighted median
Weighted mode
0.0
−0.5
−1.0
−1.5
0.1
0.2
0.3
0.4
SNP effect on || id:finn−b−I9_STR_EXH
SNP effect on CD45RA on naive CD4+ T cell || id:ebi−a−GCST90002098

AG

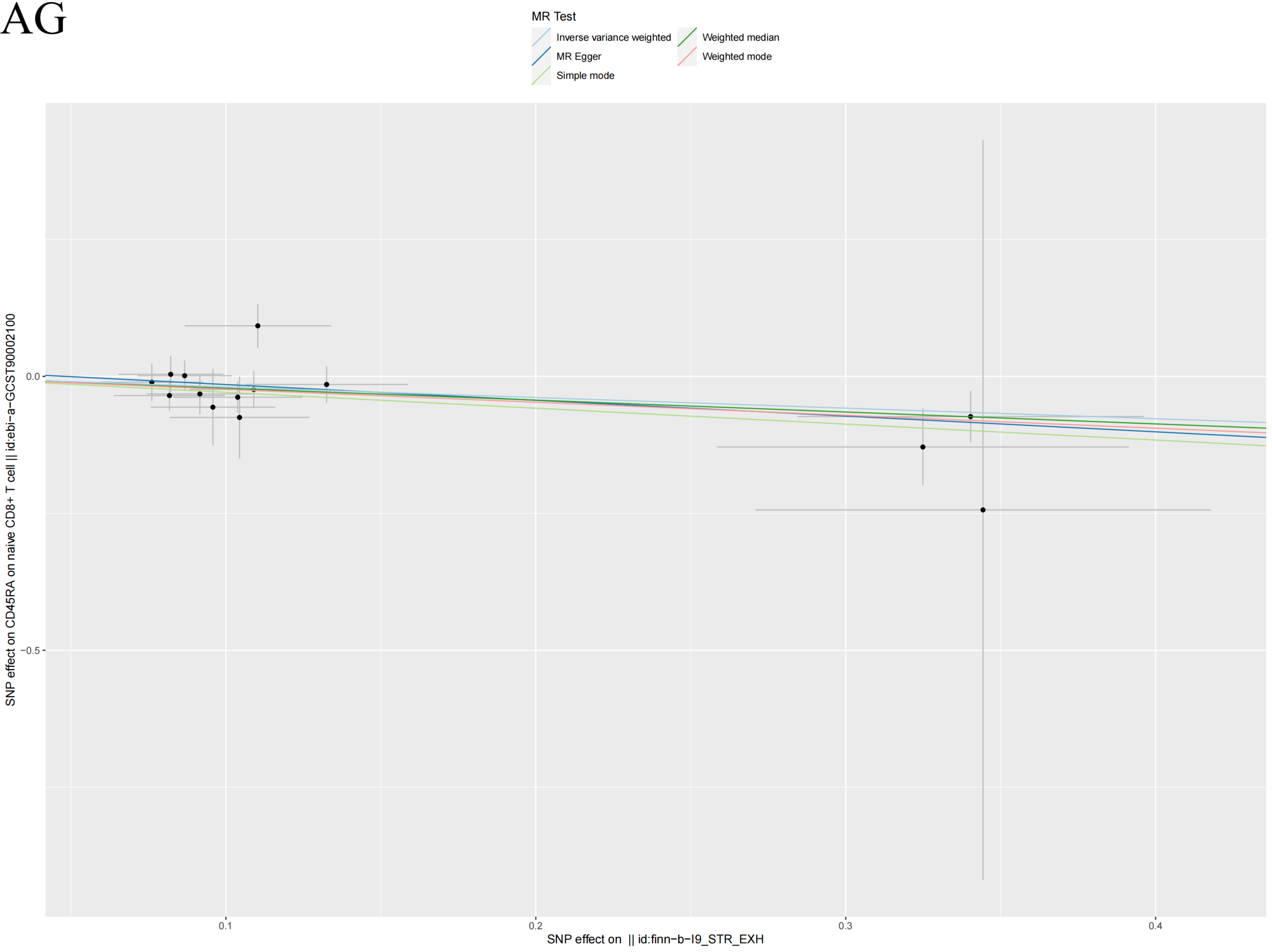

MR Test
Inverse variance weighted
MR Egger
Simple mode
Weighted median
Weighted mode
0.0
−0.5
0.1
0.2
0.3
0.4
SNP effect on CD45RA on naive CD8+ T cell || id:ebi−a−GCST90002100
SNP effect on || id:finn−b−I9_STR_EXH

AH

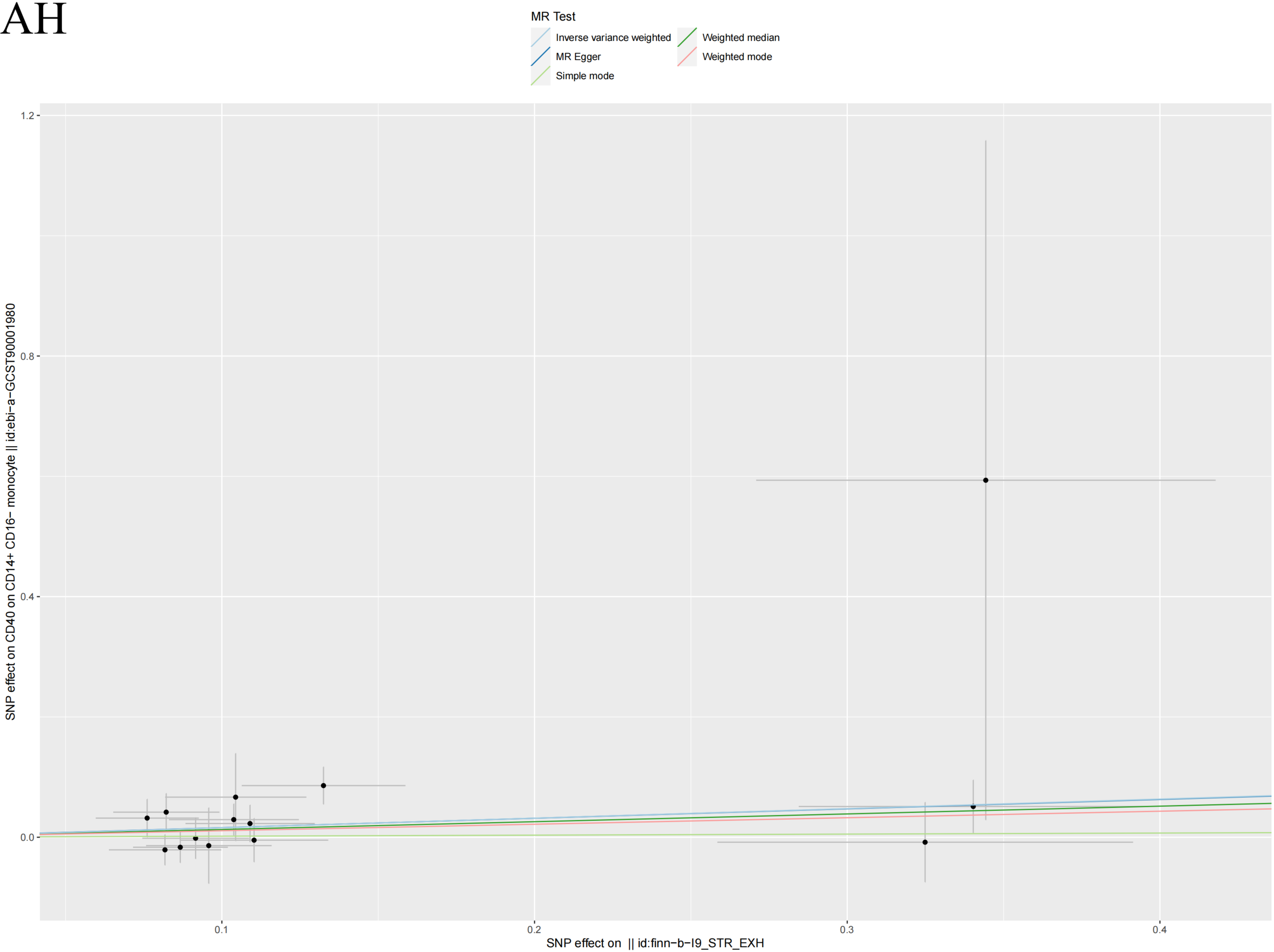
MR Test
Inverse variance weighted
MR Egger
Simple mode
Weighted median
Weighted mode
1.2
0.8
0.4
0.0
0.1
0.2
0.3
0.4
SNP effect on CD40 on CD14+ CD16− monocyte || id:ebi−a−GCST90001980
SNP effect on || id:finn−b−I9_STR_EXH

AI

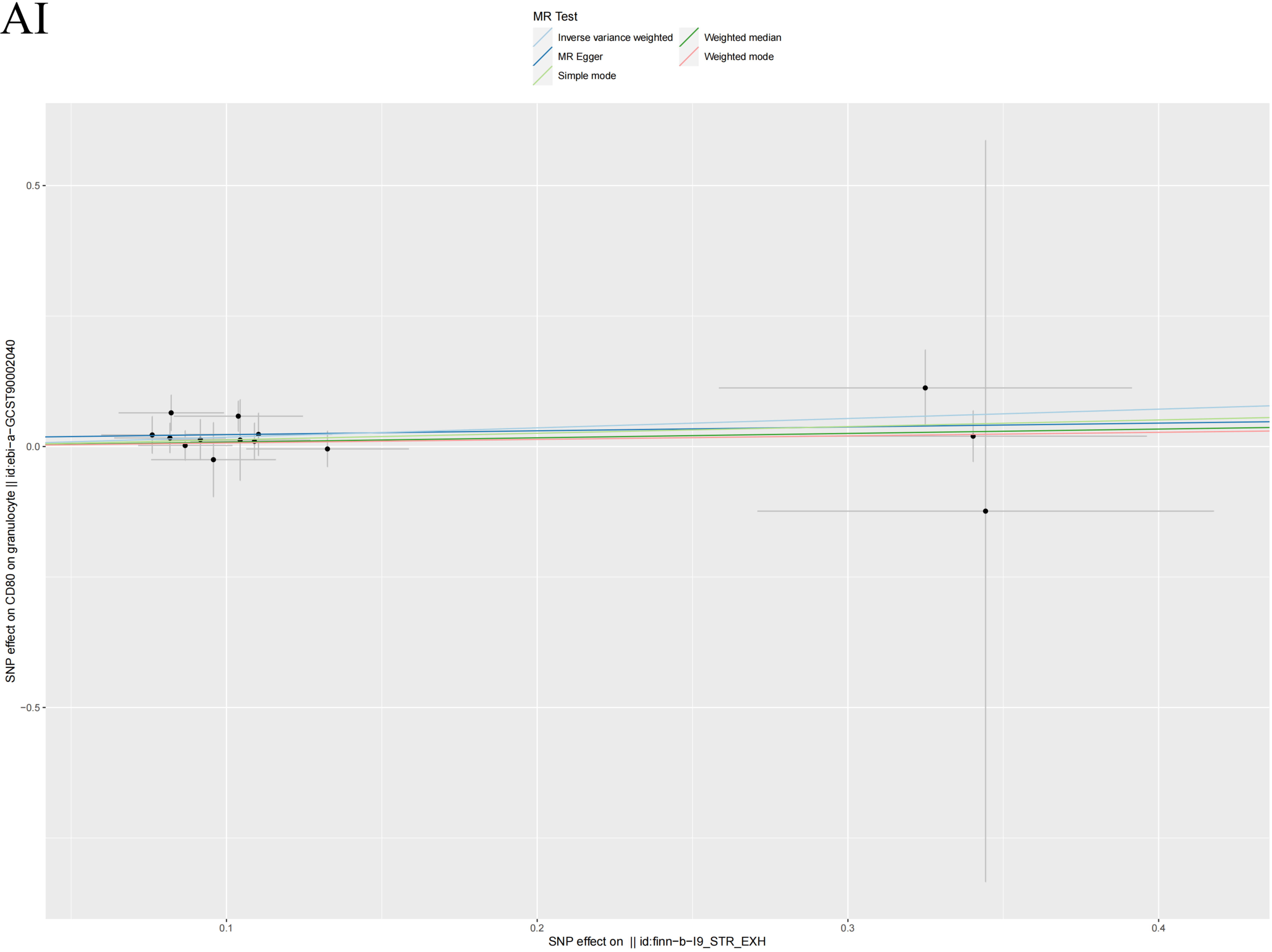
MR Test
Inverse variance weighted
MR Egger
Simple mode
Weighted median
Weighted mode
SNP effect on CD80 on granulocyte || id:ebi-a-GCST90002040
0.5
0.0
−0.5
SNP effect on || id:finn-b-I9_STR_EXH
0.1
0.2
0.3
0.4

AJ

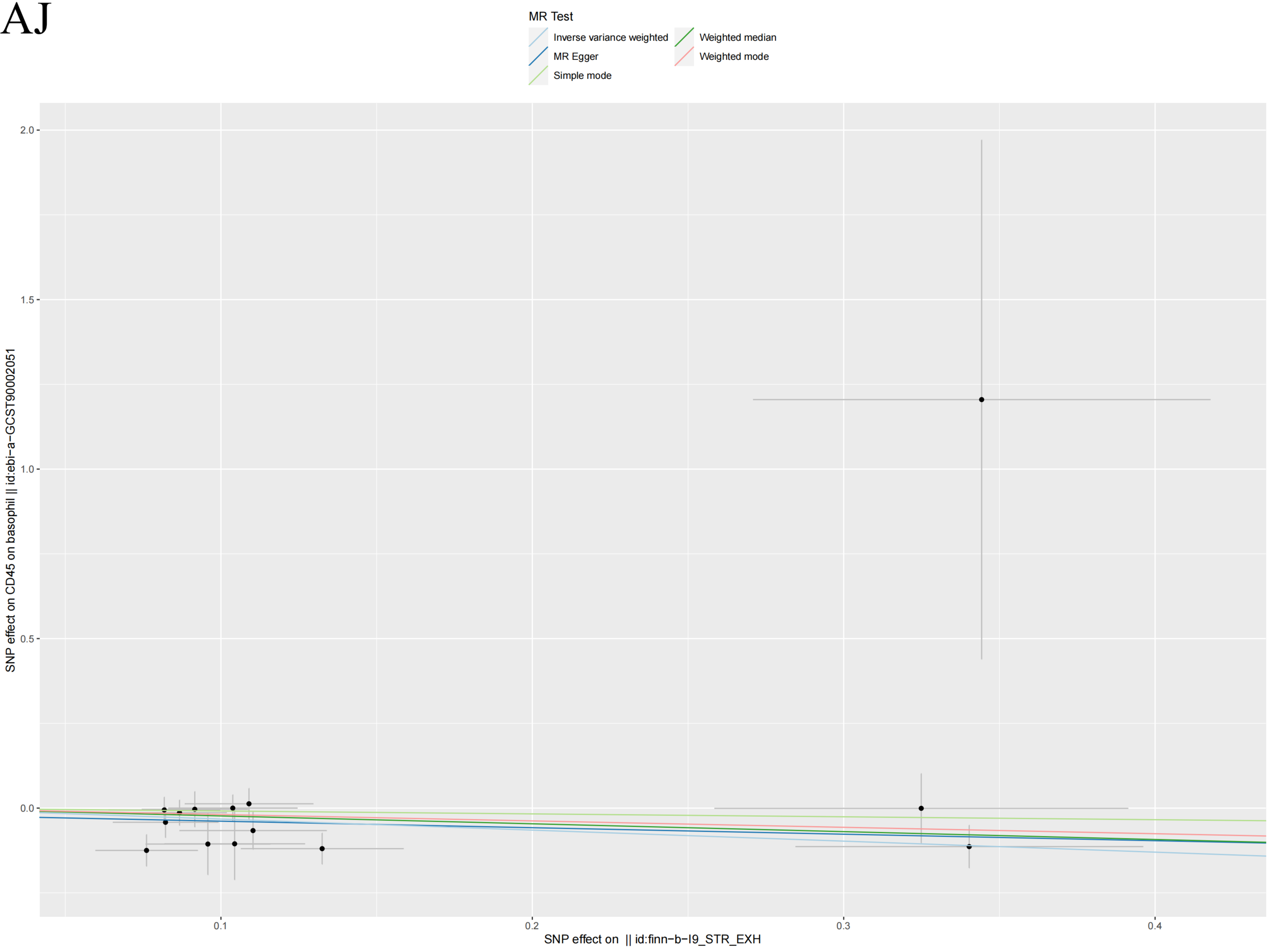

MR Test
Inverse variance weighted
MR Egger
Simple mode
Weighted median
Weighted mode
2.0
1.5
1.0
0.5
0.0
0.1
0.2
0.3
0.4
SNP effect on CD45 on basophil || id:ebi-a-GCST90002051
SNP effect on || id:finn-b-I9_STR_EXH

AK

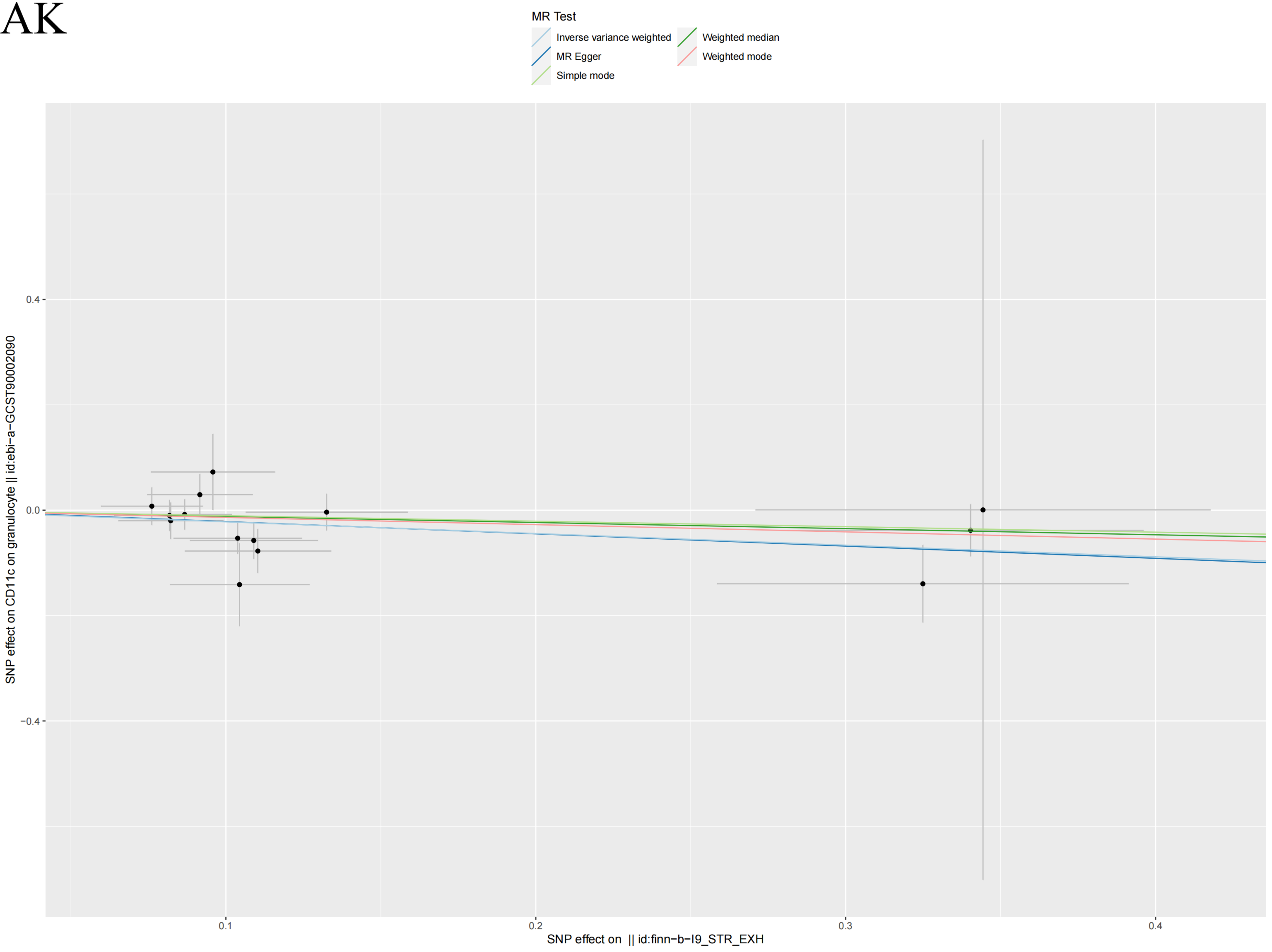

MR Test
Inverse variance weighted
MR Egger
Simple mode
Weighted median
Weighted mode
0.4
0.0
−0.4
0.1
0.2
0.3
0.4
SNP effect on CD11c on granulocyte || id:ebi−a−GCST90002090
SNP effect on || id:finn−b−I9_STR_EXH

Supplementary Figure 3. Sensitivity analysis of reverse Mendelian randomization (Scatter plot).

A. Scatter plot between IS and BAFF-R on CD24+ CD27+ B cell; B. Scatter plot between IS and BAFF-R on IgD+ CD24+ B cell; C. Scatter plot between IS and BAFF-R on IgD+ CD24- B cell; D. Scatter plot between IS and BAFF-R on IgD+ CD38- B cell; E. Scatter plot between IS and BAFF-R on IgD+ CD38- naive B cell; F. Scatter plot between IS and BAFF-R on IgD+ CD38- unswitched memory B cell; G. Scatter plot between IS and BAFF-R on IgD+ CD38+ B cell; H. Scatter plot between IS and BAFF-R on IgD+ CD38dim B cell; I. Scatter plot between IS and BAFF-R on IgD- CD24- B cell; J. Scatter plot between IS and BAFF-R on IgD- CD27- B cell; K. Scatter plot between IS and BAFF-R on IgD- CD38- B cell; L. Scatter plot between IS and BAFF-R on IgD- CD38dim B cell; M. Scatter plot between IS and BAFF-R on memory B cell; N. Scatter plot between IS and BAFF-R on naive-mature B cell; O. Scatter plot between IS and BAFF-R on unswitched memory B cell; P. Scatter plot between IS and BAFF-R on switched memory B cell; Q. Scatter plot between IS and BAFF-R on IgD+ B cell; R. Scatter plot between IS and BAFF-R on transitional B cell; S. Scatter plot between IS and CD19 on IgD+ CD24- B cell; T. Scatter plot between IS and CD19 on IgD+ CD38- naive B cell; U. Scatter plot between IS and CD19 on naive-mature B cell; V. Scatter plot between IS and CD19 on transitional B cell; W. Scatter plot between IS and CD25 on IgD+ CD38+ B cell; X. Scatter plot between IS and CD25 on transitional B cell; Y. Scatter plot between IS and CD38 on transitional B cell; Z. Scatter plot between IS and BAFF-R on B cell; AA. Scatter plot between IS and CD3 on Effector Memory CD4+ T cell; AB. Scatter plot between IS and CCR7 on naive CD8+ T cell; AC. Scatter plot between IS and CD25 on CD45RA- CD4 not regulatory T cell; AD. Scatter plot between IS and CD45 on CD33dim HLA DR-; AE. Scatter plot between IS and SSC-A on HLA DR+ CD8+ T cell; AF. Scatter plot between IS and CD45RA on naive CD4+ T cell; AG. Scatter plot between IS and CD45RA on naive CD8+ T cell; AH. Scatter plot between IS and CD40 on CD14+ CD16- monocyte; AI. Scatter plot between IS and CD80 on granulocyte; AJ. Scatter plot between IS and CD45 on basophil; AK. Scatter plot between IS and CD11c on granulocyte.

A

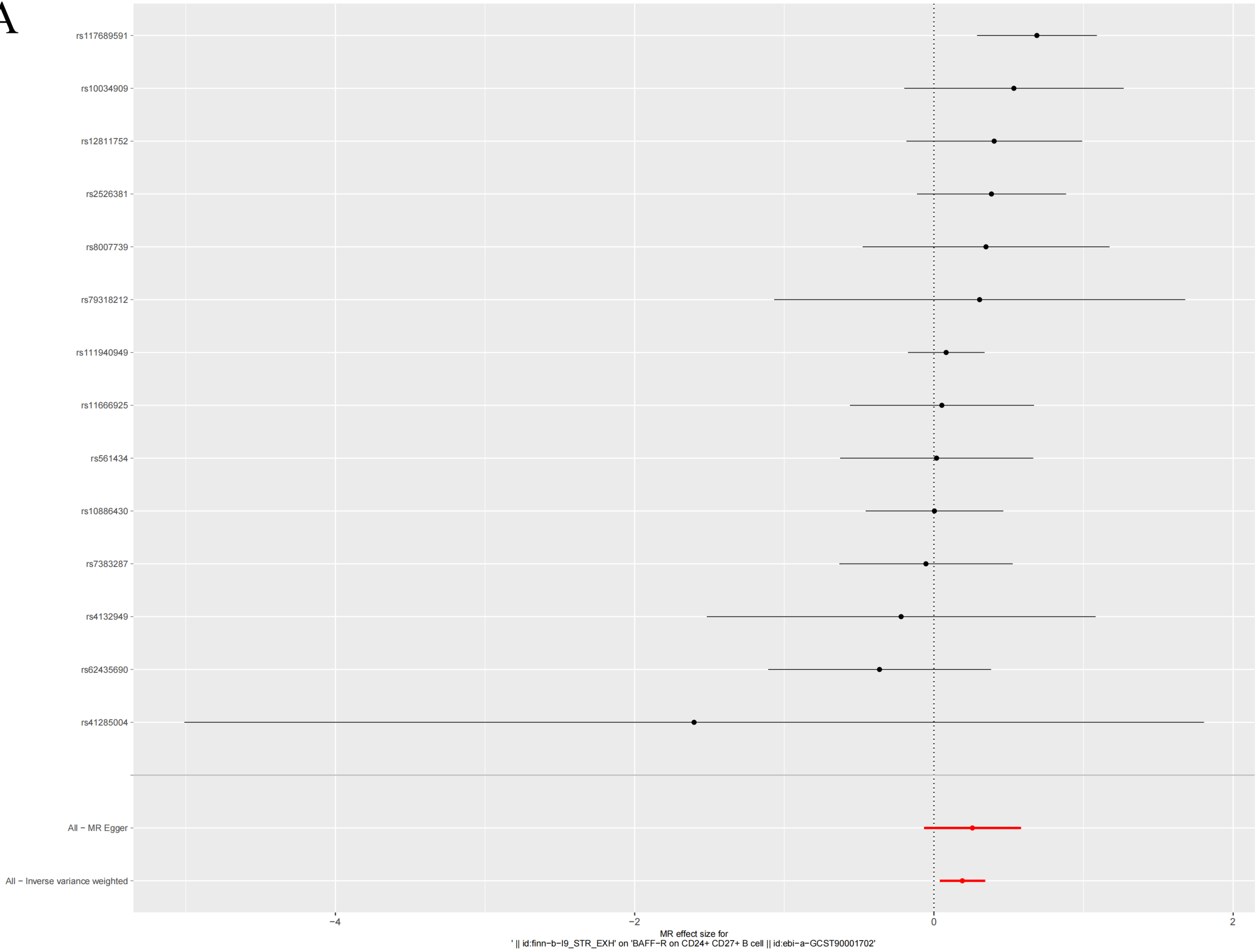

rs117689591
rs10034909
rs12811752
rs2526381
rs8007739
rs79318212
rs111940949
rs11666925
rs561434
rs10886430
rs7383287
rs4132949
rs62435690
rs41285004
All − MR Egger
All − Inverse variance weighted
−4
−2
0
2
MR effect size for
' || id:finn−b−I9_STR_EXH' on 'BAFF−R on CD24+ CD27+ B cell || id:ebi−a−GCST90001702'

B

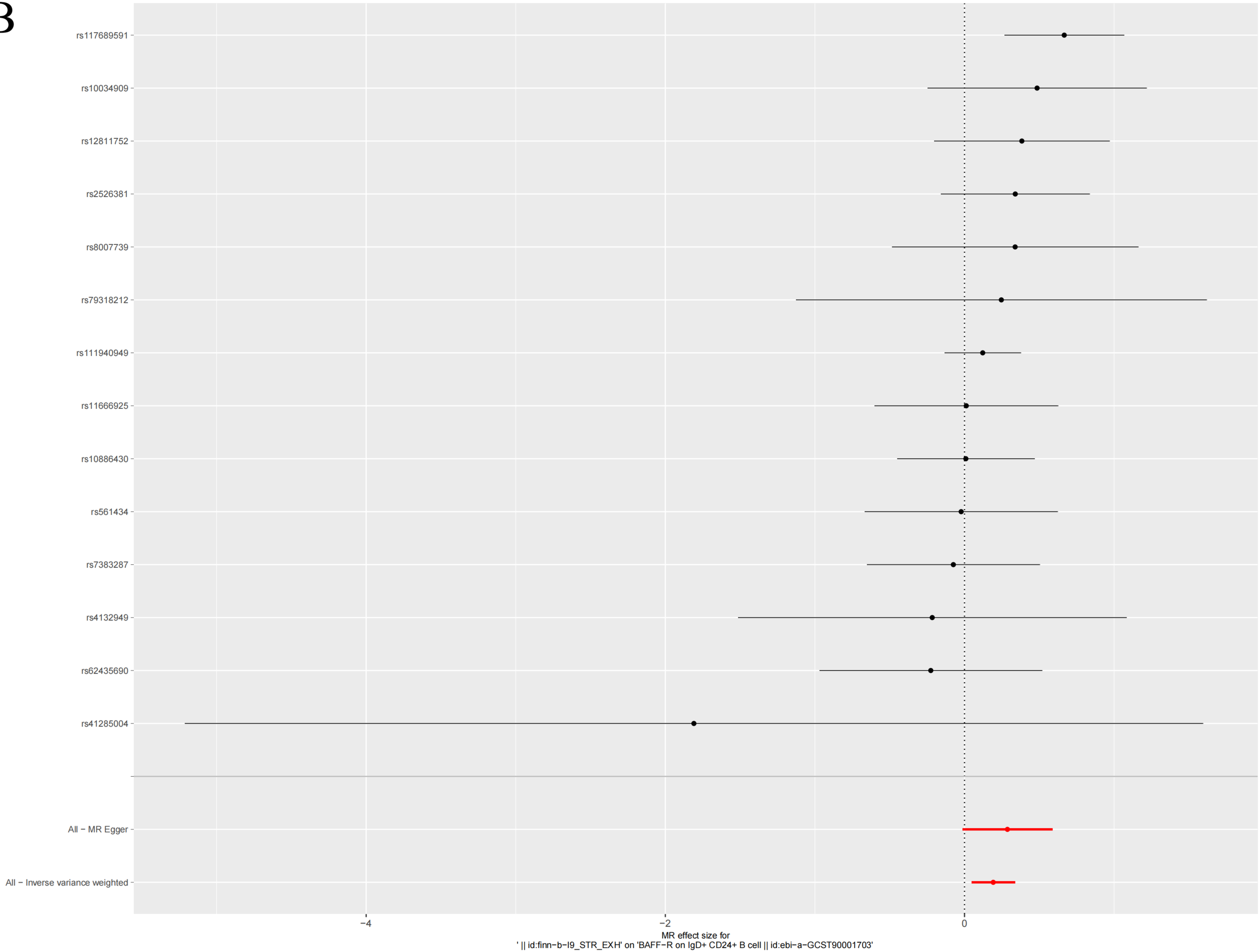

rs117689591
rs10034909
rs12811752
rs2526381
rs8007739
rs79318212
rs111940949
rs11666925
rs10886430
rs561434
rs7383287
rs4132949
rs62435690
rs41285004
All − MR Egger
All − Inverse variance weighted
−4
−2
0
MR effect size for
' || id:finn−b−I9_STR_EXH' on 'BAFF−R on IgD+ CD24+ B cell || id:ebi−a−GCST90001703'

C

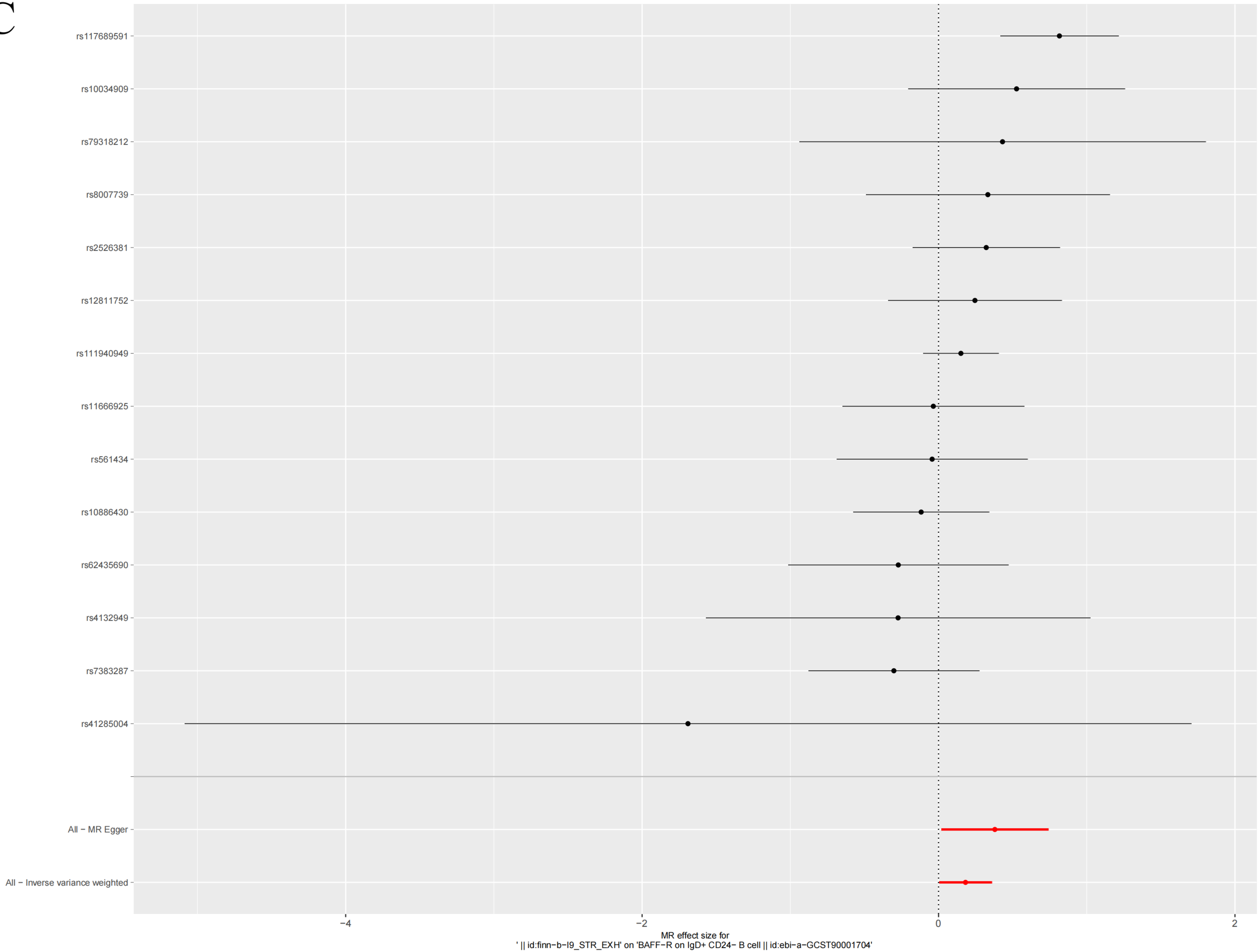
rs117689591
rs10034909
rs79318212
rs8007739
rs2526381
rs12811752
rs111940949
rs11666925
rs561434
rs10886430
rs62435690
rs4132949
rs7383287
rs41285004
All − MR Egger
All − Inverse variance weighted
−4
−2
0
2
MR effect size for
' || id:finn−b−I9_STR_EXH' on 'BAFF−R on IgD+ CD24− B cell || id:ebi−a−GCST90001704'

D

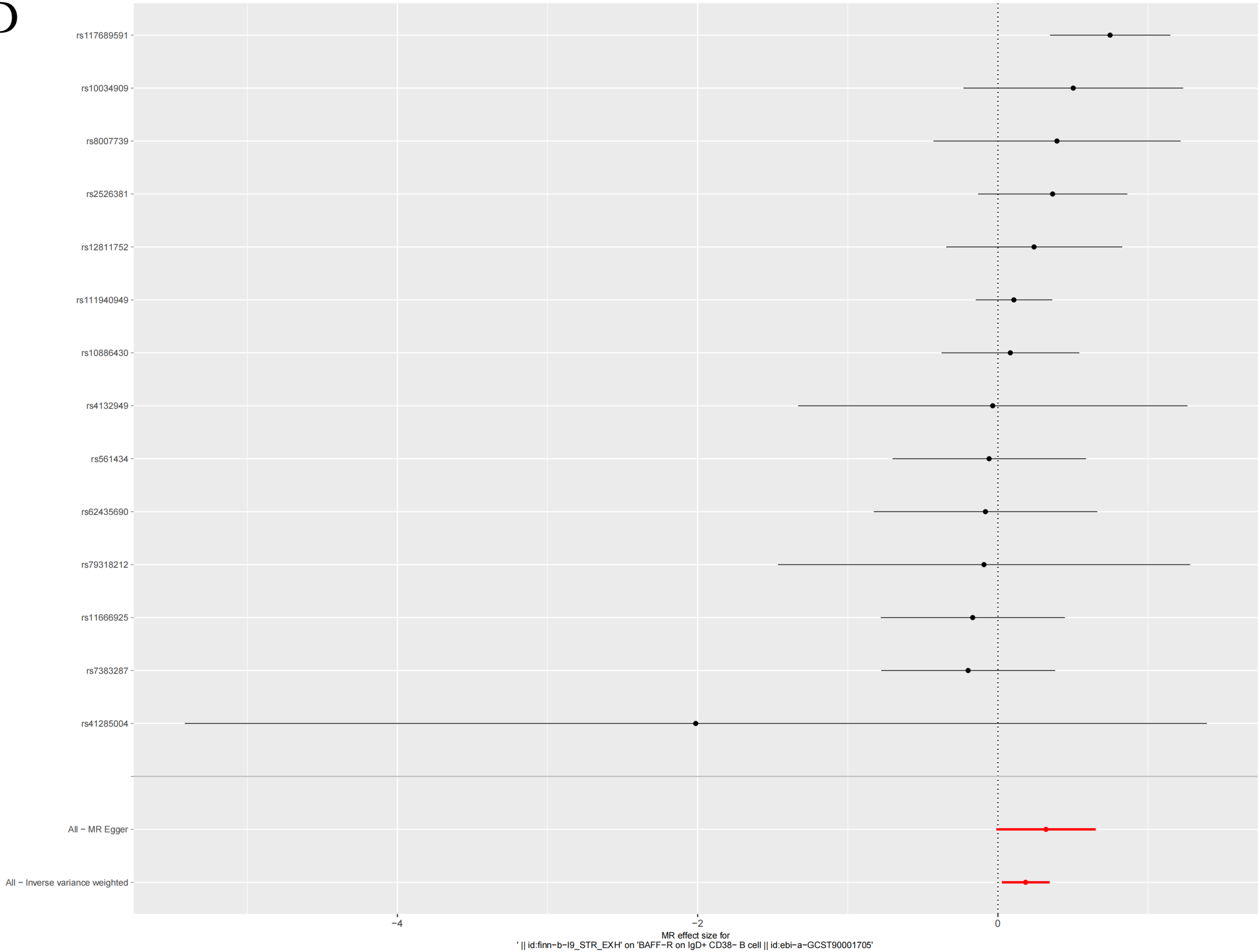

rs117689591
rs10034909
rs8007739
rs2526381
rs12811752
rs111940949
rs10886430
rs4132949
rs561434
rs62435690
rs79318212
rs11666925
rs7383287
rs41285004
All − MR Egger
All − Inverse variance weighted
−4
−2
0
MR effect size for
' || id:finn−b−I9_STR_EXH' on 'BAFF−R on IgD+ CD38− B cell || id:ebi−a−GCST90001705'

E

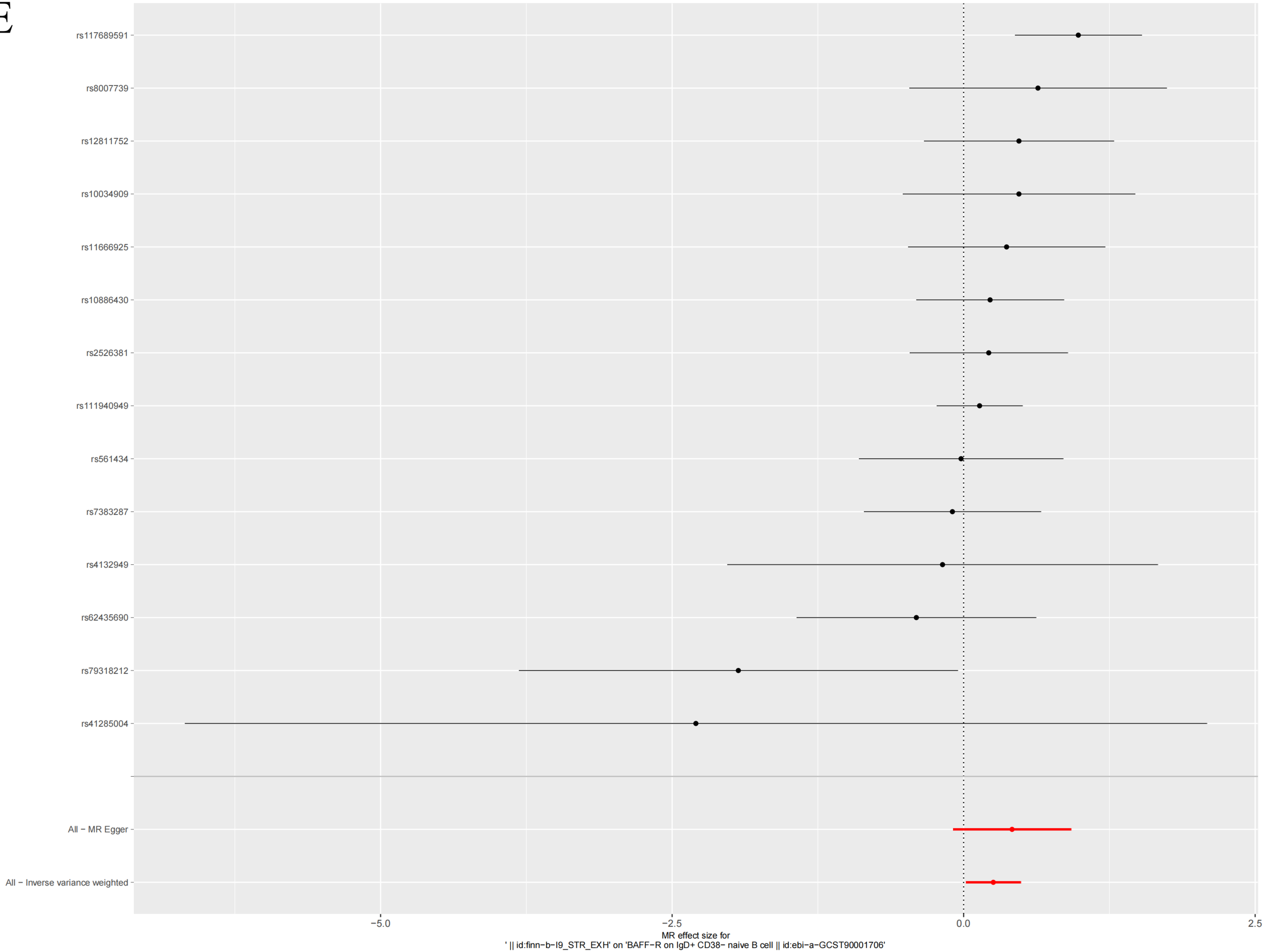
rs117689591
rs8007739
rs12811752
rs10034909
rs11666925
rs10886430
rs2526381
rs111940949
rs561434
rs7383287
rs4132949
rs62435690
rs79318212
rs41285004
All − MR Egger
All − Inverse variance weighted
−5.0
−2.5
0.0
2.5
MR effect size for
' || id:finn−b−I9_STR_EXH' on 'BAFF−R on IgD+ CD38− naive B cell || id:ebi−a−GCST90001706'

F

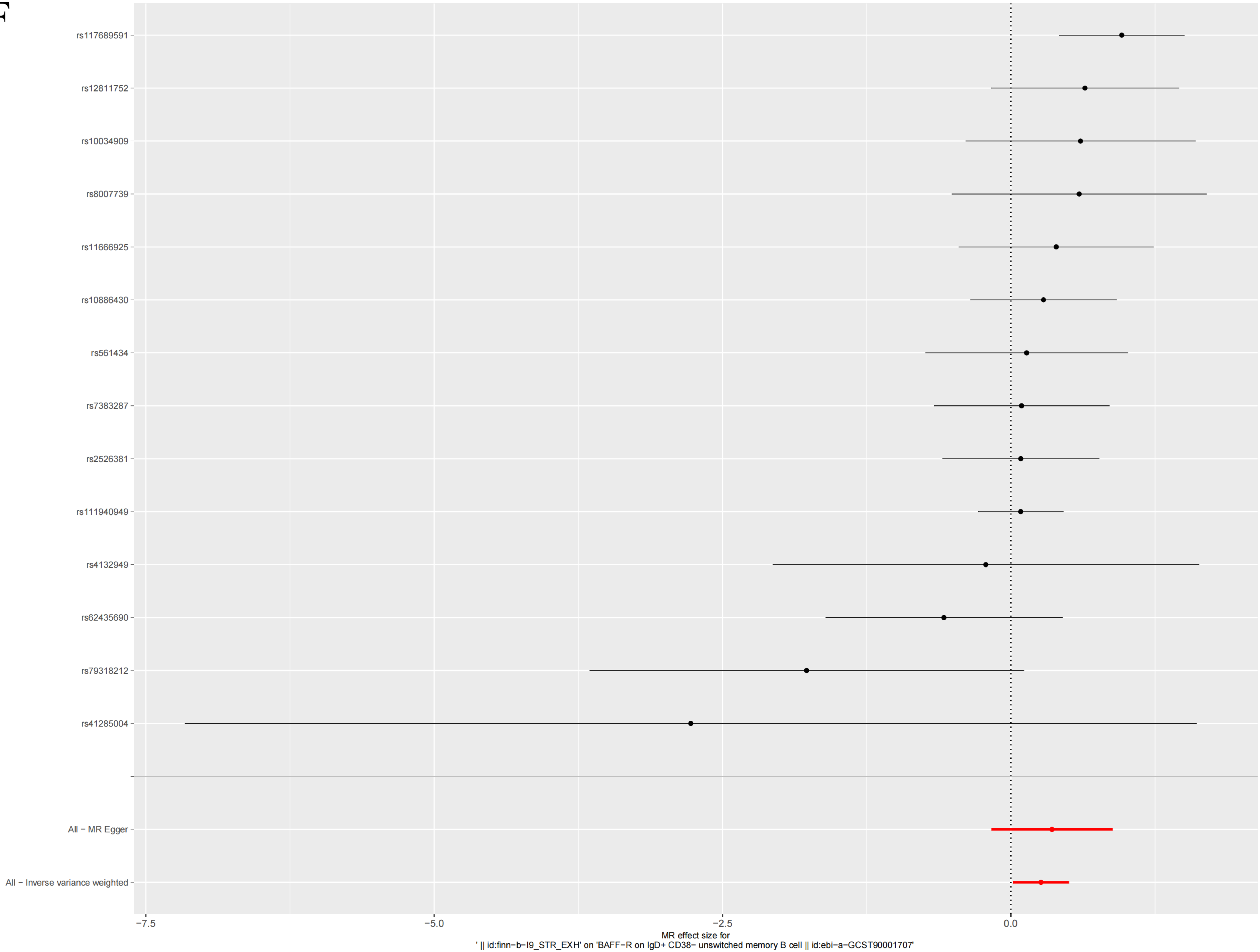
rs117689591
rs12811752
rs10034909
rs8007739
rs11666925
rs10886430
rs561434
rs7383287
rs2526381
rs111940949
rs4132949
rs62435690
rs79318212
rs41285004
All − MR Egger
All − Inverse variance weighted
−7.5
−5.0
−2.5
0.0
MR effect size for
' || id:finn−b−I9_STR_EXH' on 'BAFF−R on IgD+ CD38− unswitched memory B cell || id:ebi−a−GCST90001707'

G

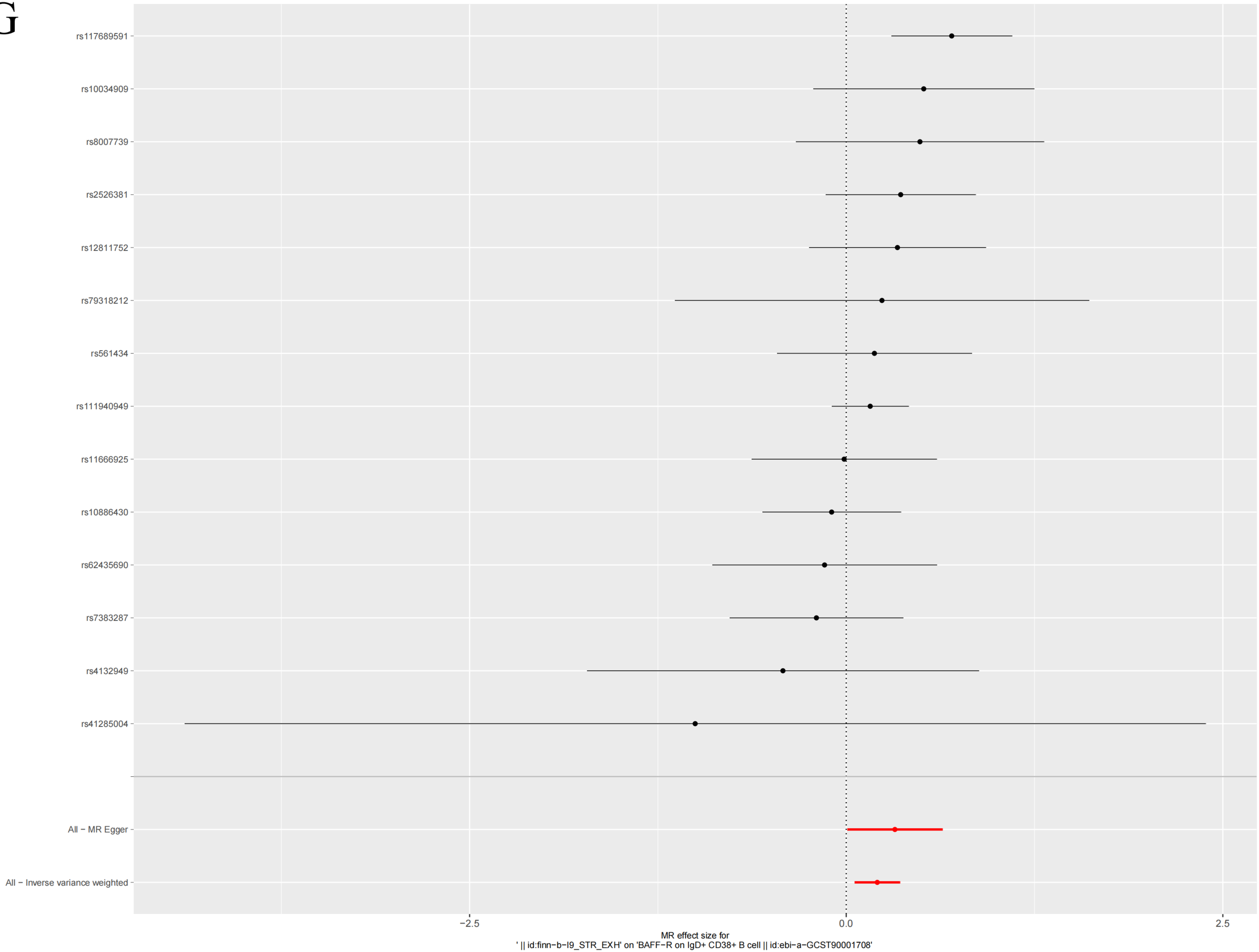

rs117689591
rs10034909
rs8007739
rs2526381
rs12811752
rs79318212
rs561434
rs111940949
rs11666925
rs10886430
rs62435690
rs7383287
rs4132949
rs41285004
All − MR Egger
All − Inverse variance weighted
−2.5
0.0
2.5
MR effect size for
' || id:finn−b−I9_STR_EXH' on 'BAFF−R on IgD+ CD38+ B cell || id:ebi−a−GCST90001708'

H

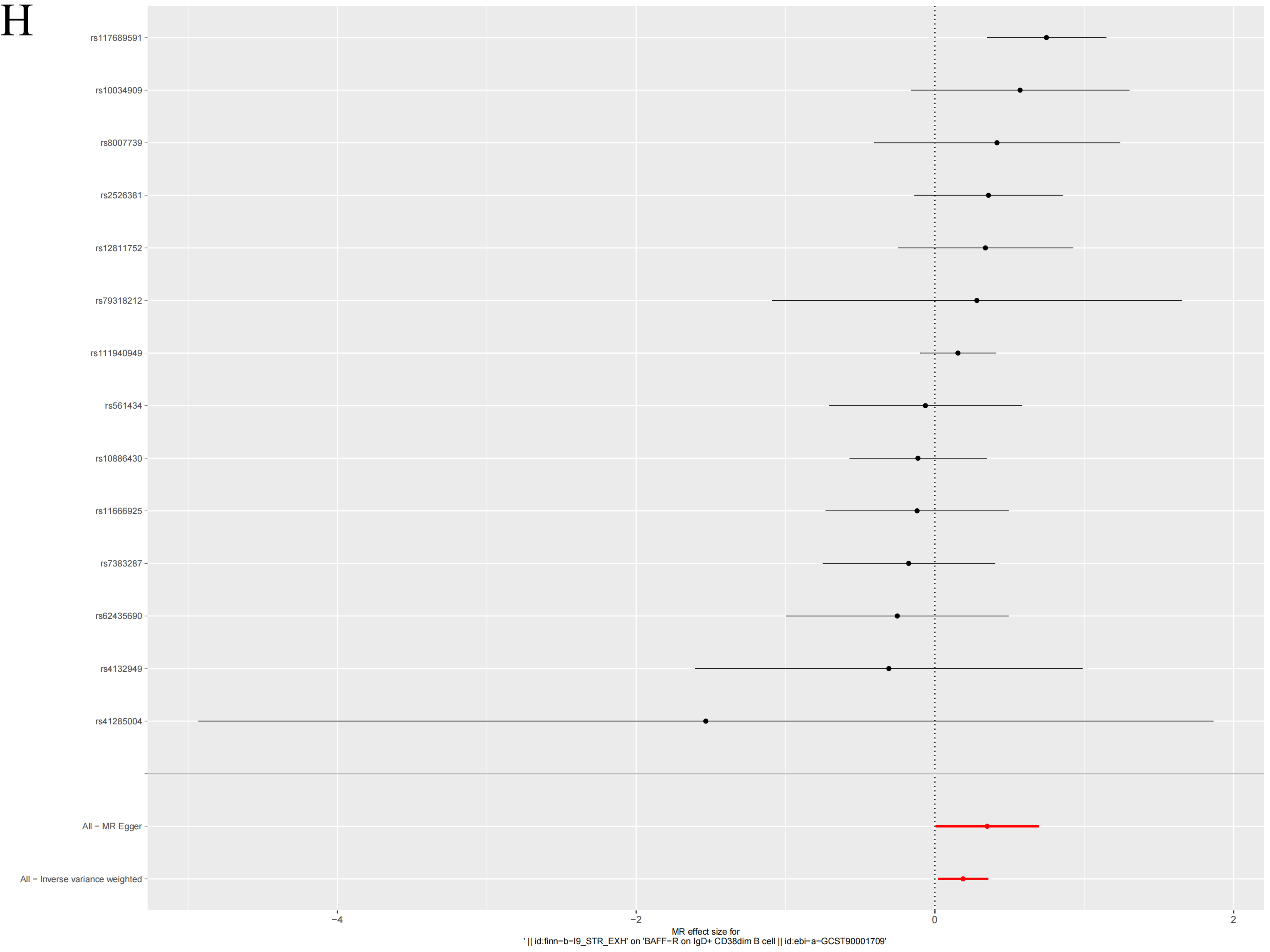
rs117689591
rs10034909
rs8007739
rs2526381
rs12811752
rs79318212
rs111940949
rs561434
rs10886430
rs11666925
rs7383287
rs62435690
rs4132949
rs41285004
All − MR Egger
All − Inverse variance weighted
−4
−2
0
2
MR effect size for
' || id:finn−b−I9_STR_EXH' on 'BAFF−R on IgD+ CD38dim B cell || id:ebi−a−GCST90001709'

I

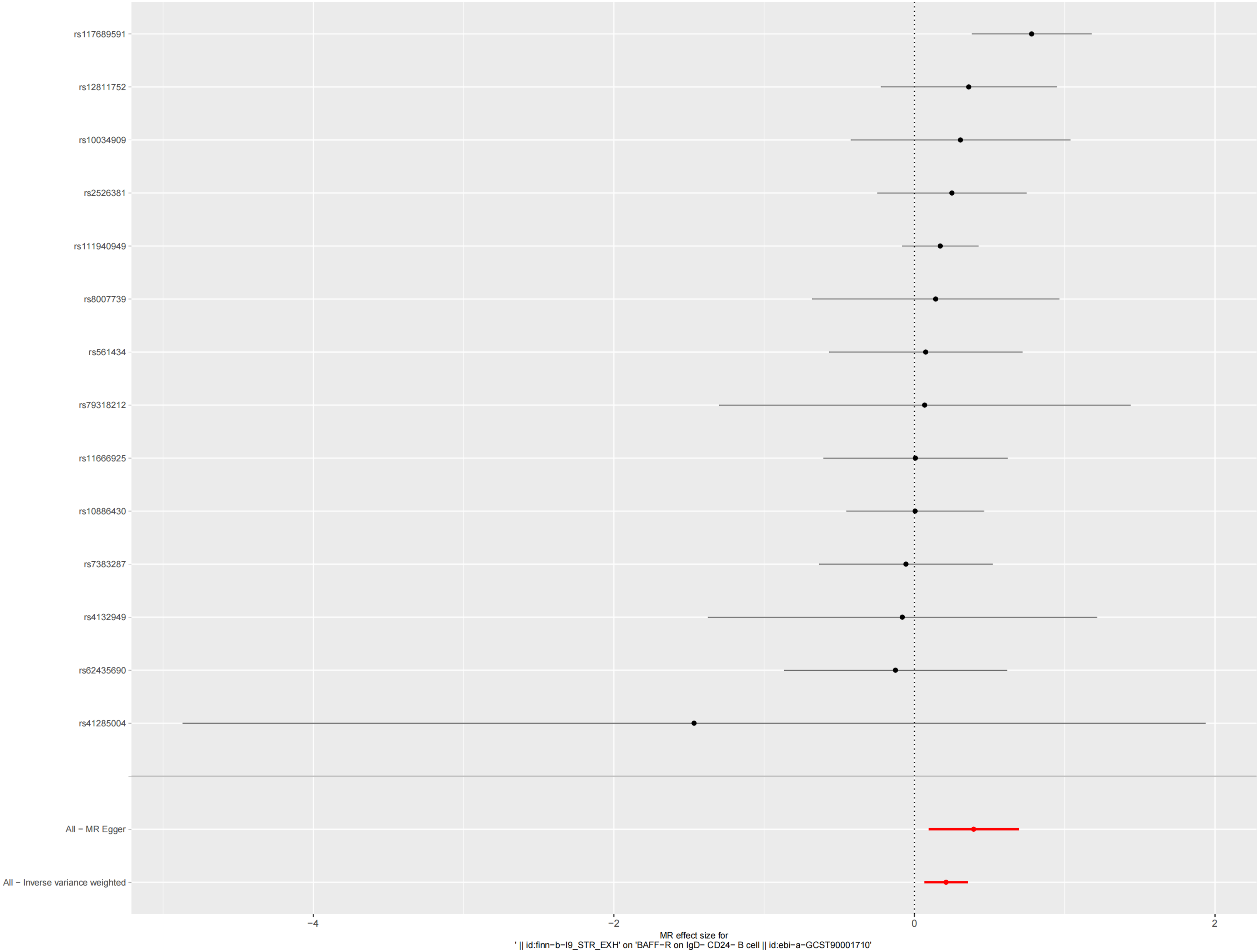
rs117689591
rs12811752
rs10034909
rs2526381
rs111940949
rs8007739
rs561434
rs79318212
rs11666925
rs10886430
rs7383287
rs4132949
rs62435690
rs41285004
All − MR Egger
All − Inverse variance weighted
−4
−2
0
2
MR effect size for
' || id:finn−b−I9_STR_EXH' on 'BAFF−R on IgD− CD24− B cell || id:ebi−a−GCST90001710'

J

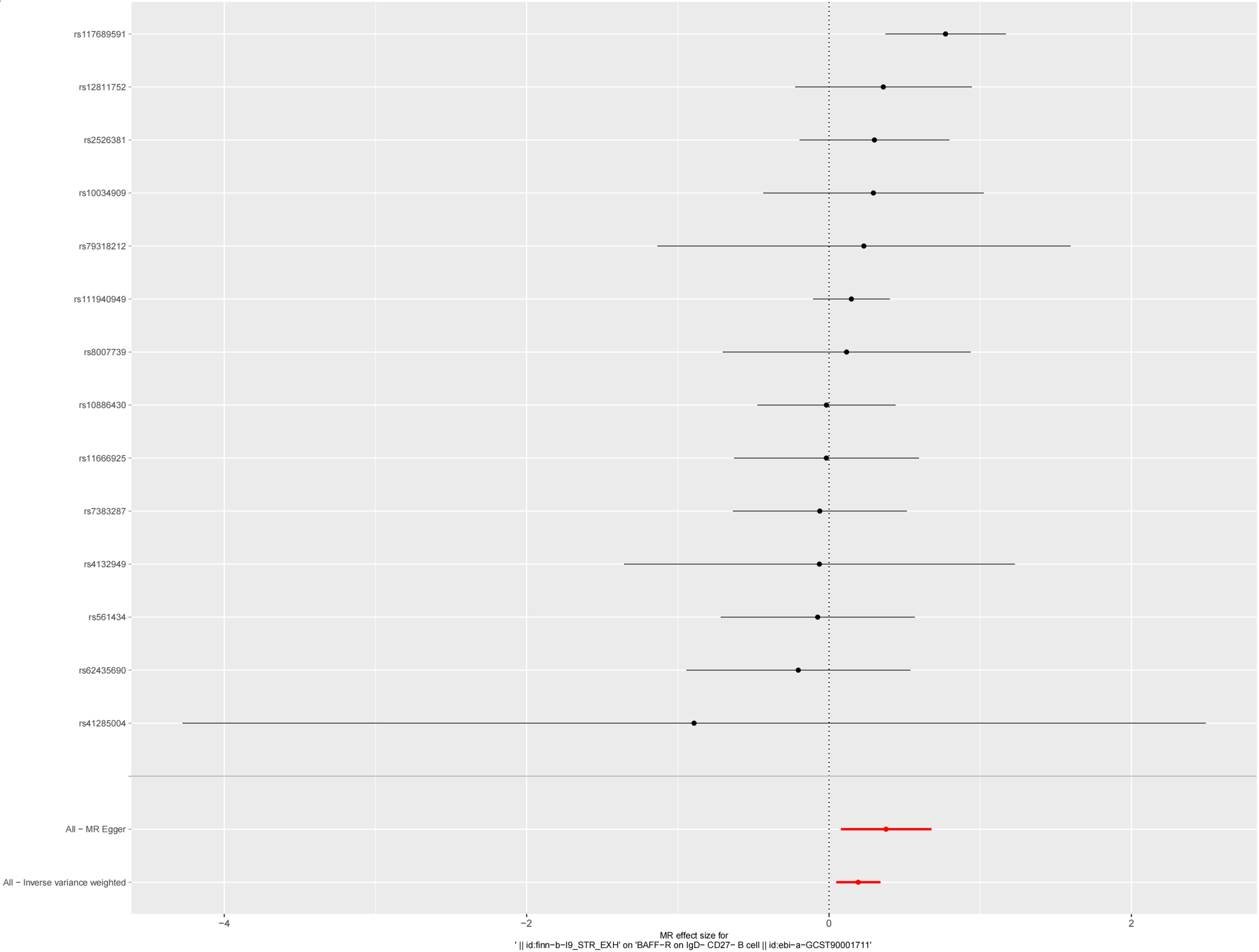

rs117689591
rs12811752
rs2526381
rs10034909
rs79318212
rs111940949
rs8007739
rs10886430
rs11666925
rs7383287
rs4132949
rs561434
rs62435690
rs41285004
All − MR Egger
All − Inverse variance weighted
−4
−2
0
2
MR effect size for
' || id:finn-b-I9_STR_EXH' on 'BAFF−R on IgD− CD27− B cell || id:ebi-a-GCST90001711'

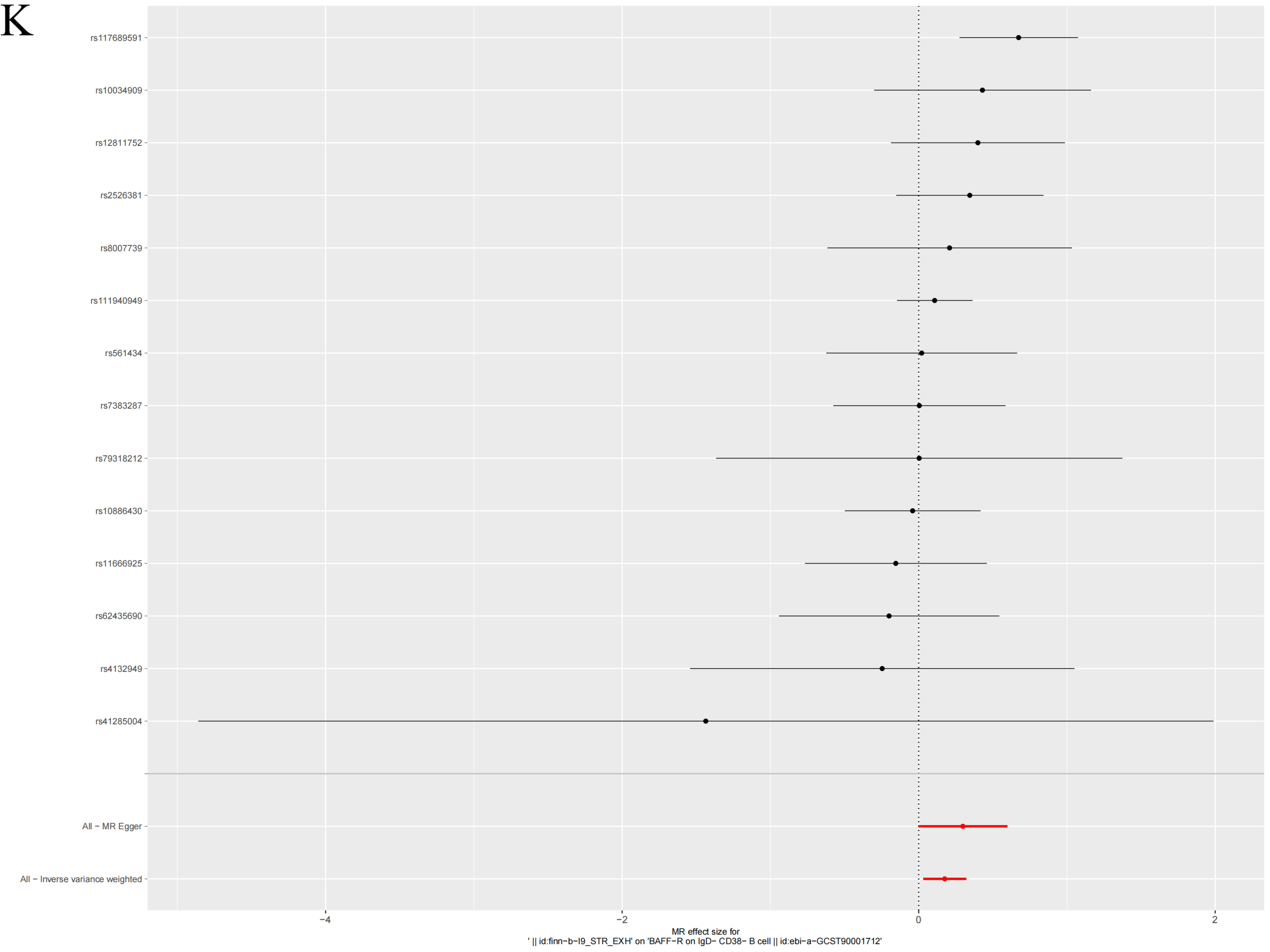
K
rs117689591
rs10034909
rs12811752
rs2526381
rs8007739
rs111940949
rs561434
rs7383287
rs79318212
rs10886430
rs11666925
rs62435690
rs4132949
rs41285004
All − MR Egger
All − Inverse variance weighted
−4
−2
0
2
MR effect size for
' || id:finn-b-I9_STR_EXH' on 'BAFF−R on IgD− CD38− B cell || id:ebi-a-GCST90001712'

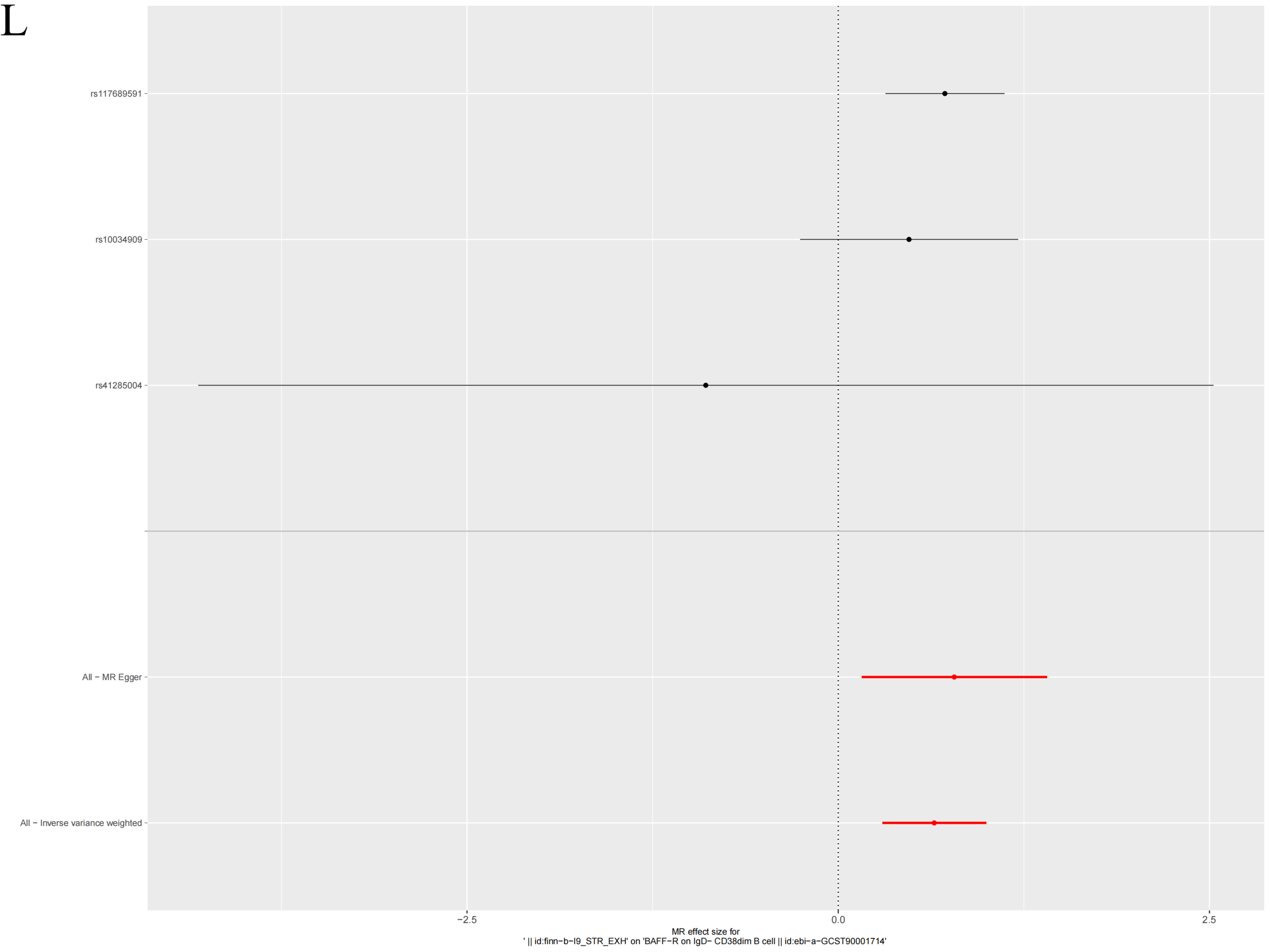
L
rs117689591
rs10034909
rs41285004
All − MR Egger
All − Inverse variance weighted
−2.5
0.0
2.5
MR effect size for
' || id:finn−b−I9_STR_EXH' on 'BAFF−R on IgD− CD38dim B cell || id:ebi−a−GCST90001714'

M

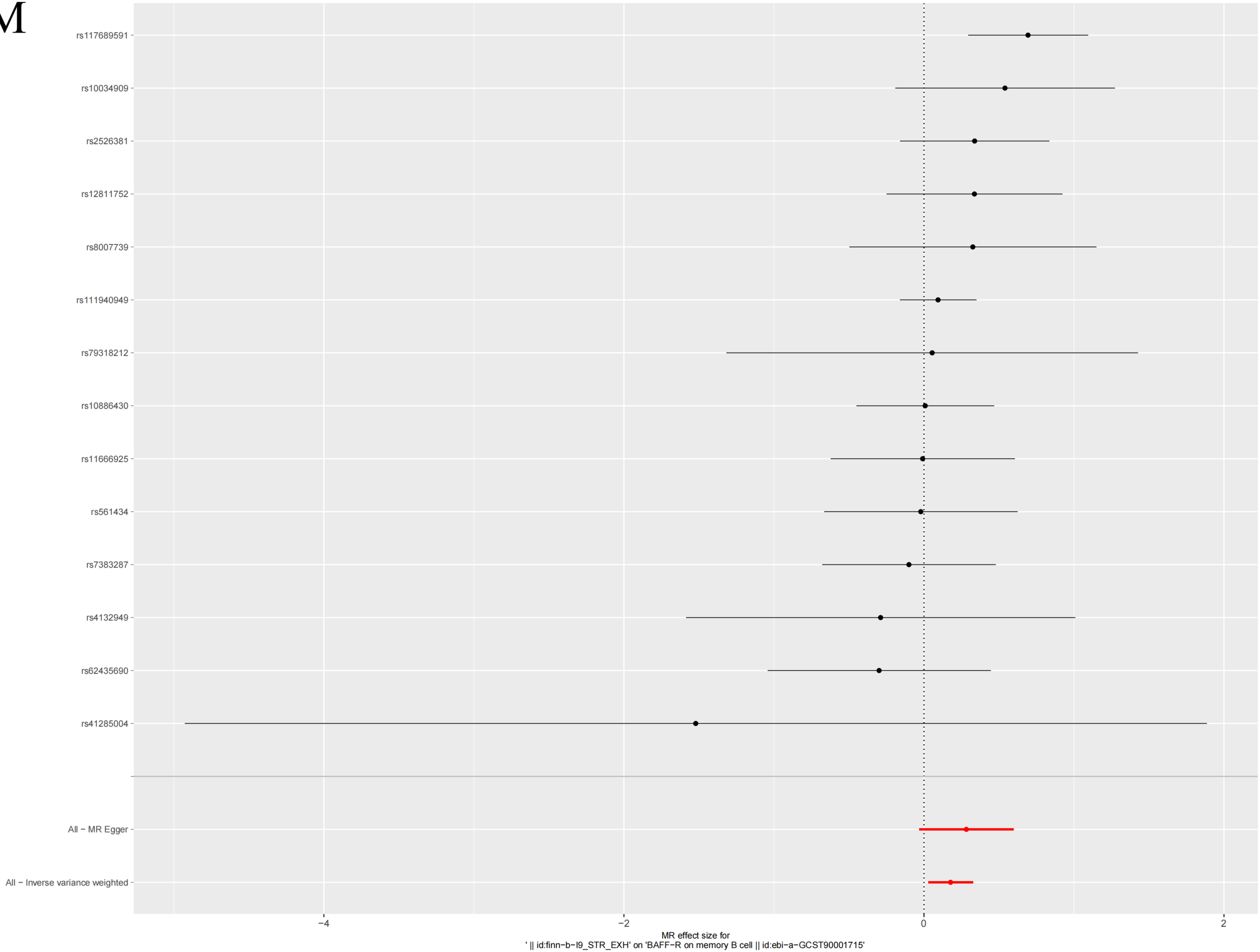

rs117689591
rs10034909
rs2526381
rs12811752
rs8007739
rs111940949
rs79318212
rs10886430
rs11666925
rs561434
rs7383287
rs4132949
rs62435690
rs41285004
All − MR Egger
All − Inverse variance weighted
−4
−2
0
2
MR effect size for
' || id:finn-b-I9_STR_EXH' on 'BAFF−R on memory B cell || id:ebi-a-GCST90001715'

N

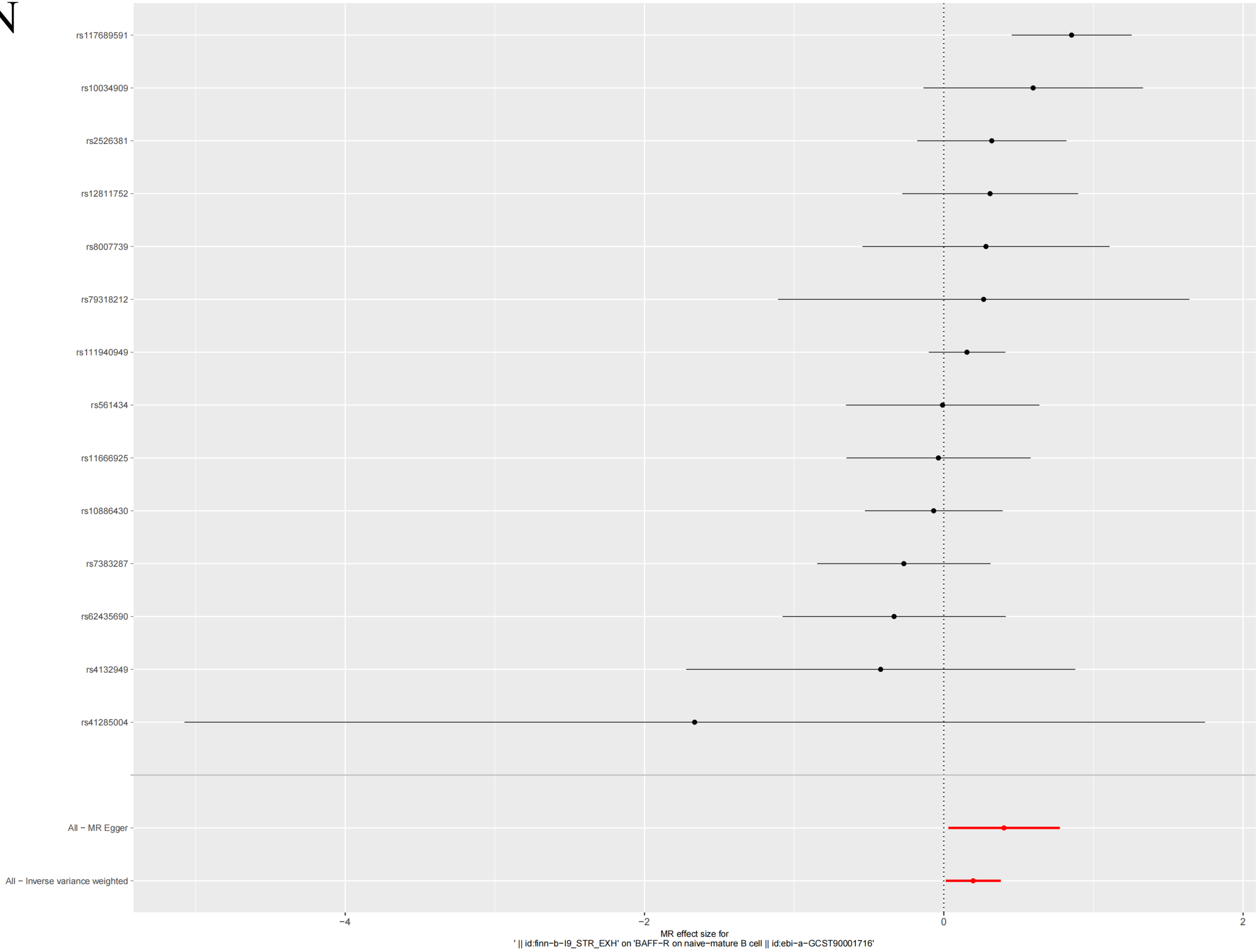
rs117689591
rs10034909
rs2526381
rs12811752
rs8007739
rs79318212
rs111940949
rs561434
rs11666925
rs10886430
rs7383287
rs62435690
rs4132949
rs41285004
All − MR Egger
All − Inverse variance weighted
−4
−2
0
2
MR effect size for
' || id:finn−b−I9_STR_EXH' on 'BAFF−R on naive−mature B cell || id:ebi−a−GCST90001716'

O

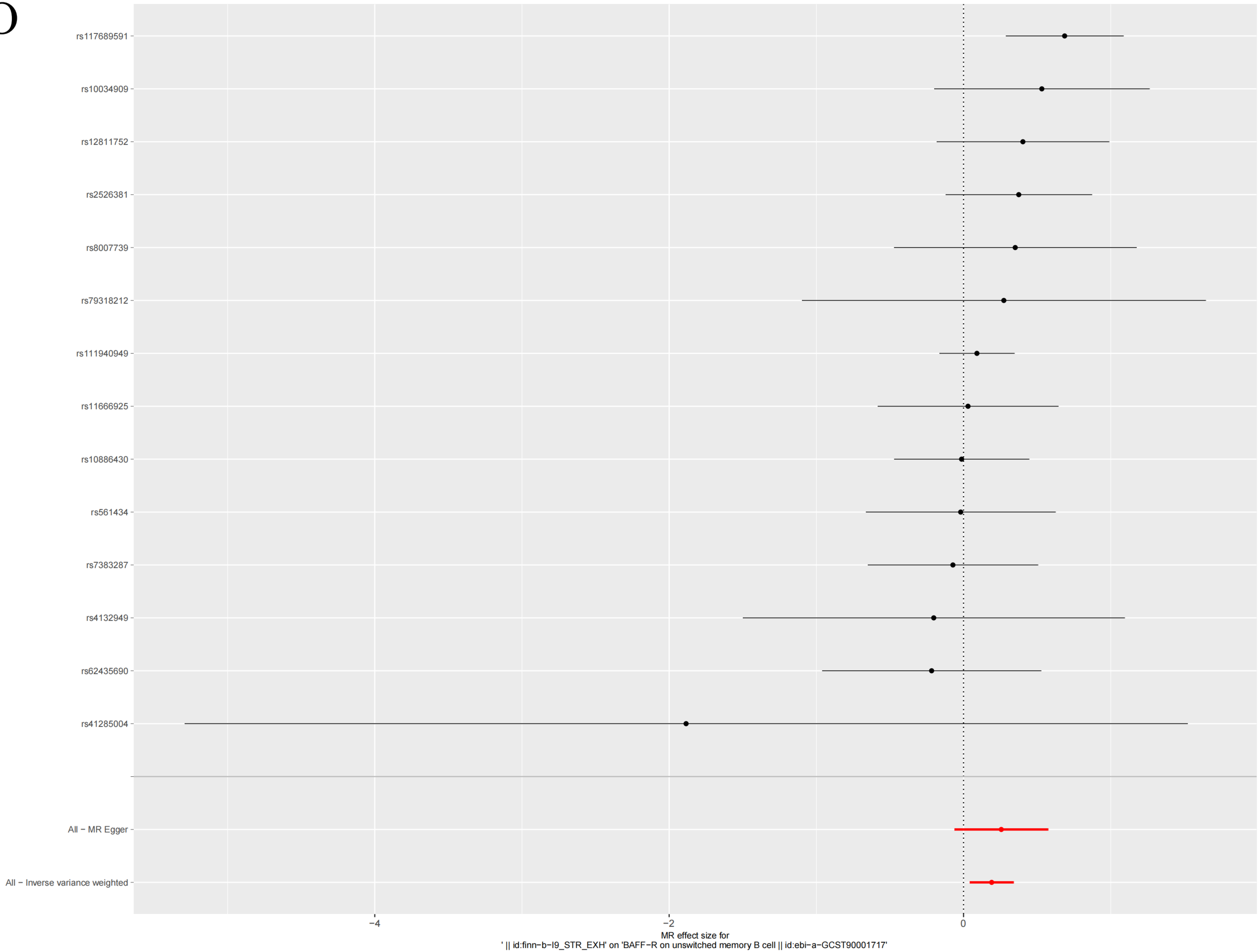

rs117689591
rs10034909
rs12811752
rs2526381
rs8007739
rs79318212
rs111940949
rs11666925
rs10886430
rs561434
rs7383287
rs4132949
rs62435690
rs41285004
All − MR Egger
All − Inverse variance weighted
−4
−2
0
MR effect size for
' || id:finn-b-I9_STR_EXH' on 'BAFF−R on unswitched memory B cell || id:ebi-a-GCST90001717'

P

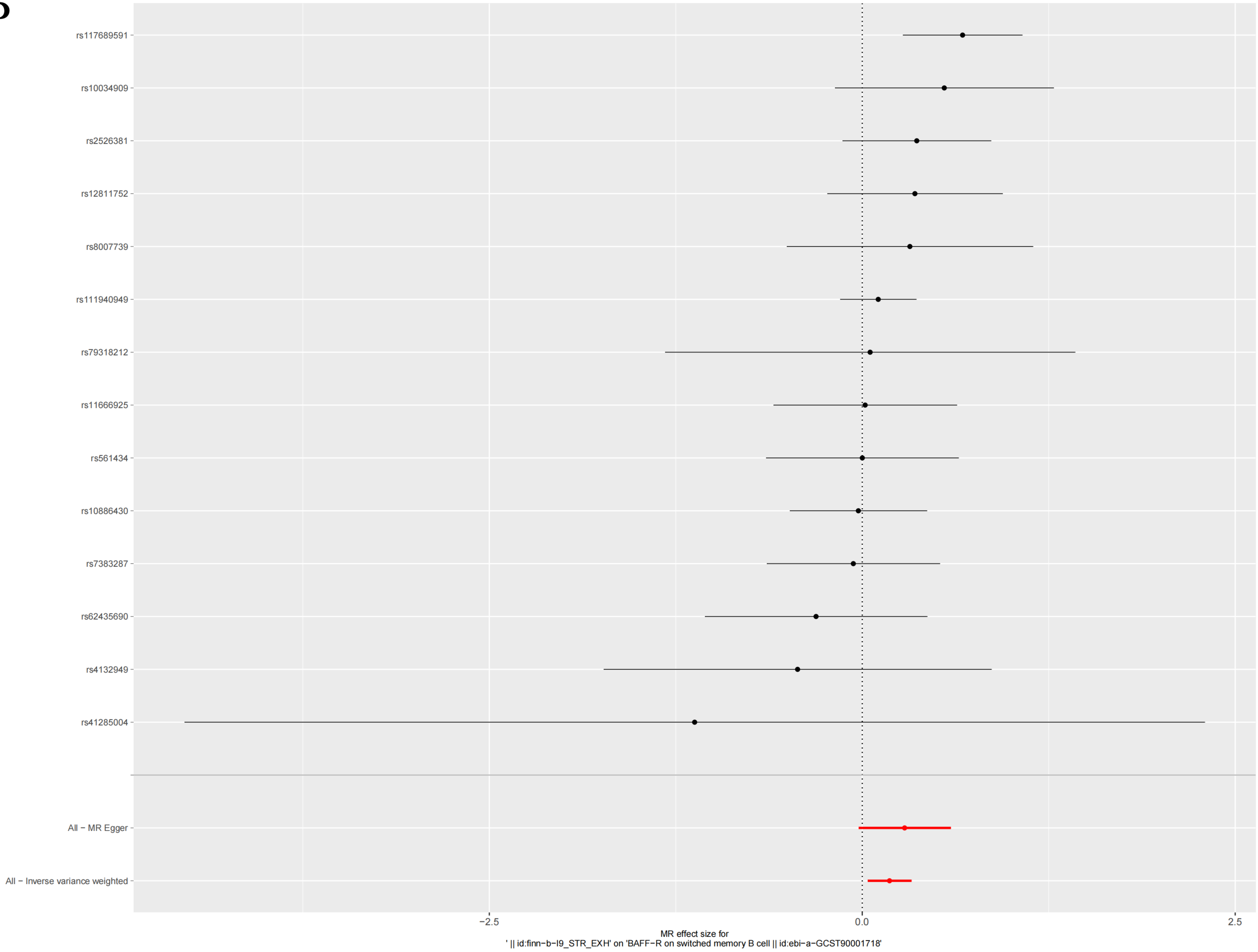
rs117689591
rs10034909
rs2526381
rs12811752
rs8007739
rs111940949
rs79318212
rs11666925
rs561434
rs10886430
rs7383287
rs62435690
rs4132949
rs41285004
All − MR Egger
All − Inverse variance weighted
−2.5
0.0
2.5
MR effect size for
' || id:finn−b−I9_STR_EXH' on 'BAFF−R on switched memory B cell || id:ebi−a−GCST90001718'

Q

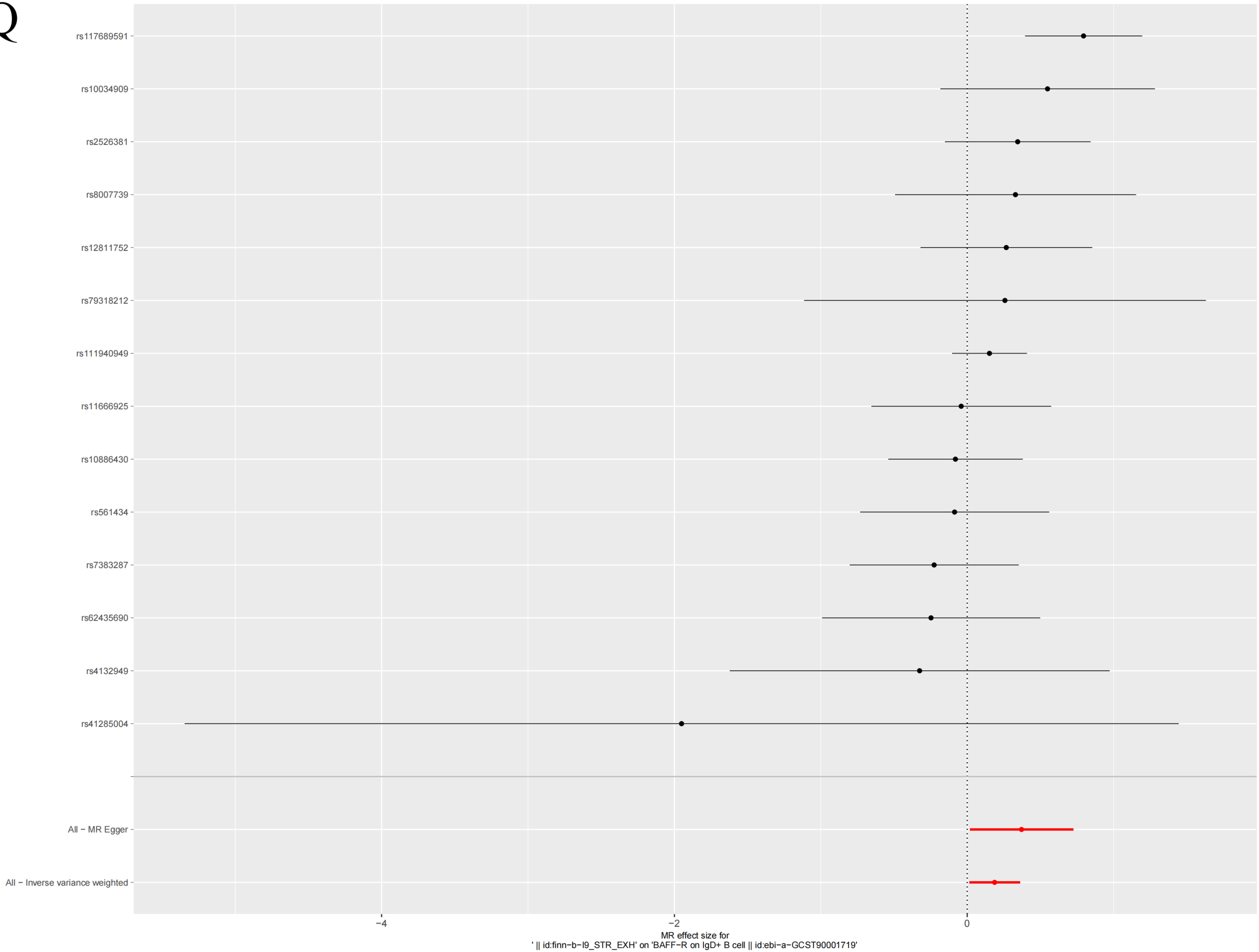

rs117689591
rs10034909
rs2526381
rs8007739
rs12811752
rs79318212
rs111940949
rs11666925
rs10886430
rs561434
rs7383287
rs62435690
rs4132949
rs41285004
All − MR Egger
All − Inverse variance weighted
−4
−2
0
MR effect size for
' || id:finn−b−I9_STR_EXH' on 'BAFF−R on IgD+ B cell || id:ebi−a−GCST90001719'

R

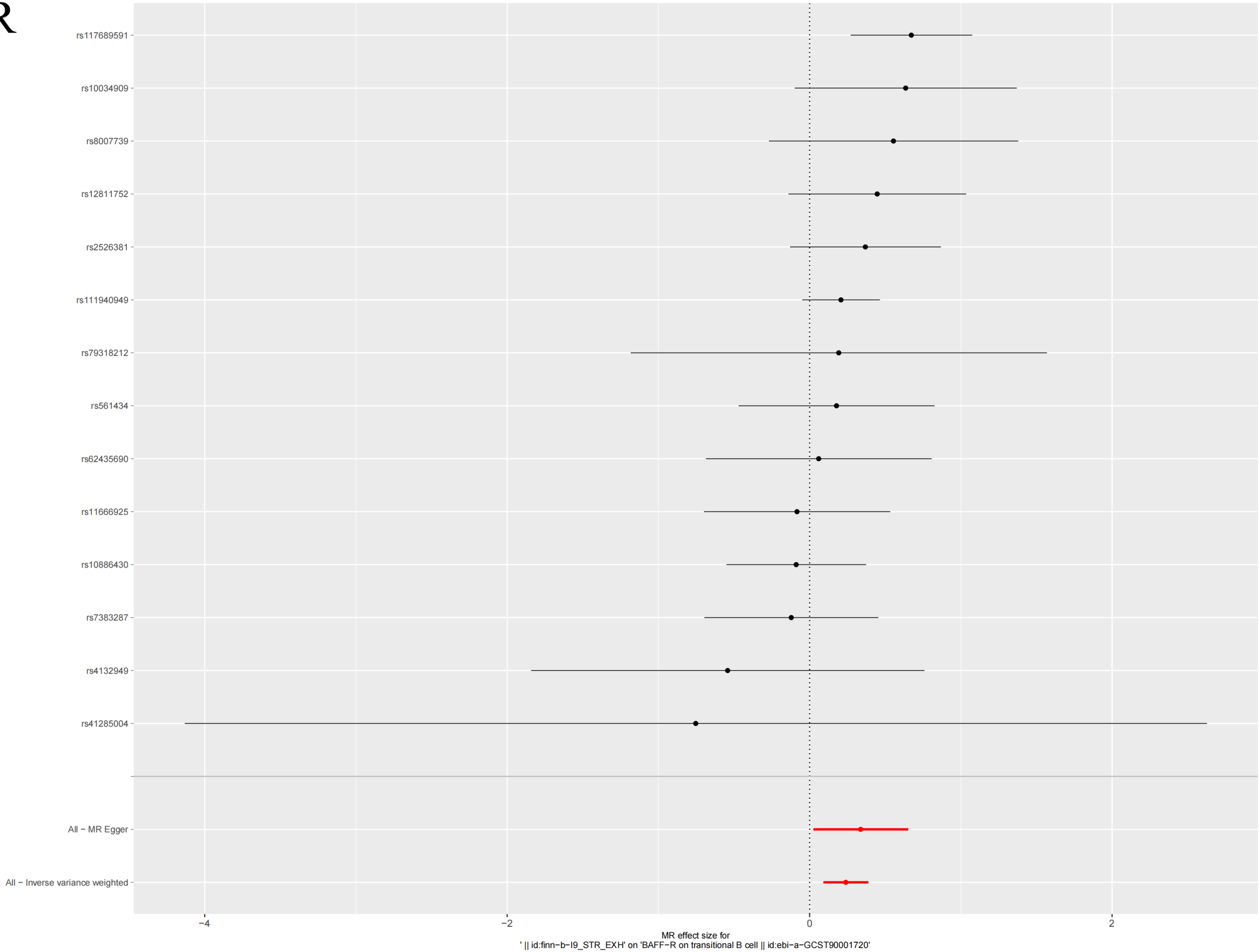
rs117689591
rs10034909
rs8007739
rs12811752
rs2526381
rs111940949
rs79318212
rs561434
rs62435690
rs11666925
rs10886430
rs7383287
rs4132949
rs41285004
All − MR Egger
All − Inverse variance weighted
−4
−2
0
2
MR effect size for
' || id:finn−b−I9_STR_EXH' on 'BAFF−R on transitional B cell || id:ebi−a−GCST90001720'

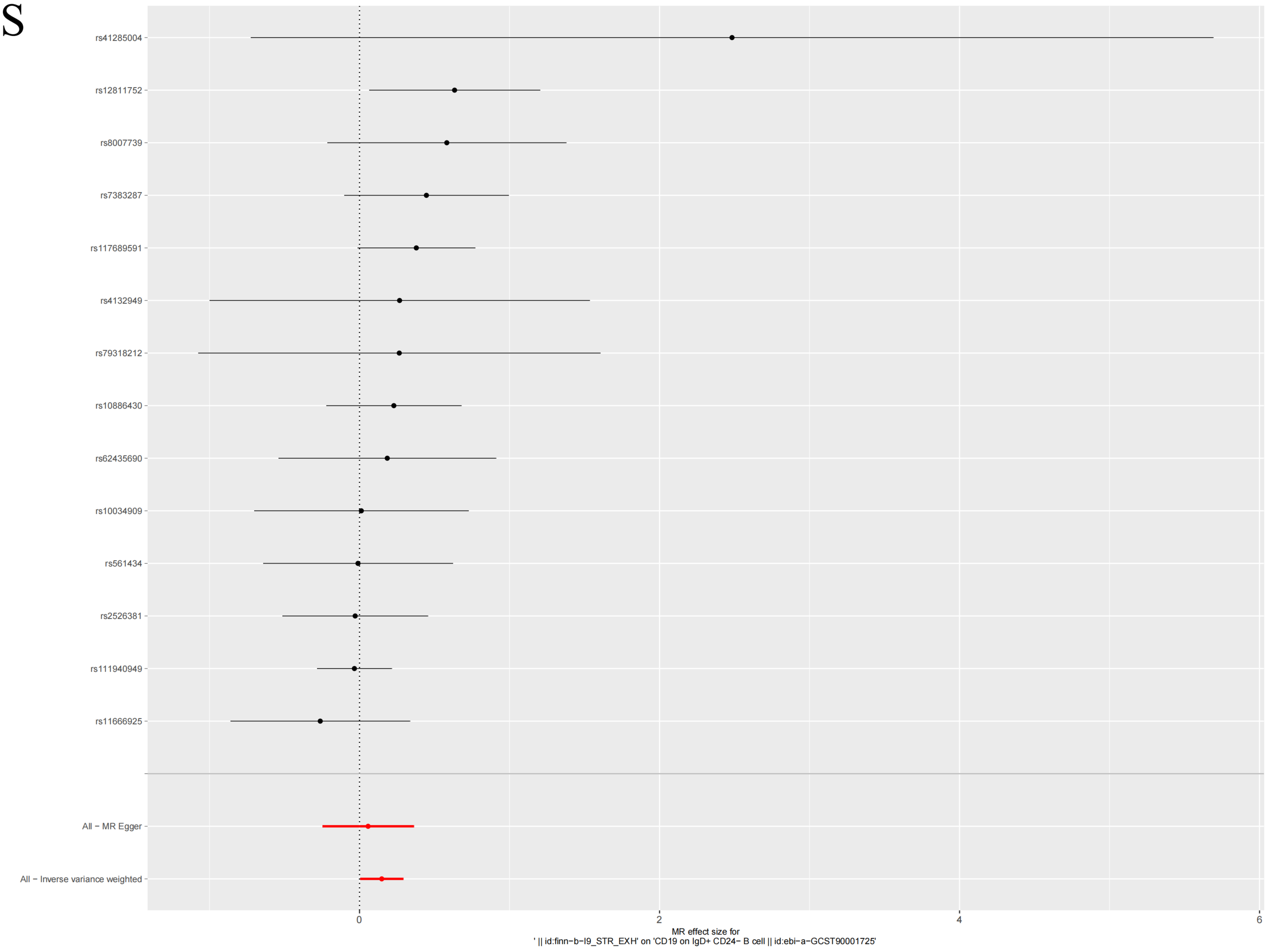
S
rs41285004
rs12811752
rs8007739
rs7383287
rs117689591
rs4132949
rs79318212
rs10886430
rs62435690
rs10034909
rs561434
rs2526381
rs111940949
rs11666925
All − MR Egger
All − Inverse variance weighted
0
2
4
6
MR effect size for
' || id:finn−b−I9_STR_EXH' on 'CD19 on IgD+ CD24− B cell || id:ebi−a−GCST90001725'

T

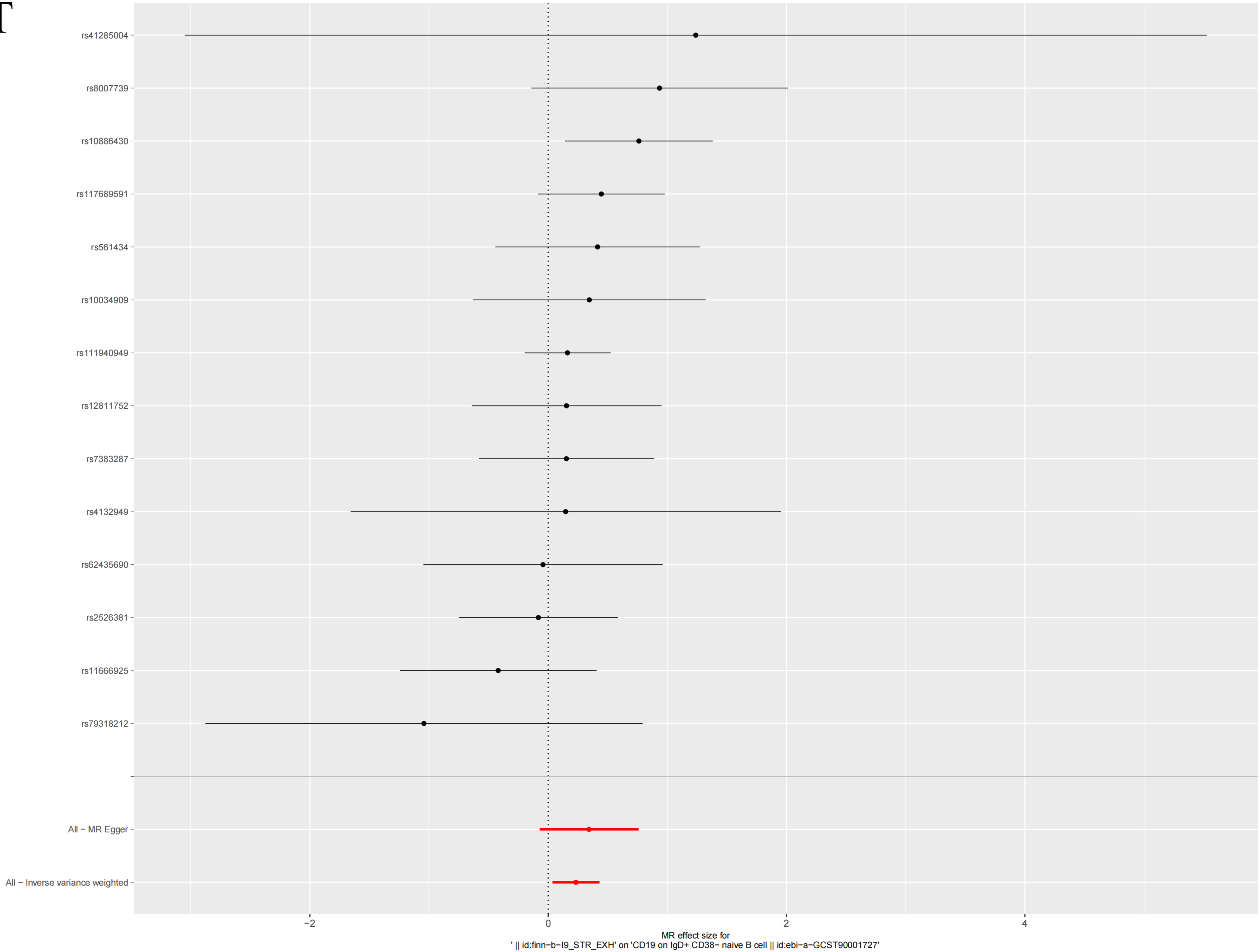
rs41285004
rs8007739
rs10886430
rs117689591
rs561434
rs10034909
rs111940949
rs12811752
rs7383287
rs4132949
rs62435690
rs2526381
rs11666925
rs79318212
All − MR Egger
All − Inverse variance weighted
−2
0
2
4
MR effect size for
' || id:finn−b−I9_STR_EXH' on 'CD19 on IgD+ CD38− naive B cell || id:ebi−a−GCST90001727'

U

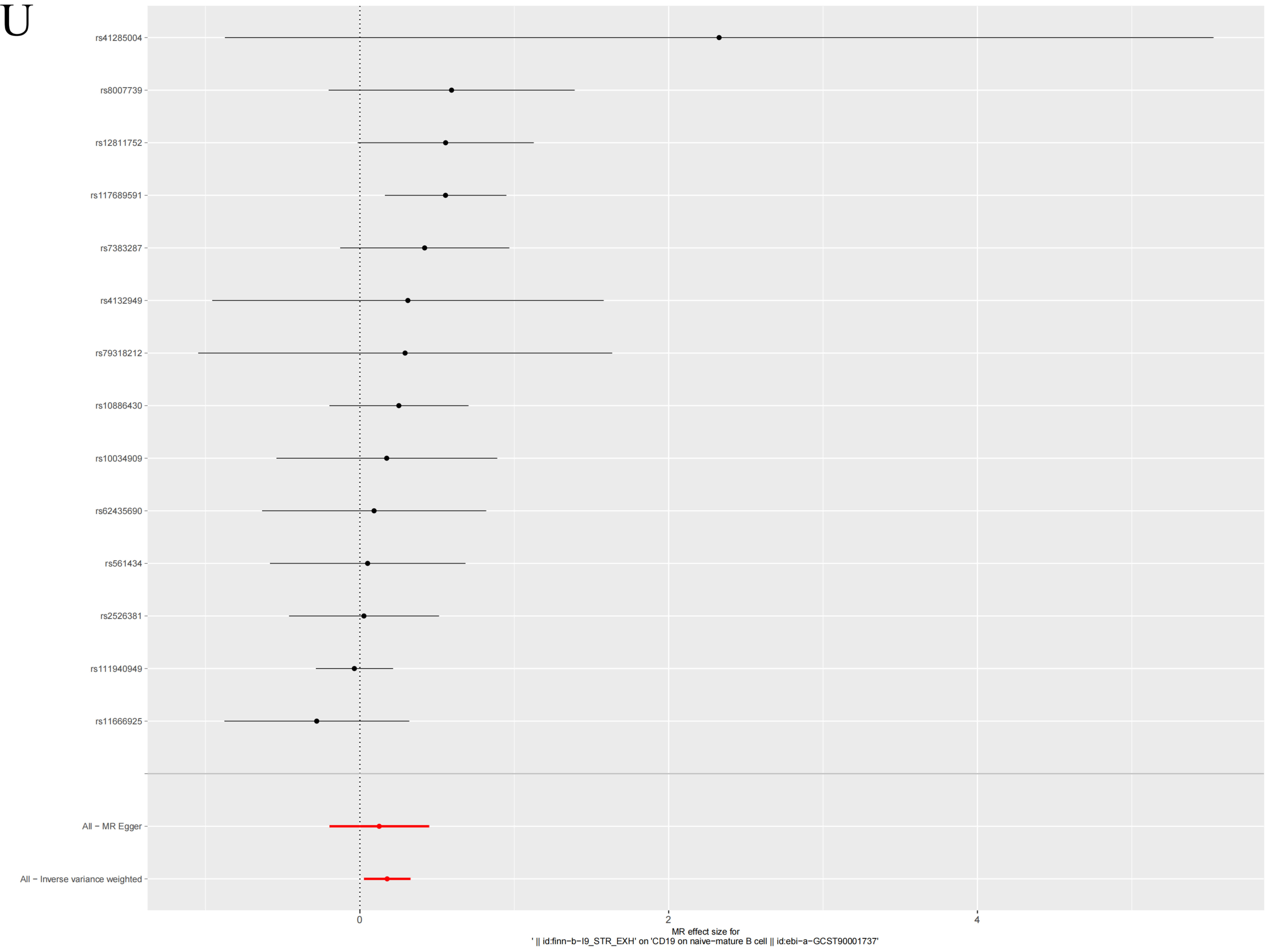

rs41285004
rs8007739
rs12811752
rs117689591
rs7383287
rs4132949
rs79318212
rs10886430
rs10034909
rs62435690
rs561434
rs2526381
rs111940949
rs11666925
All − MR Egger
All − Inverse variance weighted
0
2
4
MR effect size for
' || id:finn−b−I9_STR_EXH' on 'CD19 on naive−mature B cell || id:ebi−a−GCST90001737'

V

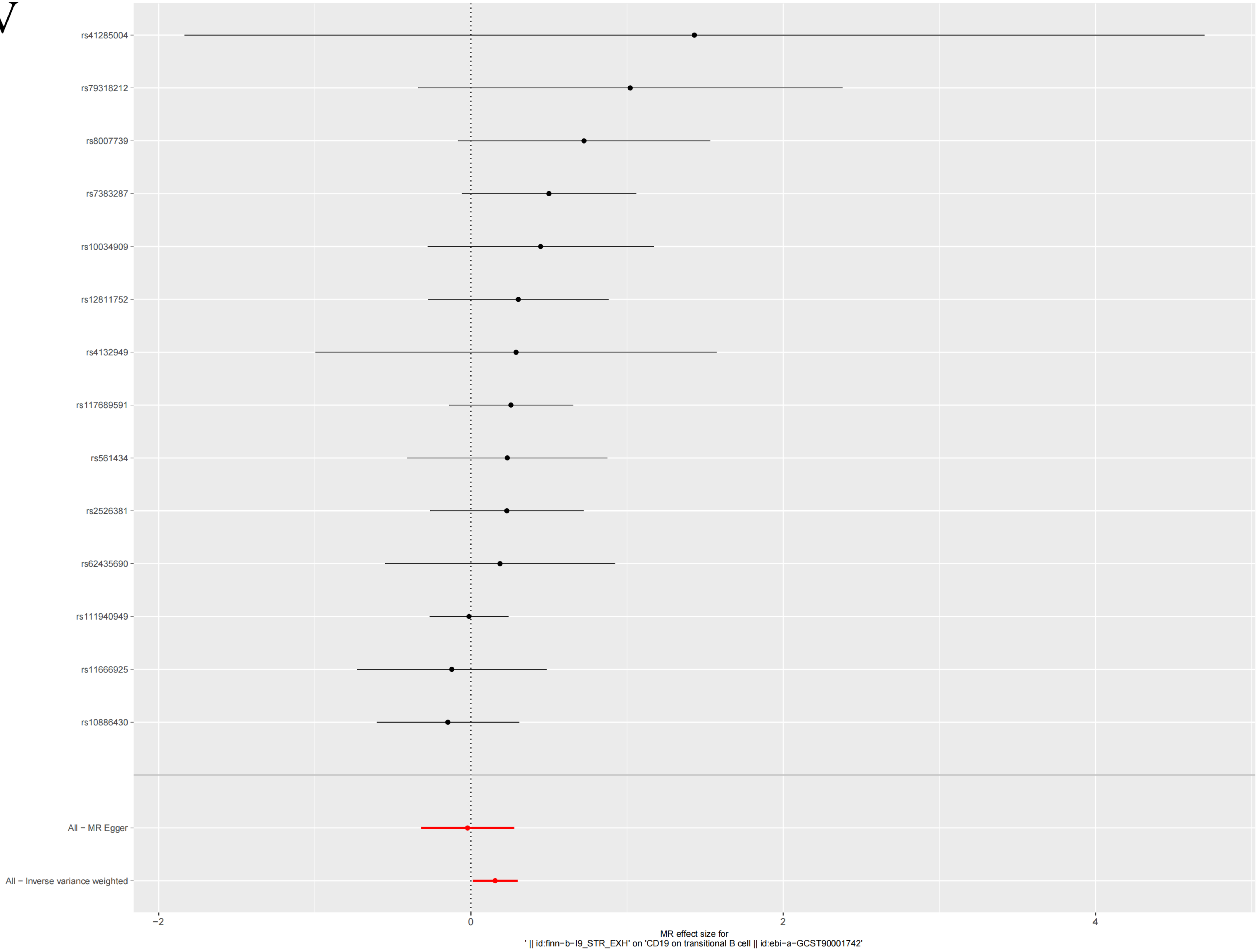

rs41285004
rs79318212
rs8007739
rs7383287
rs10034909
rs12811752
rs4132949
rs117689591
rs561434
rs2526381
rs62435690
rs111940949
rs11666925
rs10886430
All − MR Egger
All − Inverse variance weighted
−2
0
2
4
MR effect size for
' || id:finn−b−I9_STR_EXH' on 'CD19 on transitional B cell || id:ebi−a−GCST90001742'

W

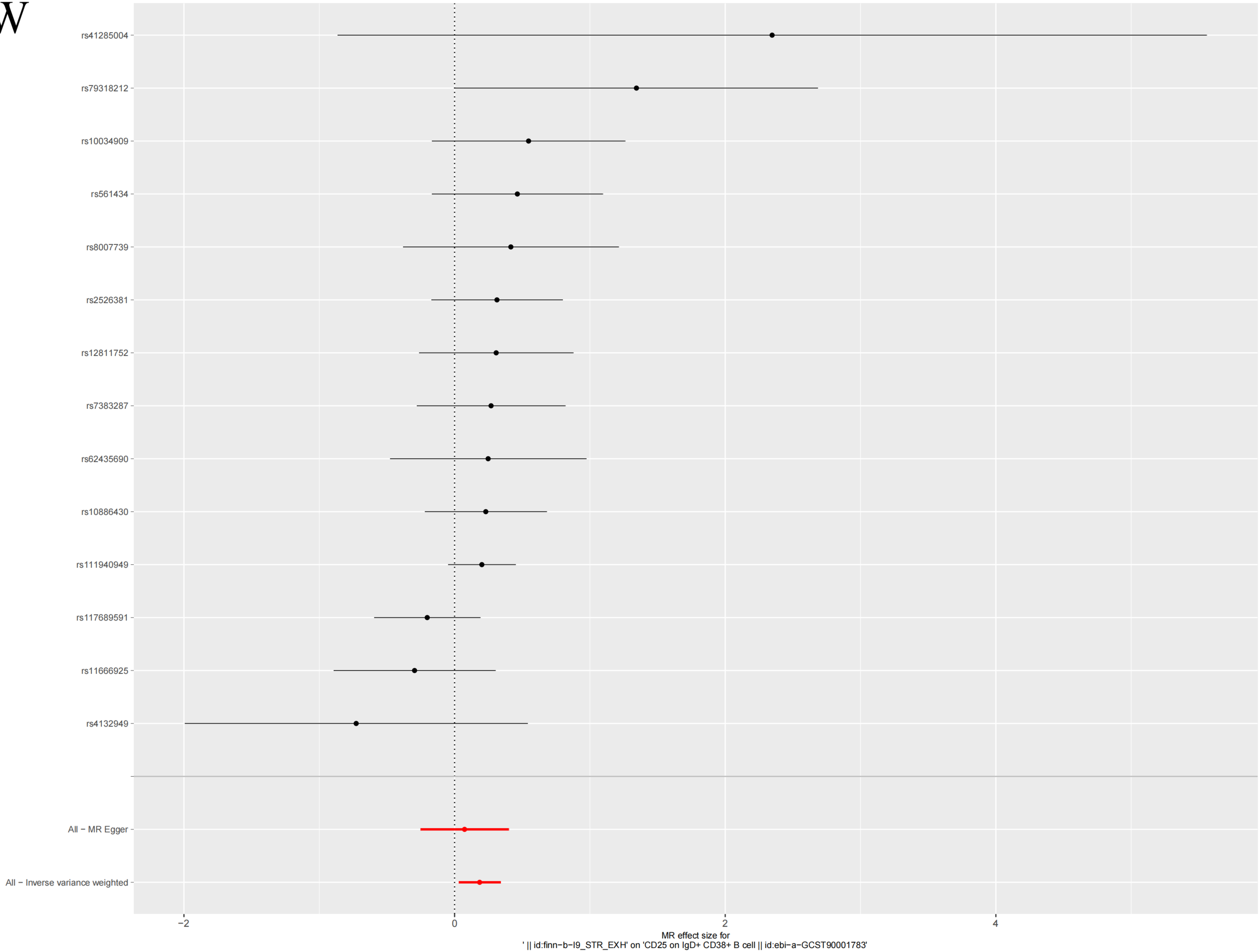
rs41285004
rs79318212
rs10034909
rs561434
rs8007739
rs2526381
rs12811752
rs7383287
rs62435690
rs10886430
rs111940949
rs117689591
rs11666925
rs4132949
All − MR Egger
All − Inverse variance weighted
−2
0
2
4
MR effect size for
' || id:finn−b−I9_STR_EXH' on 'CD25 on IgD+ CD38+ B cell || id:ebi−a−GCST90001783'

X

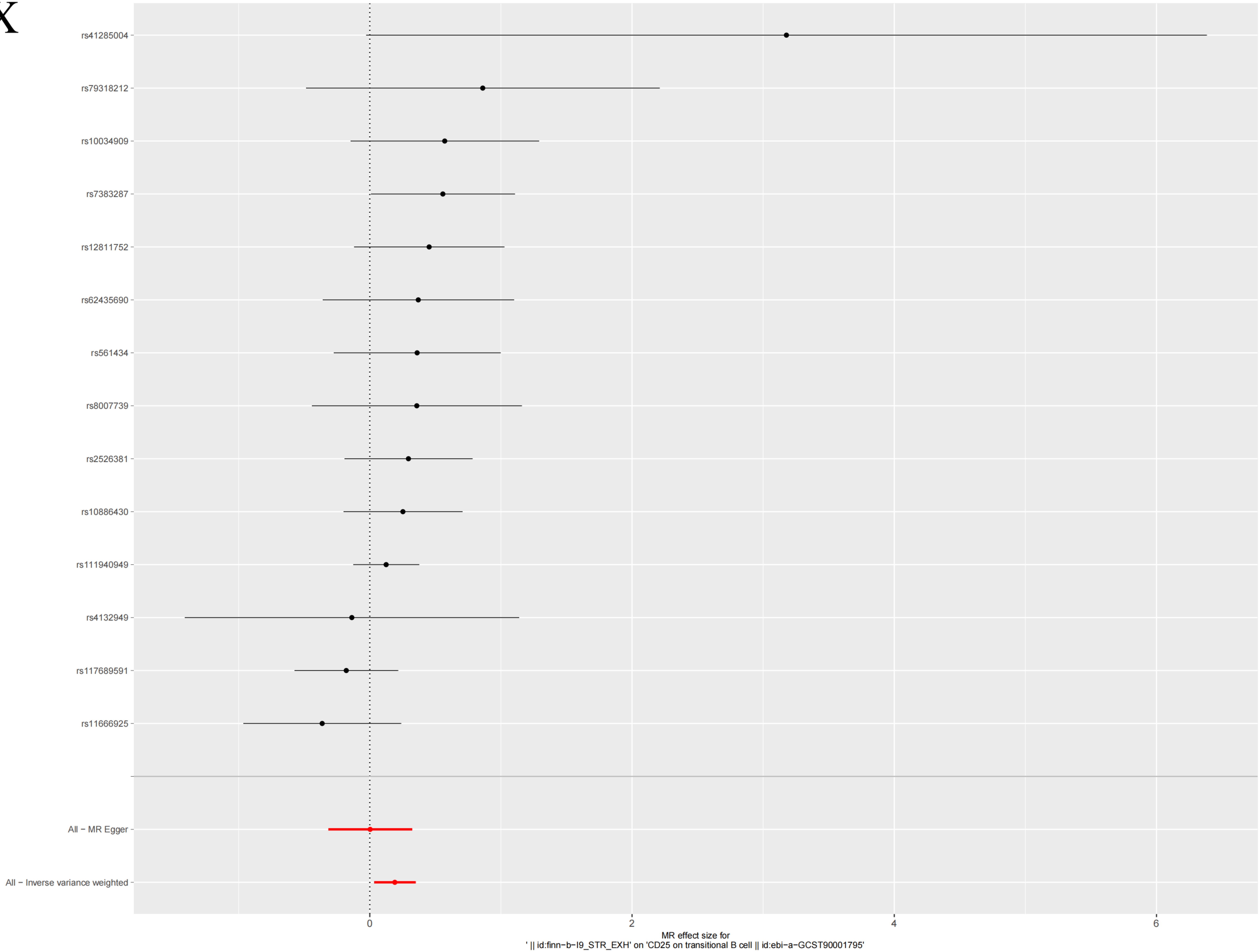
rs41285004
rs79318212
rs10034909
rs7383287
rs12811752
rs62435690
rs561434
rs8007739
rs2526381
rs10886430
rs111940949
rs4132949
rs117689591
rs11666925
All − MR Egger
All − Inverse variance weighted
0
2
4
6
MR effect size for
' || id:finn−b−I9_STR_EXH' on 'CD25 on transitional B cell || id:ebi−a−GCST90001795'

Y

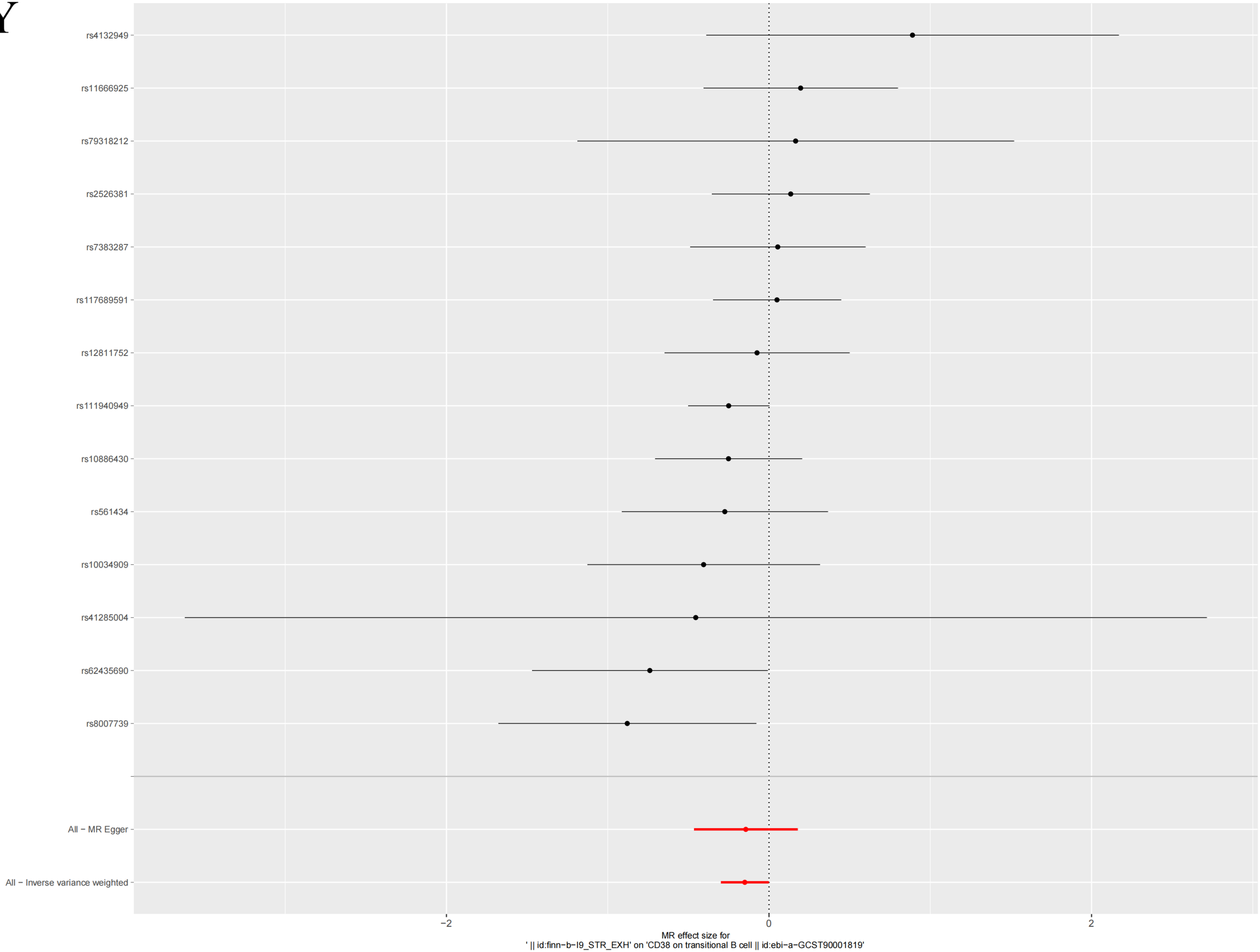

rs4132949
rs11666925
rs79318212
rs2526381
rs7383287
rs117689591
rs12811752
rs111940949
rs10886430
rs561434
rs10034909
rs41285004
rs62435690
rs8007739
All − MR Egger
All − Inverse variance weighted
−2
0
2
MR effect size for
' || id:finn−b−I9_STR_EXH' on 'CD38 on transitional B cell || id:ebi−a−GCST90001819'

Z

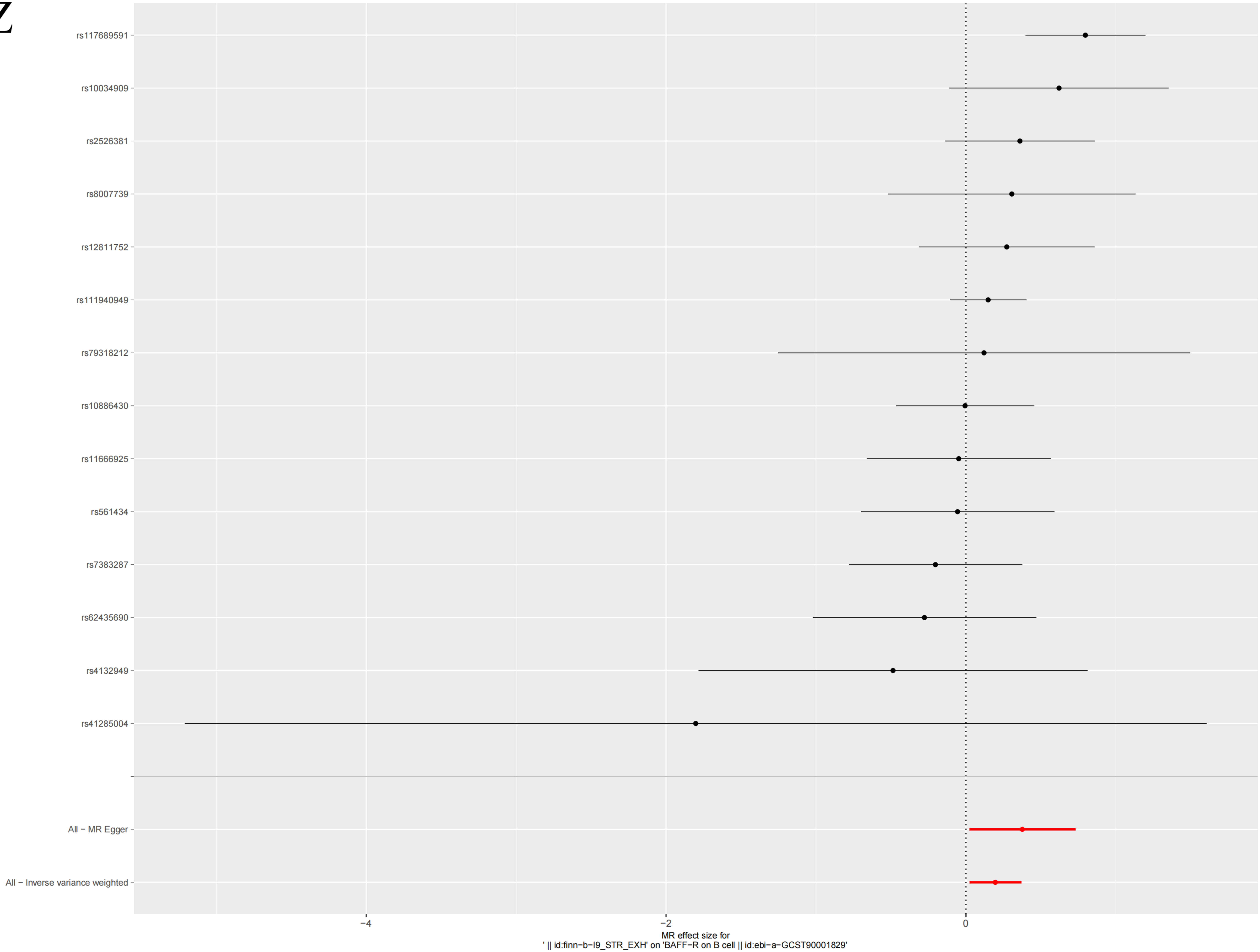
rs117689591
rs10034909
rs2526381
rs8007739
rs12811752
rs111940949
rs79318212
rs10886430
rs11666925
rs561434
rs7383287
rs62435690
rs4132949
rs41285004
All − MR Egger
All − Inverse variance weighted
−4
−2
0
MR effect size for
' || id:finn−b−I9_STR_EXH' on 'BAFF−R on B cell || id:ebi−a−GCST90001829'

AA

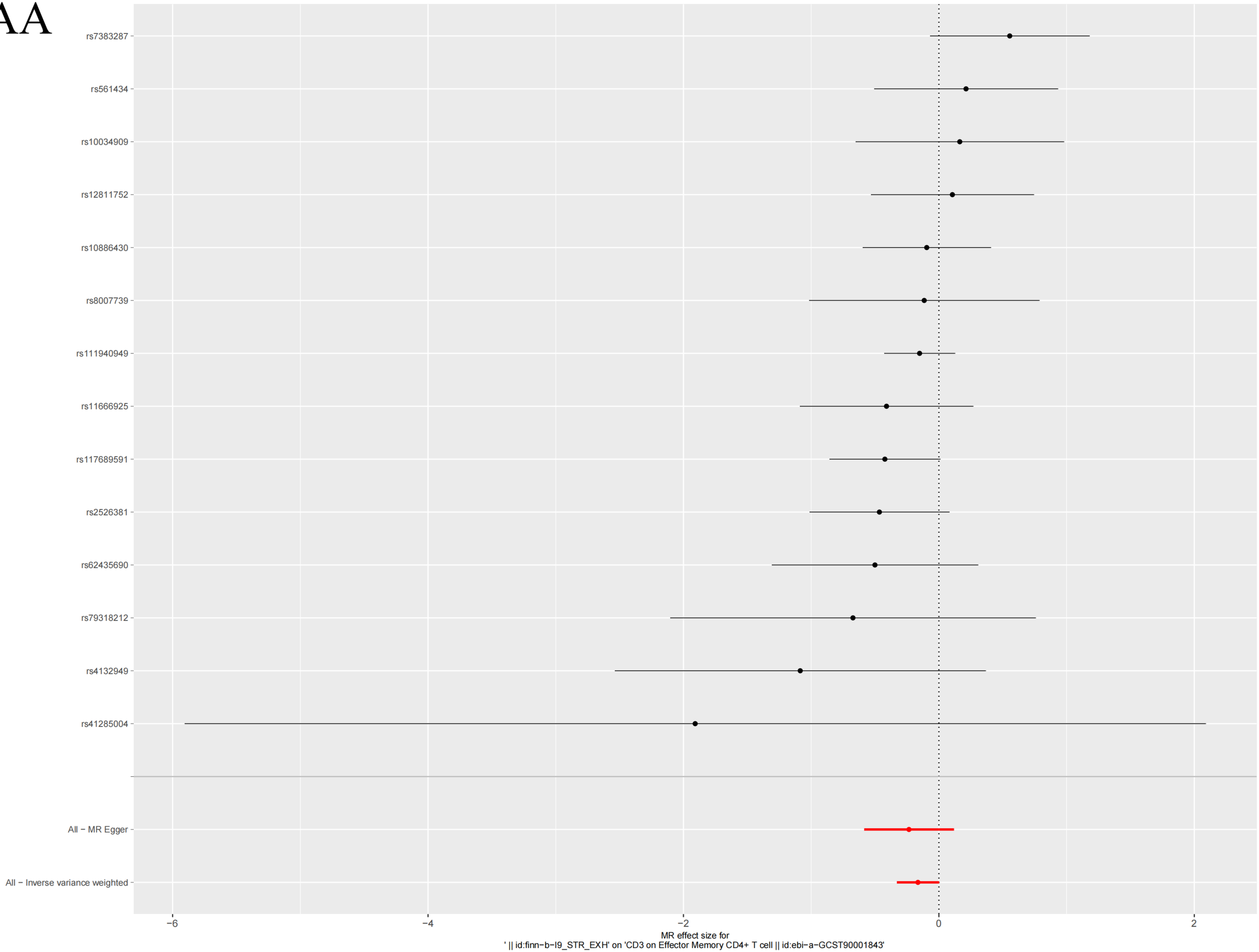

rs7383287
rs561434
rs10034909
rs12811752
rs10886430
rs8007739
rs111940949
rs11666925
rs117689591
rs2526381
rs62435690
rs79318212
rs4132949
rs41285004
All − MR Egger
All − Inverse variance weighted
−6
−4
−2
0
2
MR effect size for
' || id:finn−b−I9_STR_EXH' on 'CD3 on Effector Memory CD4+ T cell || id:ebi−a−GCST90001843'

AB

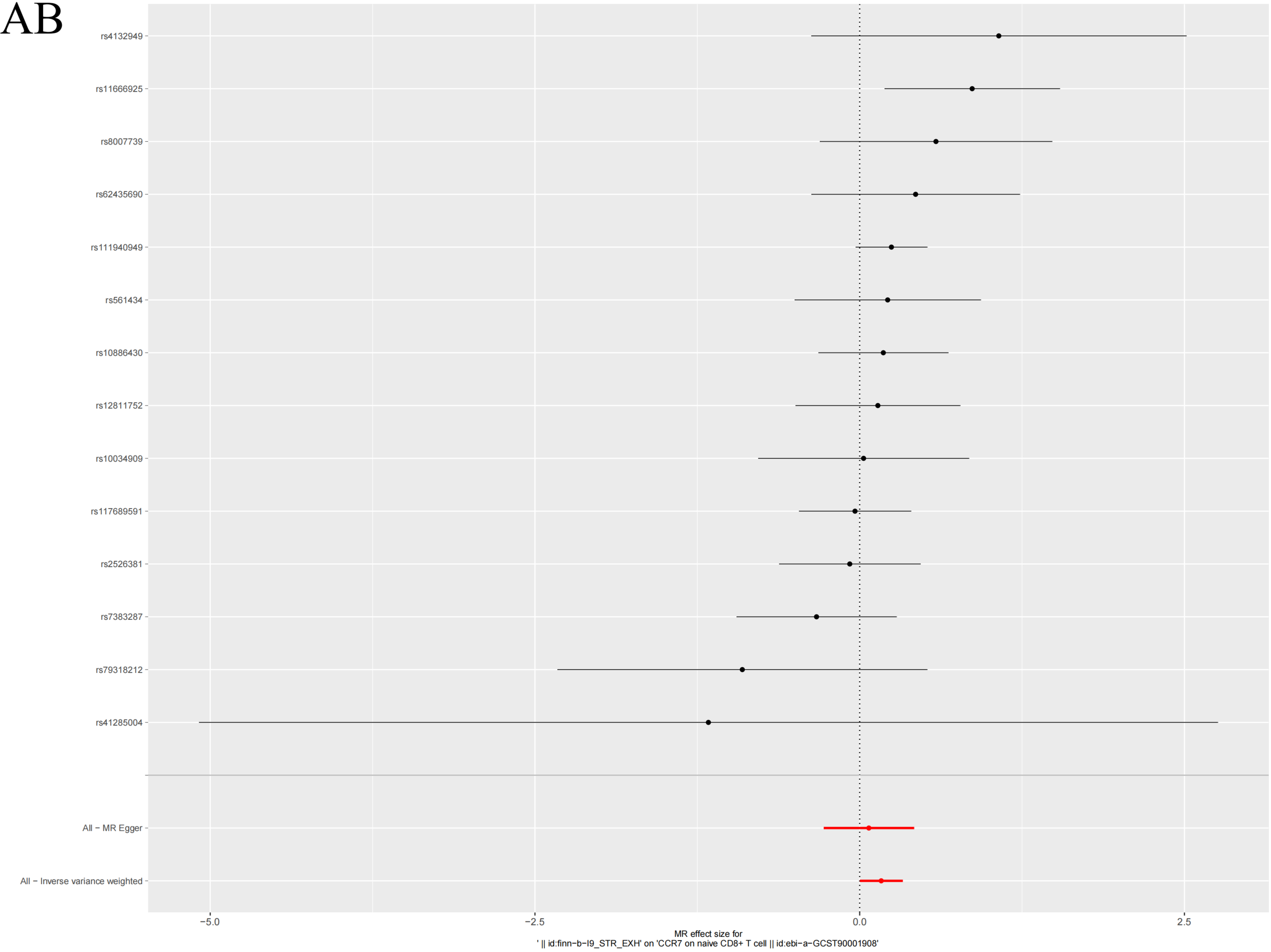

rs4132949
rs11666925
rs8007739
rs62435690
rs111940949
rs561434
rs10886430
rs12811752
rs10034909
rs117689591
rs2526381
rs7383287
rs79318212
rs41285004
All − MR Egger
All − Inverse variance weighted
−5.0
−2.5
0.0
2.5
MR effect size for
' || id:finn−b−I9_STR_EXH' on 'CCR7 on naive CD8+ T cell || id:ebi−a−GCST90001908'

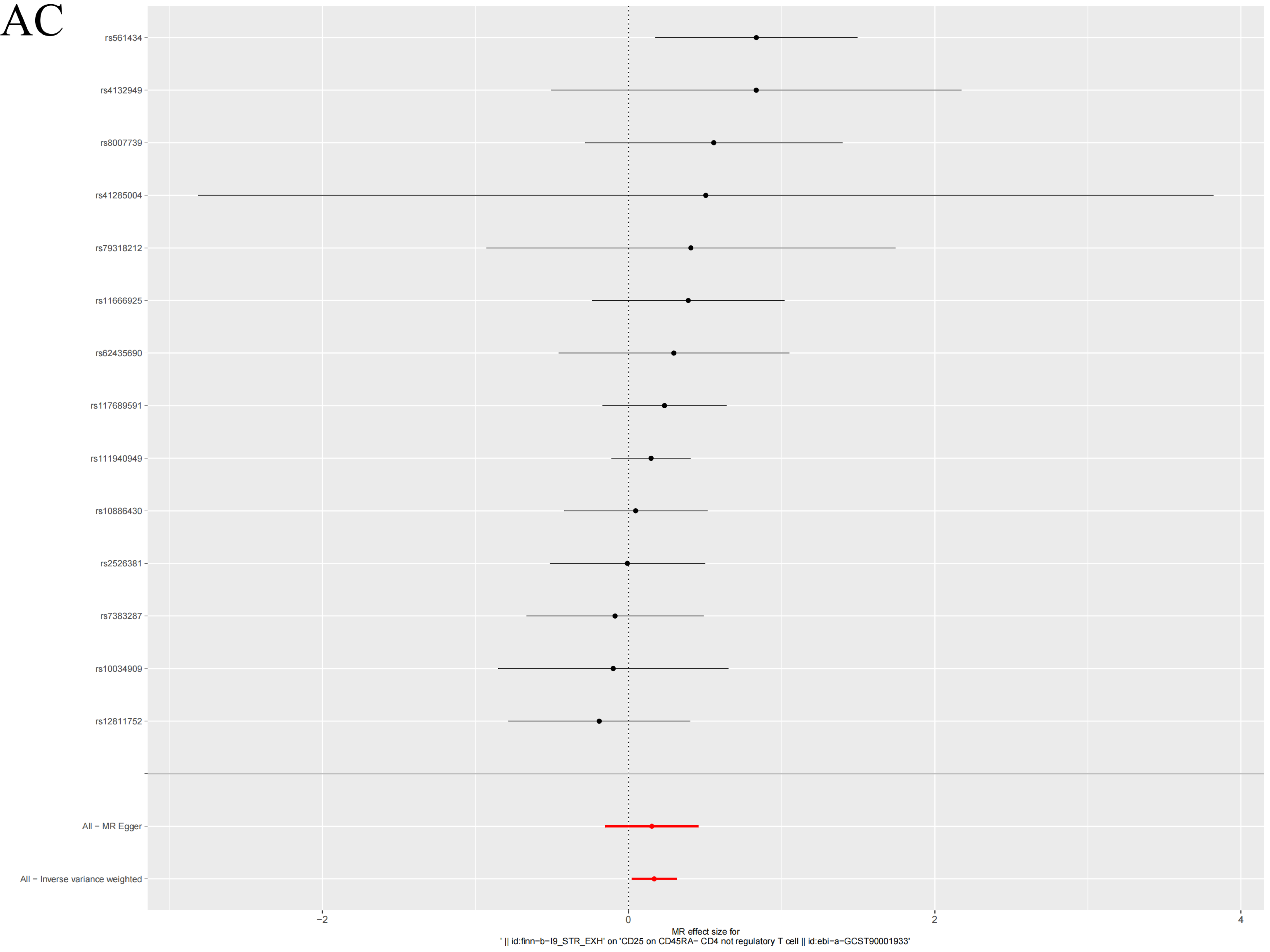
AC
rs561434
rs4132949
rs8007739
rs41285004
rs79318212
rs11666925
rs62435690
rs117689591
rs111940949
rs10886430
rs2526381
rs7383287
rs10034909
rs12811752
All − MR Egger
All − Inverse variance weighted
−2
0
2
4
MR effect size for
' || id:finn−b−I9_STR_EXH' on 'CD25 on CD45RA− CD4 not regulatory T cell || id:ebi−a−GCST90001933'

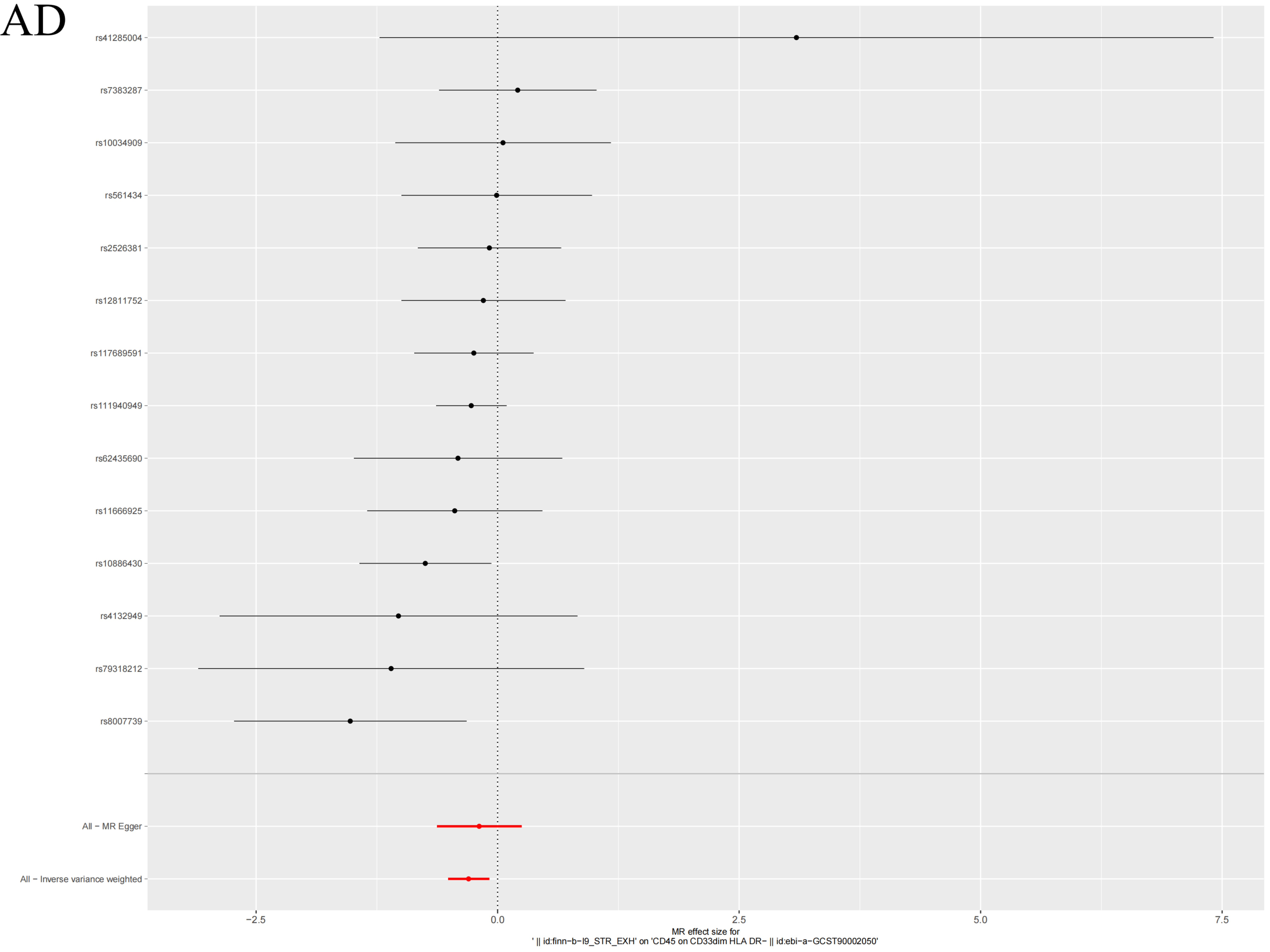
AD
rs41285004
rs7383287
rs10034909
rs561434
rs2526381
rs12811752
rs117689591
rs111940949
rs62435690
rs11666925
rs10886430
rs4132949
rs79318212
rs8007739
All − MR Egger
All − Inverse variance weighted
−2.5
0.0
2.5
5.0
7.5
MR effect size for
' || id:finn−b−I9_STR_EXH' on 'CD45 on CD33dim HLA DR− || id:ebi−a−GCST90002050'

AE

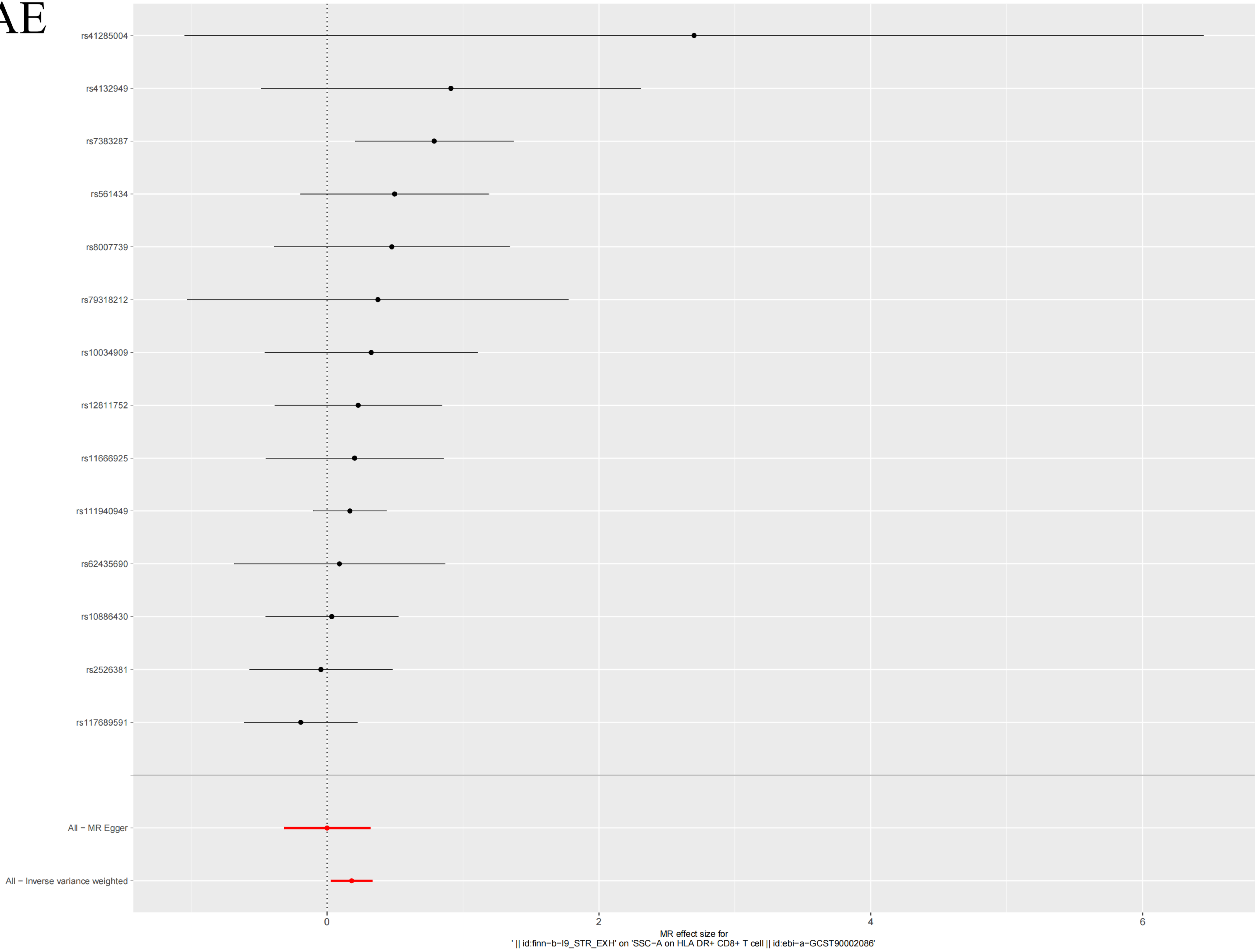
rs41285004
rs4132949
rs7383287
rs561434
rs8007739
rs79318212
rs10034909
rs12811752
rs11666925
rs111940949
rs62435690
rs10886430
rs2526381
rs117689591
All − MR Egger
All − Inverse variance weighted
0
2
4
6
MR effect size for
' || id:finn−b−I9_STR_EXH' on 'SSC−A on HLA DR+ CD8+ T cell || id:ebi−a−GCST90002086'

AF

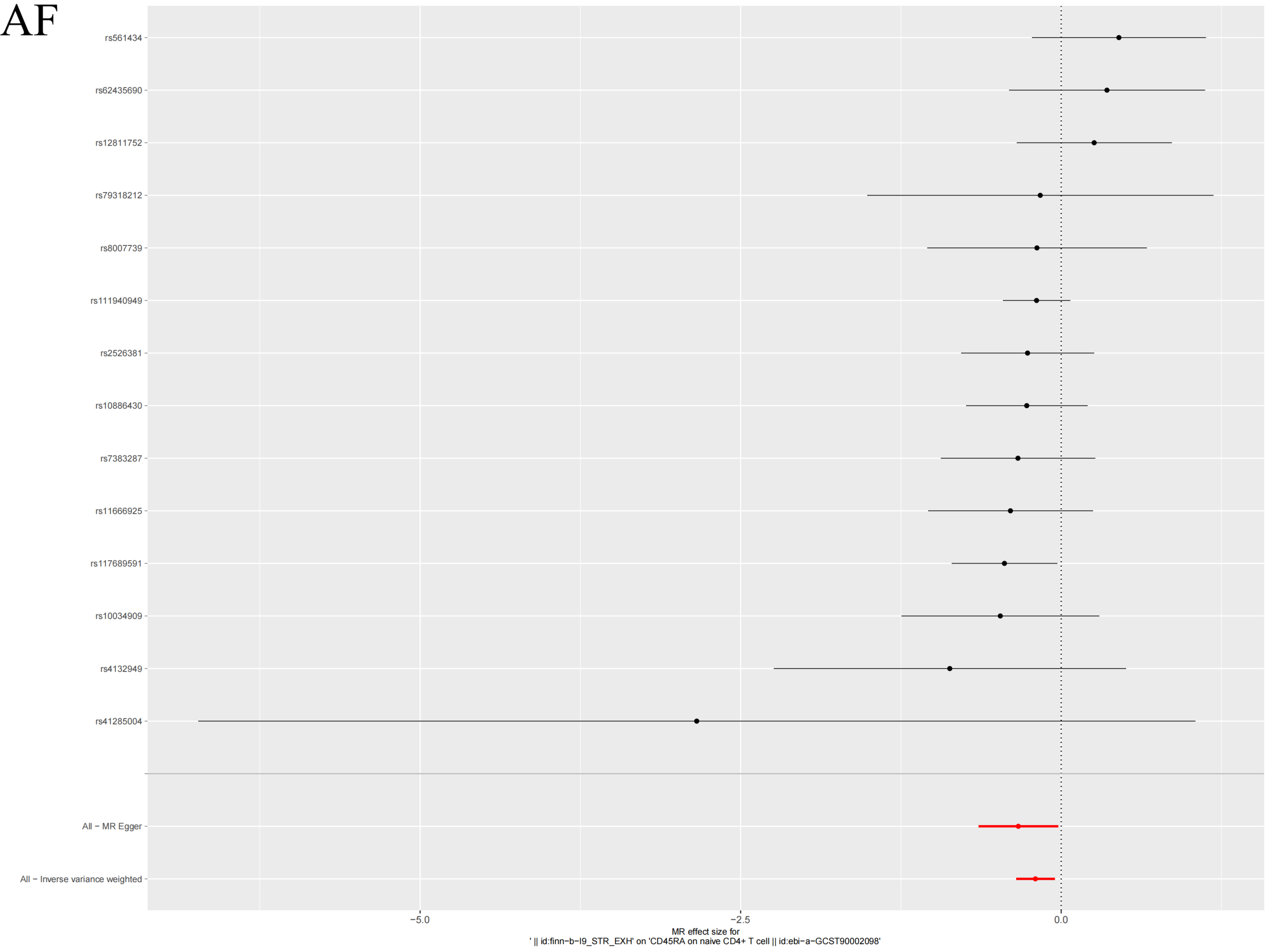
rs561434
rs62435690
rs12811752
rs79318212
rs8007739
rs111940949
rs2526381
rs10886430
rs7383287
rs11666925
rs117689591
rs10034909
rs4132949
rs41285004
All − MR Egger
All − Inverse variance weighted
−5.0
−2.5
0.0
MR effect size for
' || id:finn−b−I9_STR_EXH' on 'CD45RA on naive CD4+ T cell || id:ebi−a−GCST90002098'

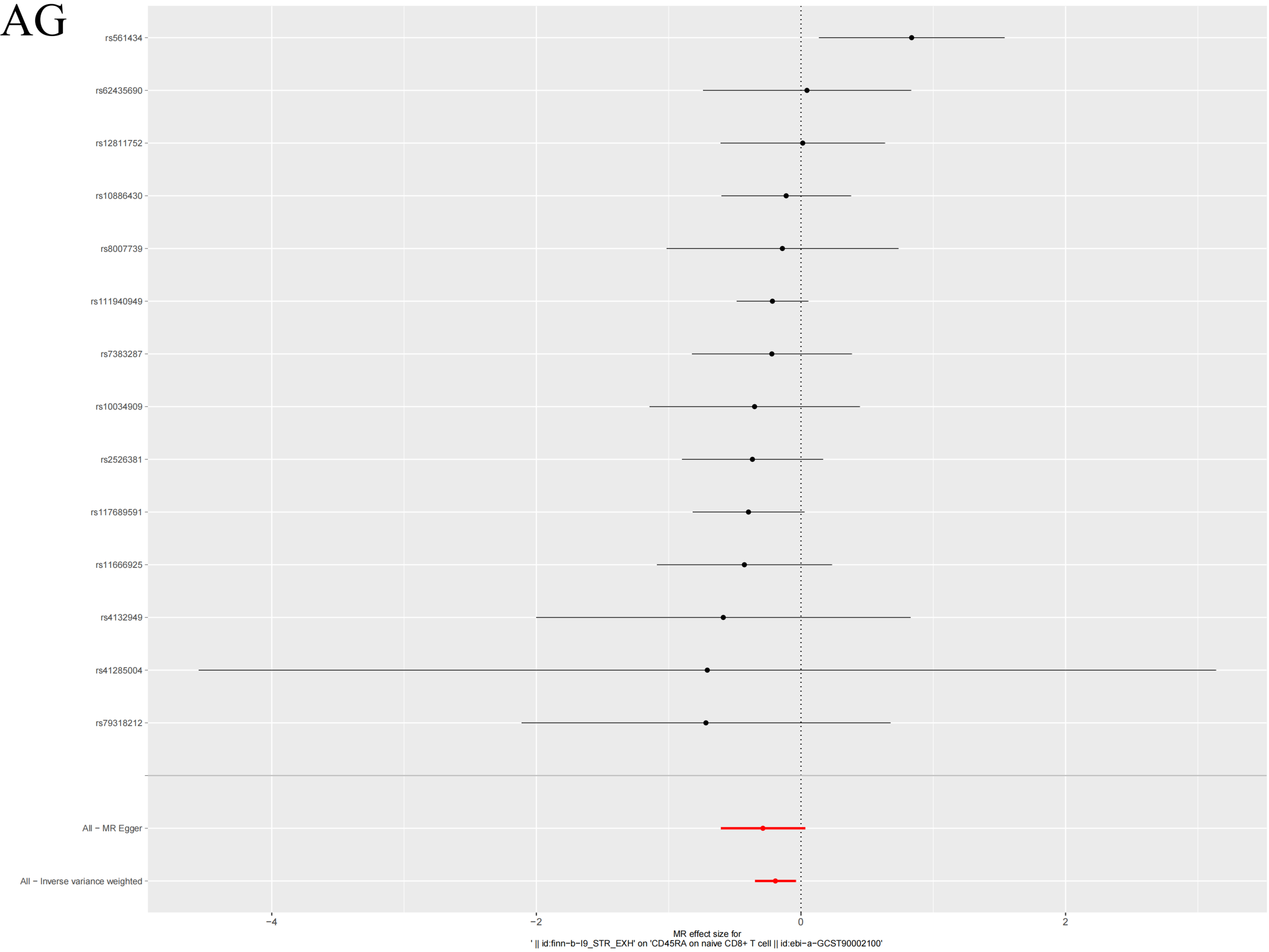
AG
rs561434
rs62435690
rs12811752
rs10886430
rs8007739
rs111940949
rs7383287
rs10034909
rs2526381
rs117689591
rs11666925
rs4132949
rs41285004
rs79318212
All − MR Egger
All − Inverse variance weighted
−4
−2
0
2
MR effect size for
' || id:finn−b−I9_STR_EXH' on 'CD45RA on naive CD8+ T cell || id:ebi−a−GCST90002100'

AH

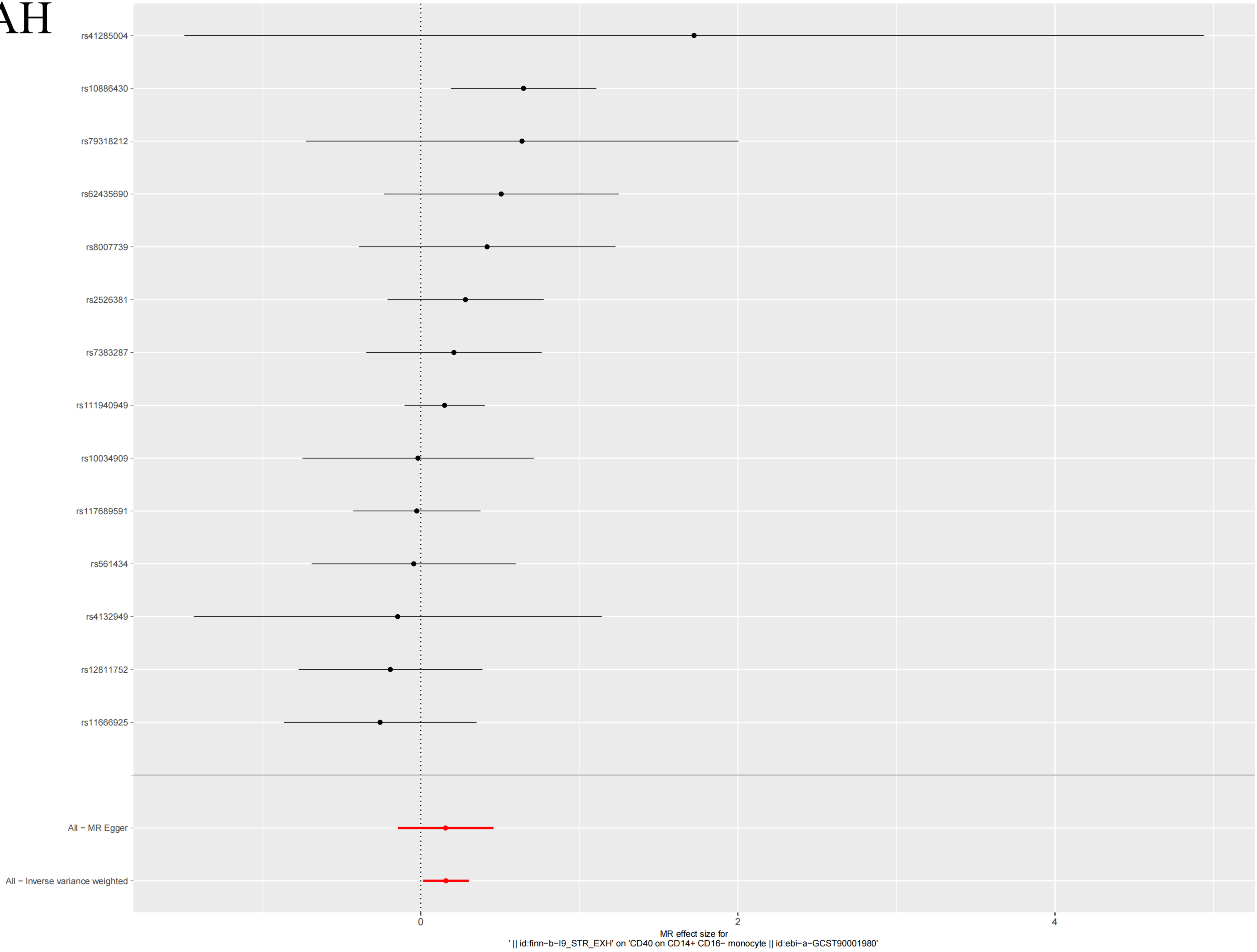

rs41285004
rs10886430
rs79318212
rs62435690
rs8007739
rs2526381
rs7383287
rs111940949
rs10034909
rs117689591
rs561434
rs4132949
rs12811752
rs11666925
All − MR Egger
All − Inverse variance weighted
0
2
4
MR effect size for
' || id:finn−b−I9_STR_EXH' on 'CD40 on CD14+ CD16− monocyte || id:ebi−a−GCST90001980'

AI

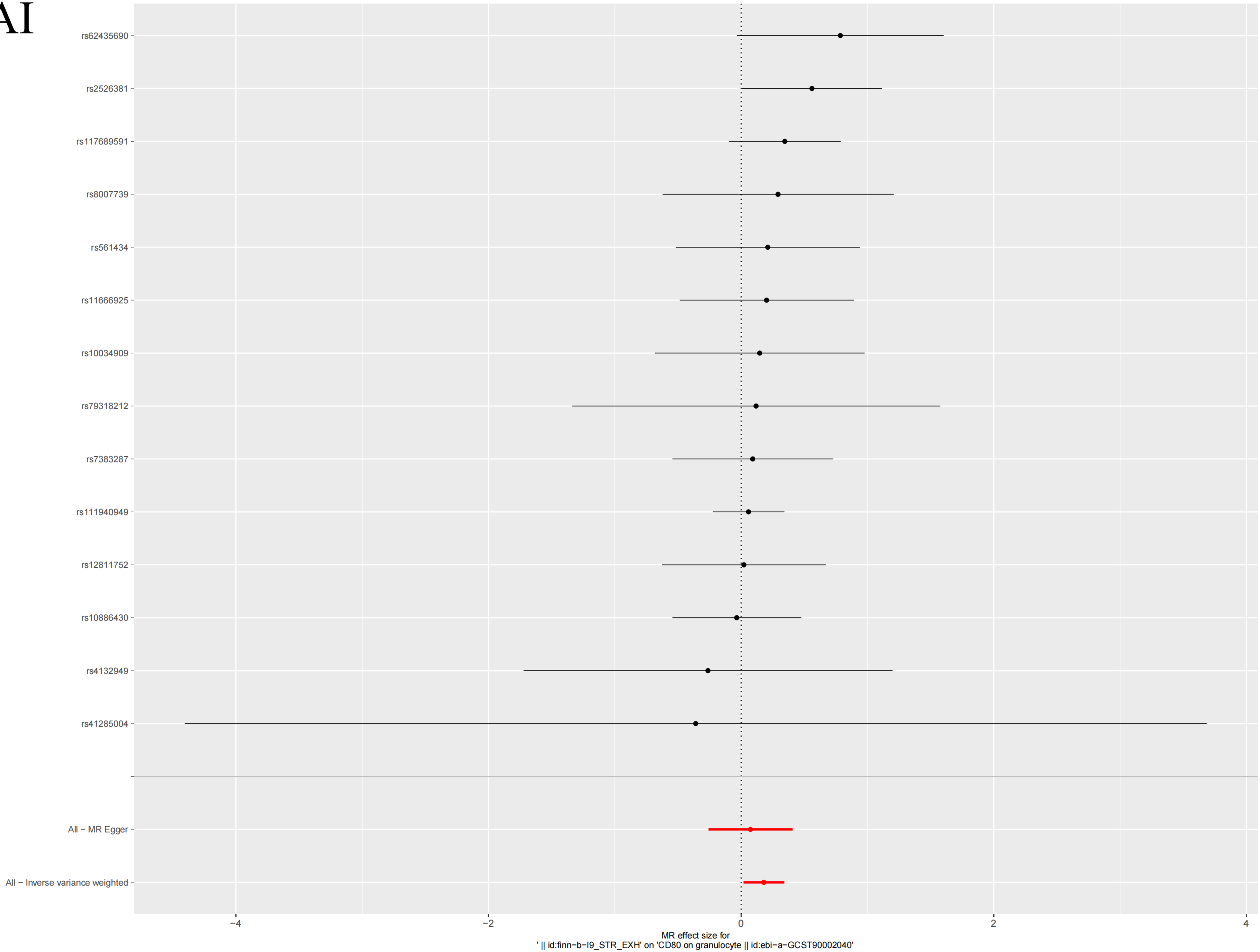

rs62435690
rs2526381
rs117689591
rs8007739
rs561434
rs11666925
rs10034909
rs79318212
rs7383287
rs111940949
rs12811752
rs10886430
rs4132949
rs41285004
All − MR Egger
All − Inverse variance weighted
−4
−2
0
2
4
MR effect size for
' || id:finn−b−I9_STR_EXH' on 'CD80 on granulocyte || id:ebi−a−GCST90002040'

AJ

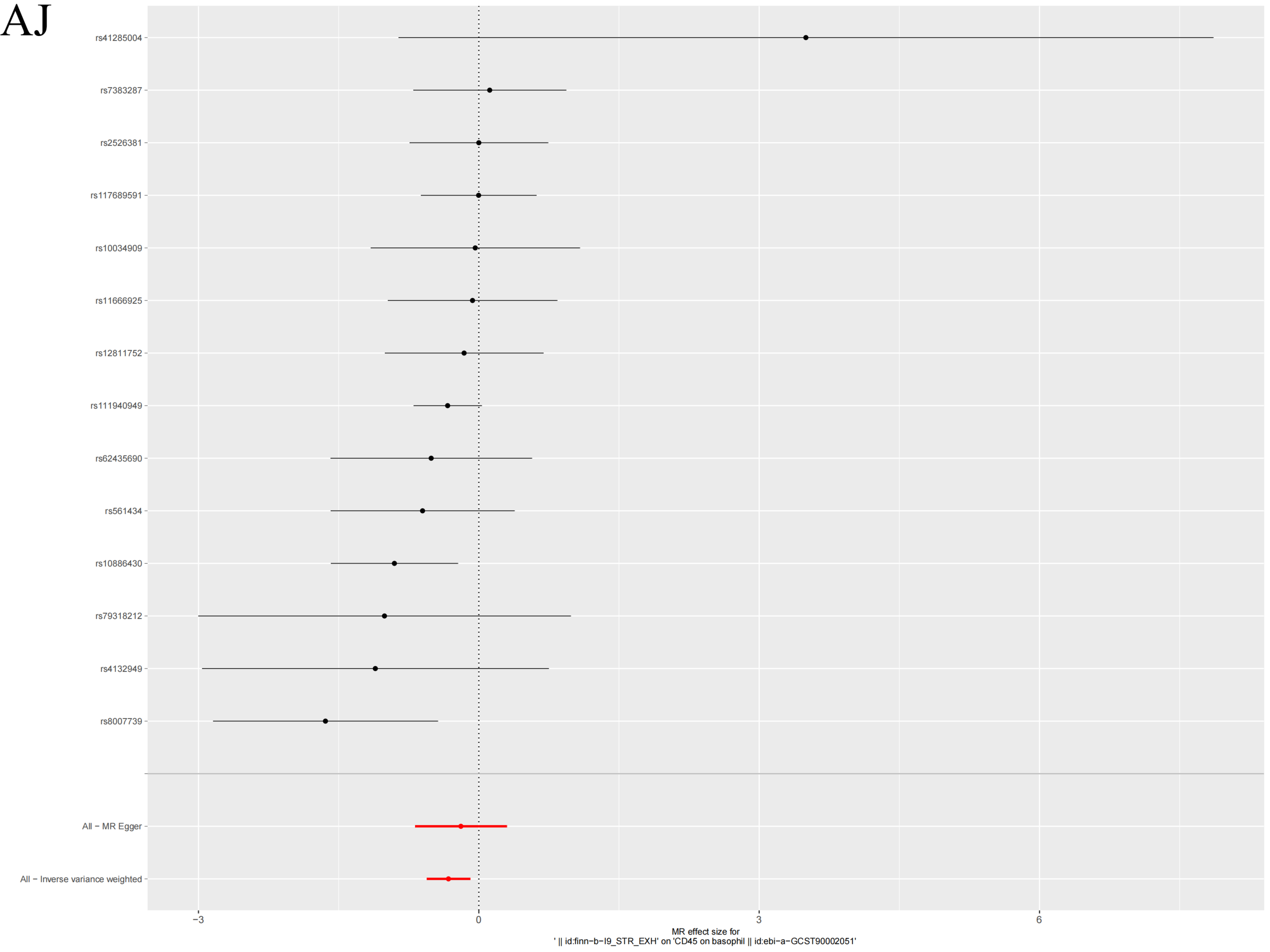
rs41285004
rs7383287
rs2526381
rs117689591
rs10034909
rs11666925
rs12811752
rs111940949
rs62435690
rs561434
rs10886430
rs79318212
rs4132949
rs8007739
All − MR Egger
All − Inverse variance weighted
−3
0
3
6
MR effect size for
' || id:finn-b-I9_STR_EXH' on 'CD45 on basophil || id:ebi-a-GCST90002051'

AK

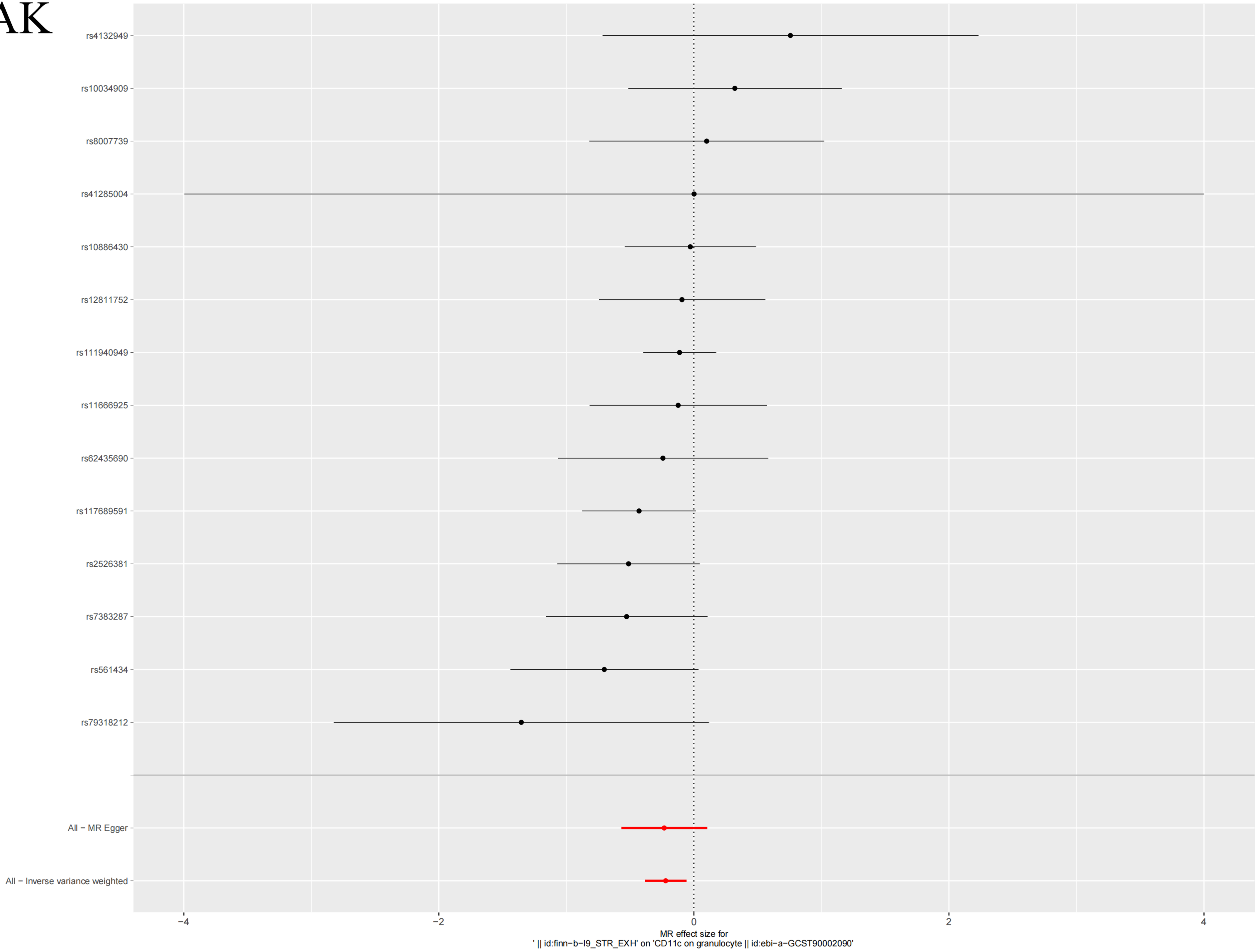
rs4132949
rs10034909
rs8007739
rs41285004
rs10886430
rs12811752
rs111940949
rs11666925
rs62435690
rs117689591
rs2526381
rs7383287
rs561434
rs79318212
All − MR Egger
All − Inverse variance weighted
−4
−2
0
2
4
MR effect size for
' || id:finn−b−I9_STR_EXH' on 'CD11c on granulocyte || id:ebi−a−GCST90002090'

Supplementary Figure 4. Sensitivity analysis of reverse Mendelian randomization (Funnel plot).

A. Funnel plot between IS and BAFF-R on CD24+ CD27+ B cell; B. Funnel plot between IS and BAFF-R on IgD+ CD24+ B cell; C. Funnel plot between IS and BAFF-R on IgD+ CD24- B cell; D. Funnel plot between IS and BAFF-R on IgD+ CD38- B cell; E. Funnel plot between IS and BAFF-R on IgD+ CD38- naive B cell; F. Funnel plot between IS and BAFF-R on IgD+ CD38- unswitched memory B cell; G. Funnel plot between IS and BAFF-R on IgD+ CD38+ B cell; H. Funnel plot between IS and BAFF-R on IgD+ CD38dim B cell; I. Funnel plot between IS and BAFF-R on IgD- CD24- B cell; J. Funnel plot between IS and BAFF-R on IgD- CD27- B cell; K. Funnel plot between IS and BAFF-R on IgD- CD38- B cell; L. Funnel plot between IS and BAFF-R on IgD- CD38dim B cell; M. Funnel plot between IS and BAFF-R on memory B cell; N. Funnel plot between IS and BAFF-R on naive-mature B cell; O. Funnel plot between IS and BAFF-R on unswitched memory B cell; P. Funnel plot between IS and BAFF-R on switched memory B cell; Q. Funnel plot between IS and BAFF-R on IgD+ B cell; R. Funnel plot between IS and BAFF-R on transitional B cell; S. Funnel plot between IS and CD19 on IgD+ CD24- B cell; T. Funnel plot between IS and CD19 on IgD+ CD38- naive B cell; U. Funnel plot between IS and CD19 on naive-mature B cell; V. Funnel plot between IS and CD19 on transitional B cell; W. Funnel plot between IS and CD25 on IgD+ CD38+ B cell; X. Funnel plot between IS and CD25 on transitional B cell; Y. Funnel plot between IS and CD38 on transitional B cell; Z. Funnel plot between IS and BAFF-R on B cell; AA. Funnel plot between IS and CD3 on Effector Memory CD4+ T cell; AB. Funnel plot between IS and CCR7 on naive CD8+ T cell; AC. Funnel plot between IS and CD25 on CD45RA- CD4 not regulatory T cell; AD. Funnel plot between IS and CD45 on CD33dim HLA DR-; AE. Funnel plot between IS and SSC-A on HLA DR+ CD8+ T cell; AF. Funnel plot between IS and CD45RA on naive CD4+ T cell; AG. Funnel plot between IS and CD45RA on naive CD8+ T cell; AH. Funnel plot between IS and CD40 on CD14+ CD16- monocyte; AI. Funnel plot between IS and CD80 on granulocyte; AJ. Funnel plot between IS and CD45 on basophil; AK. Funnel plot between IS and CD11c on granulocyte.
